# Supplementary figures and images for: Targeting oncogenic KRasG13C with nucleotide-based covalent inhibitors
Source: eLife. 2023 Mar 27;12:e82184. doi: 10.7554/eLife.82184 (PMC10042540; doi:10.7554/eLife.82184)

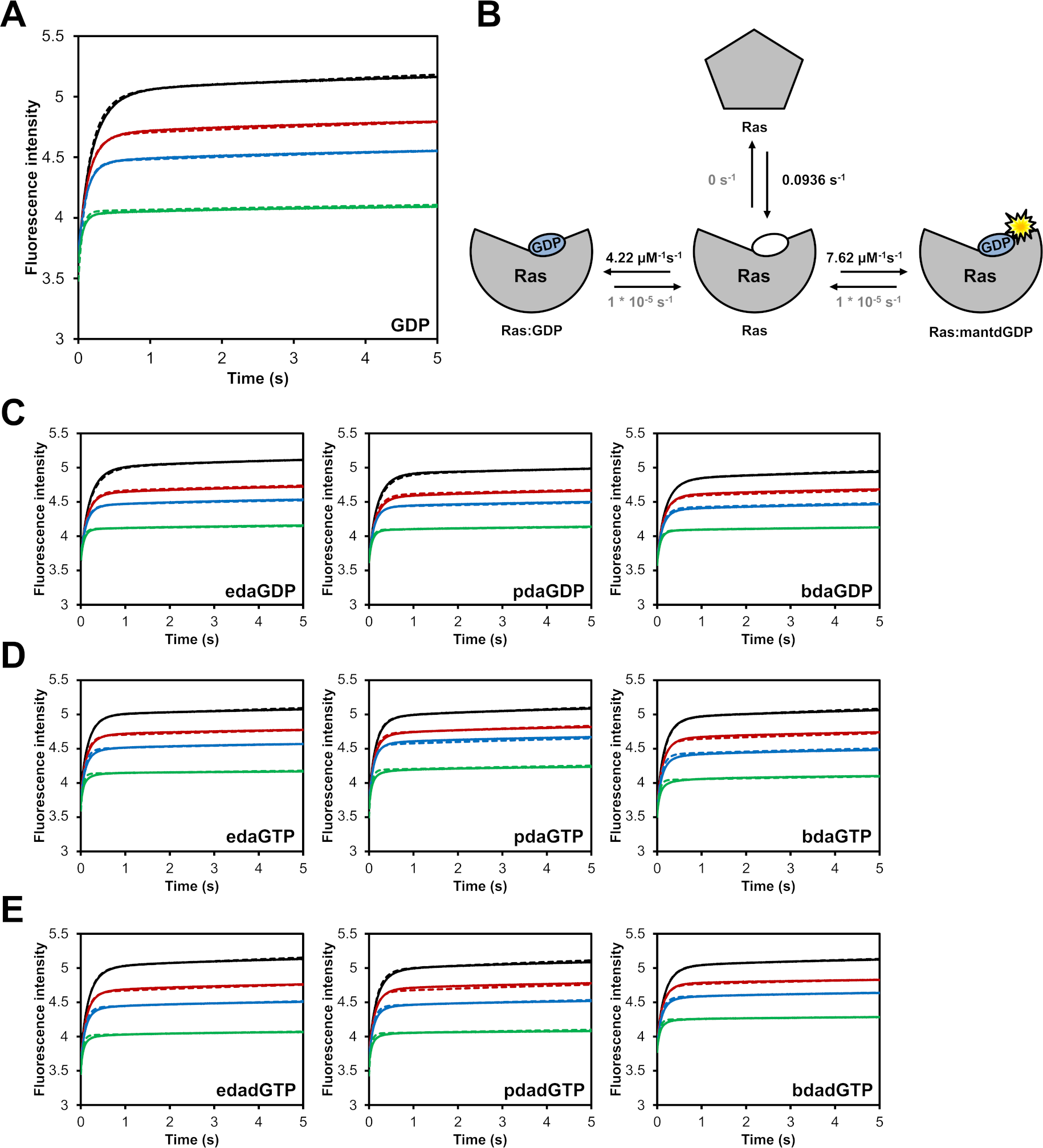

Supplement: Table 1—source data 1. — (A) Competitive binding experiments of GDP and mantdGDP; 1 μM KRas and 2 μM mantdGDP was used in the absence (black curve) or in the presence of 1 µM (red), 2 µM (blue) or 6 µM (green) competing nucleotides in a stopped-flow instrument. (B) The binding curves were globally fit to the indicated model using KinTek Explorer to obtain the corresponding association rates (kon). (C, D, E) Competitive binding experiments between mantdGDP and GDP, GTP, or dGTP analogues. Calculated kon values are shown in Table 1—source data 2. [file elife-82184-table1-data1.zip › Table 1-source data 1.tiff]

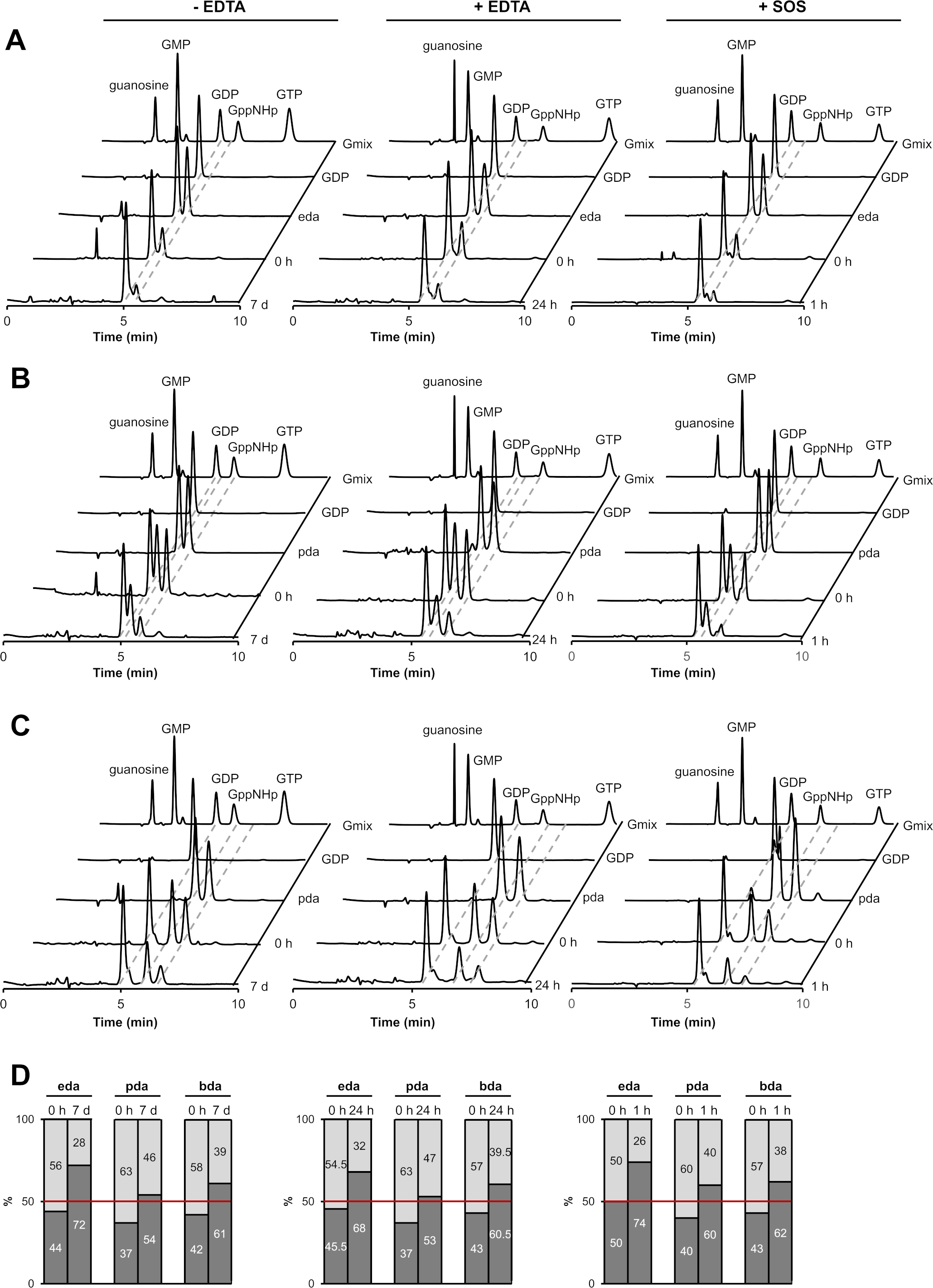

Supplement: Table 1—source data 3. — (A, B, C) Competitive binding experiments of GDP and the acetyl-derivatives of GDP; 50 μM KRasWT:GDP was mixed with 50 µM of edaGDP, pdaGDP or bdaGDP and incubated for 7 days at room temperature in the absence of EDTA, for 24 hr at 4 °C in the presence of 10 mM EDTA or for 1 hr in the presence of SOS (0.5 µM). After buffer exchange for removal of any unbound nucleotides, the resulting mixtures were analyzed by isocratic HPLC runs. (D) Relative amounts of the nucleotides and GDP bound to KRas were compared. It should be noted that the relative affinities mentioned in the method section and shown in Table 1—source data 4 are calculated from these experiments and represent an average affinity of the 2’ isomers, and that of the 3’-isomer seen to be bound in the X-ray structure is presumably actually higher than this, since it can be seen from Figure 2—figure supplement 1 that there is a preference for one of the isomers, presumably the 3’-isomer, so that this species and the derivatives of dGDP/dGTP must have a very similar affinity to that of GDP/GTP. [file elife-82184-table1-data3.zip › Table 1-source data 3.tiff]

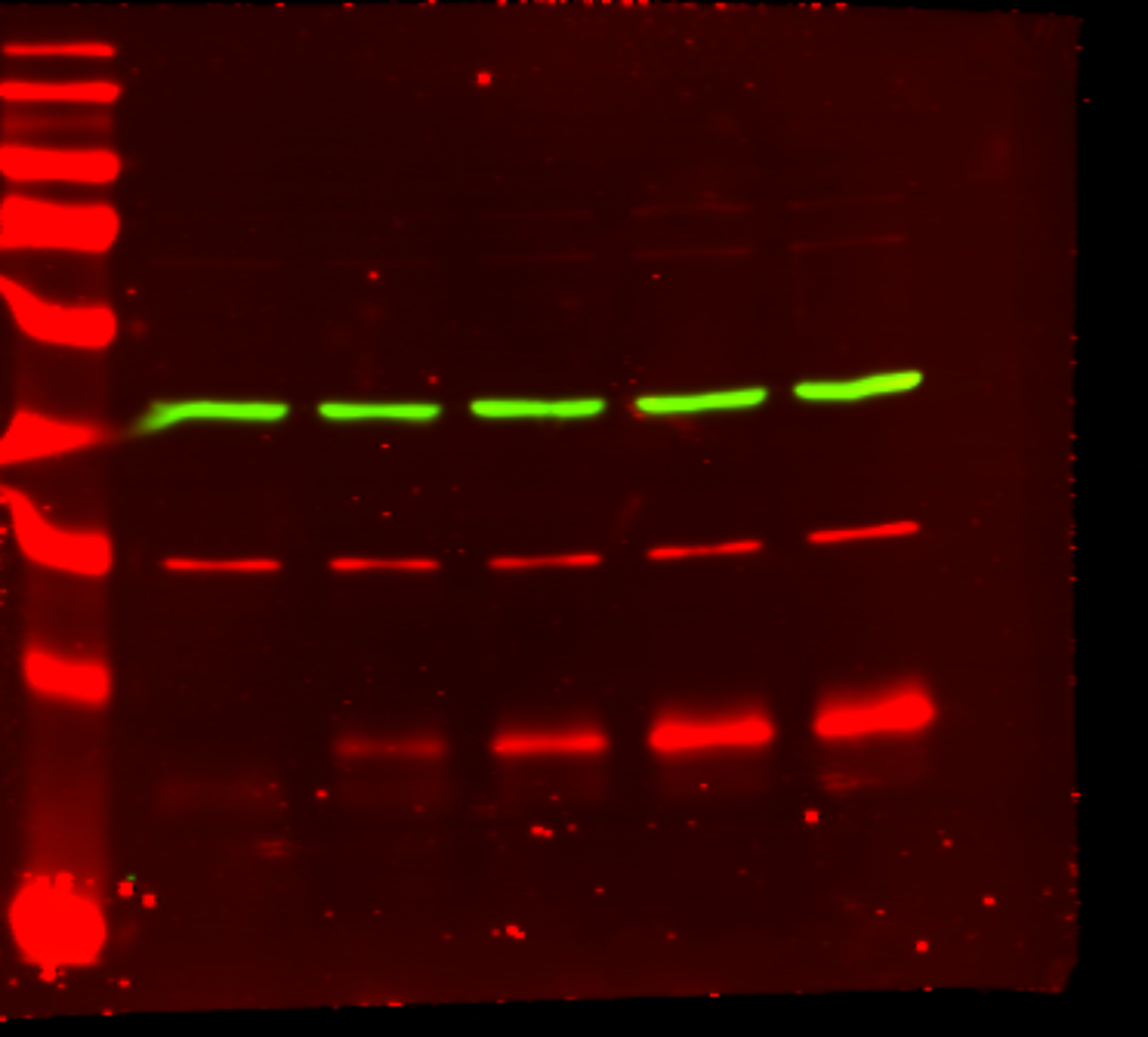

Supplement: Figure 3—source data 1. [file elife-82184-fig3-data1.zip › Figure 3-source data 1/KRasG13C-edaGDP/1_KRasG13C-edaGDP_KRas.tif]

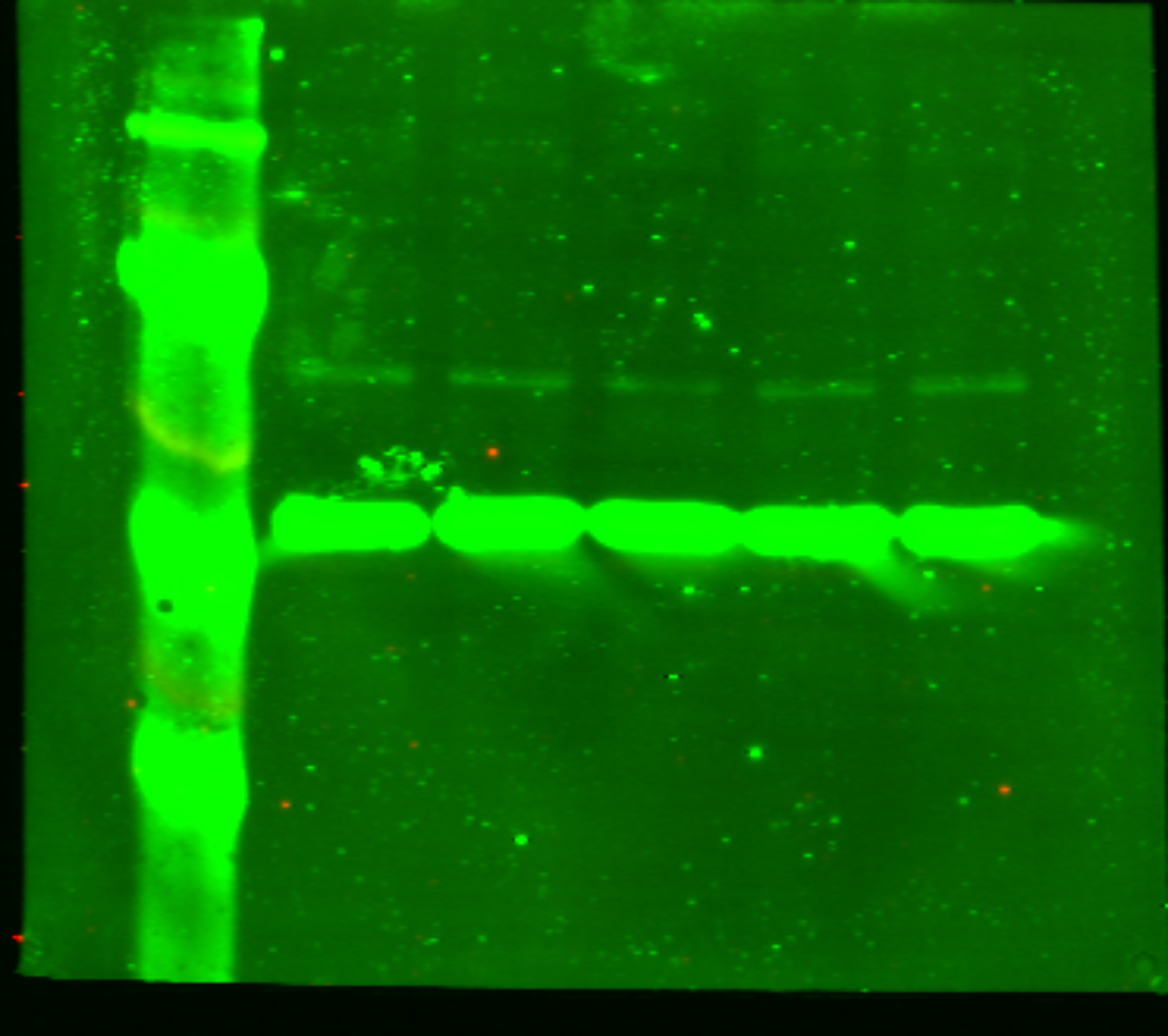

Supplement: Figure 3—source data 1. [file elife-82184-fig3-data1.zip › Figure 3-source data 1/KRasG13C-edaGDP/1_KRasG13C-edaGDP_pAkt.tif]

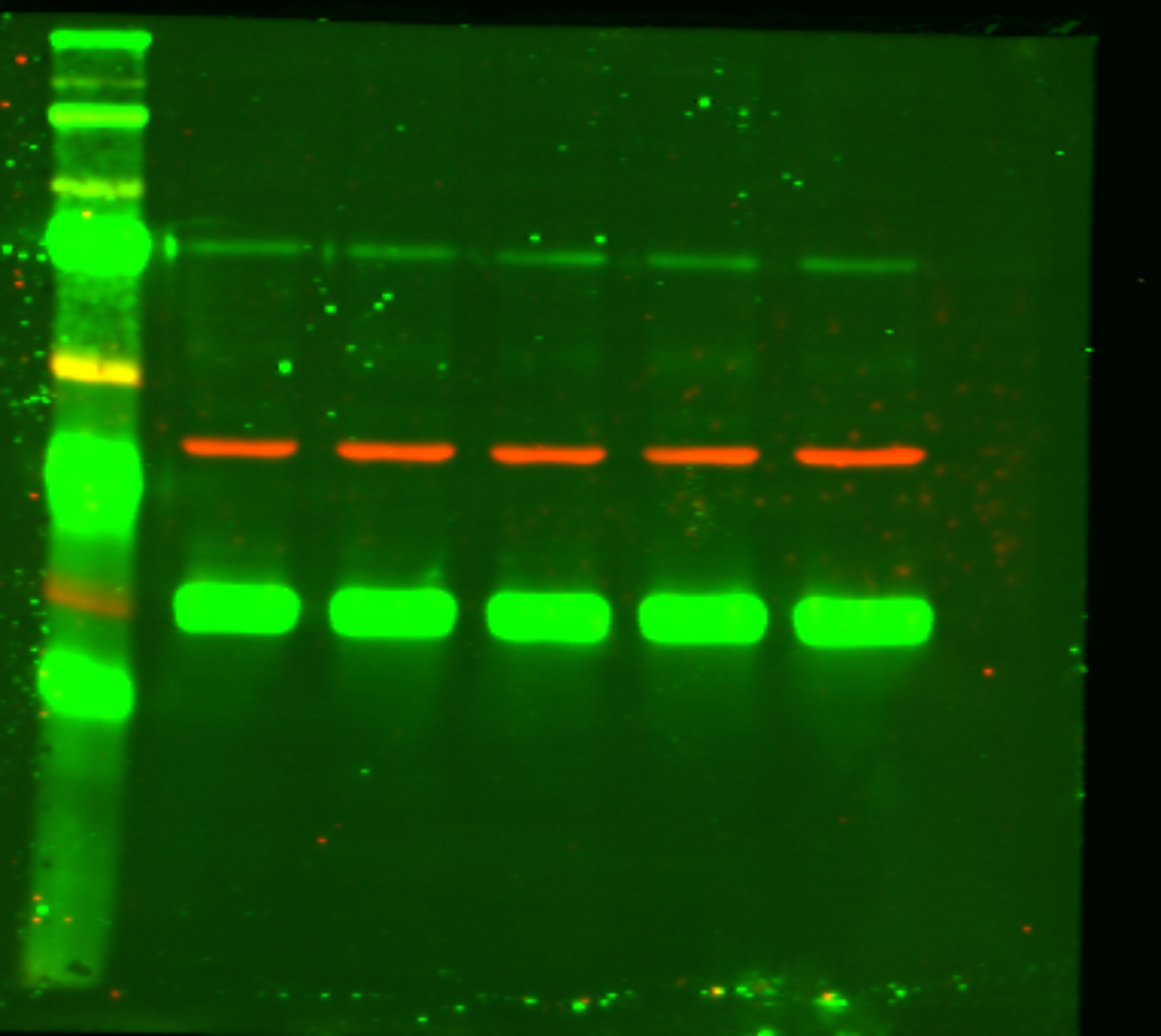

Supplement: Figure 3—source data 1. [file elife-82184-fig3-data1.zip › Figure 3-source data 1/KRasG13C-edaGDP/1_KRasG13C-edaGDP_pcRaf_pS6.tif]

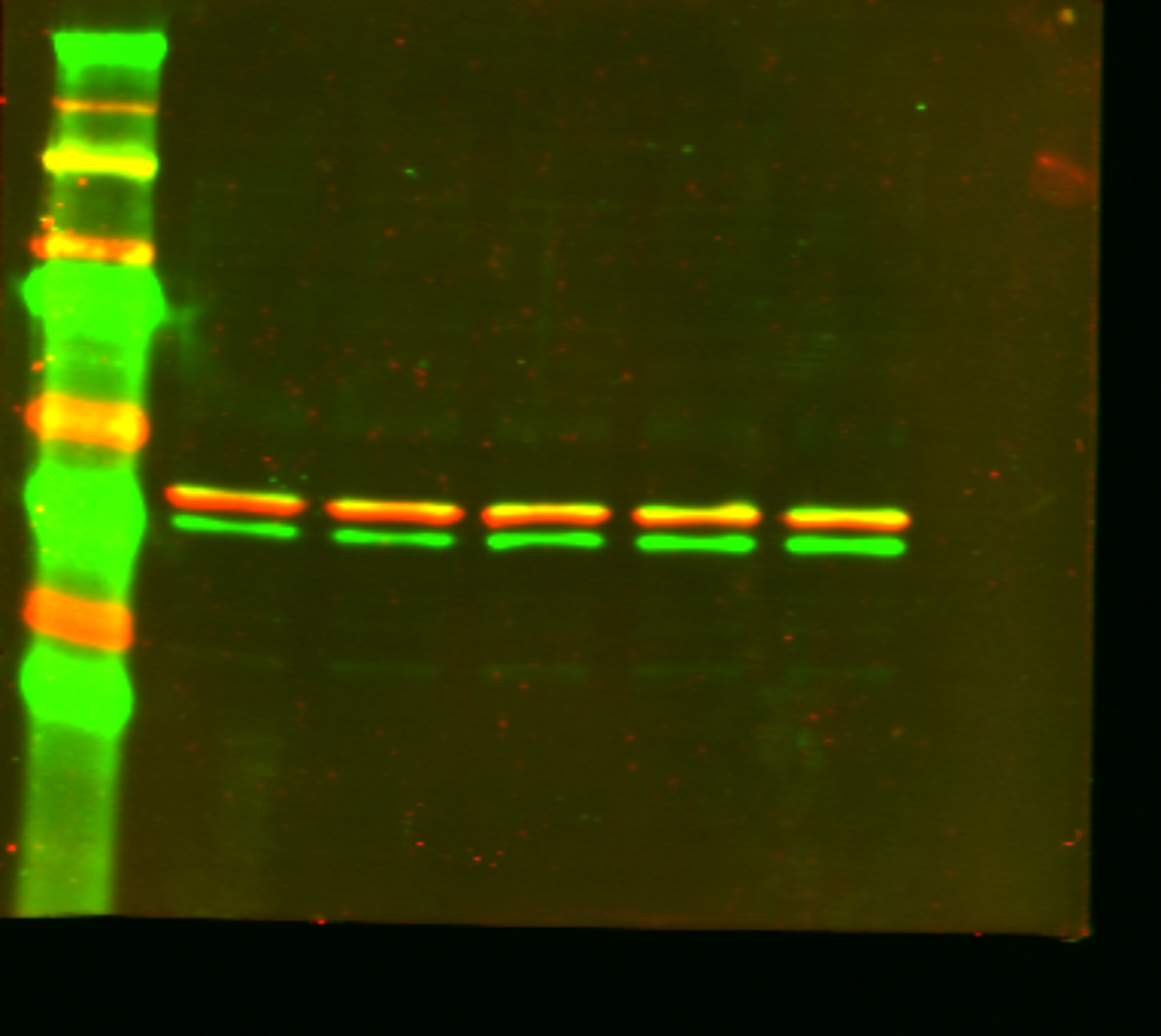

Supplement: Figure 3—source data 1. [file elife-82184-fig3-data1.zip › Figure 3-source data 1/KRasG13C-edaGDP/1_KRasG13C-edaGDP_pErk.tif]

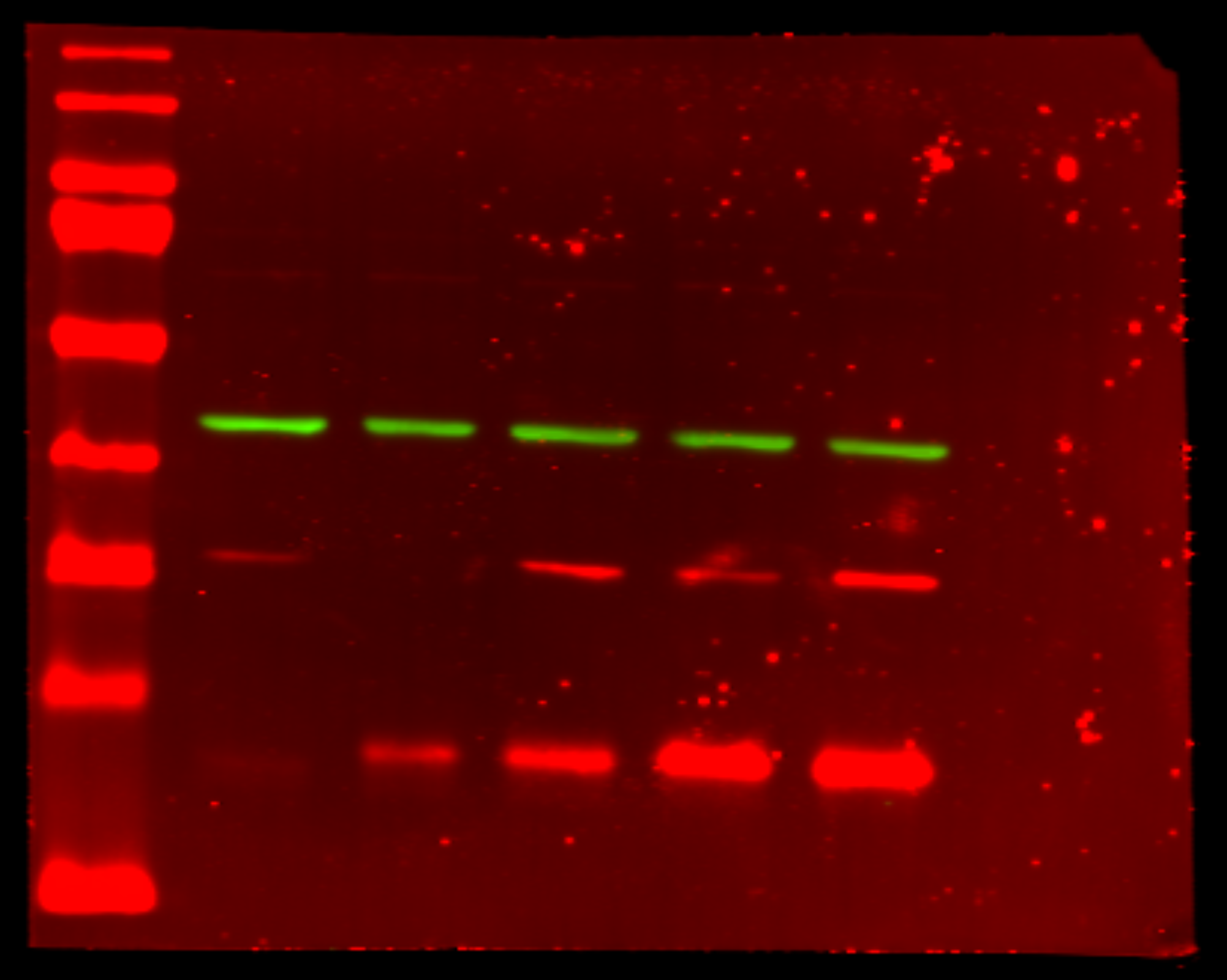

Supplement: Figure 3—source data 1. [file elife-82184-fig3-data1.zip › Figure 3-source data 1/KRasG13C/1_KRasG13C_KRas.tif]

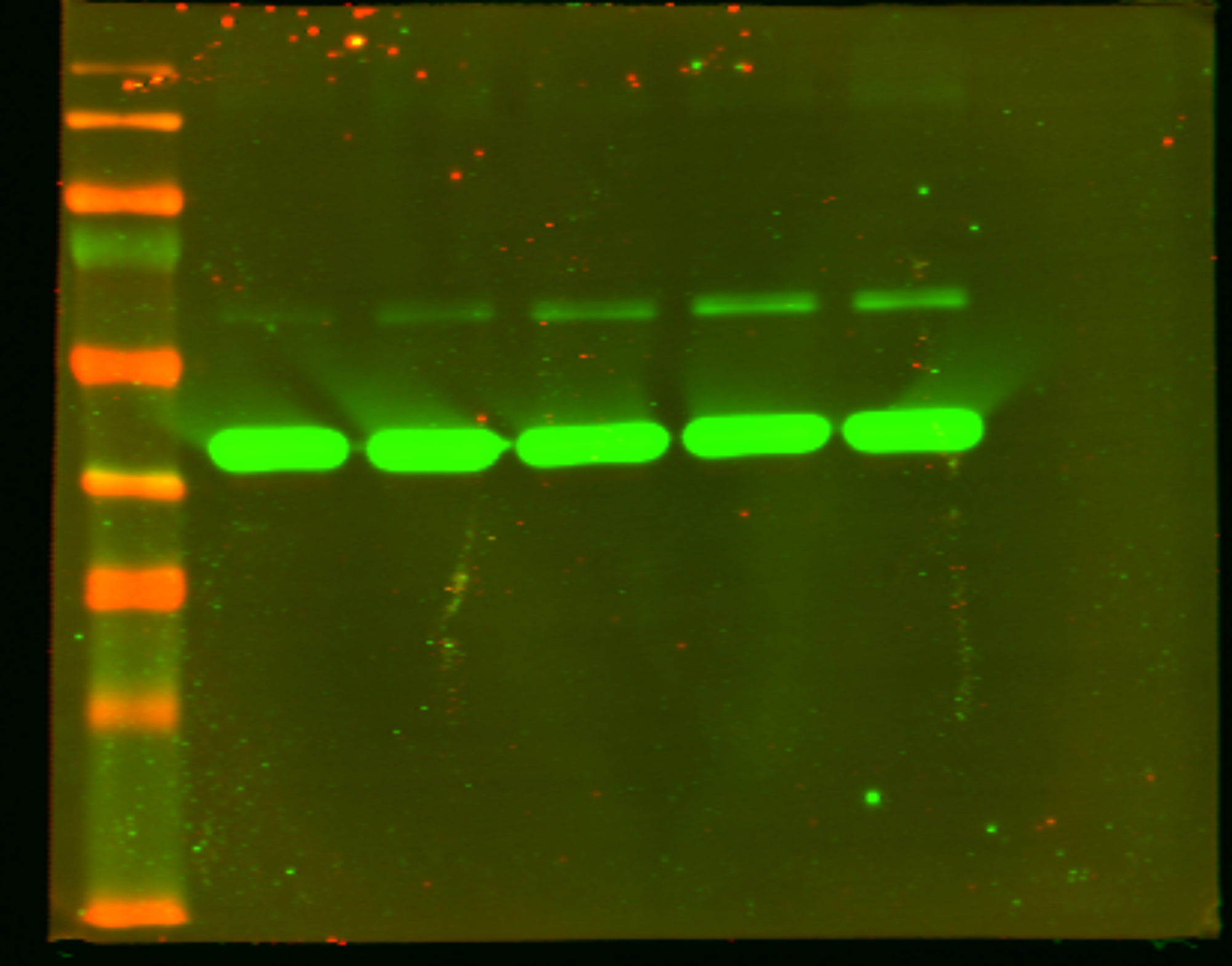

Supplement: Figure 3—source data 1. [file elife-82184-fig3-data1.zip › Figure 3-source data 1/KRasG13C/1_KRasG13C_pAkt.tif]

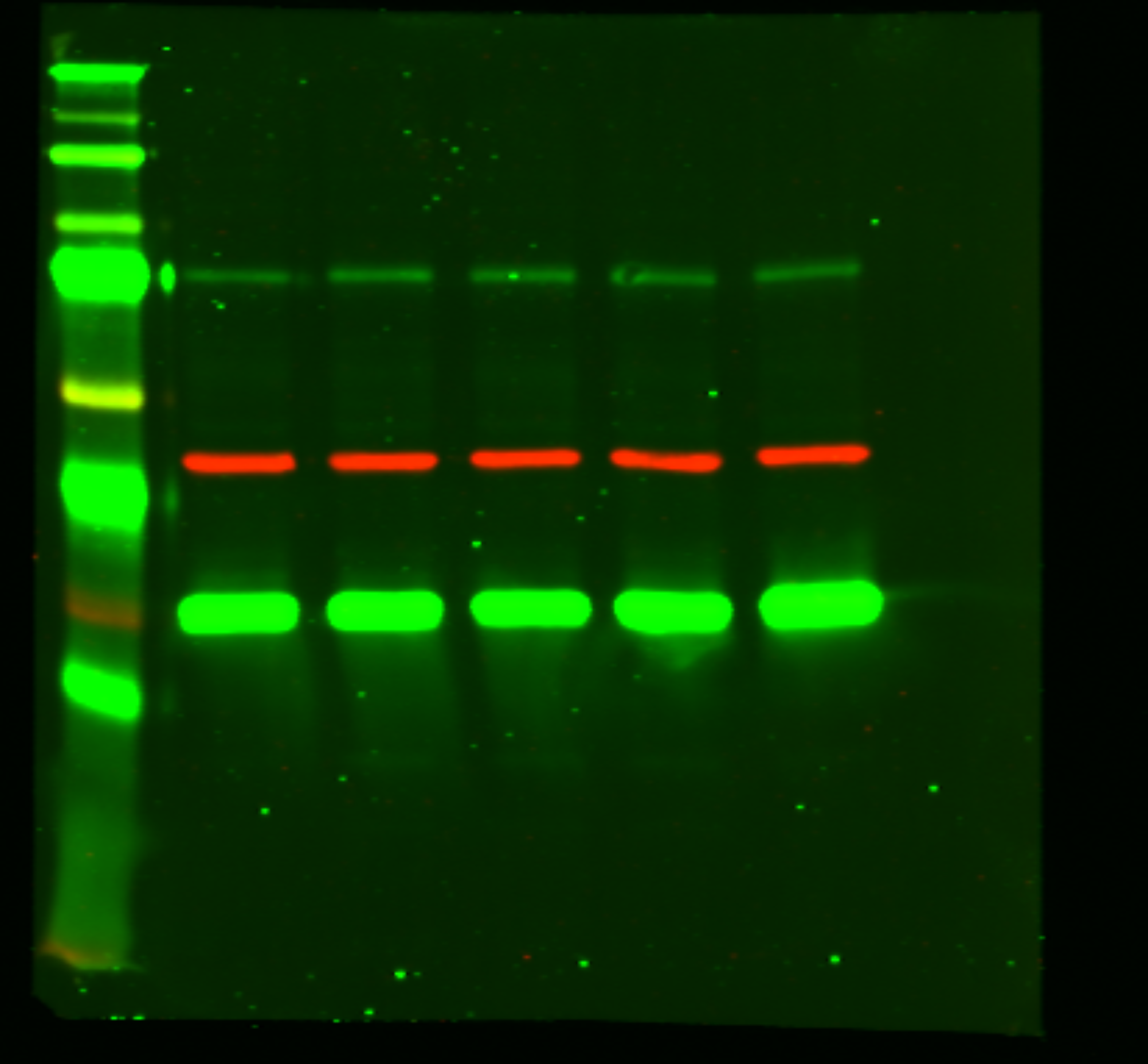

Supplement: Figure 3—source data 1. [file elife-82184-fig3-data1.zip › Figure 3-source data 1/KRasG13C/1_KRasG13C_pcRaf_pS6.tif]

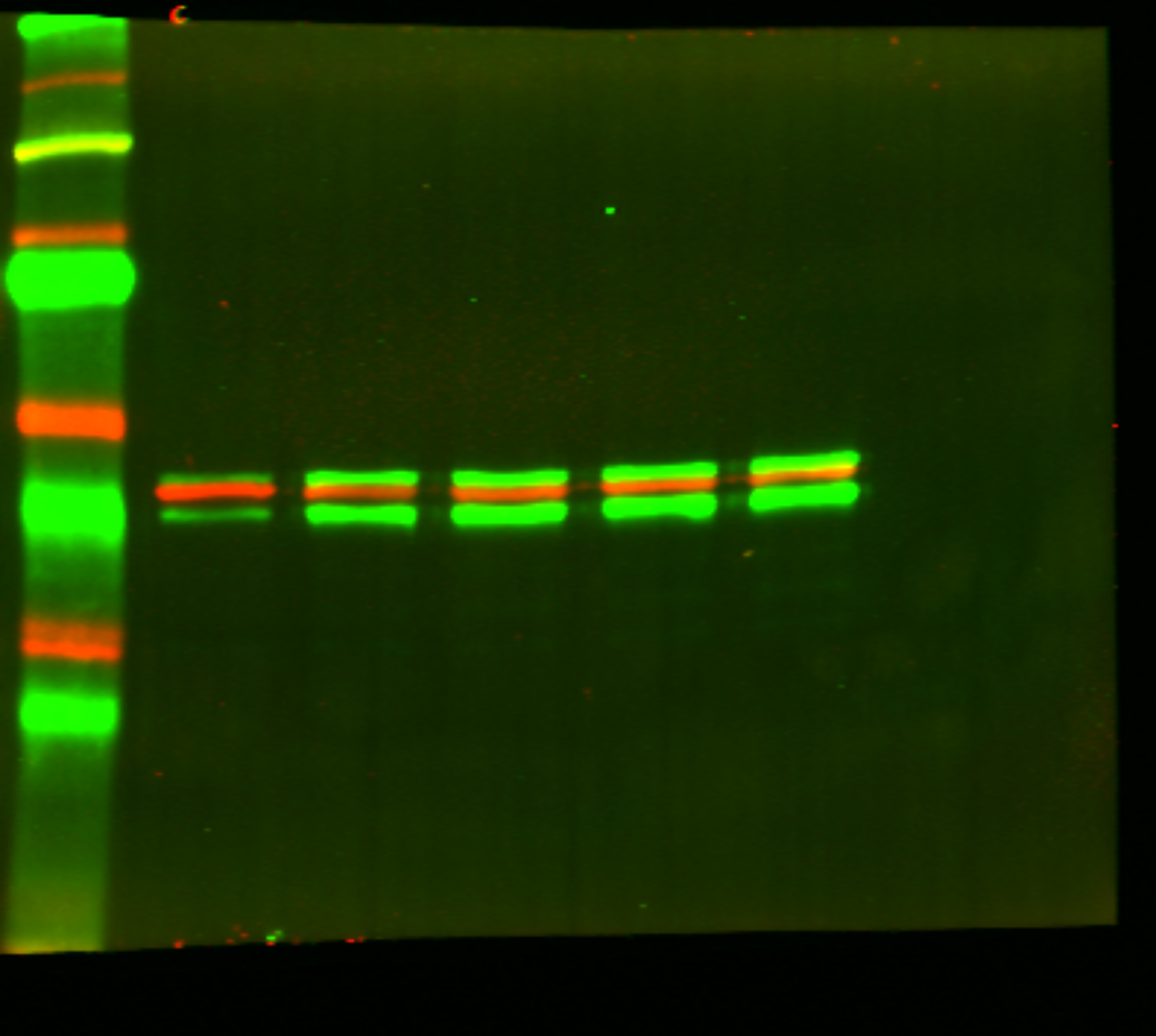

Supplement: Figure 3—source data 1. [file elife-82184-fig3-data1.zip › Figure 3-source data 1/KRasG13C/1_KRasG13C_pErk.tif]

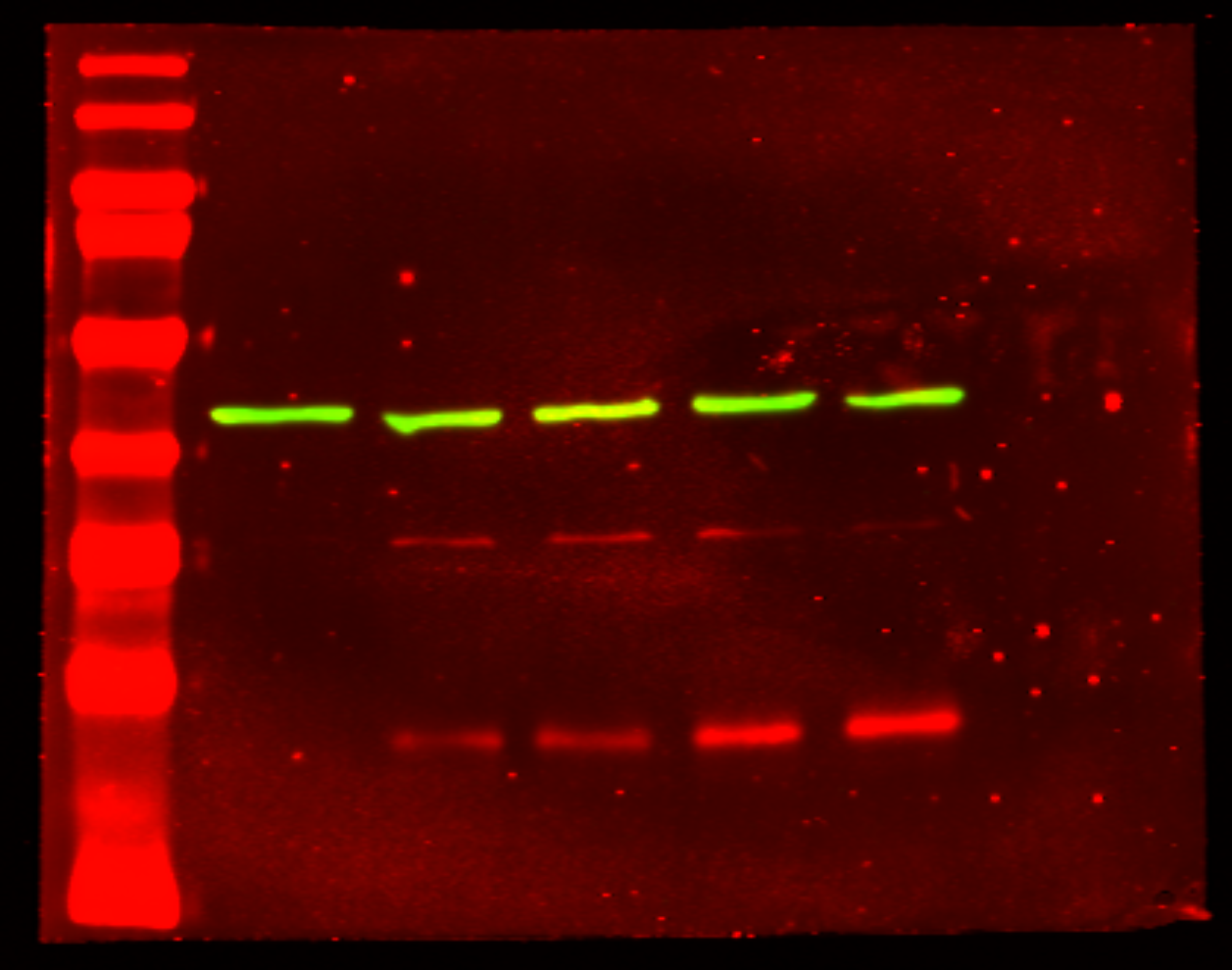

Supplement: Figure 3—source data 1. [file elife-82184-fig3-data1.zip › Figure 3-source data 1/KRasWT/1_KRasWT_KRas.tif]

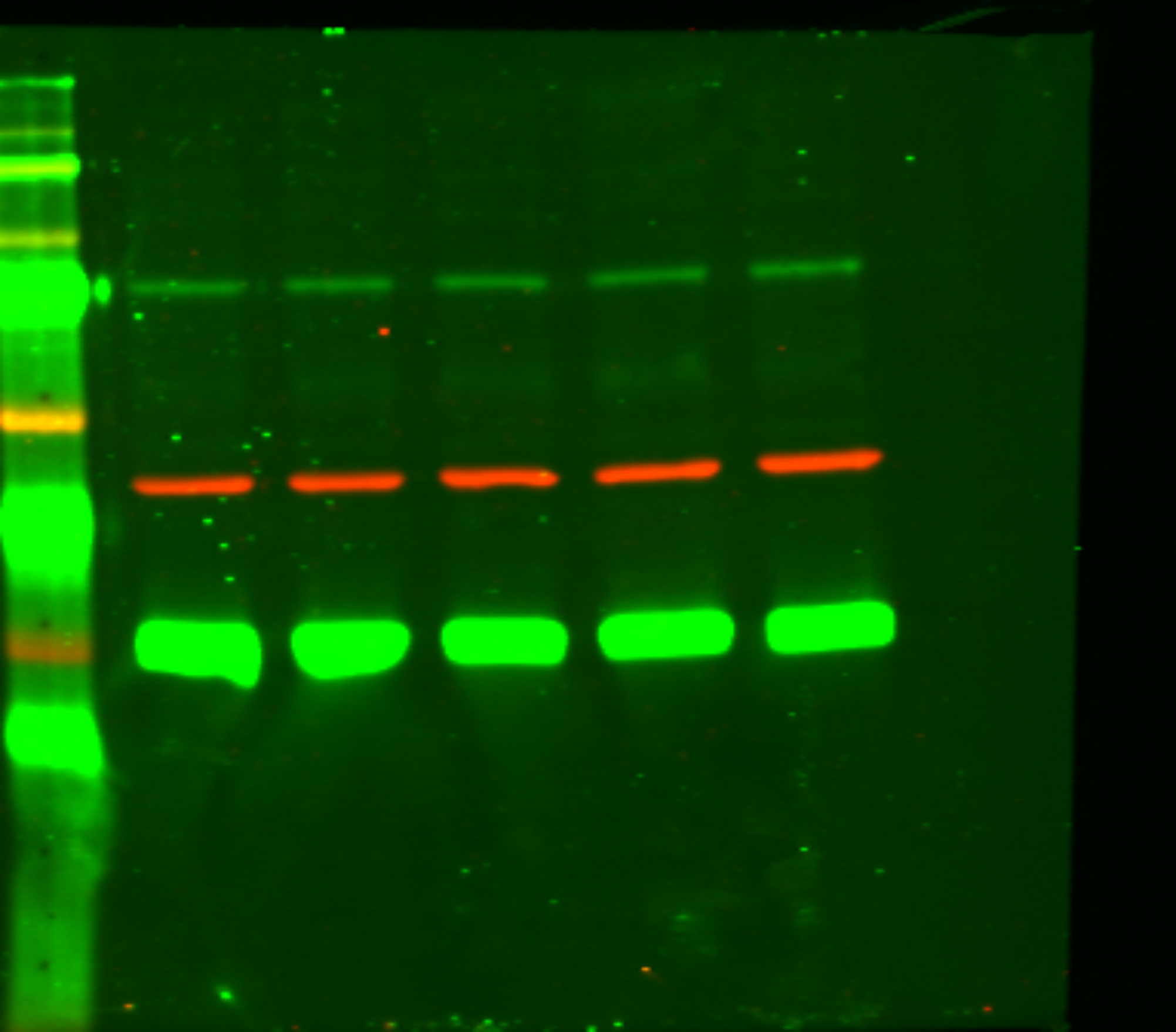

Supplement: Figure 3—source data 1. [file elife-82184-fig3-data1.zip › Figure 3-source data 1/KRasWT/1_KRasWT_pcRaf_pS6.tif]

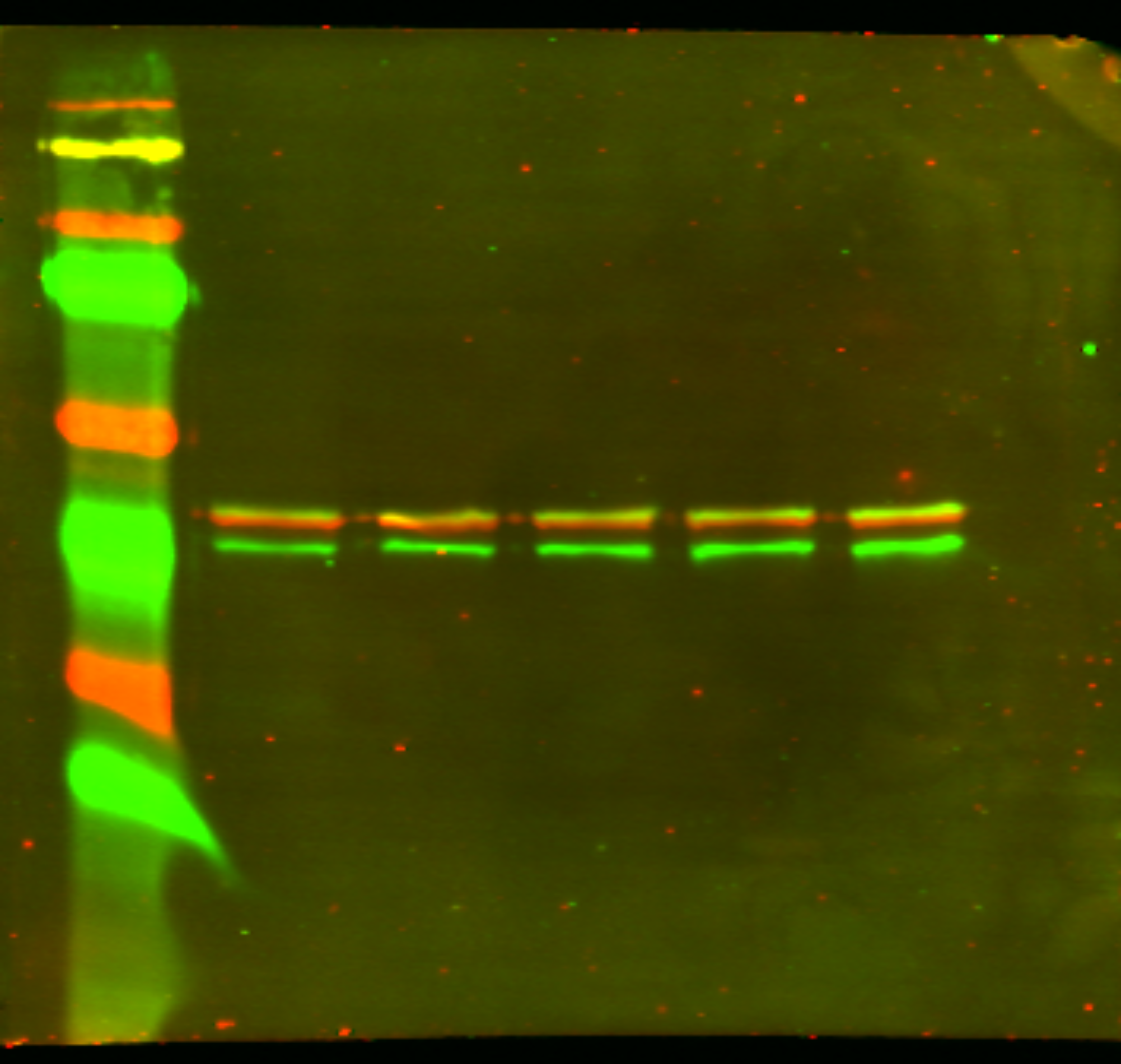

Supplement: Figure 3—source data 1. [file elife-82184-fig3-data1.zip › Figure 3-source data 1/KRasWT/1_KRasWT_pErK.tif]

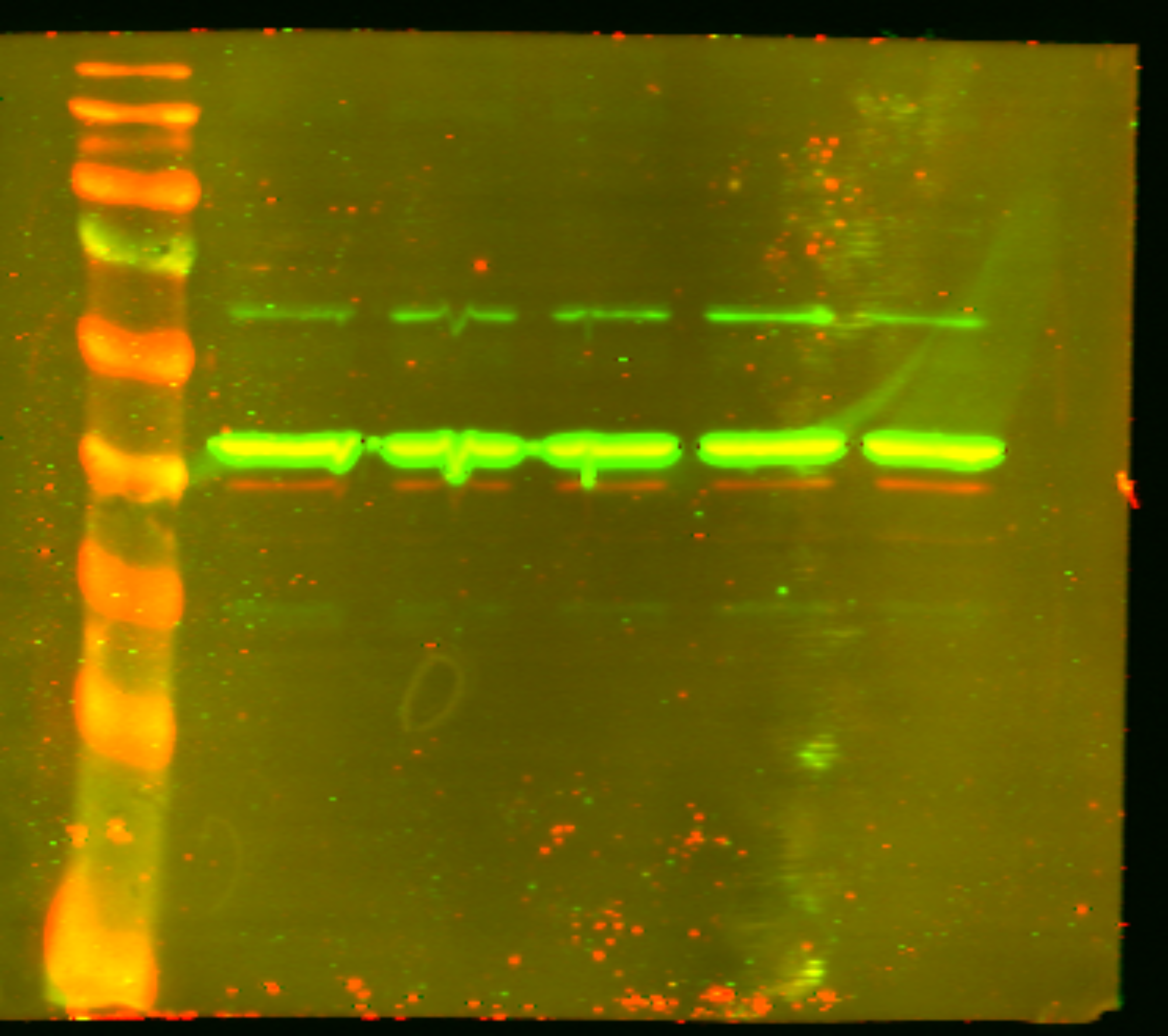

Supplement: Figure 3—source data 1. [file elife-82184-fig3-data1.zip › Figure 3-source data 1/KRasWT/1_KRasWT_tErk_pAkt.tif]

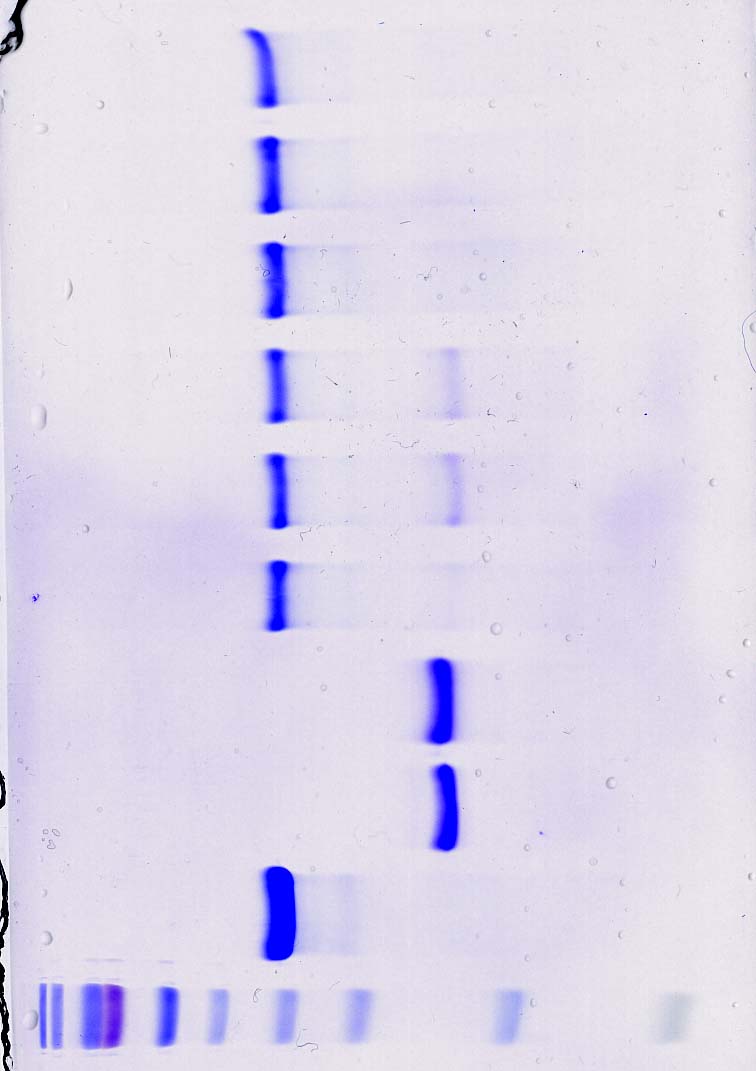

Supplement: Figure 3—figure supplement 3—source data 1. [file elife-82184-fig3-figsupp3-data1.zip › Figure 3-figure supplement 3-source data/Figure 3-figure supplement 3-source data.jpg]

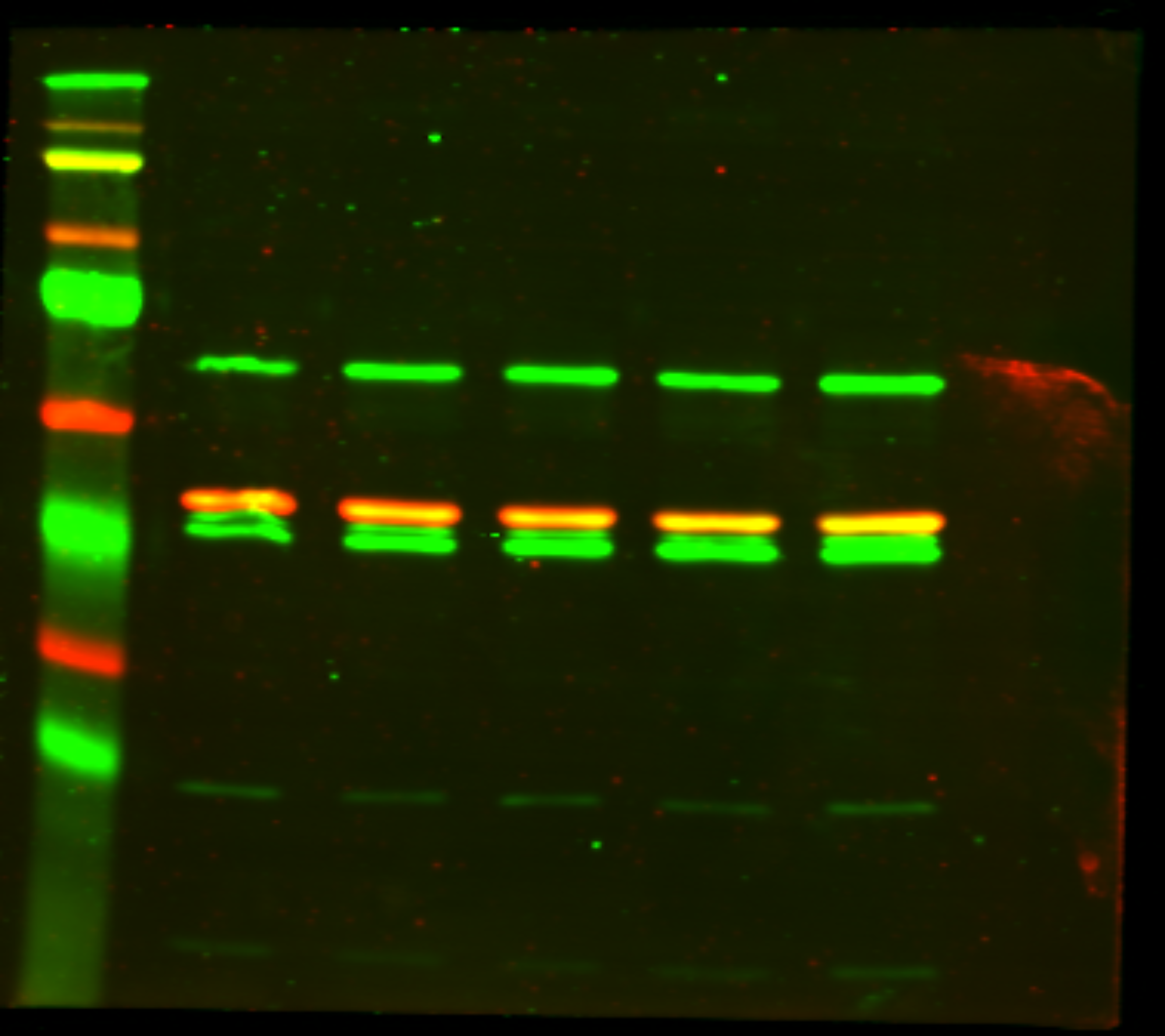

Supplement: Figure 3—figure supplement 6—source data 1. [file elife-82184-fig3-figsupp6-data1.zip › Figure 3-figure supplement 6-source data/A_KRasWT/1/1_KRasWT_tAkt.tif]

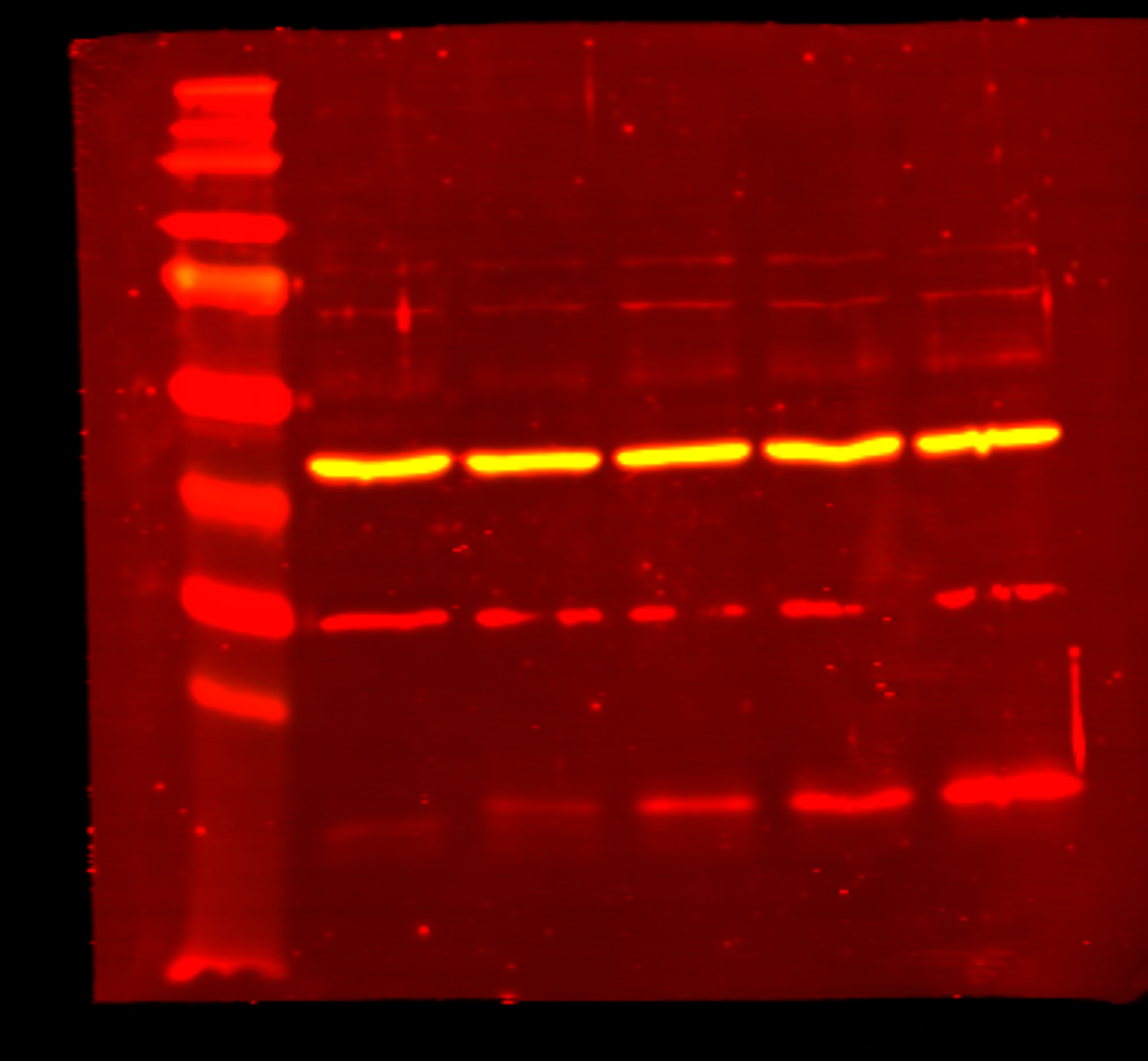

Supplement: Figure 3—figure supplement 6—source data 1. [file elife-82184-fig3-figsupp6-data1.zip › Figure 3-figure supplement 6-source data/A_KRasWT/2/2_KRasWT_KRas.tif]

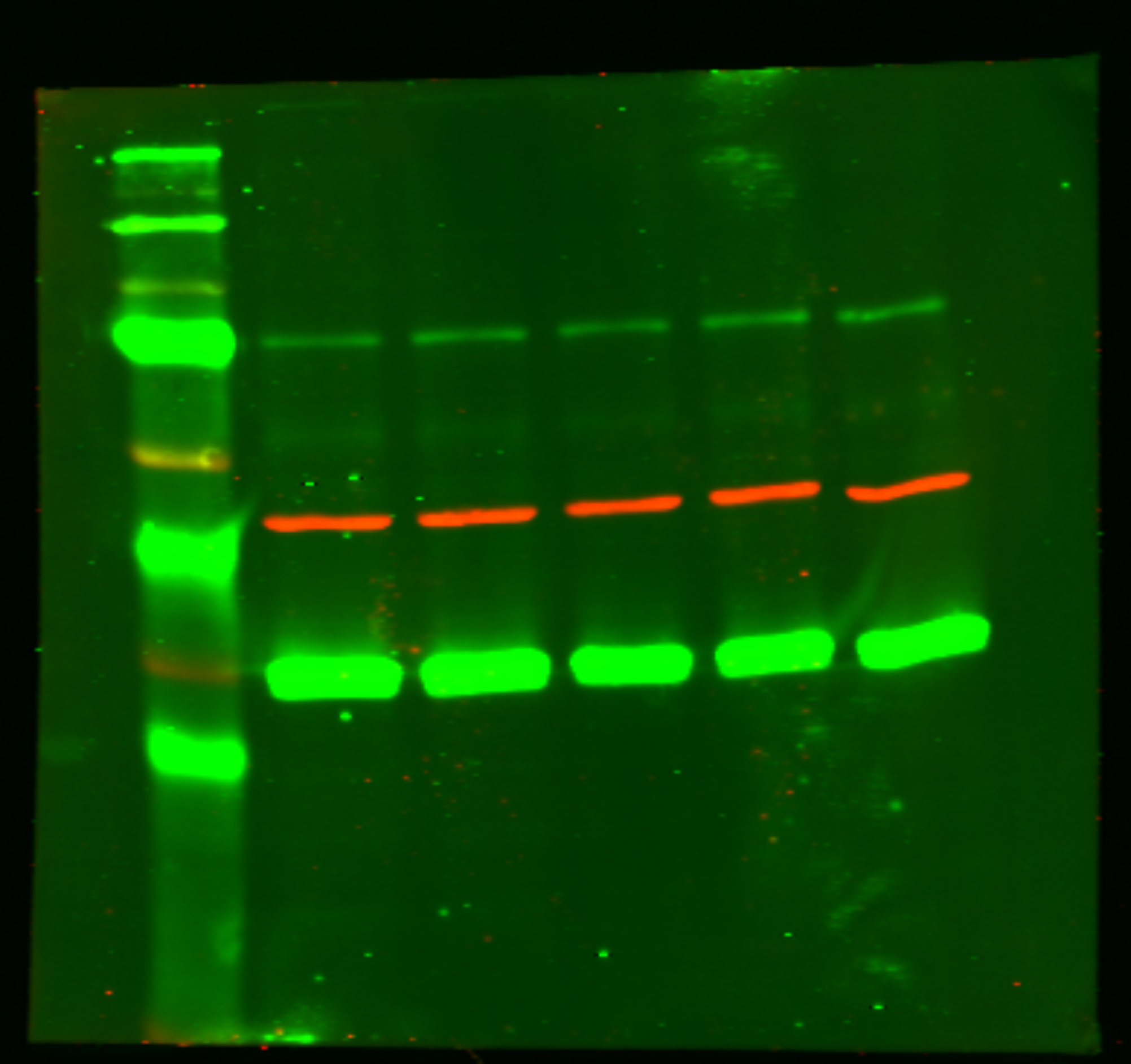

Supplement: Figure 3—figure supplement 6—source data 1. [file elife-82184-fig3-figsupp6-data1.zip › Figure 3-figure supplement 6-source data/A_KRasWT/2/2_KRasWT_pcRaf_pS6.tif]

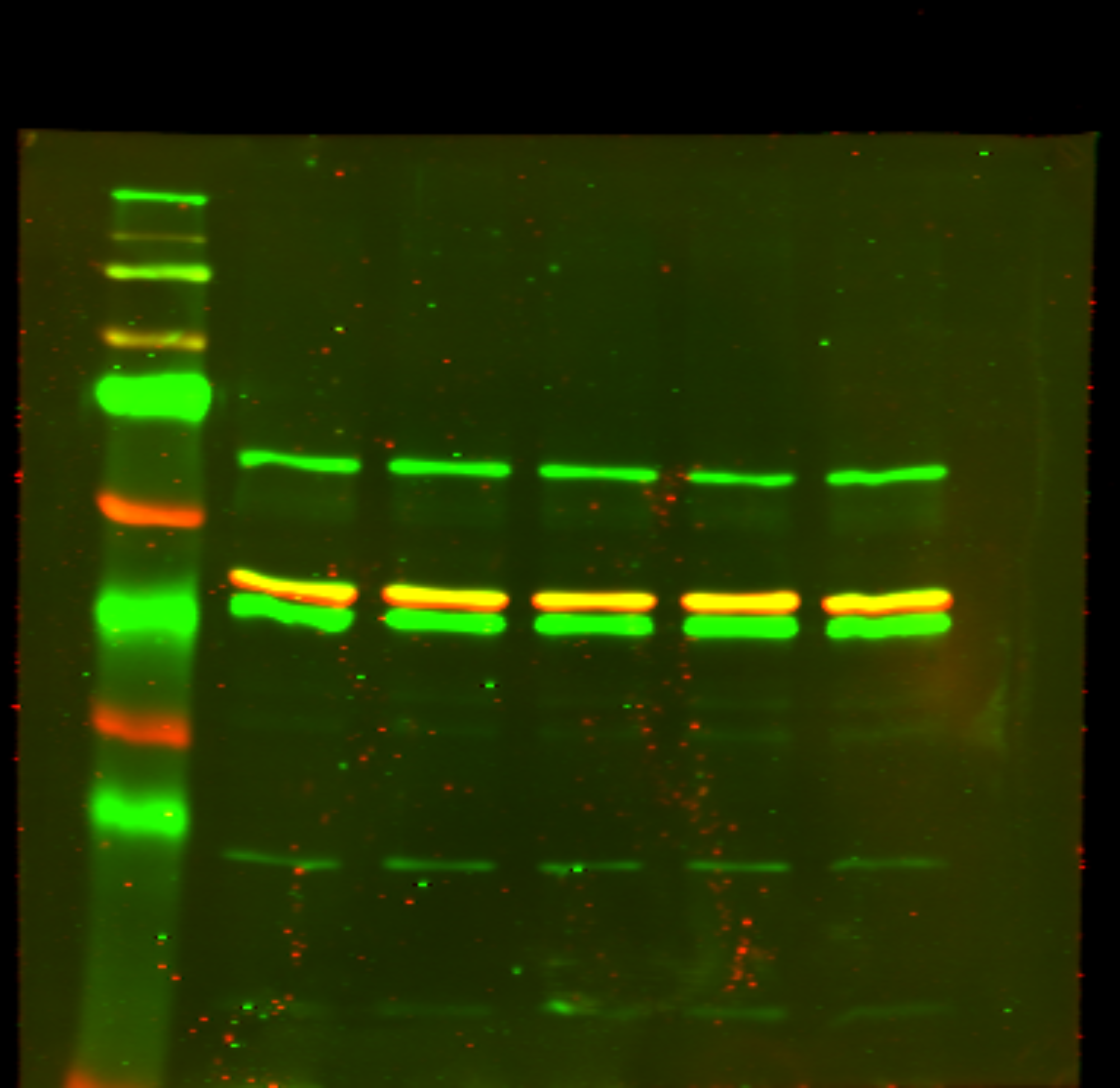

Supplement: Figure 3—figure supplement 6—source data 1. [file elife-82184-fig3-figsupp6-data1.zip › Figure 3-figure supplement 6-source data/A_KRasWT/2/2_KRasWT_pErk_tAkt.tif]

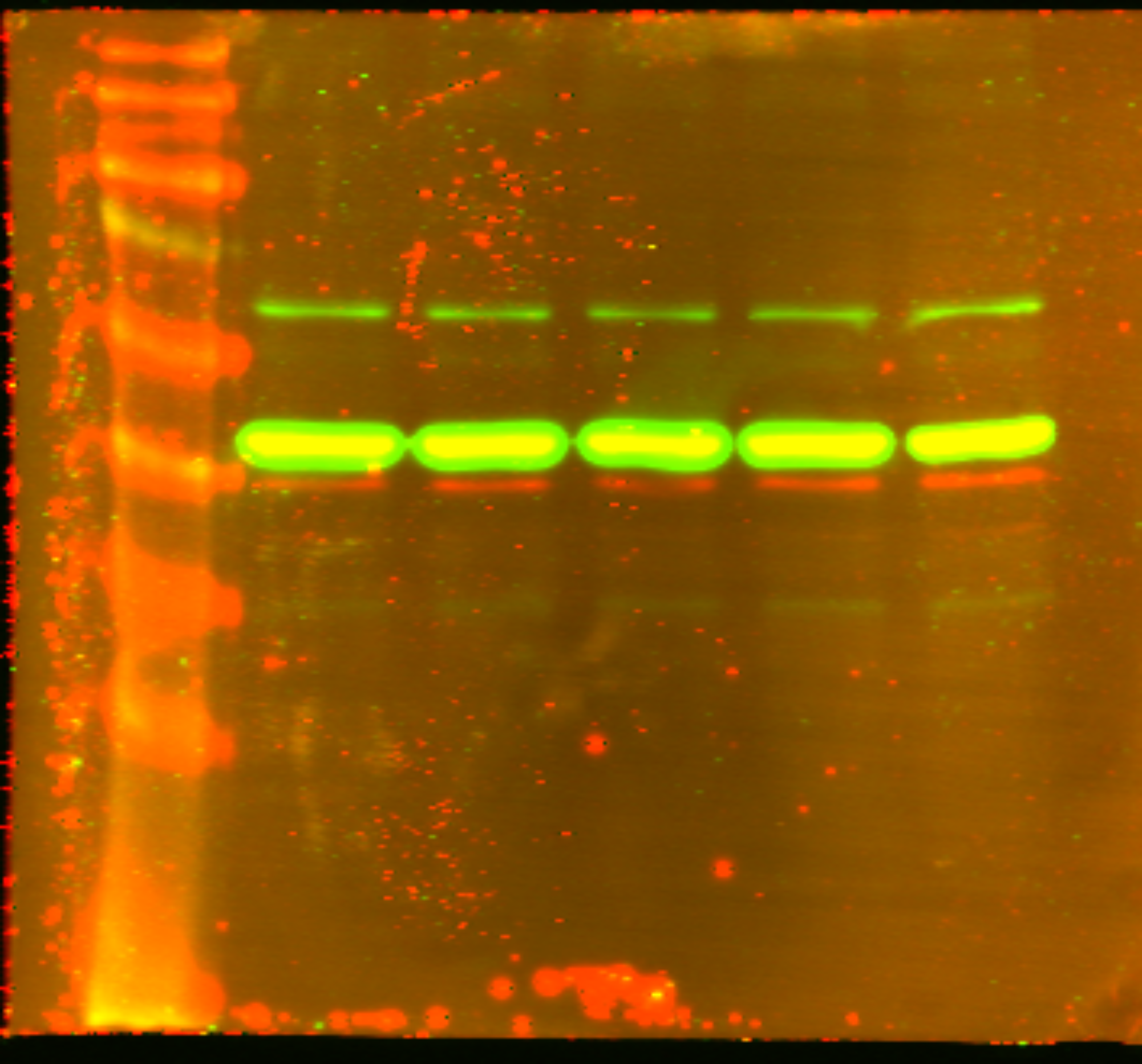

Supplement: Figure 3—figure supplement 6—source data 1. [file elife-82184-fig3-figsupp6-data1.zip › Figure 3-figure supplement 6-source data/A_KRasWT/2/2_KRasWT_tErk_pAkt.tif]

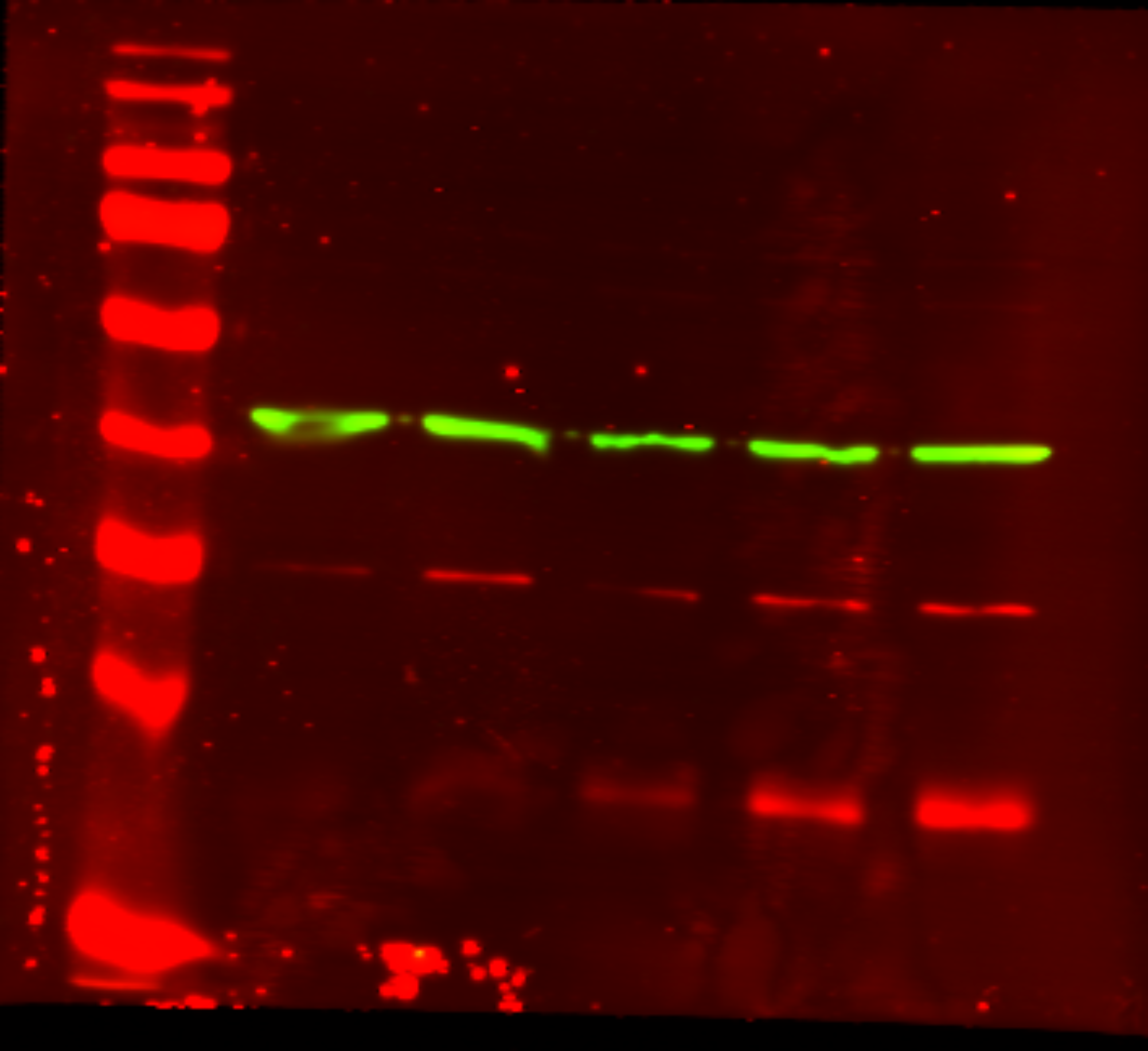

Supplement: Figure 3—figure supplement 6—source data 1. [file elife-82184-fig3-figsupp6-data1.zip › Figure 3-figure supplement 6-source data/A_KRasWT/3/3_KRasWT_KRas.tif]

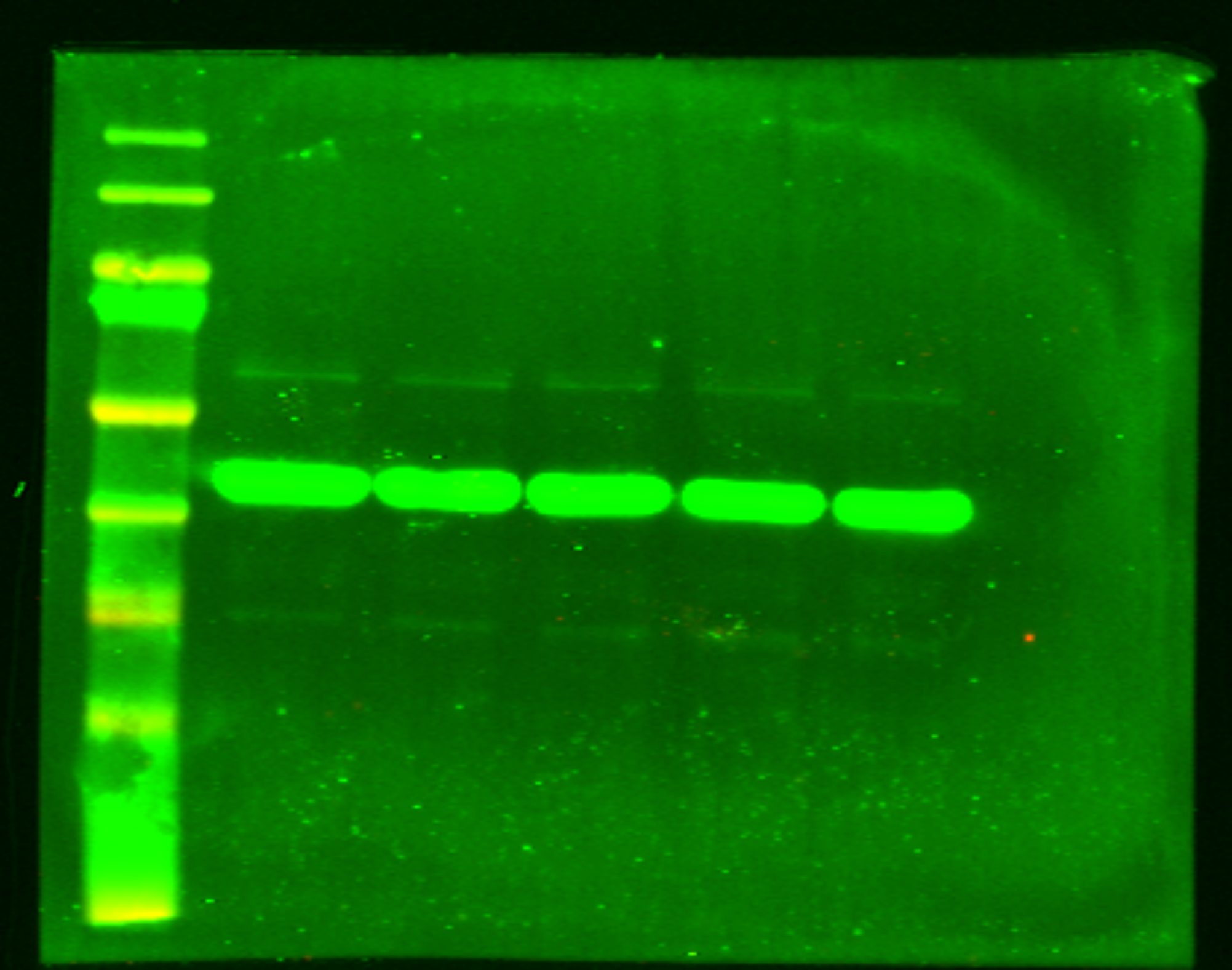

Supplement: Figure 3—figure supplement 6—source data 1. [file elife-82184-fig3-figsupp6-data1.zip › Figure 3-figure supplement 6-source data/A_KRasWT/3/3_KRasWT_pAkt.tif]

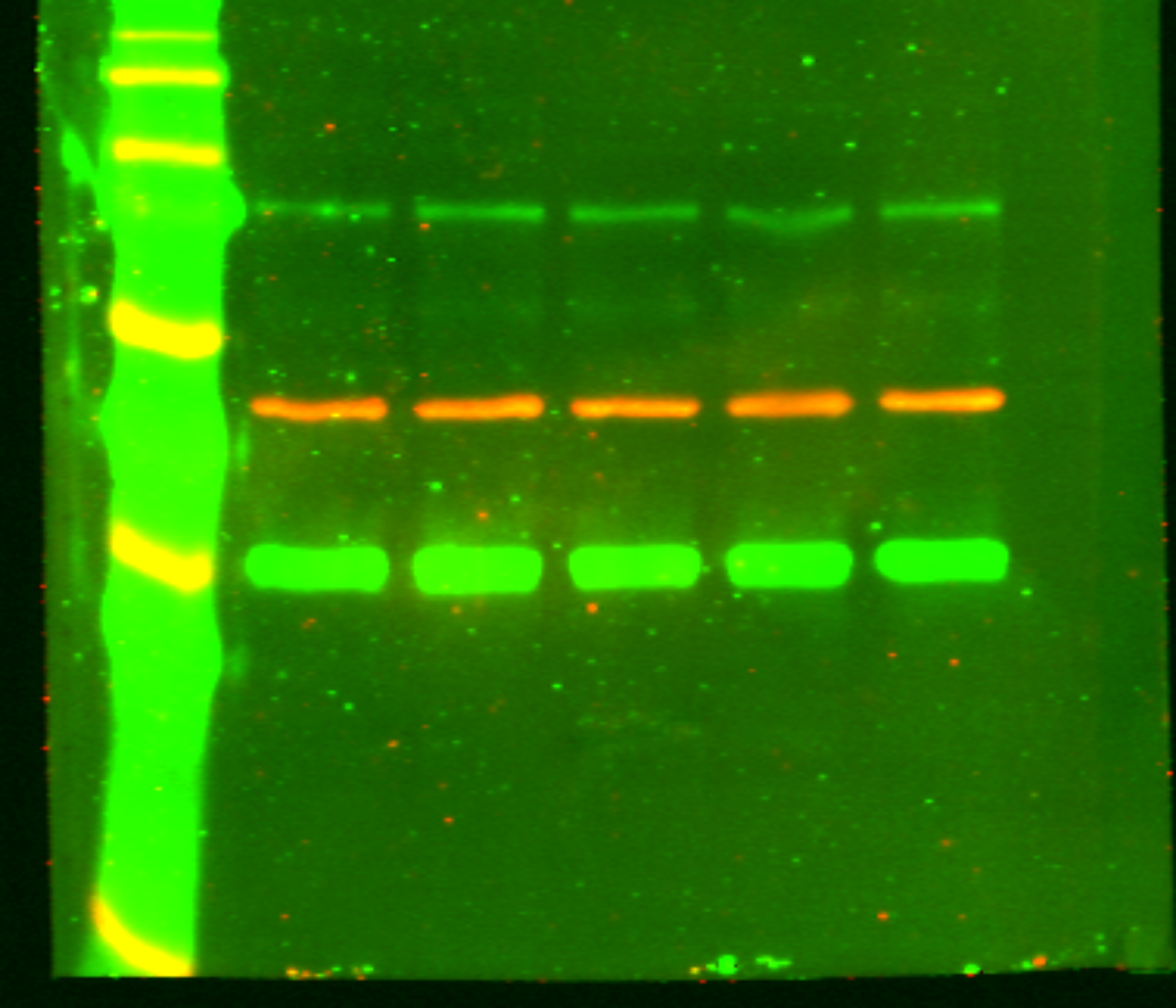

Supplement: Figure 3—figure supplement 6—source data 1. [file elife-82184-fig3-figsupp6-data1.zip › Figure 3-figure supplement 6-source data/A_KRasWT/3/3_KRasWT_pcRaf_pS6.tif]

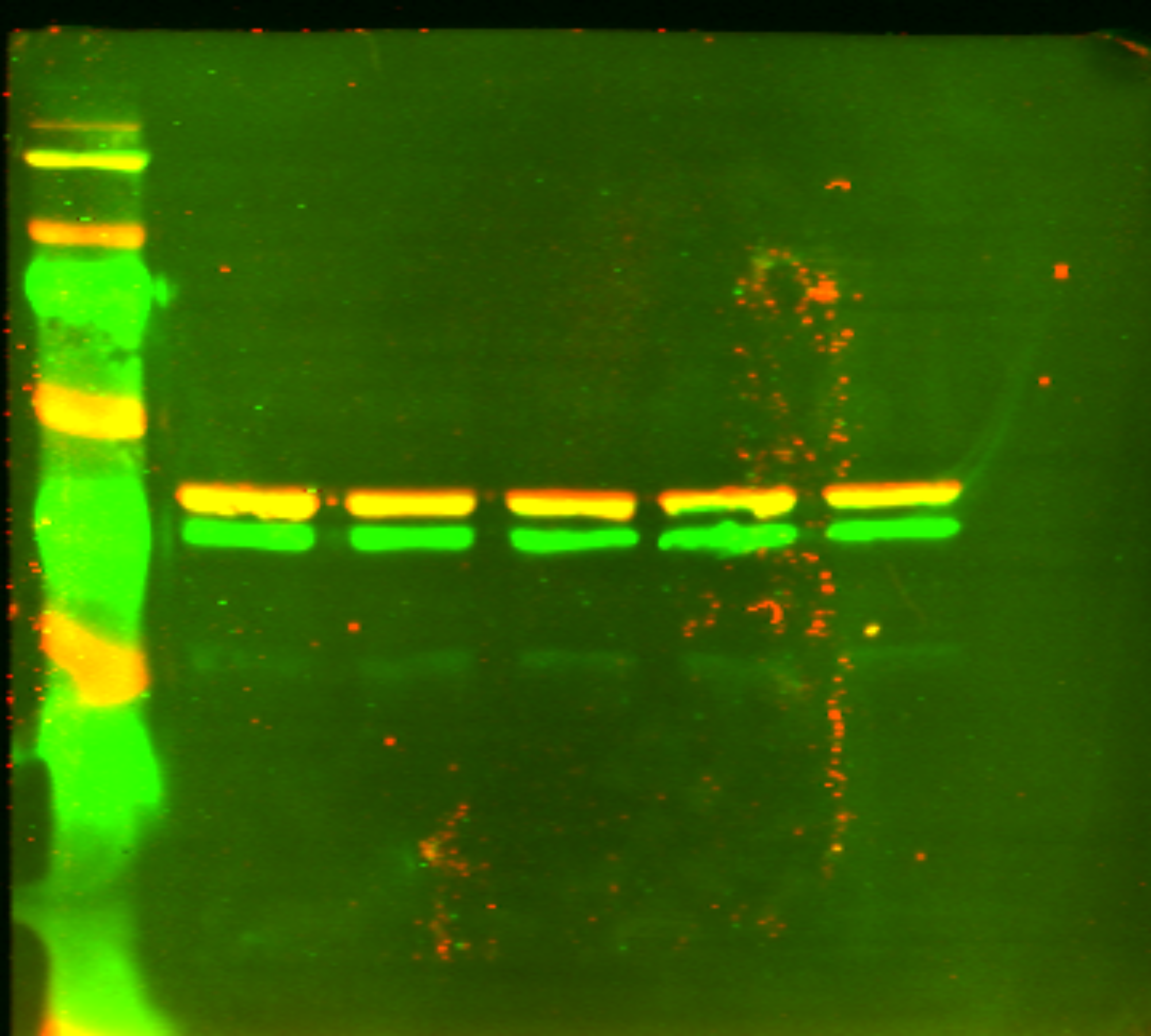

Supplement: Figure 3—figure supplement 6—source data 1. [file elife-82184-fig3-figsupp6-data1.zip › Figure 3-figure supplement 6-source data/A_KRasWT/3/3_KRasWT_pErk.tif]

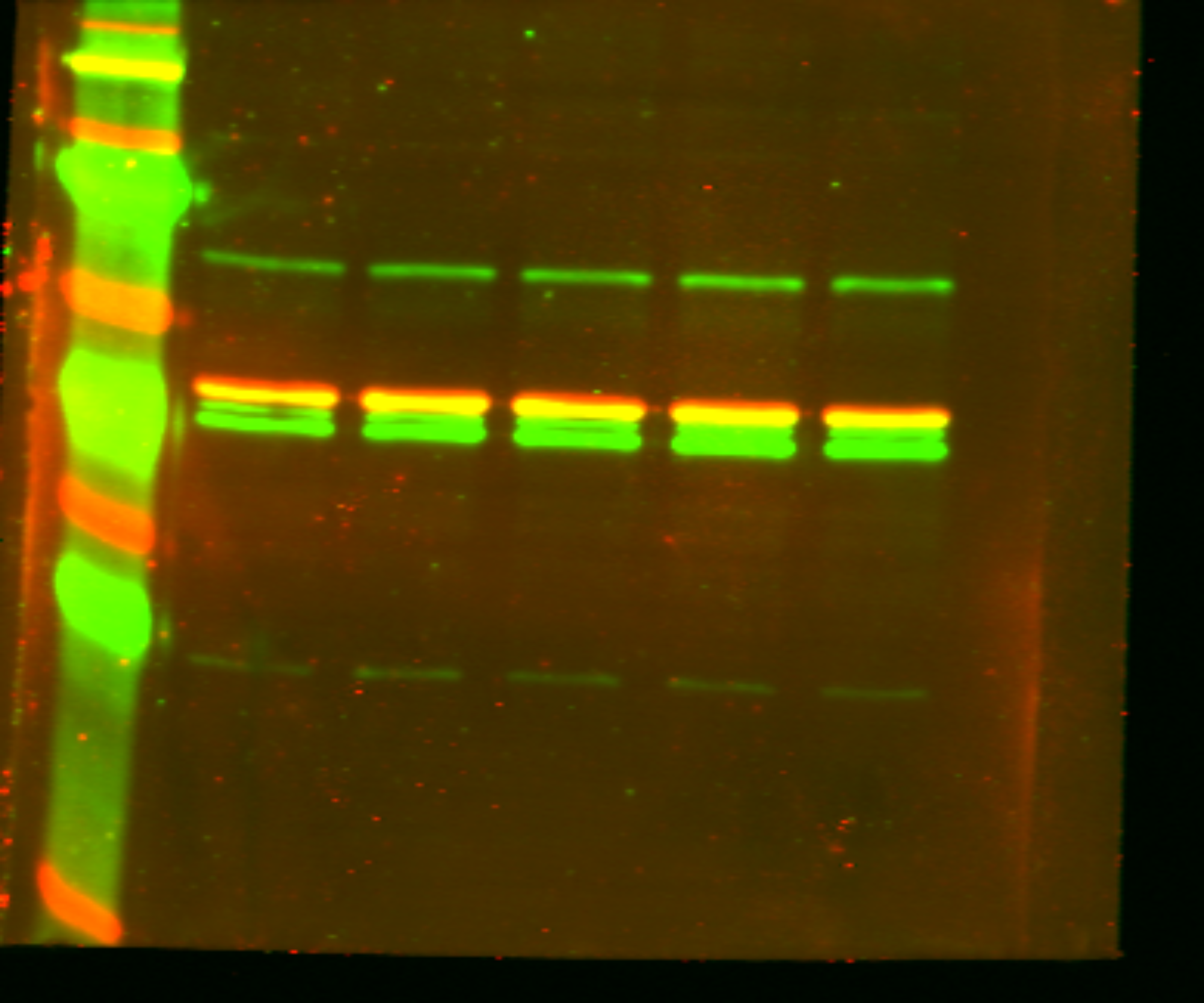

Supplement: Figure 3—figure supplement 6—source data 1. [file elife-82184-fig3-figsupp6-data1.zip › Figure 3-figure supplement 6-source data/A_KRasWT/3/3_KRasWT_tAkt.tif]

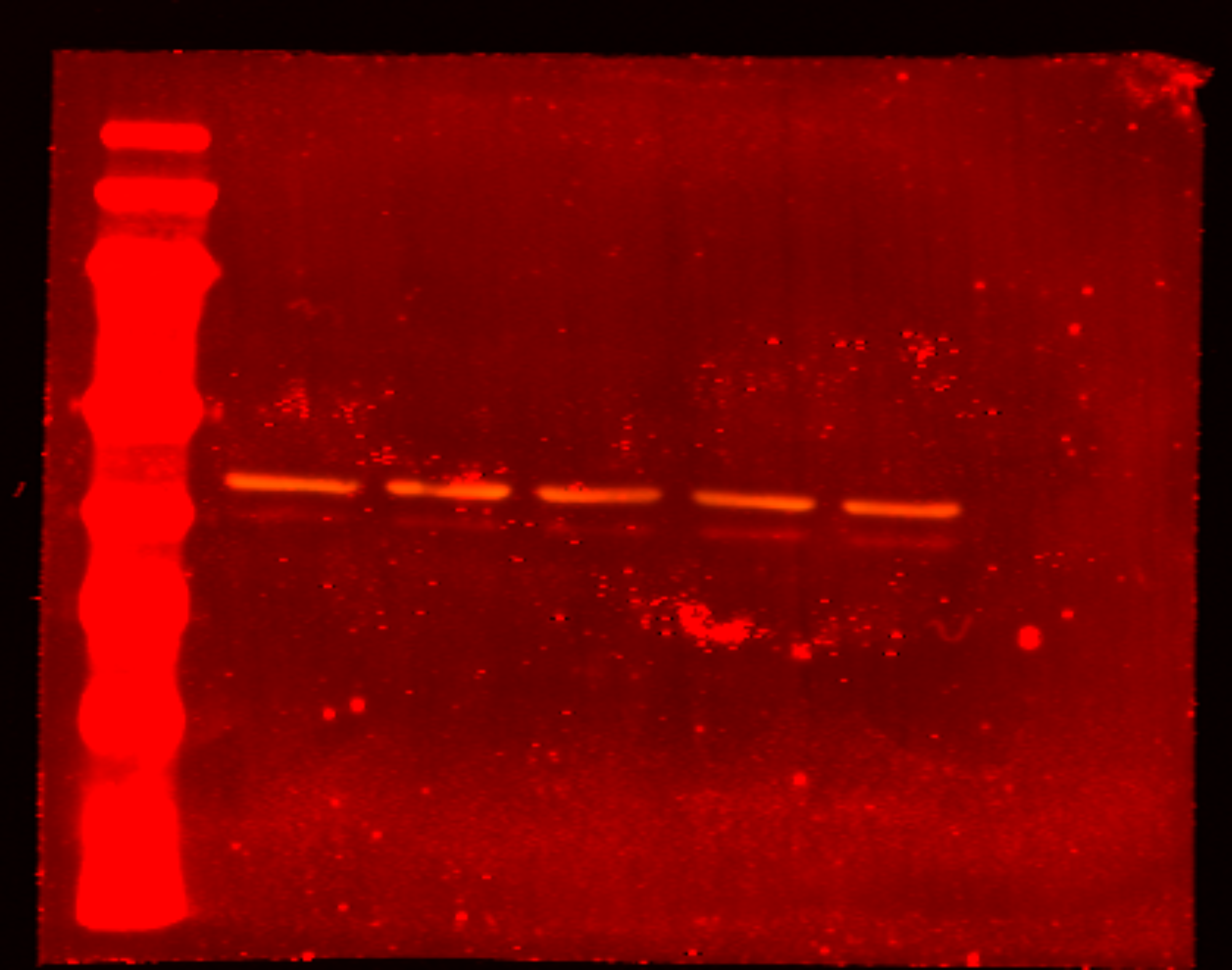

Supplement: Figure 3—figure supplement 6—source data 1. [file elife-82184-fig3-figsupp6-data1.zip › Figure 3-figure supplement 6-source data/A_KRasWT/3/3_KRasWT_tErk.tif]

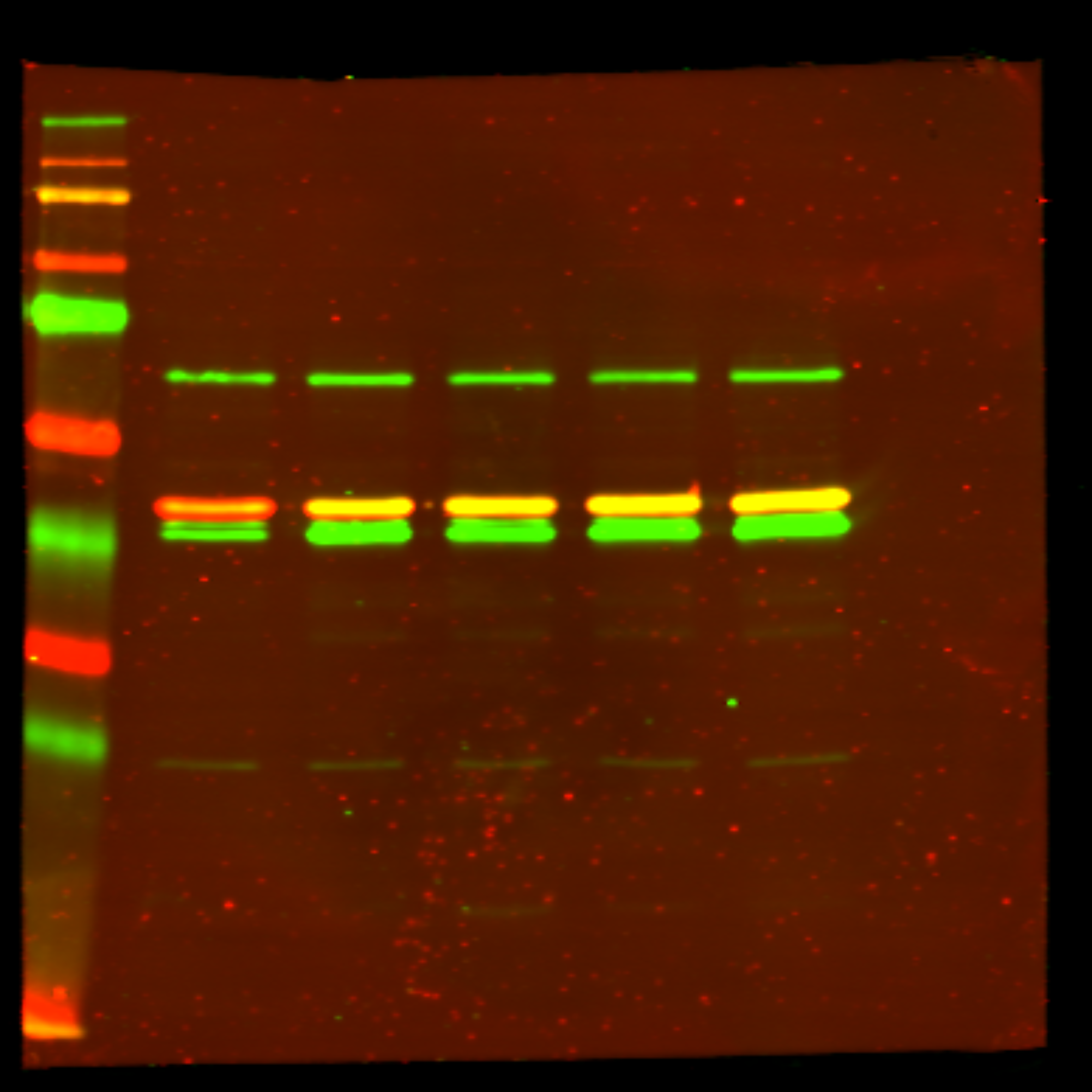

Supplement: Figure 3—figure supplement 6—source data 1. [file elife-82184-fig3-figsupp6-data1.zip › Figure 3-figure supplement 6-source data/B_KRasG13C/1/1_KRasG13C_tAkt.tif]

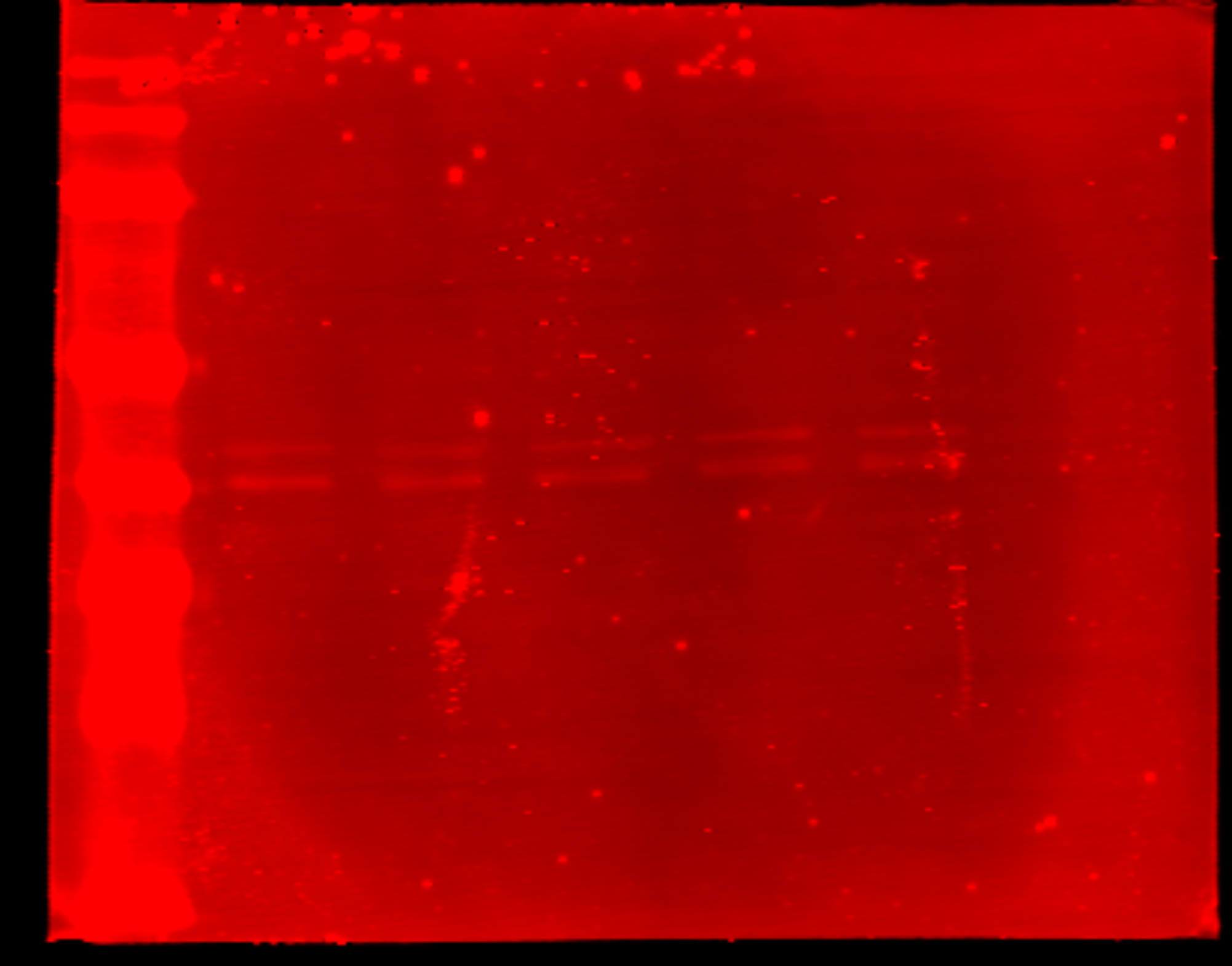

Supplement: Figure 3—figure supplement 6—source data 1. [file elife-82184-fig3-figsupp6-data1.zip › Figure 3-figure supplement 6-source data/B_KRasG13C/1/1_KRasG13C_tErk.tif]

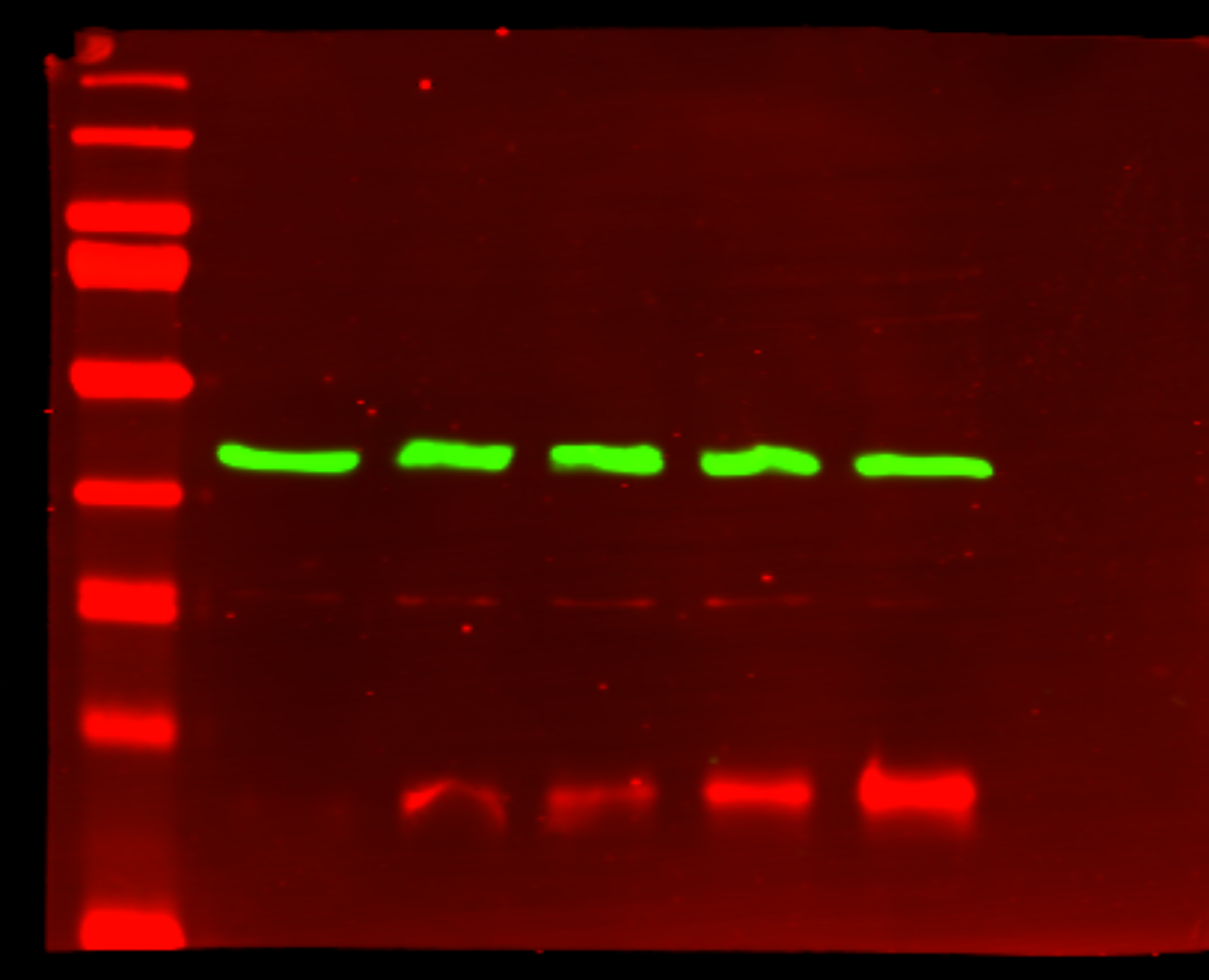

Supplement: Figure 3—figure supplement 6—source data 1. [file elife-82184-fig3-figsupp6-data1.zip › Figure 3-figure supplement 6-source data/B_KRasG13C/2/2_KRasG13C_KRas.tif]

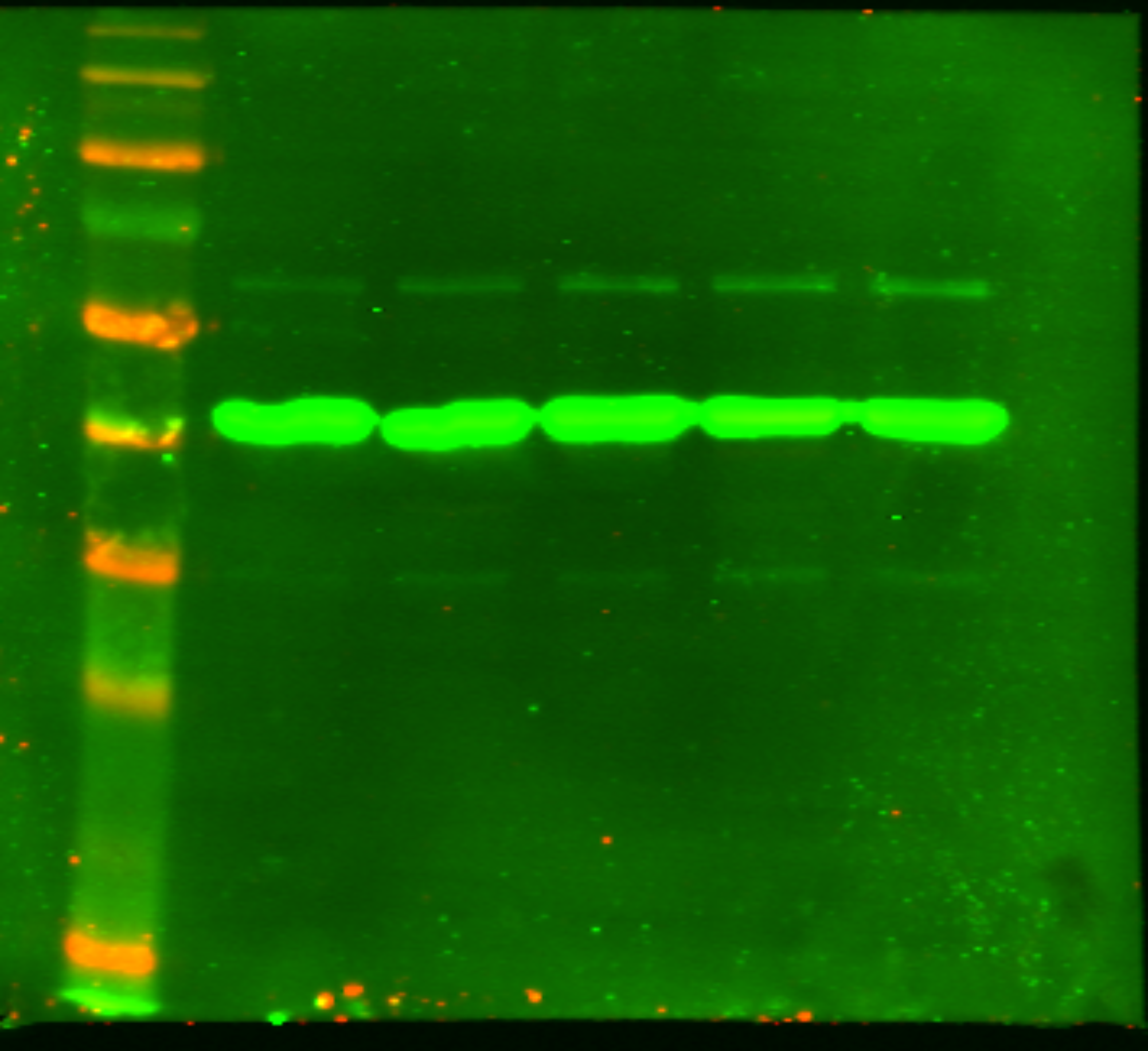

Supplement: Figure 3—figure supplement 6—source data 1. [file elife-82184-fig3-figsupp6-data1.zip › Figure 3-figure supplement 6-source data/B_KRasG13C/2/2_KRasG13C_pAkt.tif]

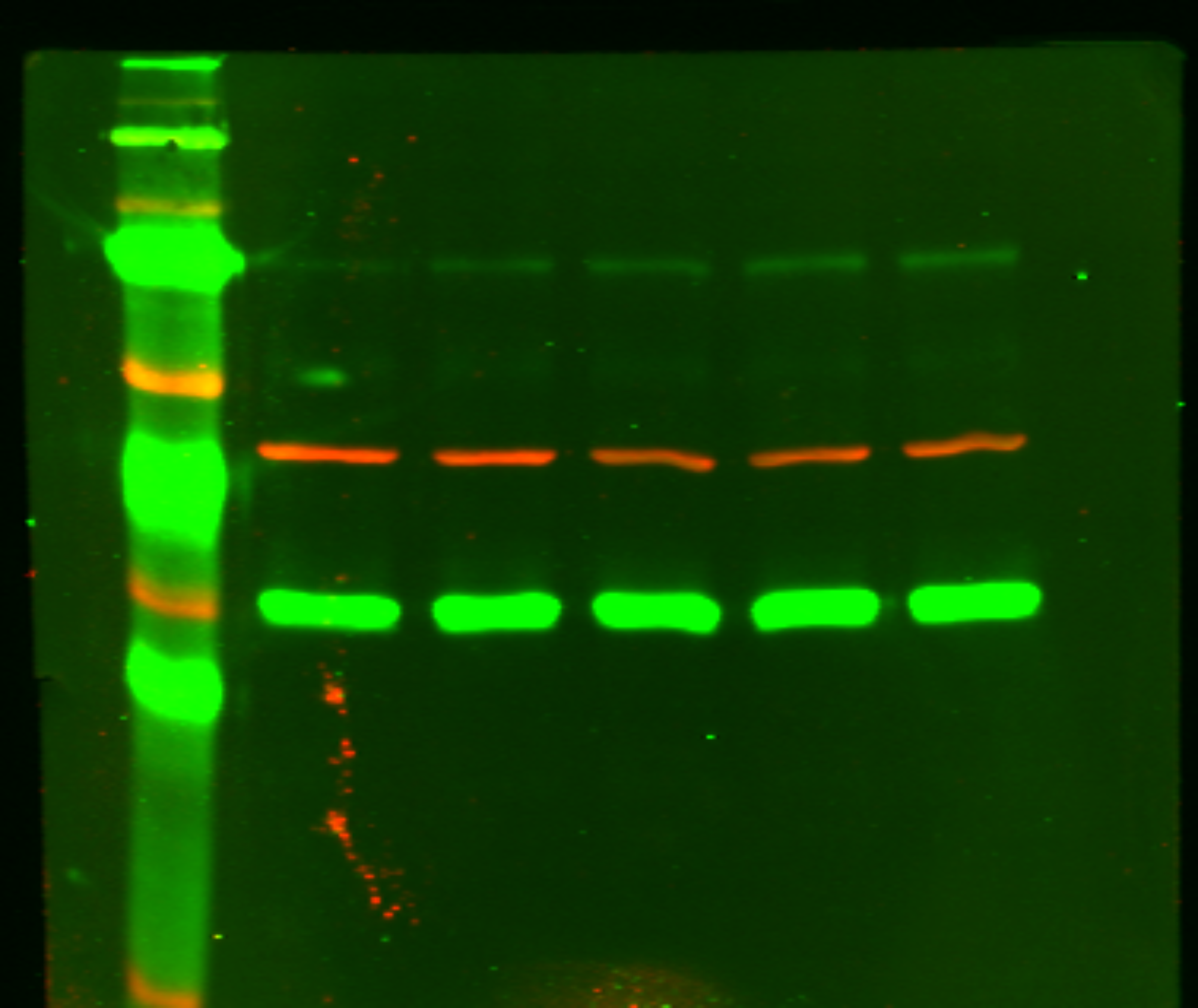

Supplement: Figure 3—figure supplement 6—source data 1. [file elife-82184-fig3-figsupp6-data1.zip › Figure 3-figure supplement 6-source data/B_KRasG13C/2/2_KRasG13C_pcRaf_pS6.tif]

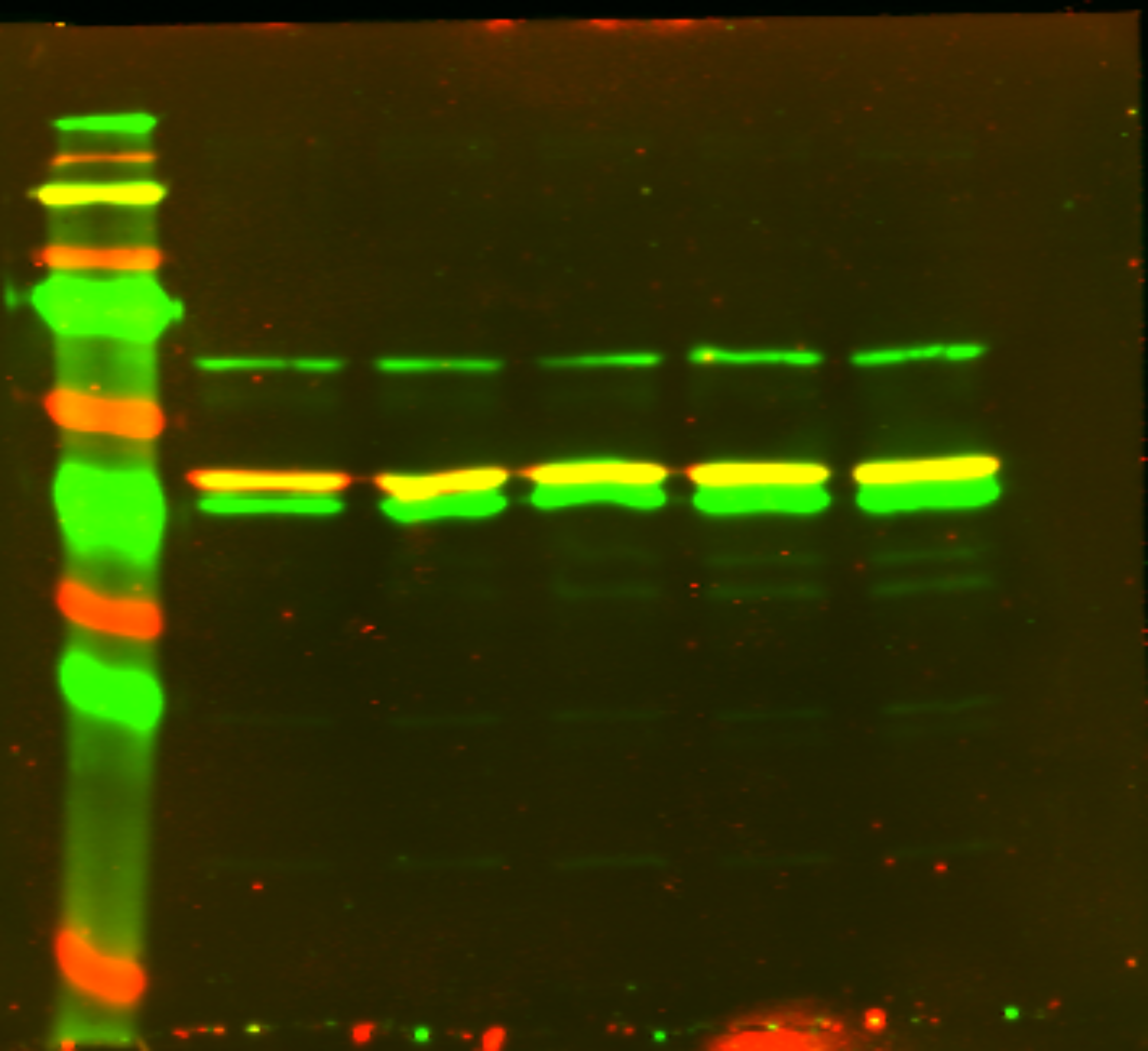

Supplement: Figure 3—figure supplement 6—source data 1. [file elife-82184-fig3-figsupp6-data1.zip › Figure 3-figure supplement 6-source data/B_KRasG13C/2/2_KRasG13C_tAkt_pErk.tif]

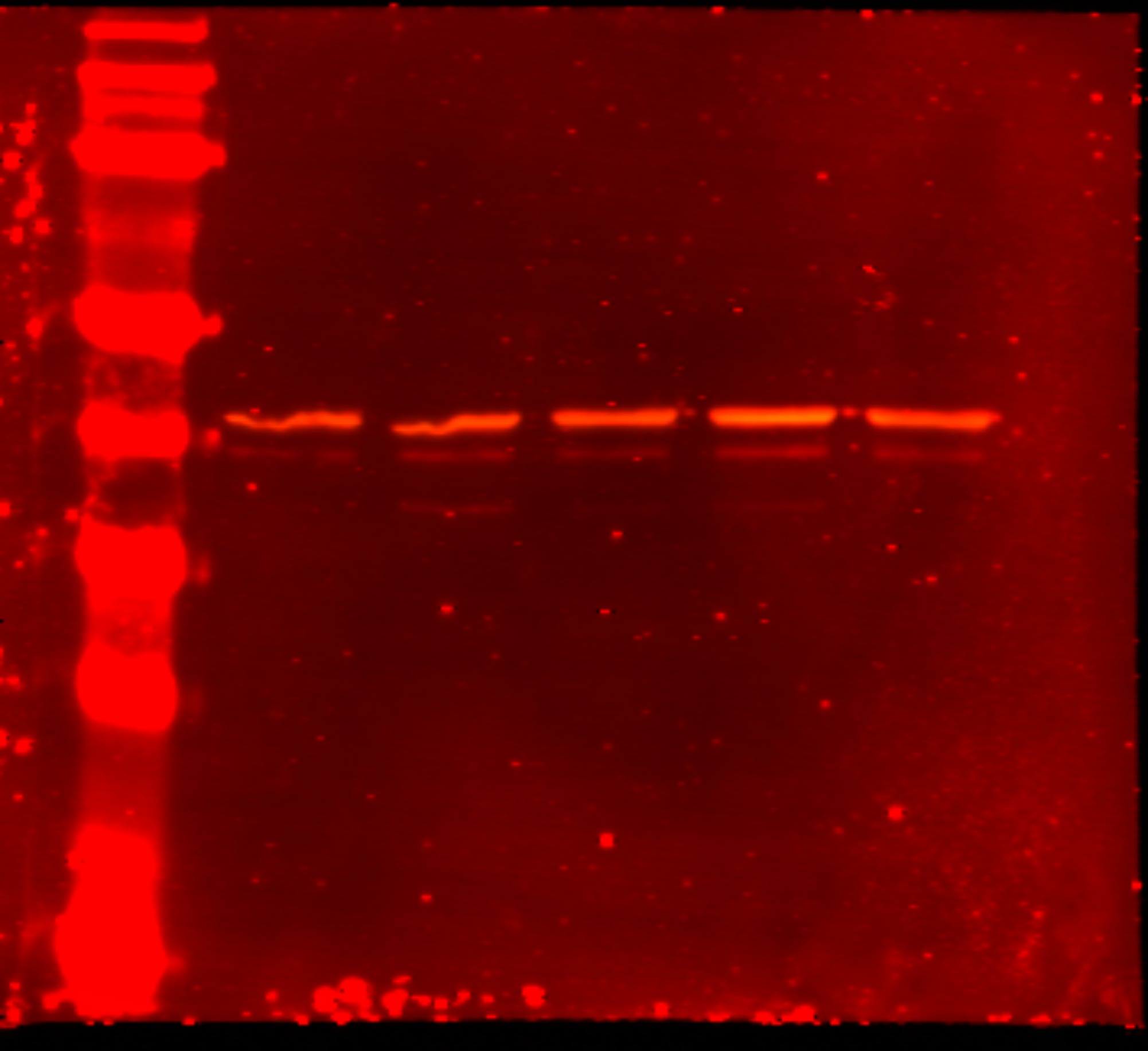

Supplement: Figure 3—figure supplement 6—source data 1. [file elife-82184-fig3-figsupp6-data1.zip › Figure 3-figure supplement 6-source data/B_KRasG13C/2/2_KRasG13C_tErk.tif]

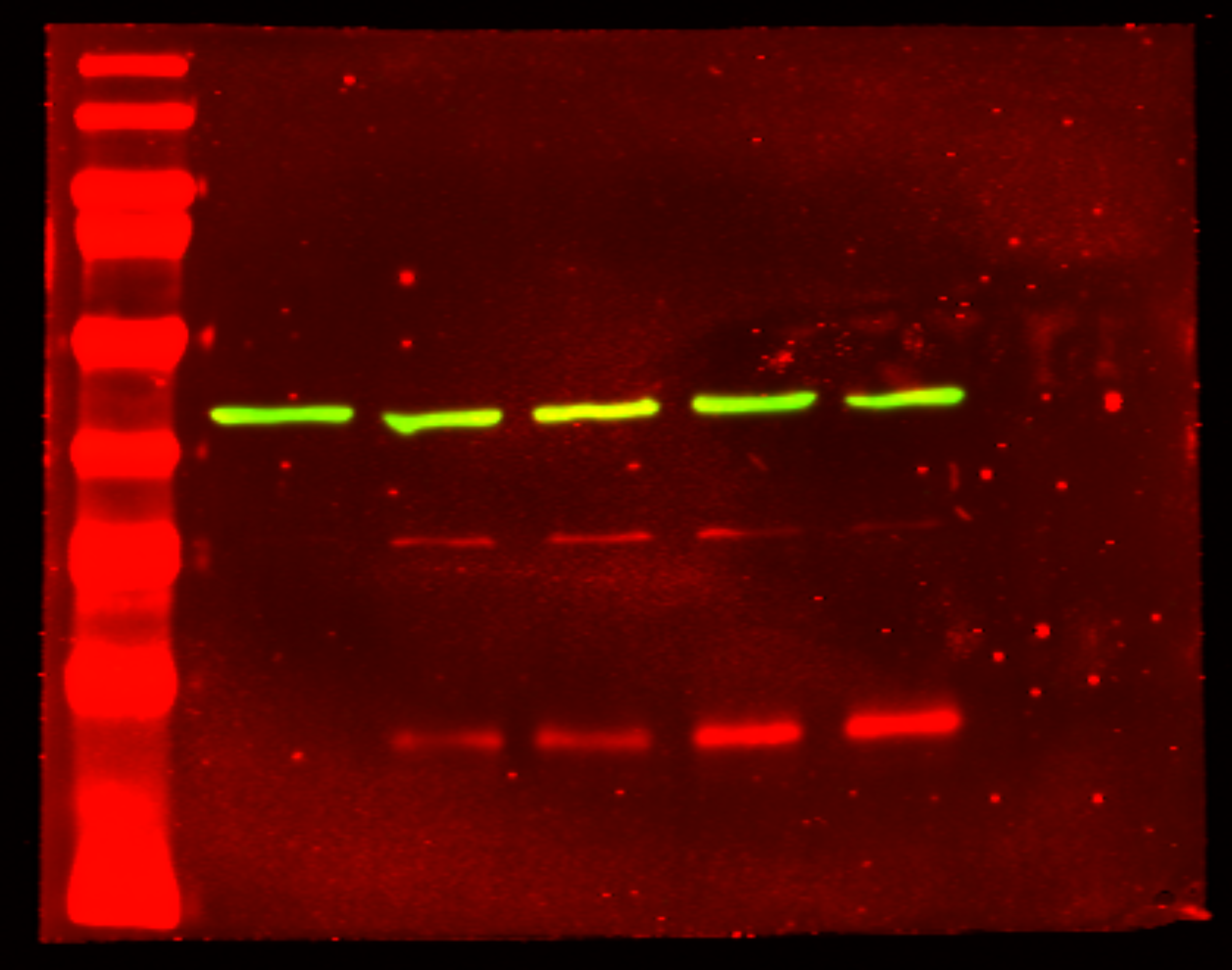

Supplement: Figure 3—figure supplement 6—source data 1. [file elife-82184-fig3-figsupp6-data1.zip › Figure 3-figure supplement 6-source data/B_KRasG13C/3/3_KRasG13C_KRas.tif]

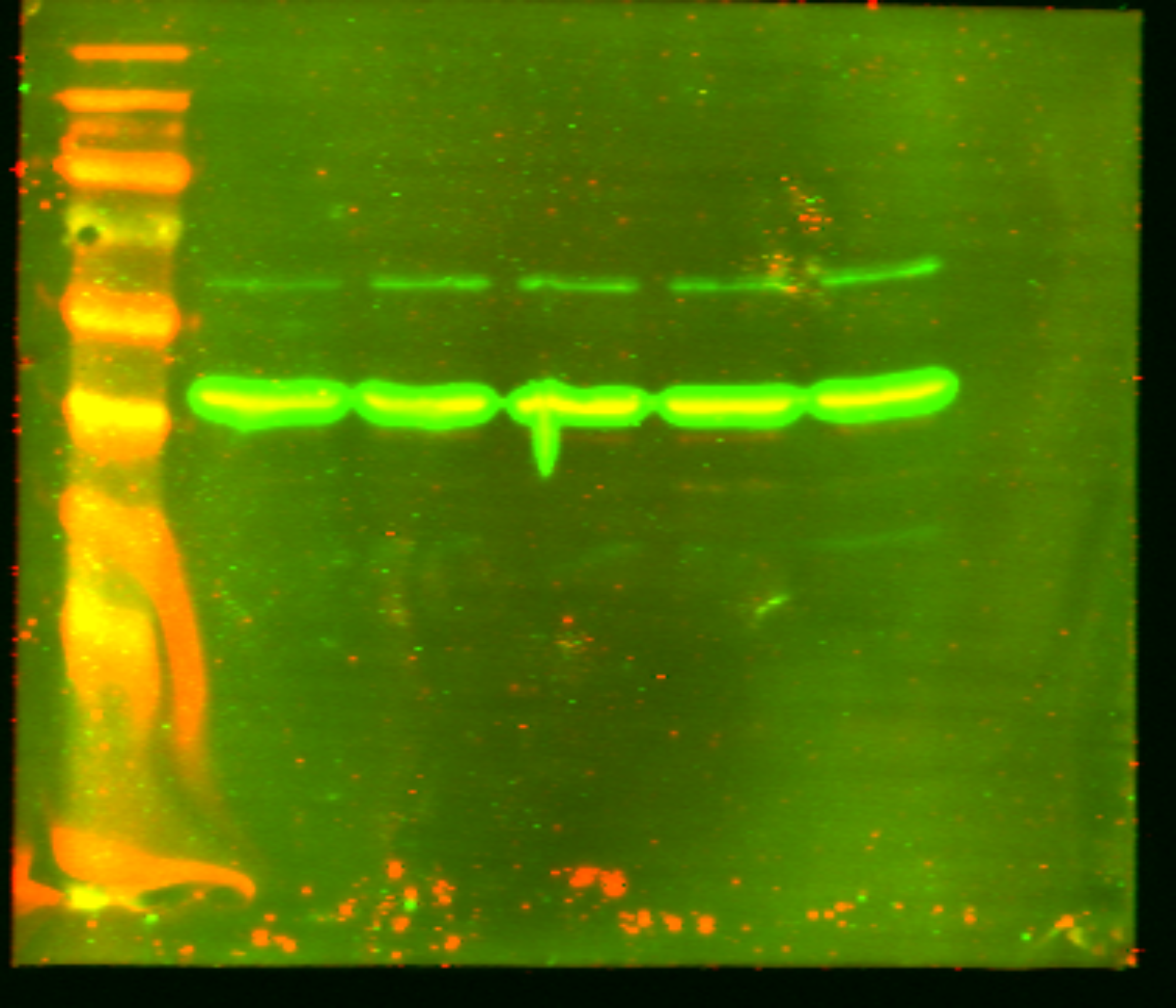

Supplement: Figure 3—figure supplement 6—source data 1. [file elife-82184-fig3-figsupp6-data1.zip › Figure 3-figure supplement 6-source data/B_KRasG13C/3/3_KRasG13C_pAkt.tif]

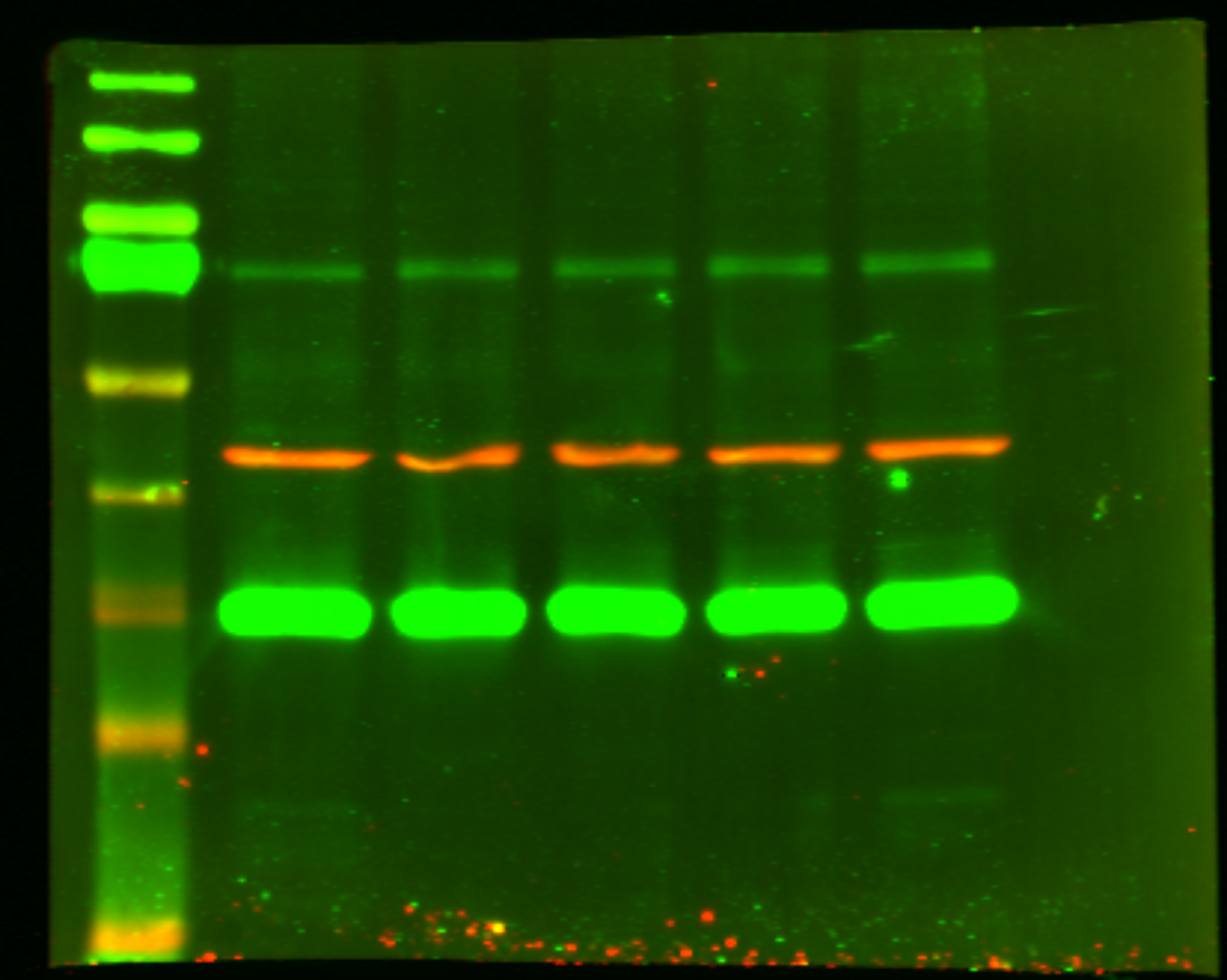

Supplement: Figure 3—figure supplement 6—source data 1. [file elife-82184-fig3-figsupp6-data1.zip › Figure 3-figure supplement 6-source data/B_KRasG13C/3/3_KRasG13C_pcRaf_pS6.tif]

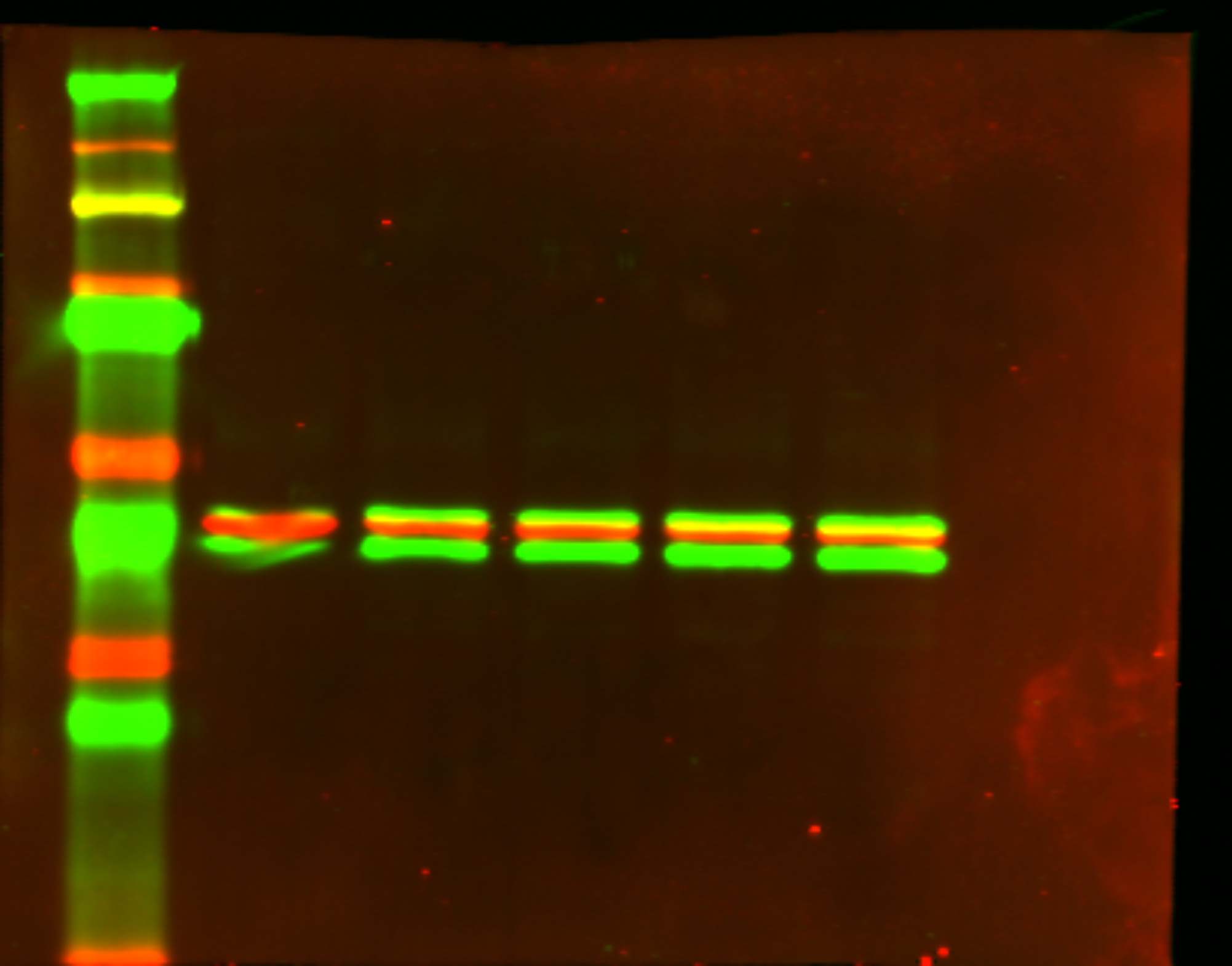

Supplement: Figure 3—figure supplement 6—source data 1. [file elife-82184-fig3-figsupp6-data1.zip › Figure 3-figure supplement 6-source data/B_KRasG13C/3/3_KRasG13C_pErk.tif]

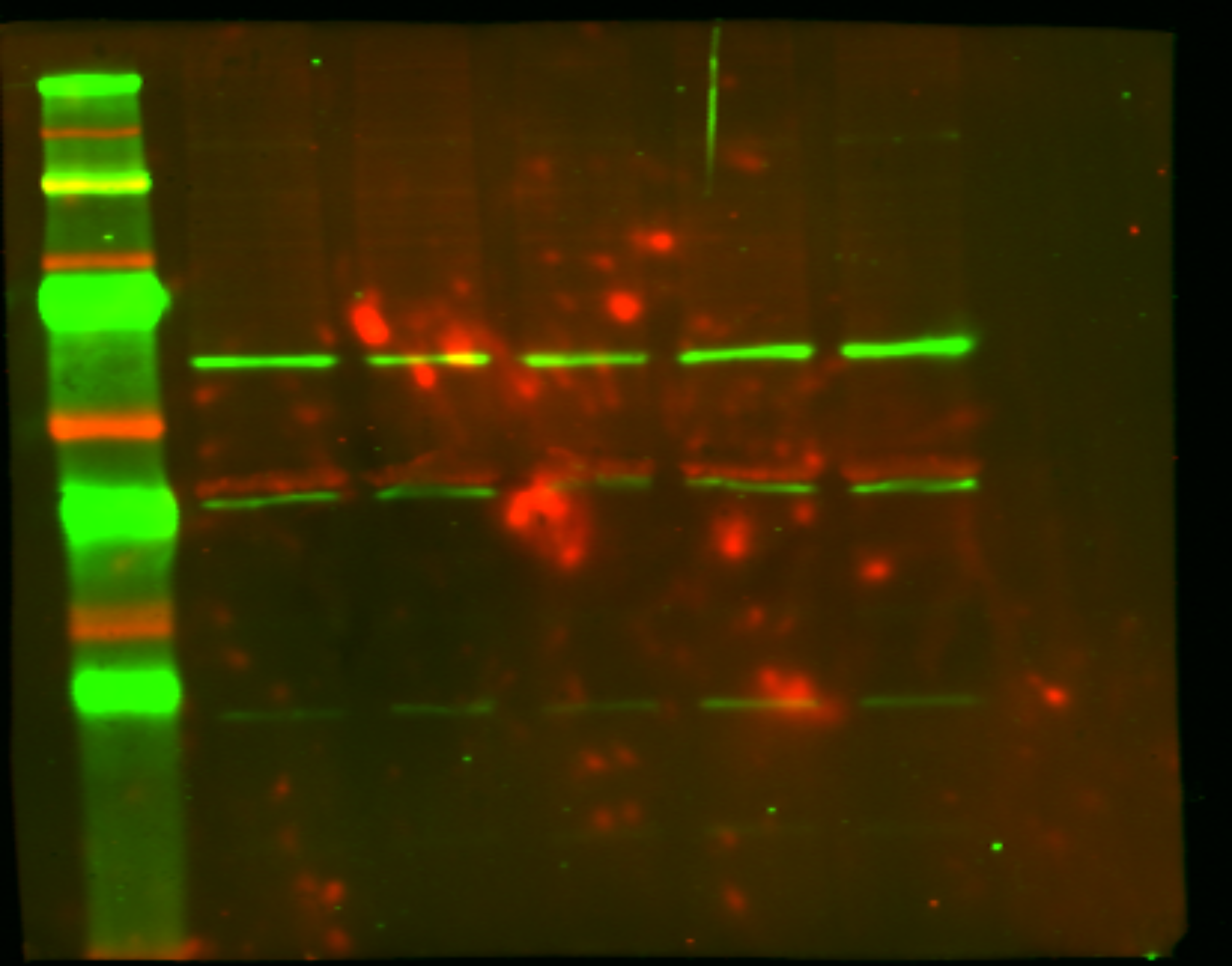

Supplement: Figure 3—figure supplement 6—source data 1. [file elife-82184-fig3-figsupp6-data1.zip › Figure 3-figure supplement 6-source data/B_KRasG13C/3/3_KRasG13C_tAkt.tif]

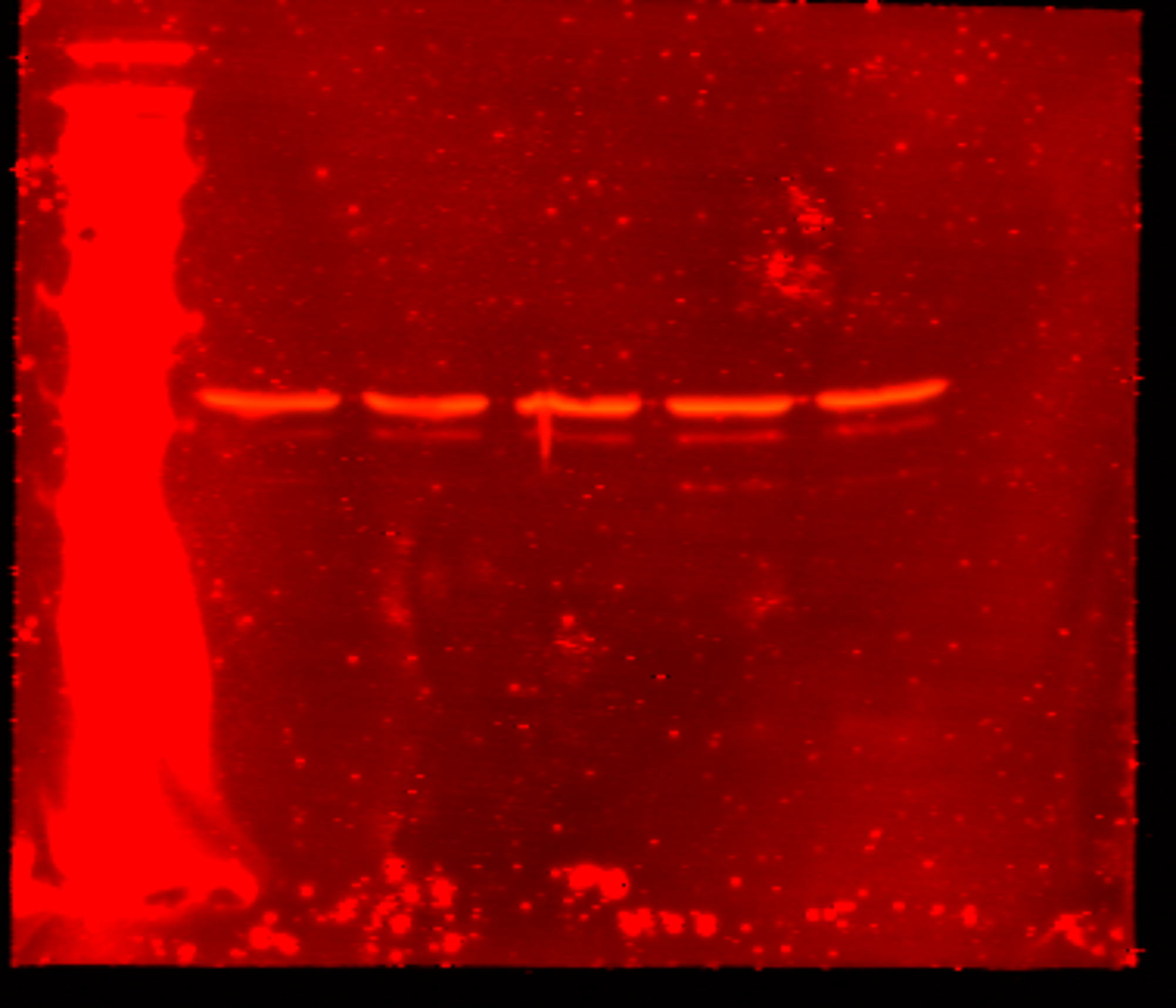

Supplement: Figure 3—figure supplement 6—source data 1. [file elife-82184-fig3-figsupp6-data1.zip › Figure 3-figure supplement 6-source data/B_KRasG13C/3/3_KRasG13C_tErk.tif]

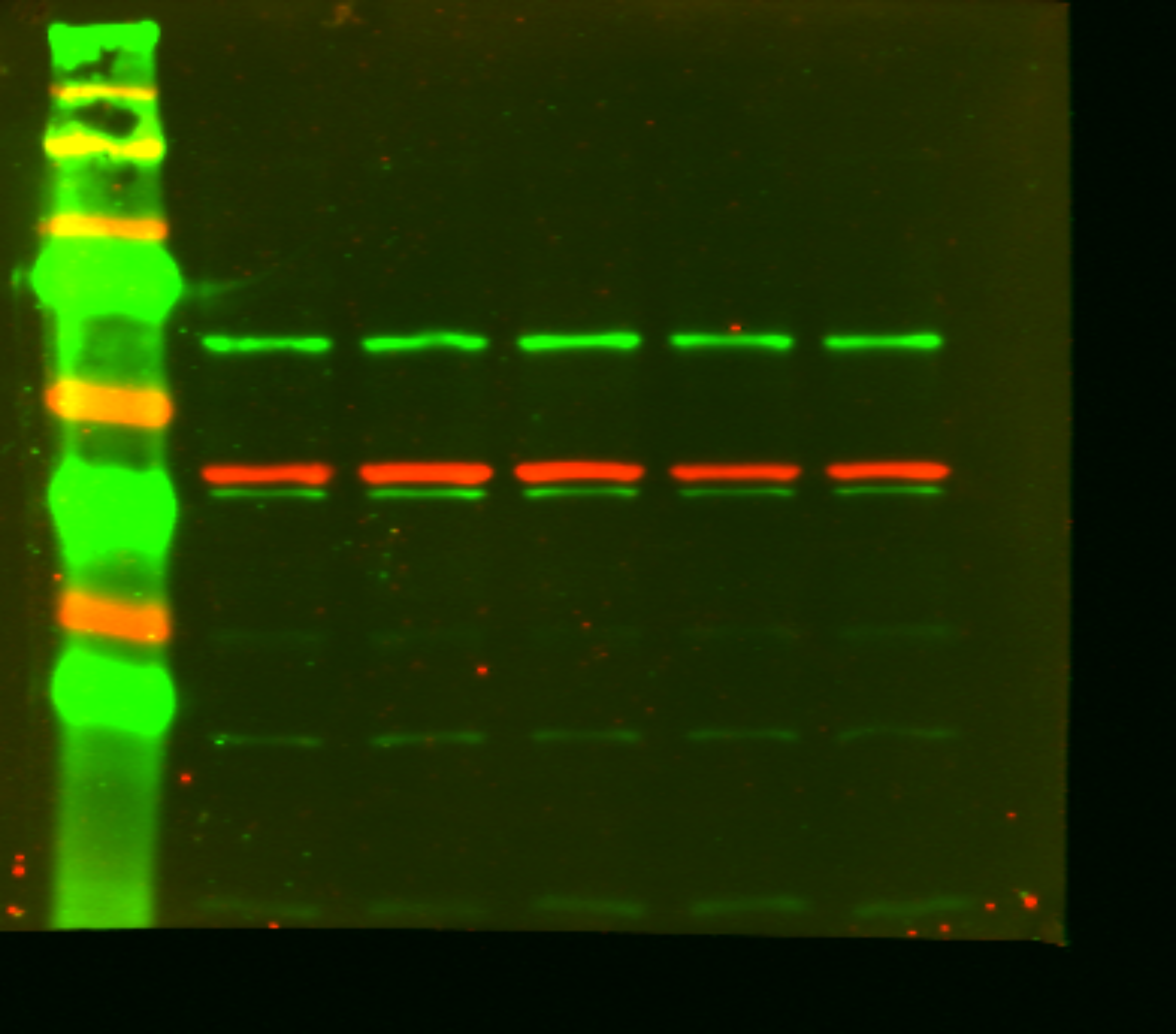

Supplement: Figure 3—figure supplement 6—source data 1. [file elife-82184-fig3-figsupp6-data1.zip › Figure 3-figure supplement 6-source data/C_KRasG13C-edaGDP/1/1_KRasG13C-edaGDP_tAkt.tif]

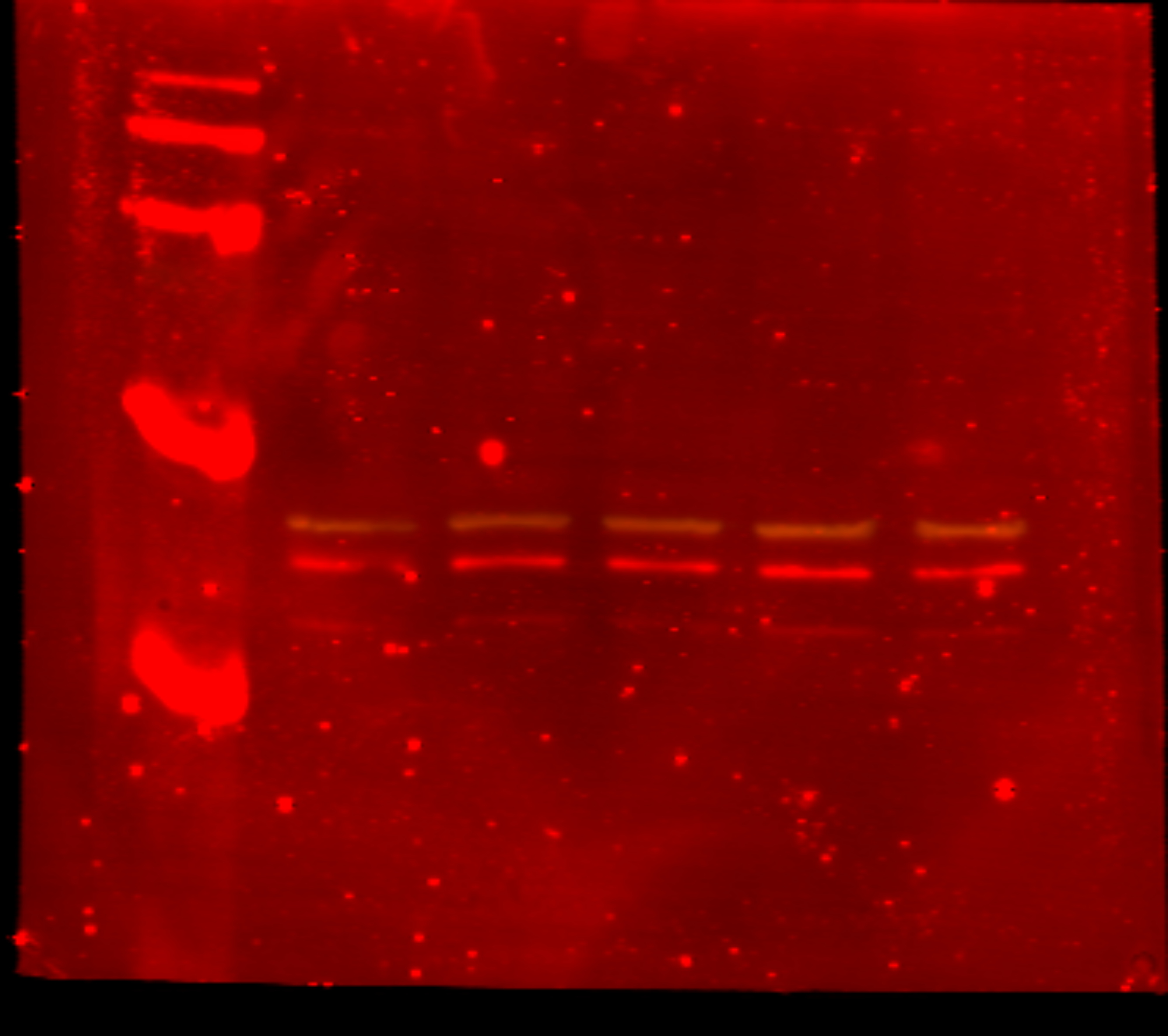

Supplement: Figure 3—figure supplement 6—source data 1. [file elife-82184-fig3-figsupp6-data1.zip › Figure 3-figure supplement 6-source data/C_KRasG13C-edaGDP/1/1_KRasG13C-edaGDP_tErk.tif]

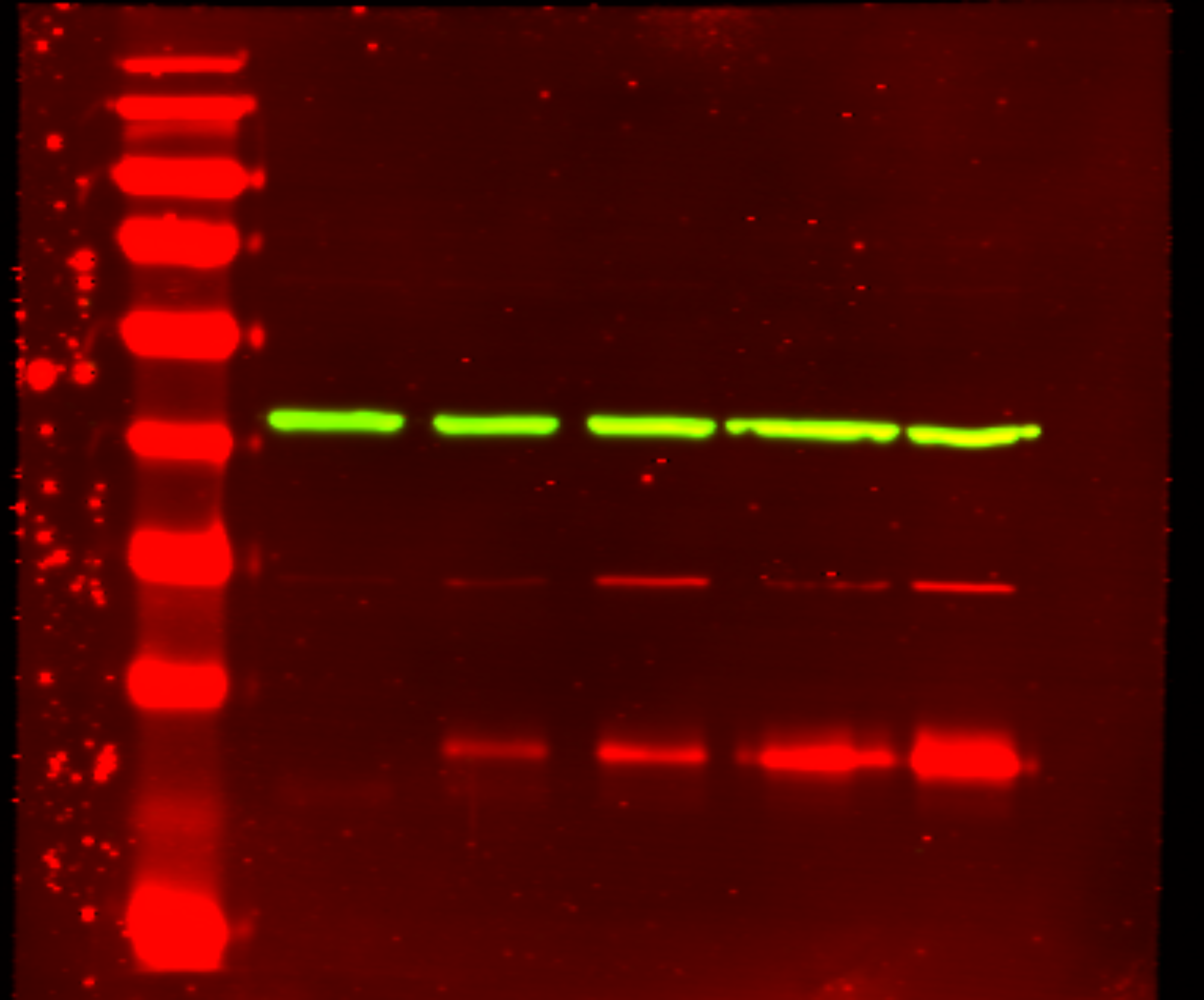

Supplement: Figure 3—figure supplement 6—source data 1. [file elife-82184-fig3-figsupp6-data1.zip › Figure 3-figure supplement 6-source data/C_KRasG13C-edaGDP/2/2_KRasG13C-edaGDP_KRas.tif]

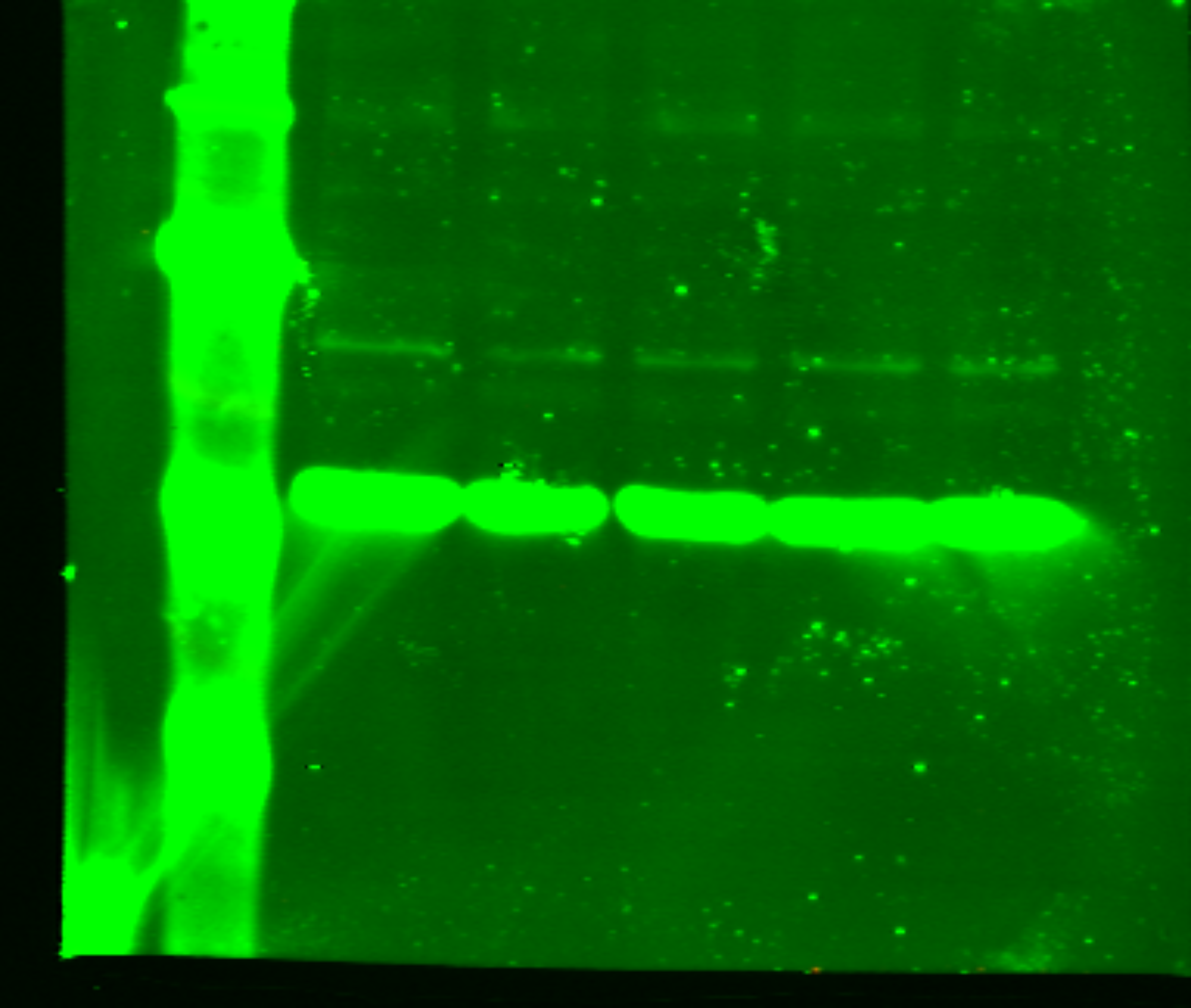

Supplement: Figure 3—figure supplement 6—source data 1. [file elife-82184-fig3-figsupp6-data1.zip › Figure 3-figure supplement 6-source data/C_KRasG13C-edaGDP/2/2_KRasG13C-edaGDP_pAkt.tif]

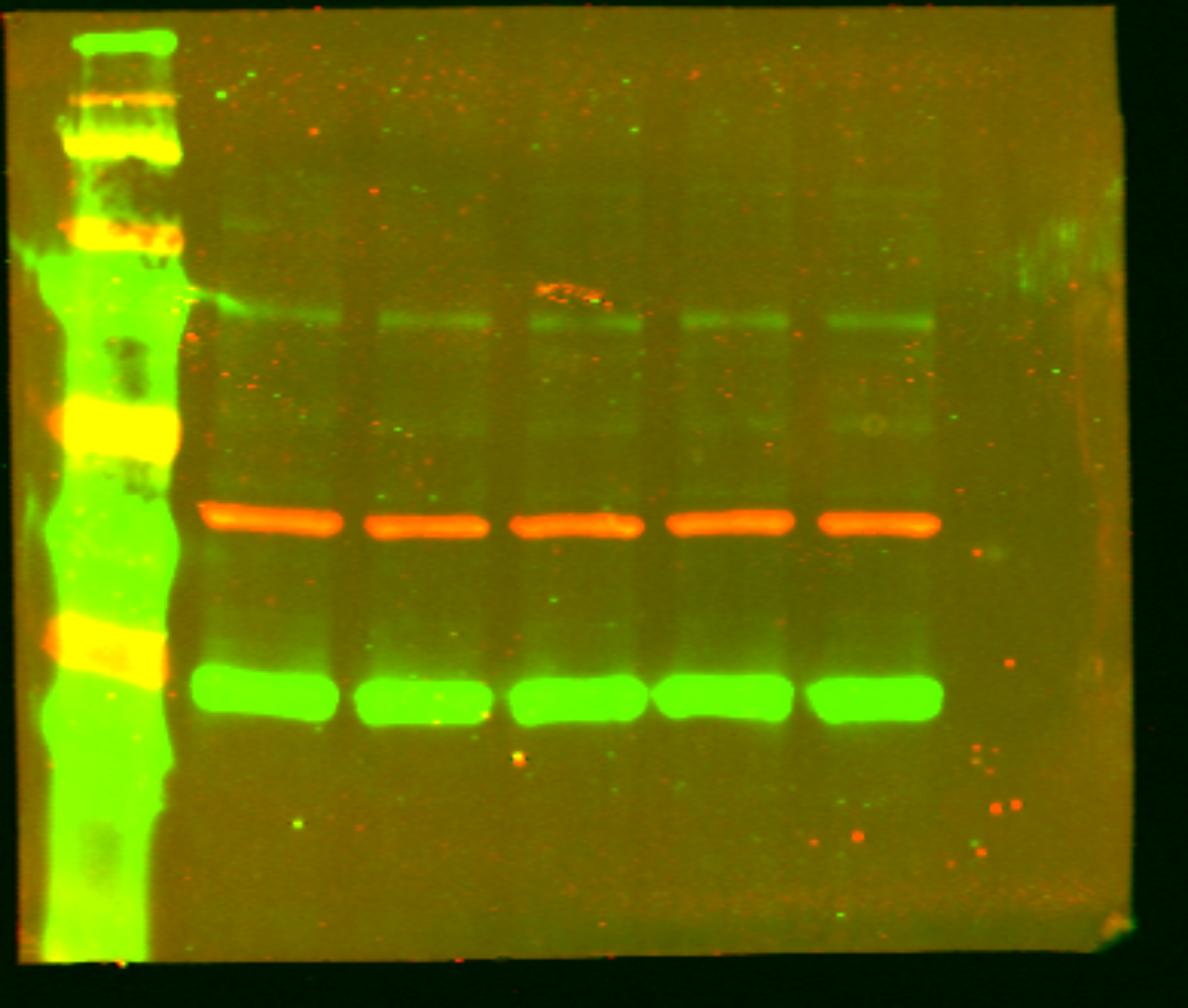

Supplement: Figure 3—figure supplement 6—source data 1. [file elife-82184-fig3-figsupp6-data1.zip › Figure 3-figure supplement 6-source data/C_KRasG13C-edaGDP/2/2_KRasG13C-edaGDP_pcRaf_pS6.tif]

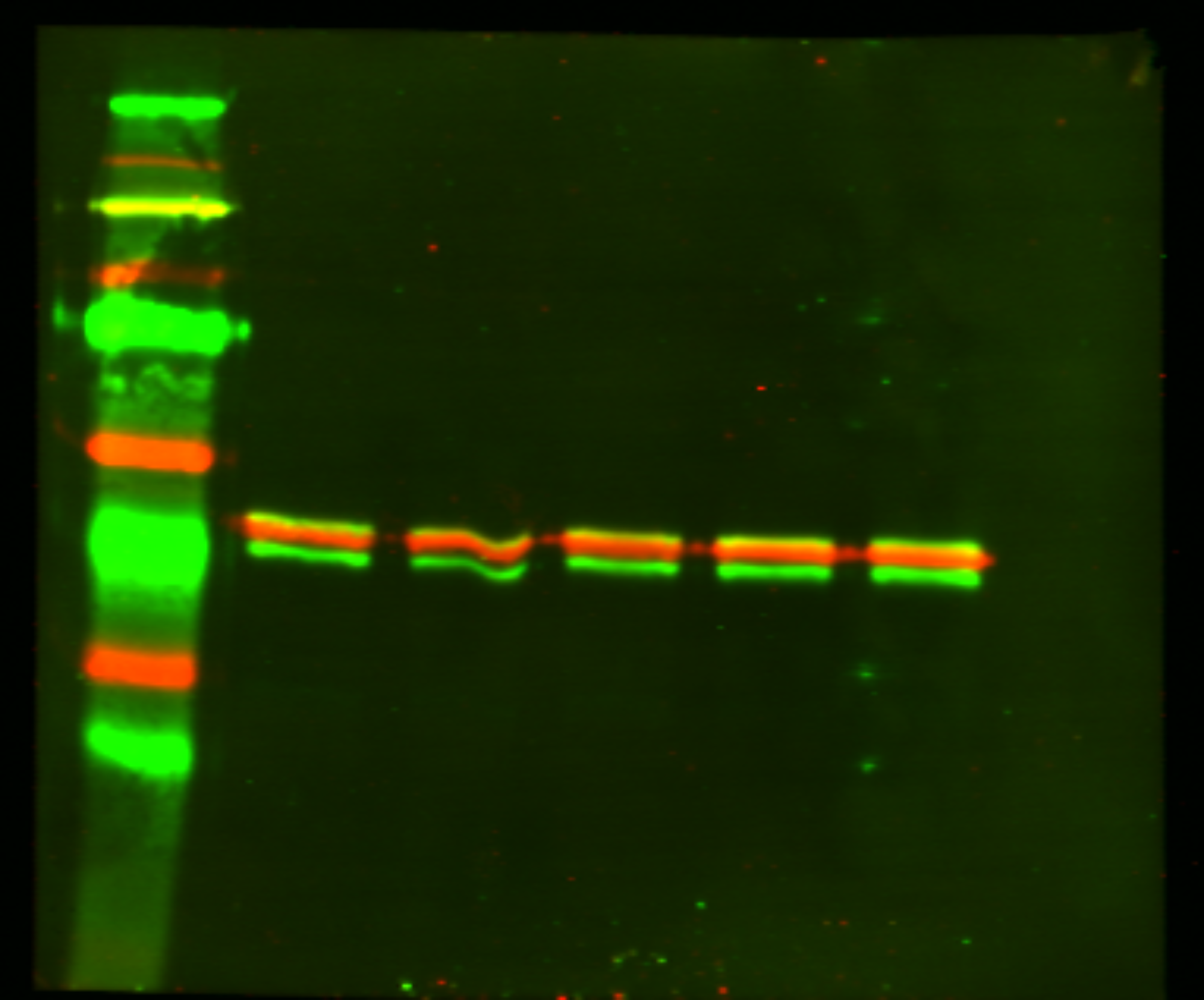

Supplement: Figure 3—figure supplement 6—source data 1. [file elife-82184-fig3-figsupp6-data1.zip › Figure 3-figure supplement 6-source data/C_KRasG13C-edaGDP/2/2_KRasG13C-edaGDP_pErk.tif]

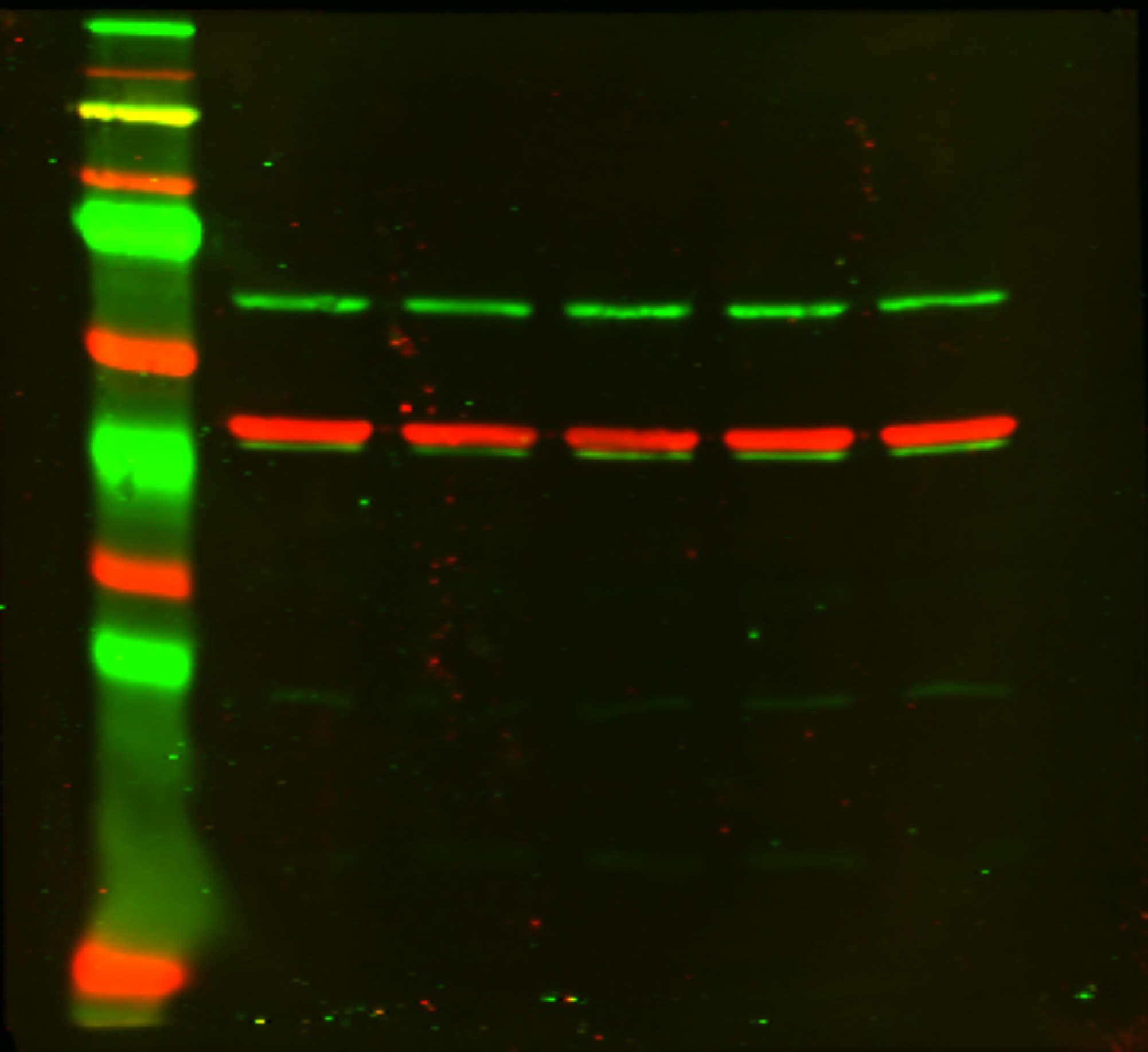

Supplement: Figure 3—figure supplement 6—source data 1. [file elife-82184-fig3-figsupp6-data1.zip › Figure 3-figure supplement 6-source data/C_KRasG13C-edaGDP/2/2_KRasG13C-edaGDP_tAkt.tif]

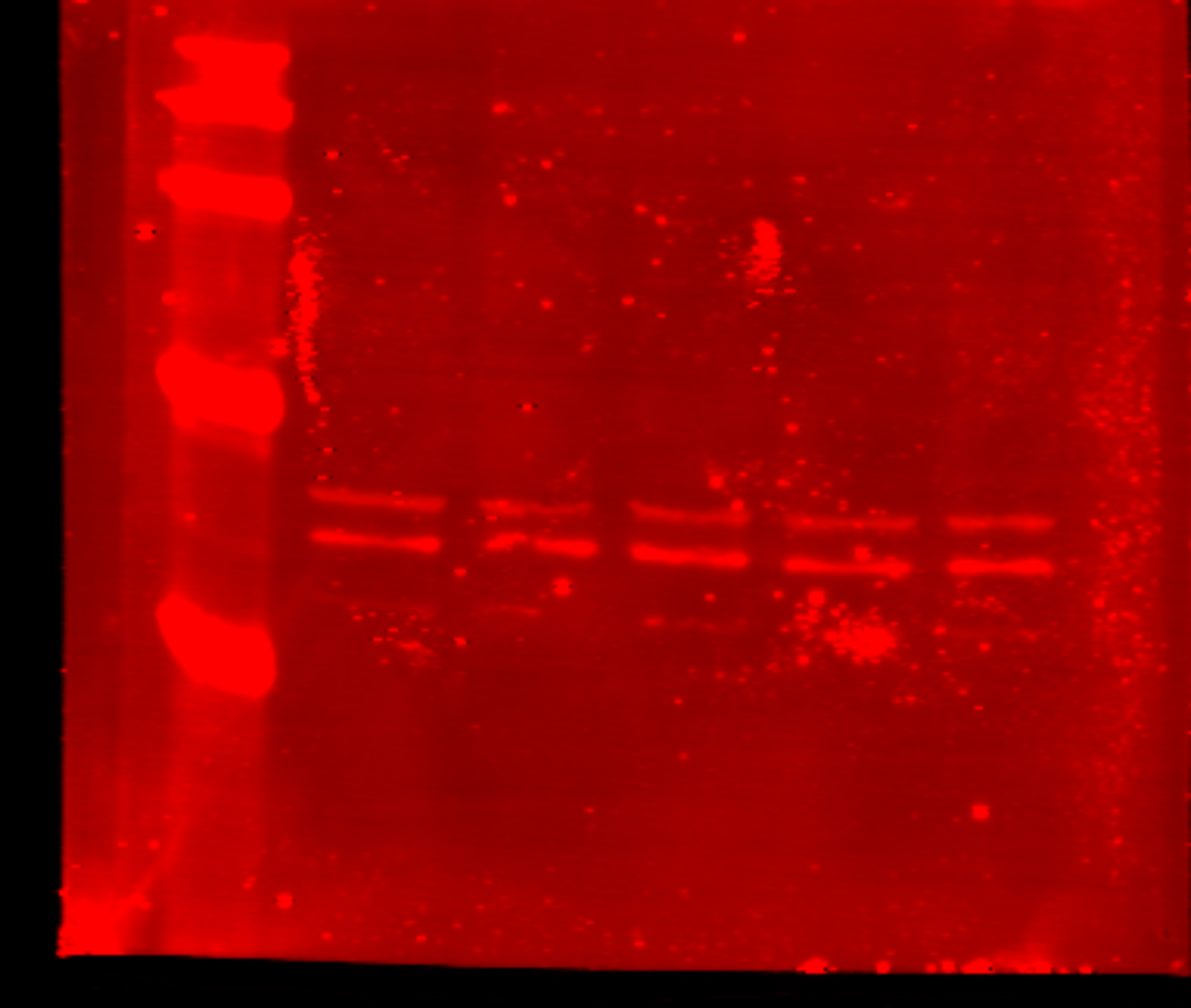

Supplement: Figure 3—figure supplement 6—source data 1. [file elife-82184-fig3-figsupp6-data1.zip › Figure 3-figure supplement 6-source data/C_KRasG13C-edaGDP/2/2_KRasG13C-edaGDP_tErk.tif]

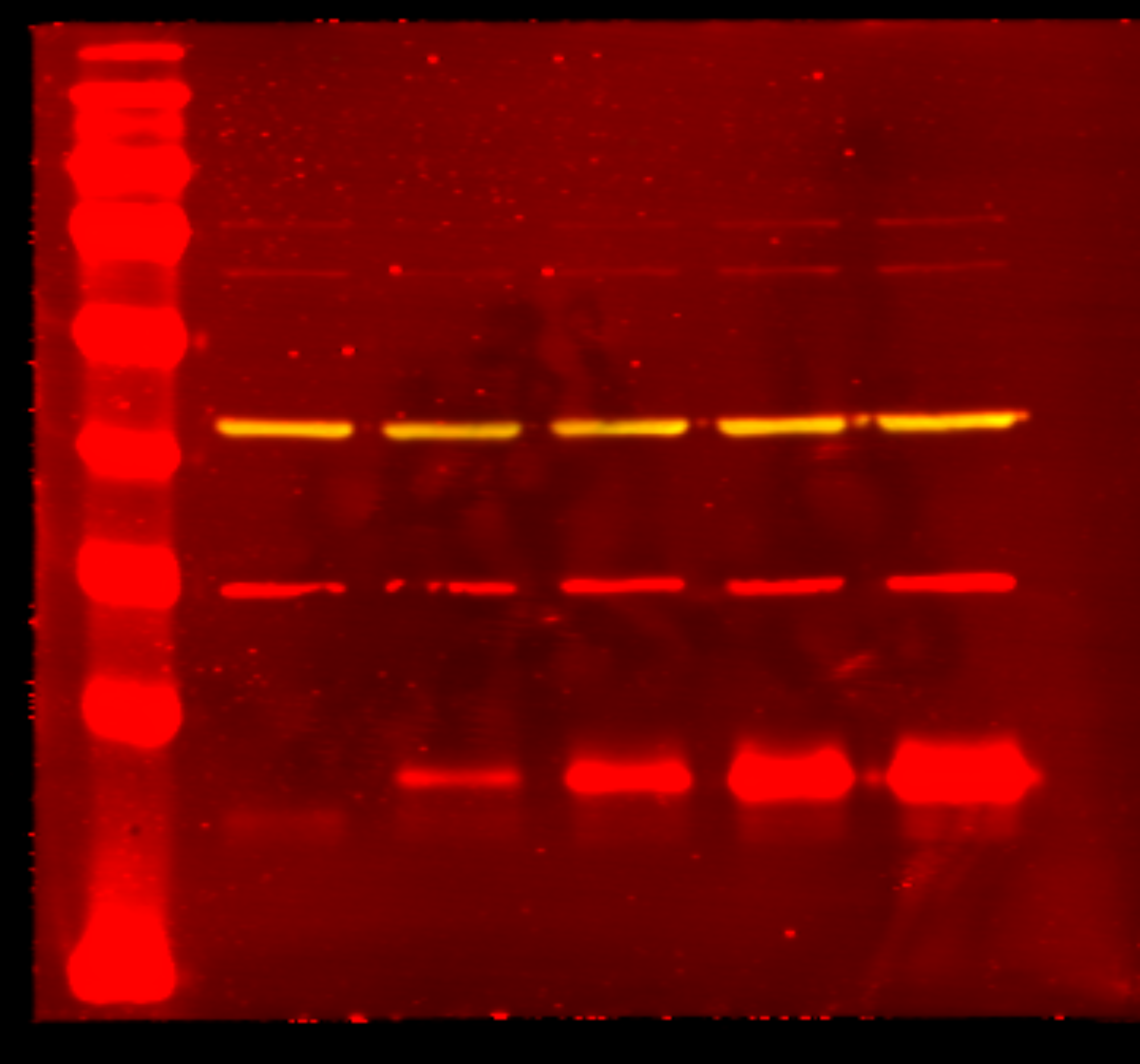

Supplement: Figure 3—figure supplement 6—source data 1. [file elife-82184-fig3-figsupp6-data1.zip › Figure 3-figure supplement 6-source data/C_KRasG13C-edaGDP/3/3_KRasG13C-edaGDP_KRas.tif]

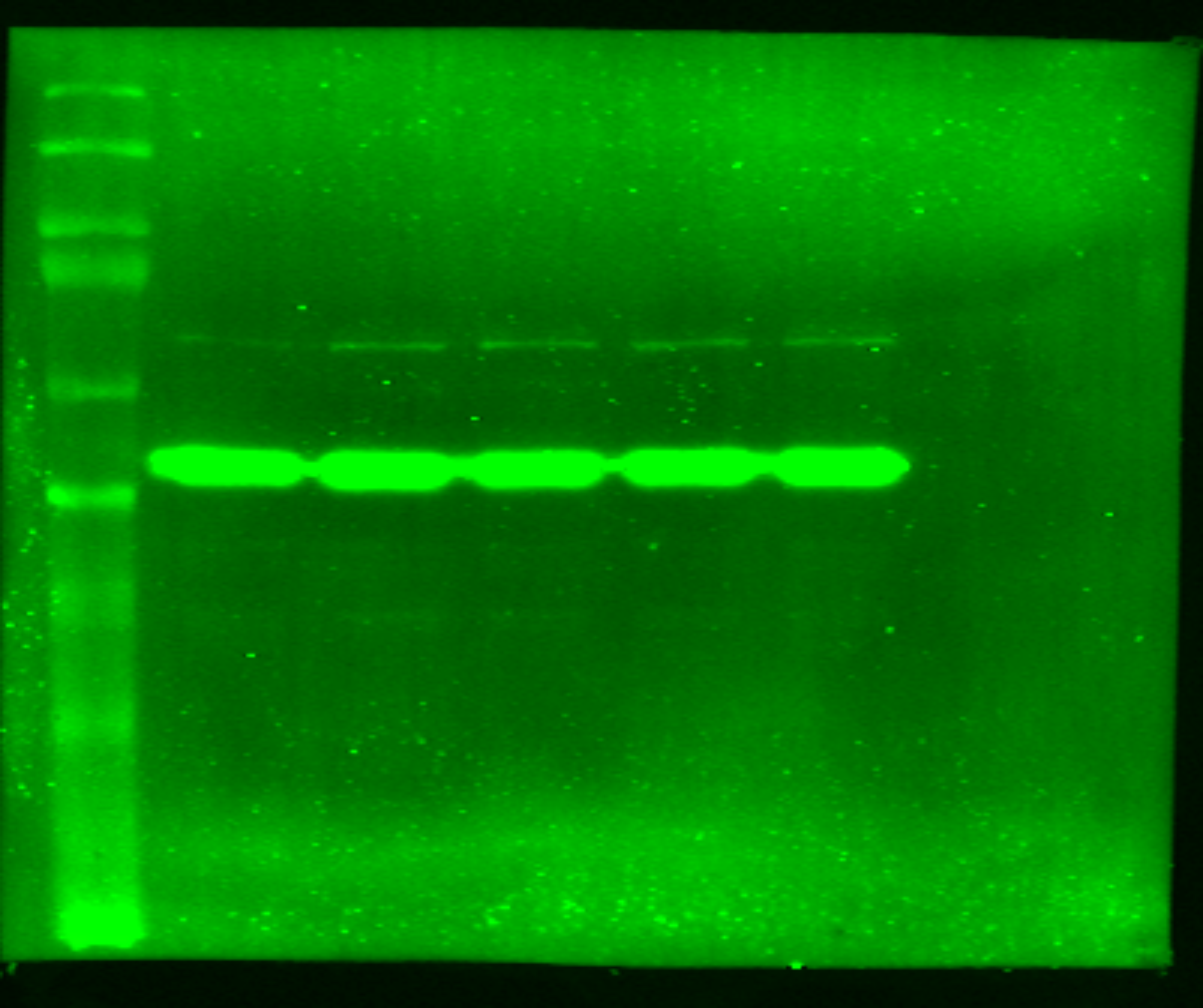

Supplement: Figure 3—figure supplement 6—source data 1. [file elife-82184-fig3-figsupp6-data1.zip › Figure 3-figure supplement 6-source data/C_KRasG13C-edaGDP/3/3_KRasG13C-edaGDP_pAkt.tif]

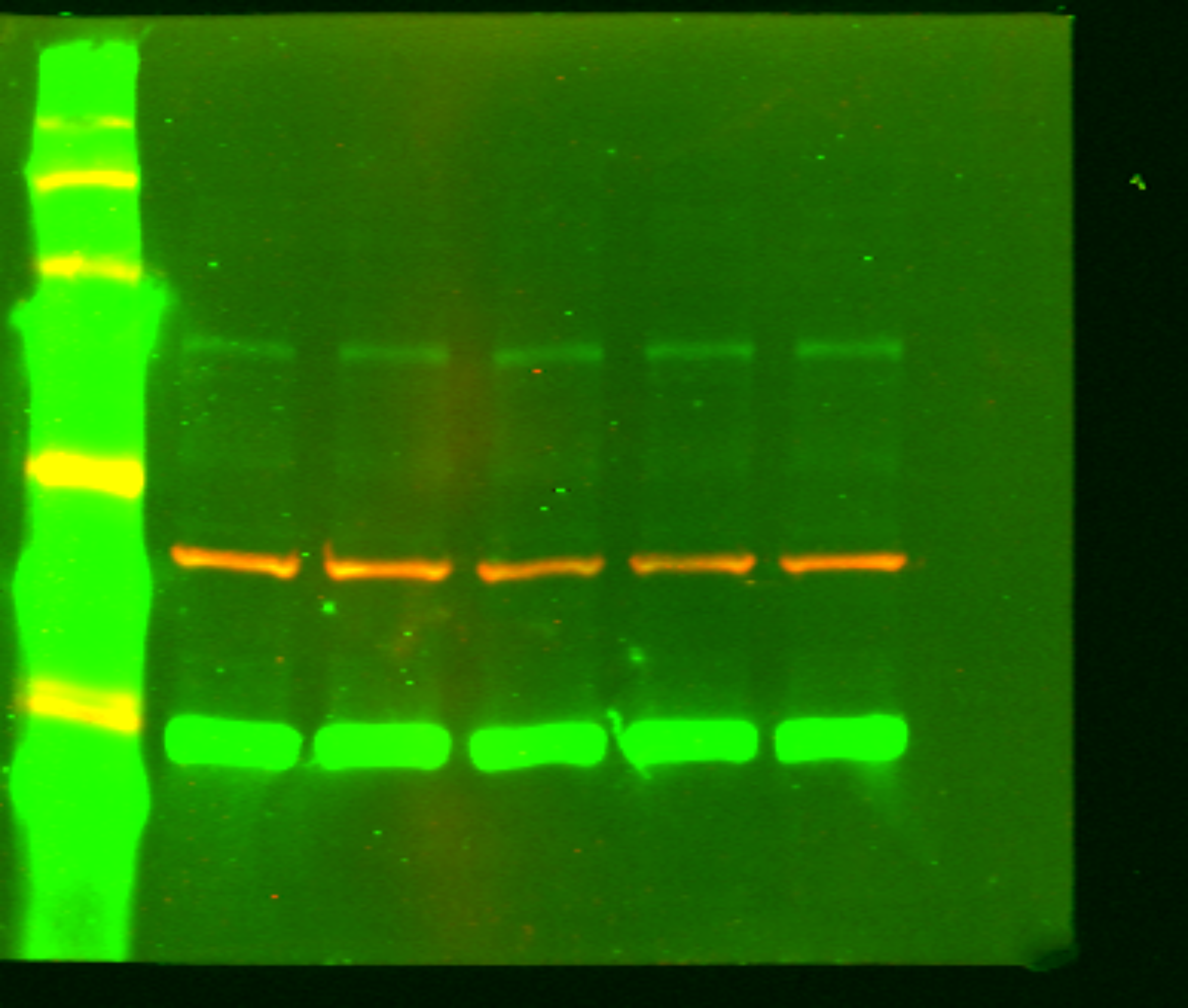

Supplement: Figure 3—figure supplement 6—source data 1. [file elife-82184-fig3-figsupp6-data1.zip › Figure 3-figure supplement 6-source data/C_KRasG13C-edaGDP/3/3_KRasG13C-edaGDP_pcRaf_pS6.tif]

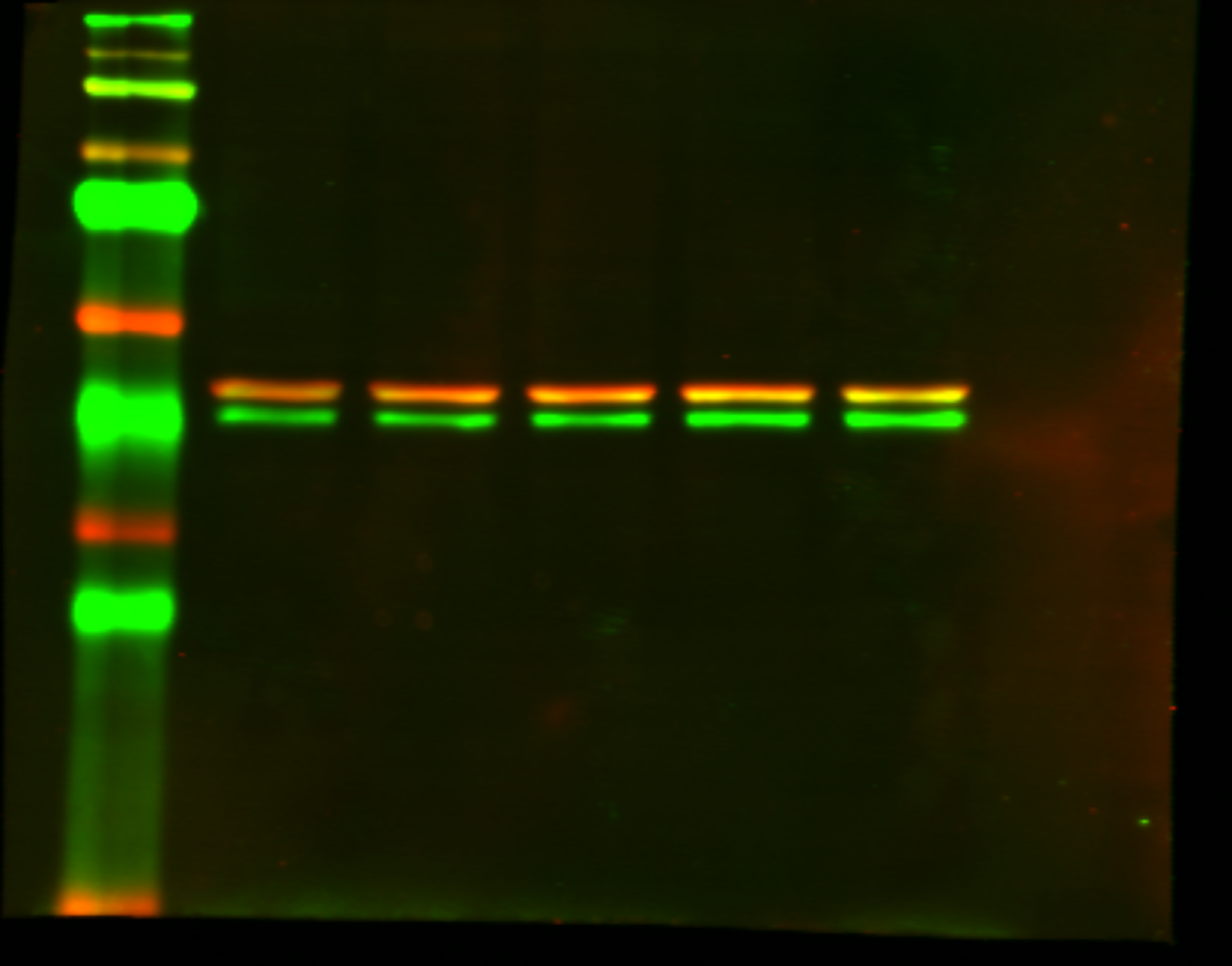

Supplement: Figure 3—figure supplement 6—source data 1. [file elife-82184-fig3-figsupp6-data1.zip › Figure 3-figure supplement 6-source data/C_KRasG13C-edaGDP/3/3_KRasG13C-edaGDP_pErk.tif]

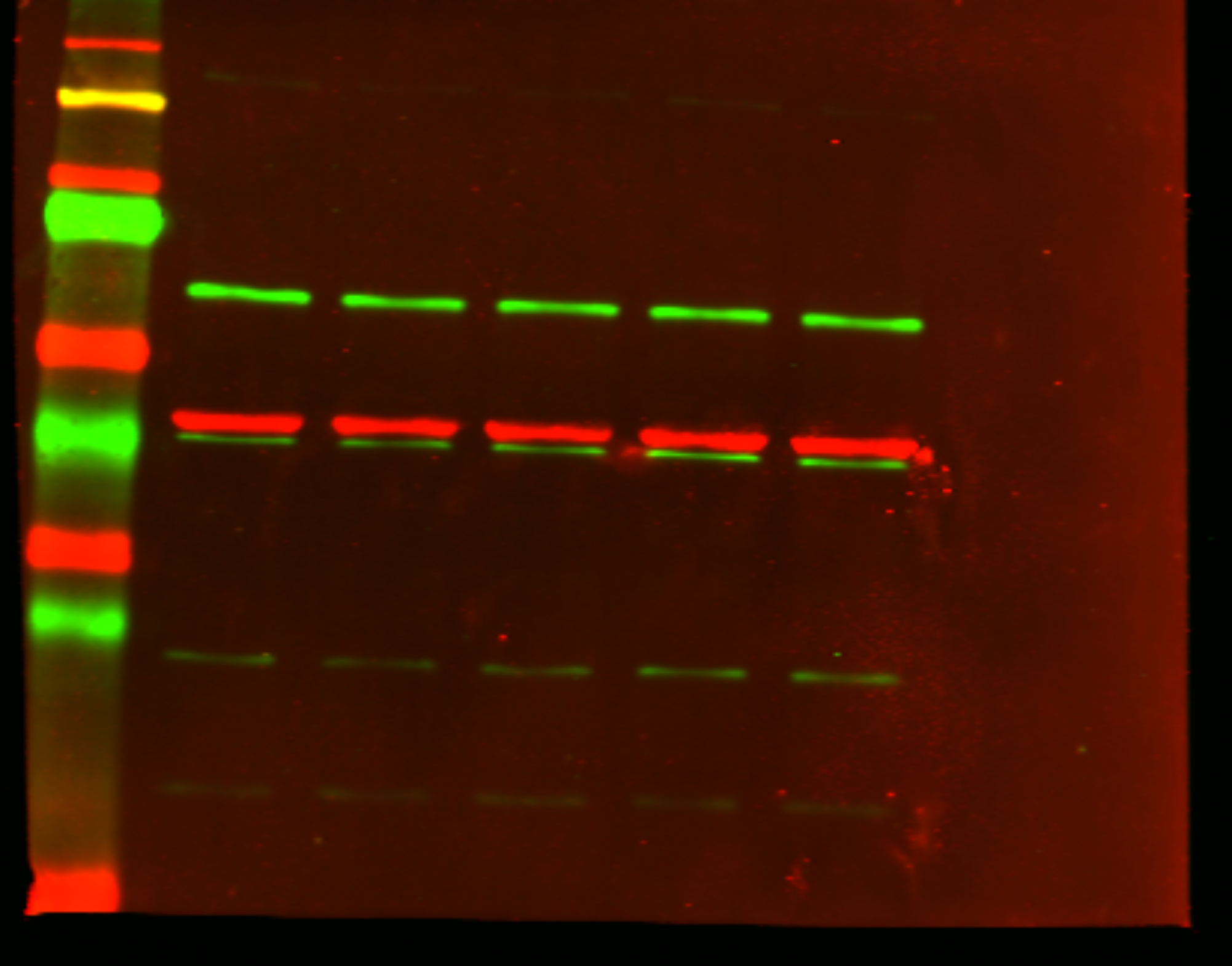

Supplement: Figure 3—figure supplement 6—source data 1. [file elife-82184-fig3-figsupp6-data1.zip › Figure 3-figure supplement 6-source data/C_KRasG13C-edaGDP/3/3_KRasG13C-edaGDP_tAkt.tif]

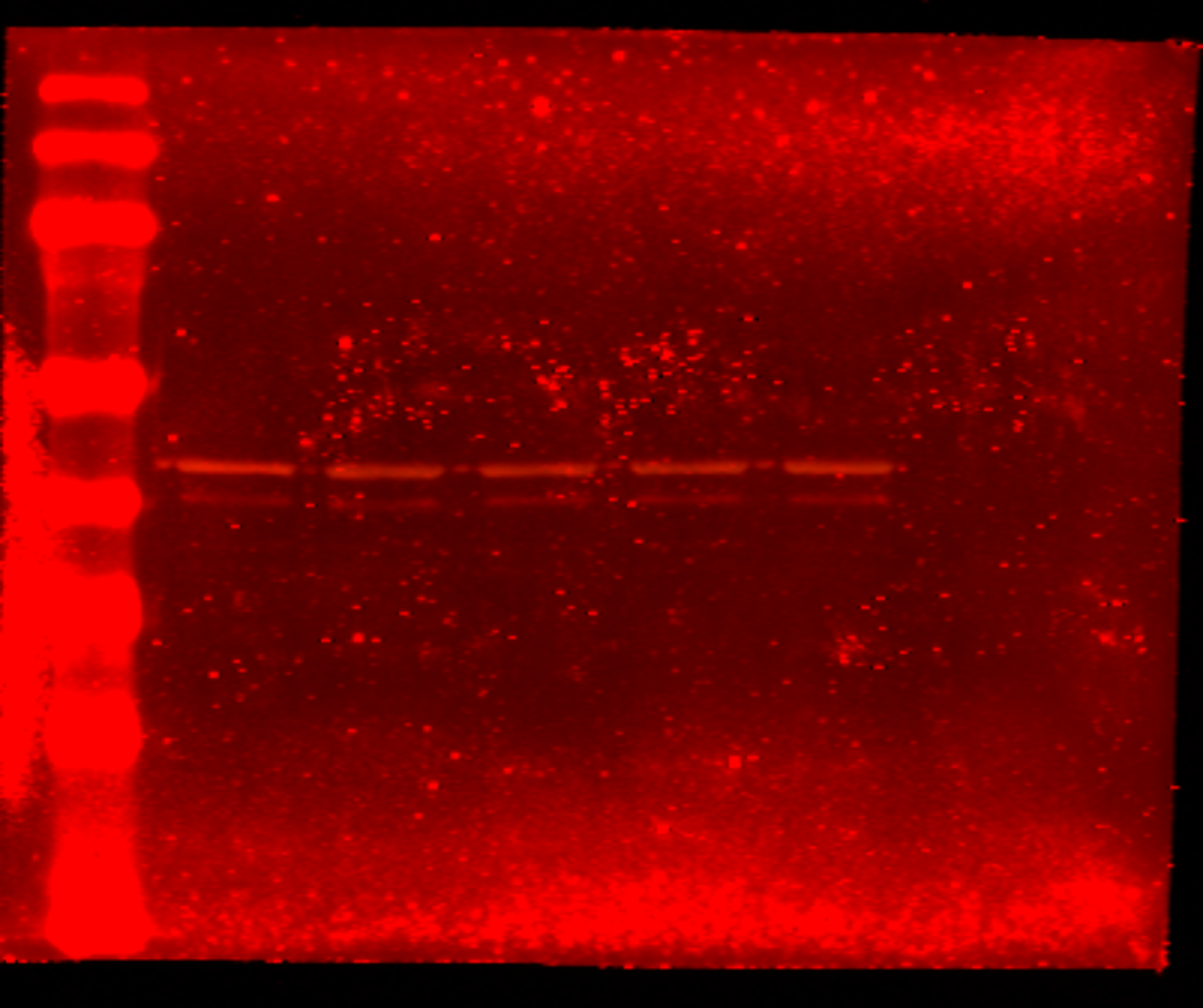

Supplement: Figure 3—figure supplement 6—source data 1. [file elife-82184-fig3-figsupp6-data1.zip › Figure 3-figure supplement 6-source data/C_KRasG13C-edaGDP/3/3_KRasG13C-edaGDP_tErk.tif]

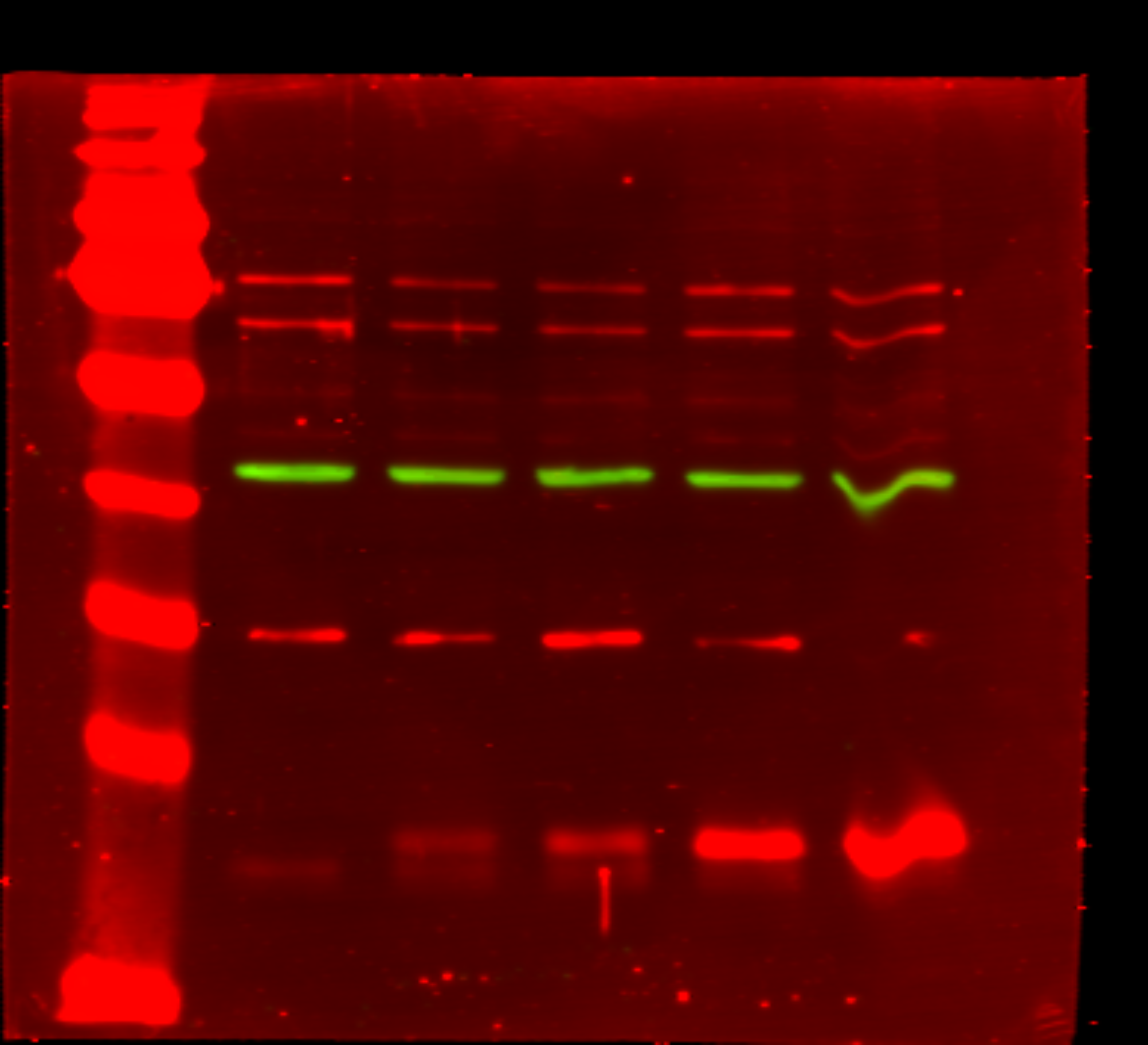

Supplement: Figure 3—figure supplement 8—source data 1. [file elife-82184-fig3-figsupp8-data1.zip › Figure 3-figure supplement 8-source data/A/1/1_KRasG13CacetyledaGDP_KRas.tif]

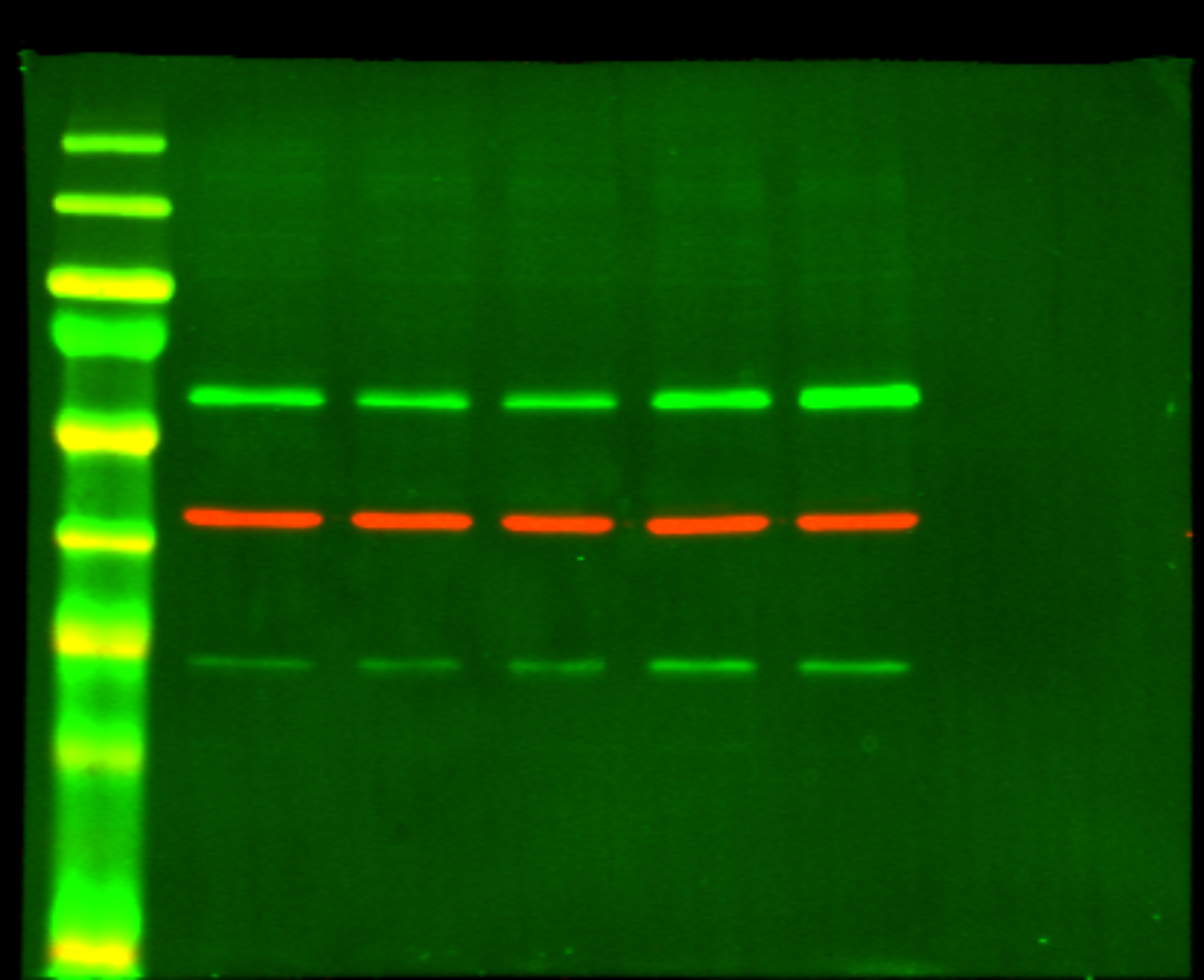

Supplement: Figure 3—figure supplement 8—source data 1. [file elife-82184-fig3-figsupp8-data1.zip › Figure 3-figure supplement 8-source data/A/1/1_KRasG13CacetyledaGDP_pAkt.tif]

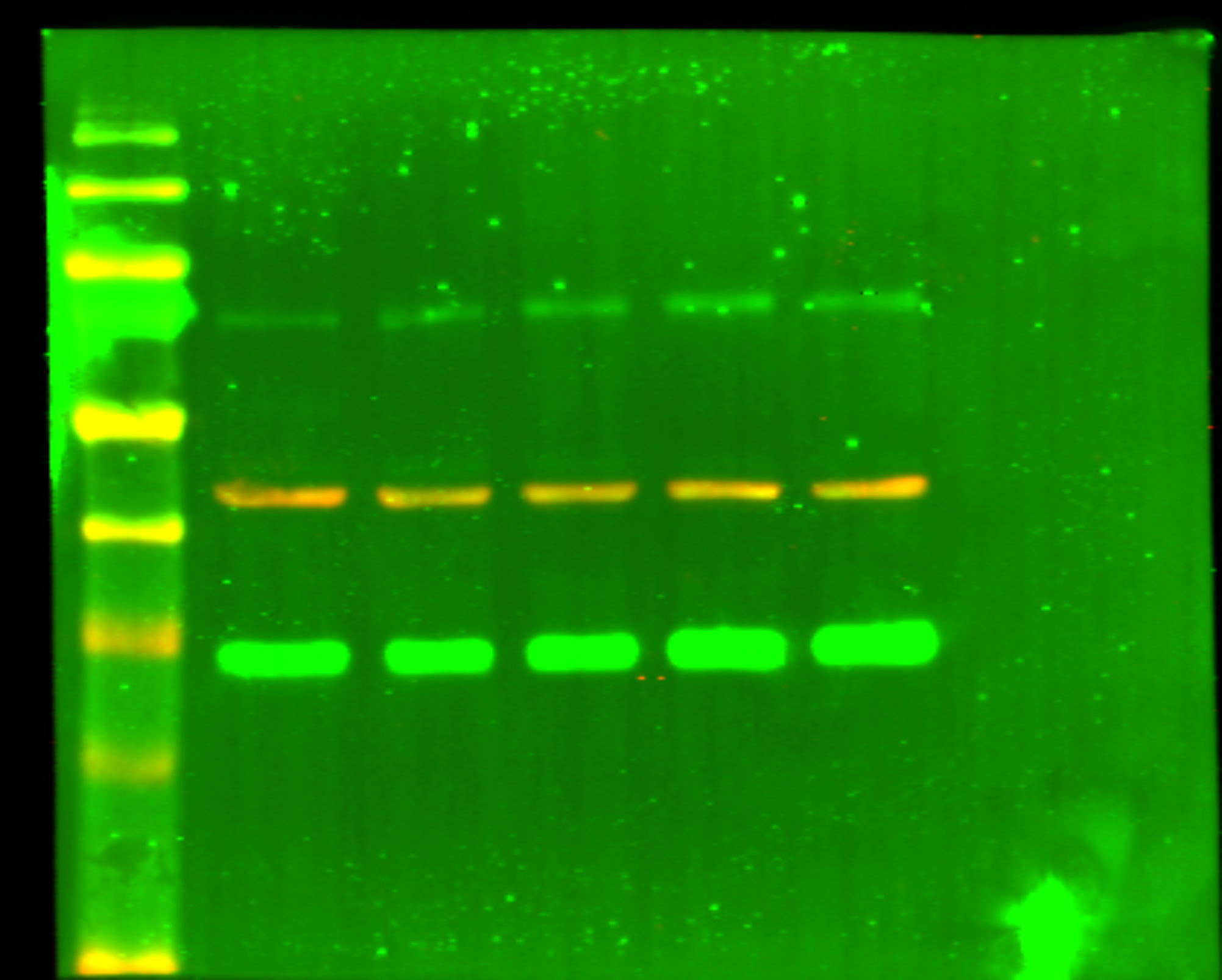

Supplement: Figure 3—figure supplement 8—source data 1. [file elife-82184-fig3-figsupp8-data1.zip › Figure 3-figure supplement 8-source data/A/1/1_KRasG13CacetyledaGDP_pcRaf_pS6.tif]

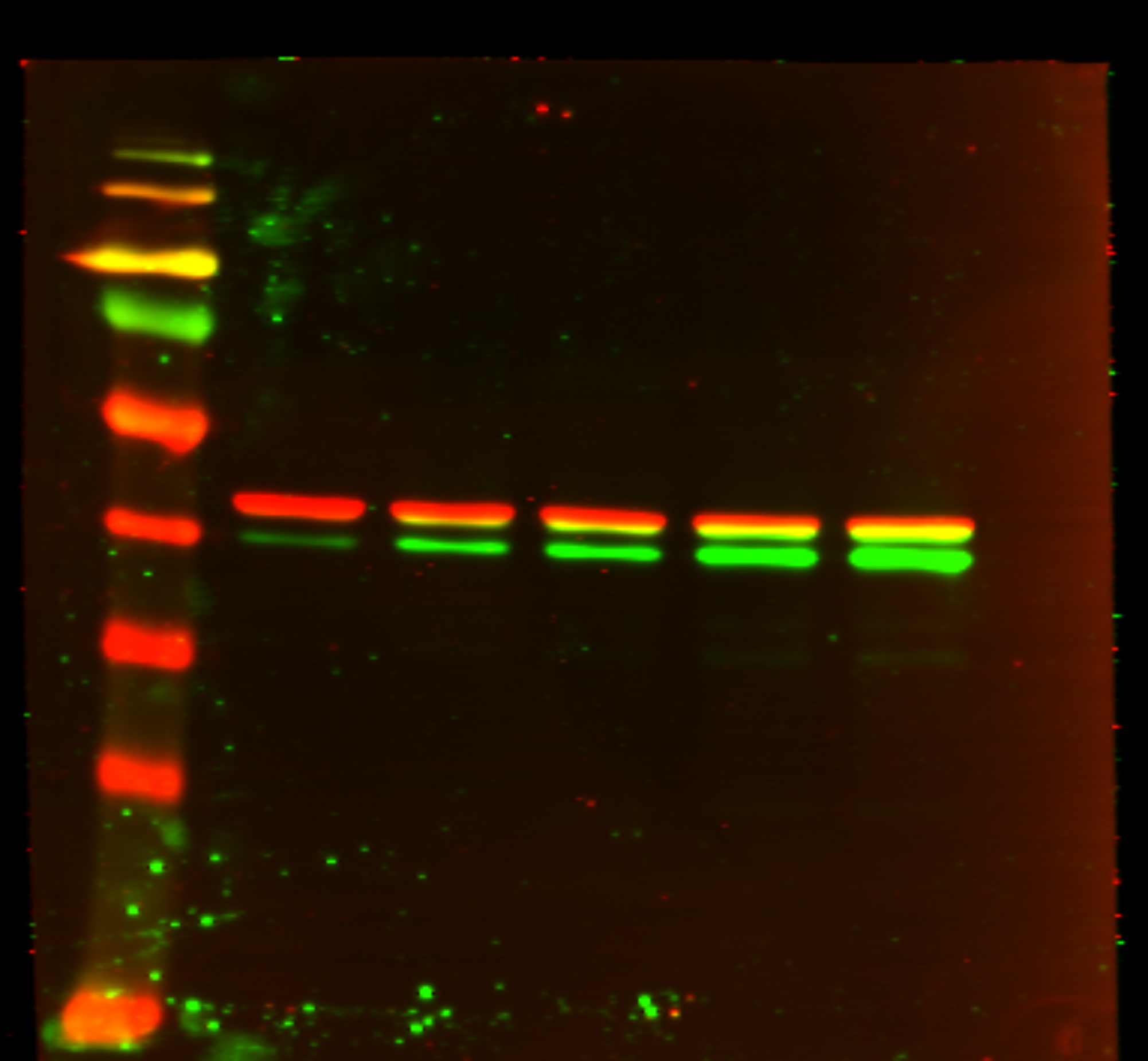

Supplement: Figure 3—figure supplement 8—source data 1. [file elife-82184-fig3-figsupp8-data1.zip › Figure 3-figure supplement 8-source data/A/1/1_KRasG13CacetyledaGDP_pErk.tif]

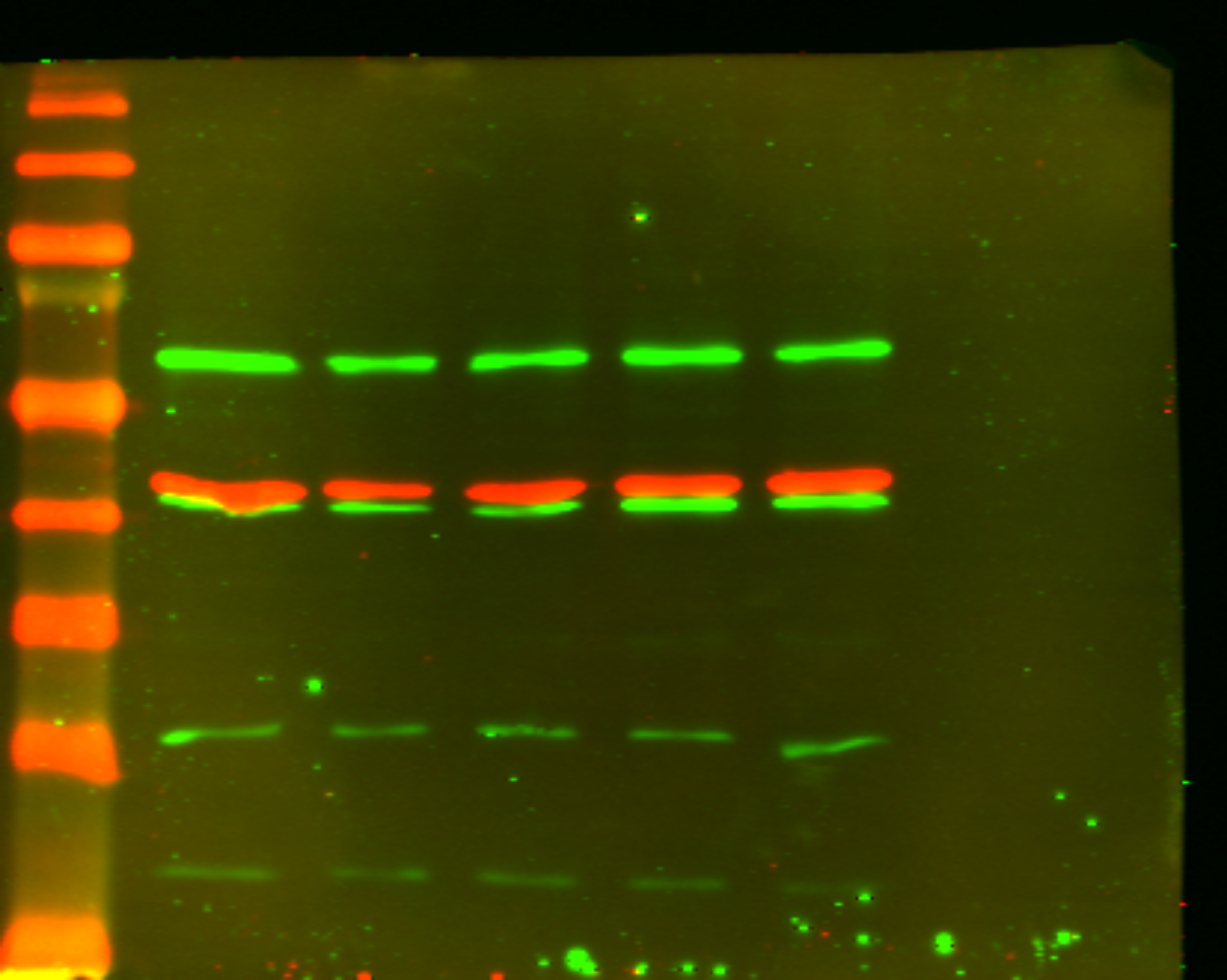

Supplement: Figure 3—figure supplement 8—source data 1. [file elife-82184-fig3-figsupp8-data1.zip › Figure 3-figure supplement 8-source data/A/1/1_KRasG13CacetyledaGDP_tAkt.tif]

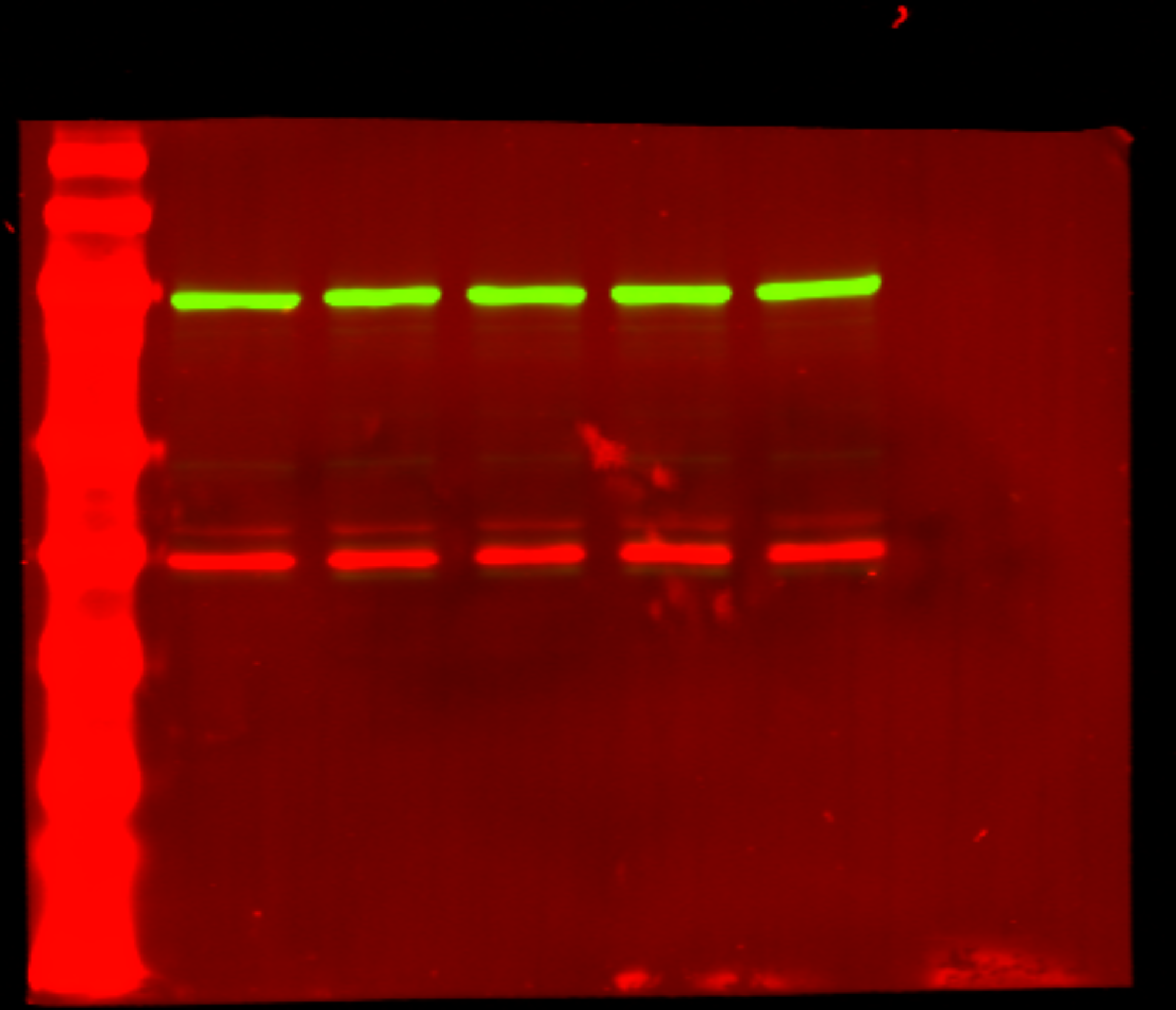

Supplement: Figure 3—figure supplement 8—source data 1. [file elife-82184-fig3-figsupp8-data1.zip › Figure 3-figure supplement 8-source data/A/1/1_KRasG13CacetyledaGDP_tErk.tif]

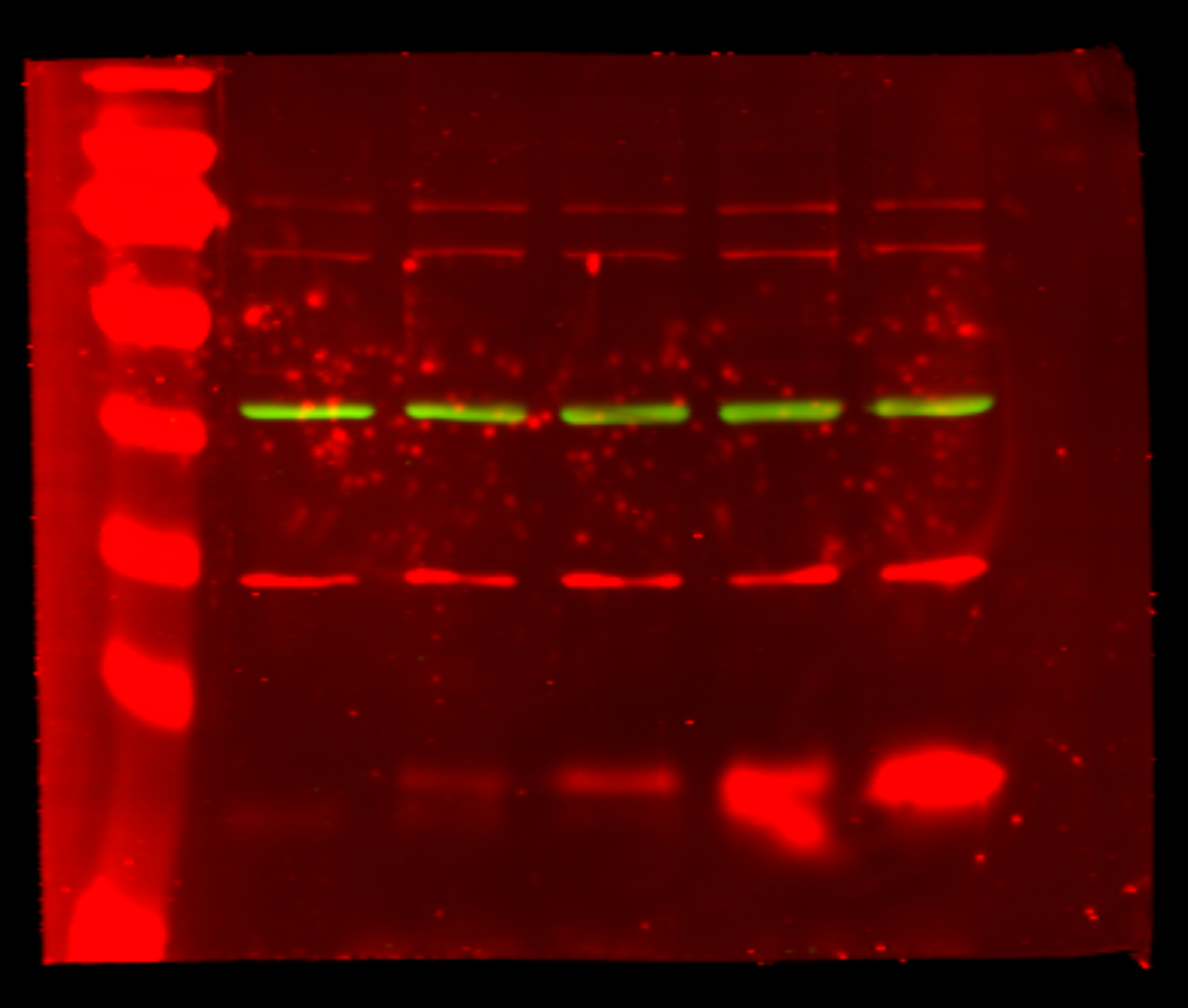

Supplement: Figure 3—figure supplement 8—source data 1. [file elife-82184-fig3-figsupp8-data1.zip › Figure 3-figure supplement 8-source data/A/2/2_KRasG13CacetyledaGDP_KRas.tif]

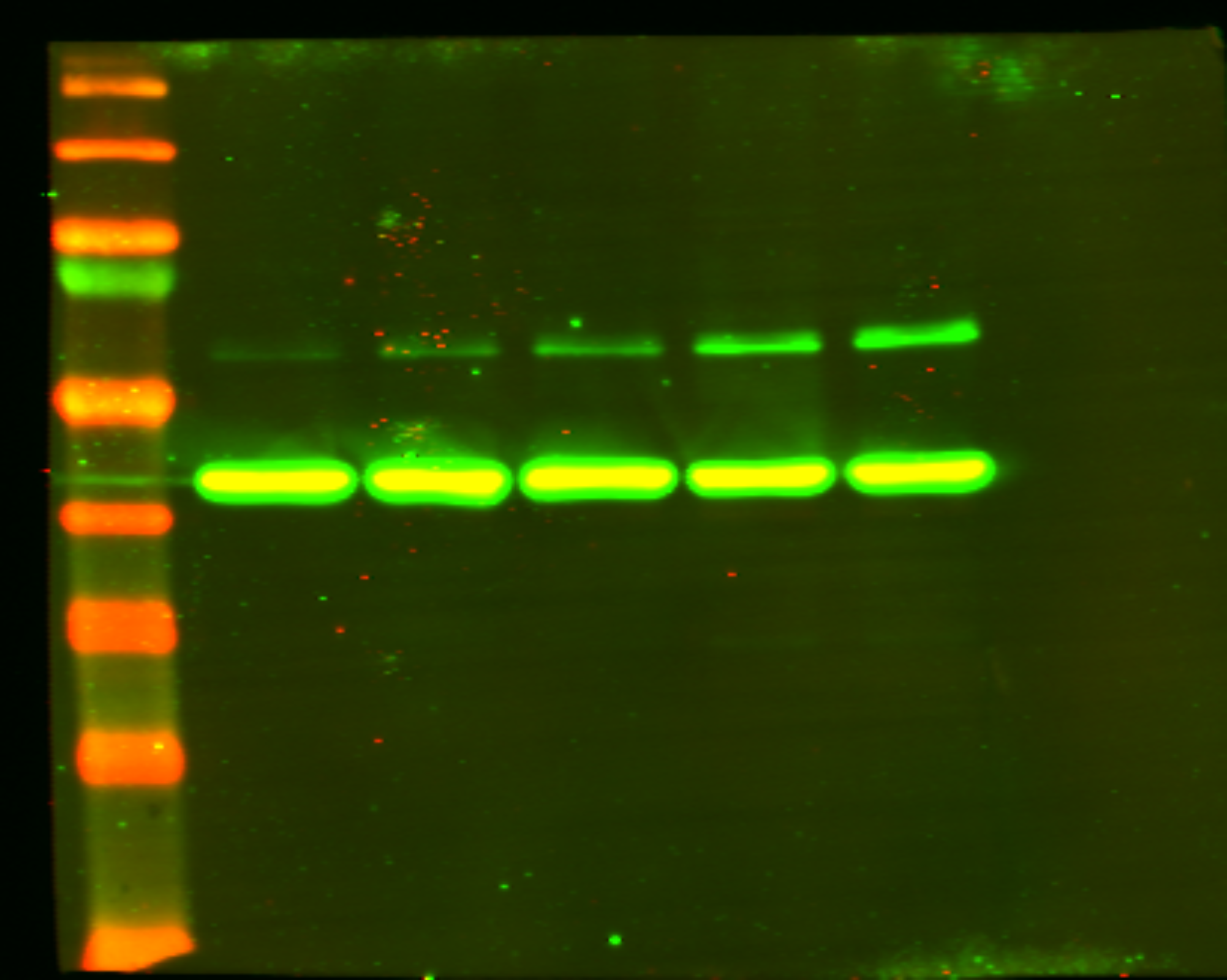

Supplement: Figure 3—figure supplement 8—source data 1. [file elife-82184-fig3-figsupp8-data1.zip › Figure 3-figure supplement 8-source data/A/2/2_KRasG13CacetyledaGDP_pAkt.tif]

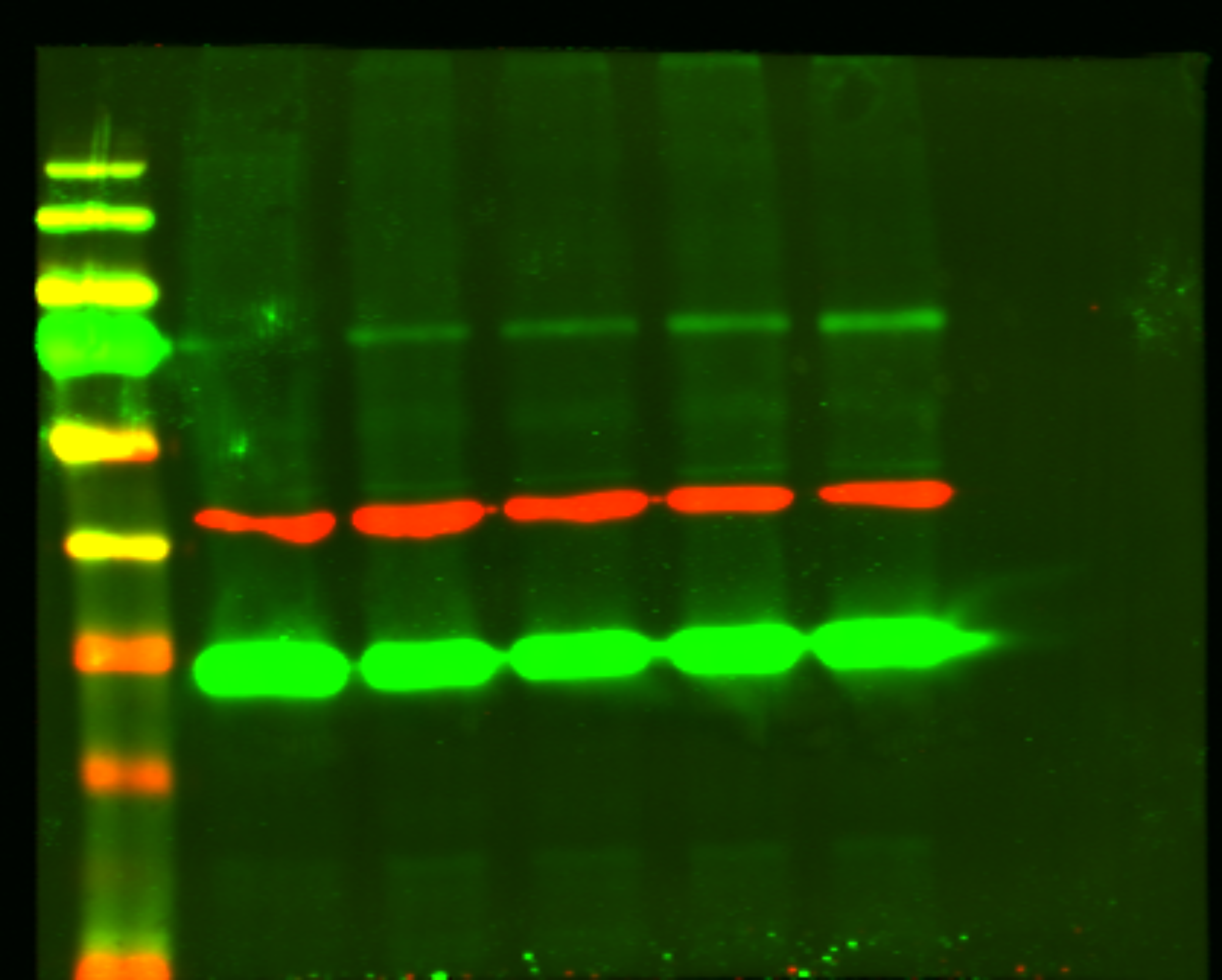

Supplement: Figure 3—figure supplement 8—source data 1. [file elife-82184-fig3-figsupp8-data1.zip › Figure 3-figure supplement 8-source data/A/2/2_KRasG13CacetyledaGDP_pcRaf.tif]

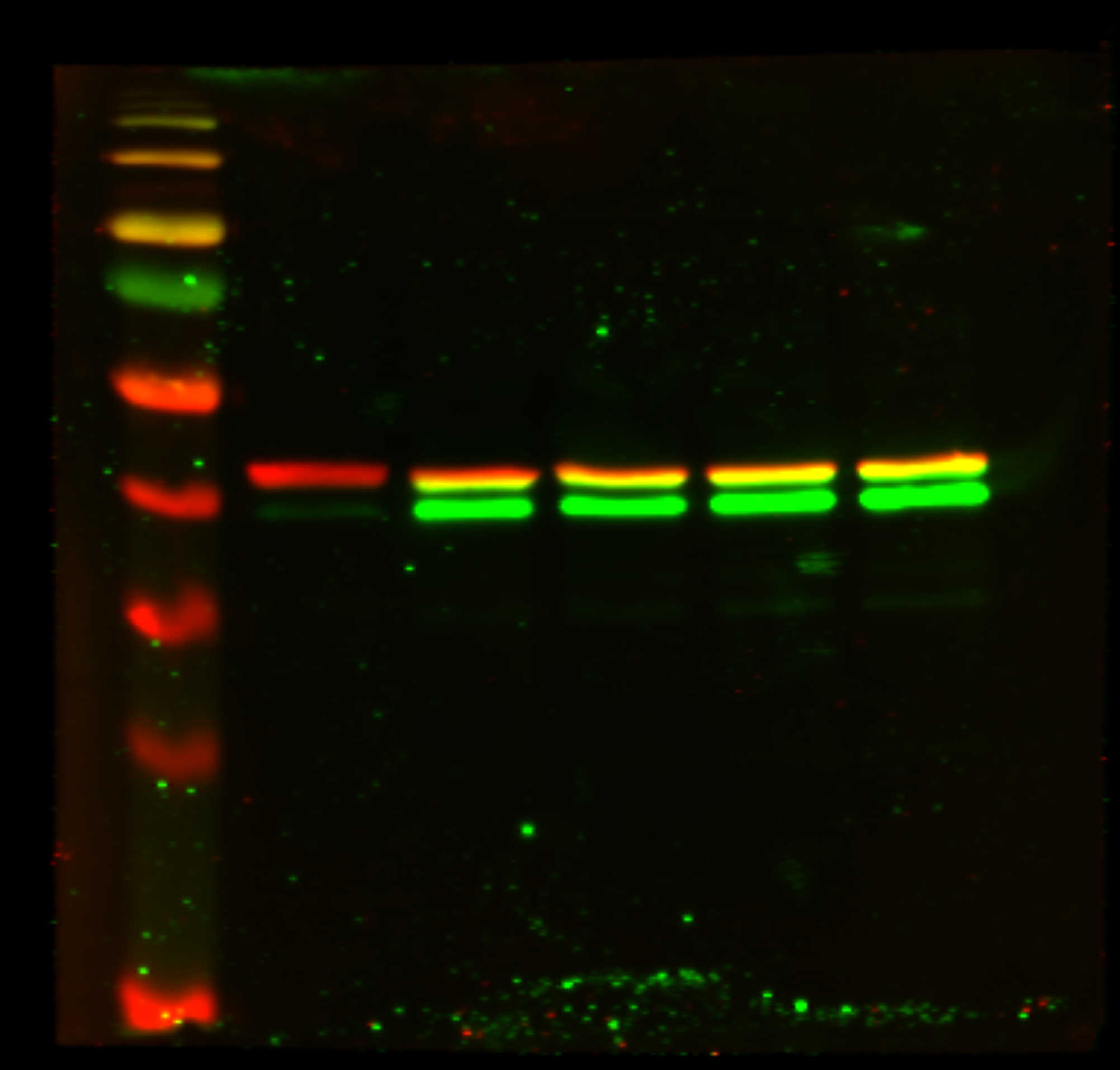

Supplement: Figure 3—figure supplement 8—source data 1. [file elife-82184-fig3-figsupp8-data1.zip › Figure 3-figure supplement 8-source data/A/2/2_KRasG13CacetyledaGDP_pErk.tif]

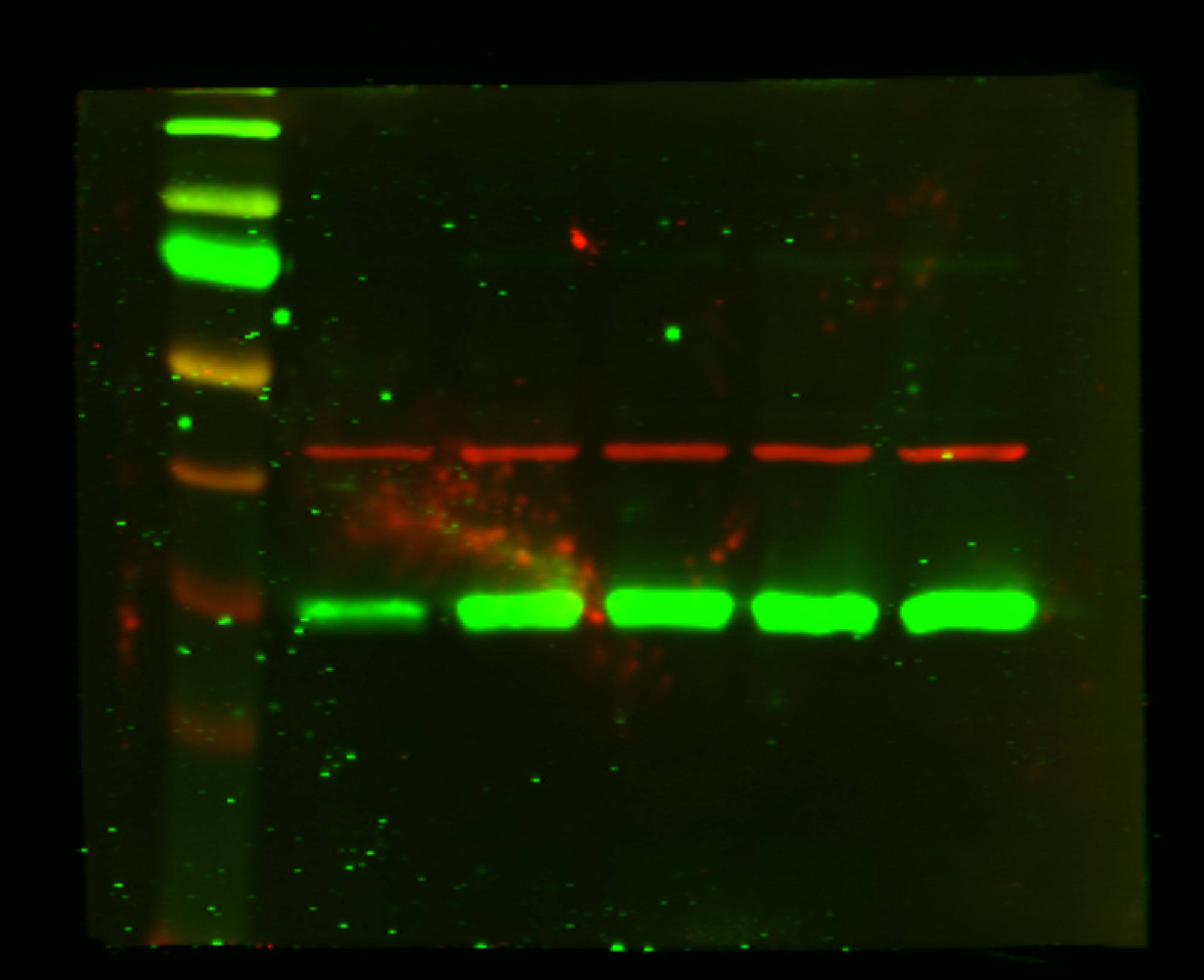

Supplement: Figure 3—figure supplement 8—source data 1. [file elife-82184-fig3-figsupp8-data1.zip › Figure 3-figure supplement 8-source data/A/2/2_KRasG13CacetyledaGDP_pS6.tif]

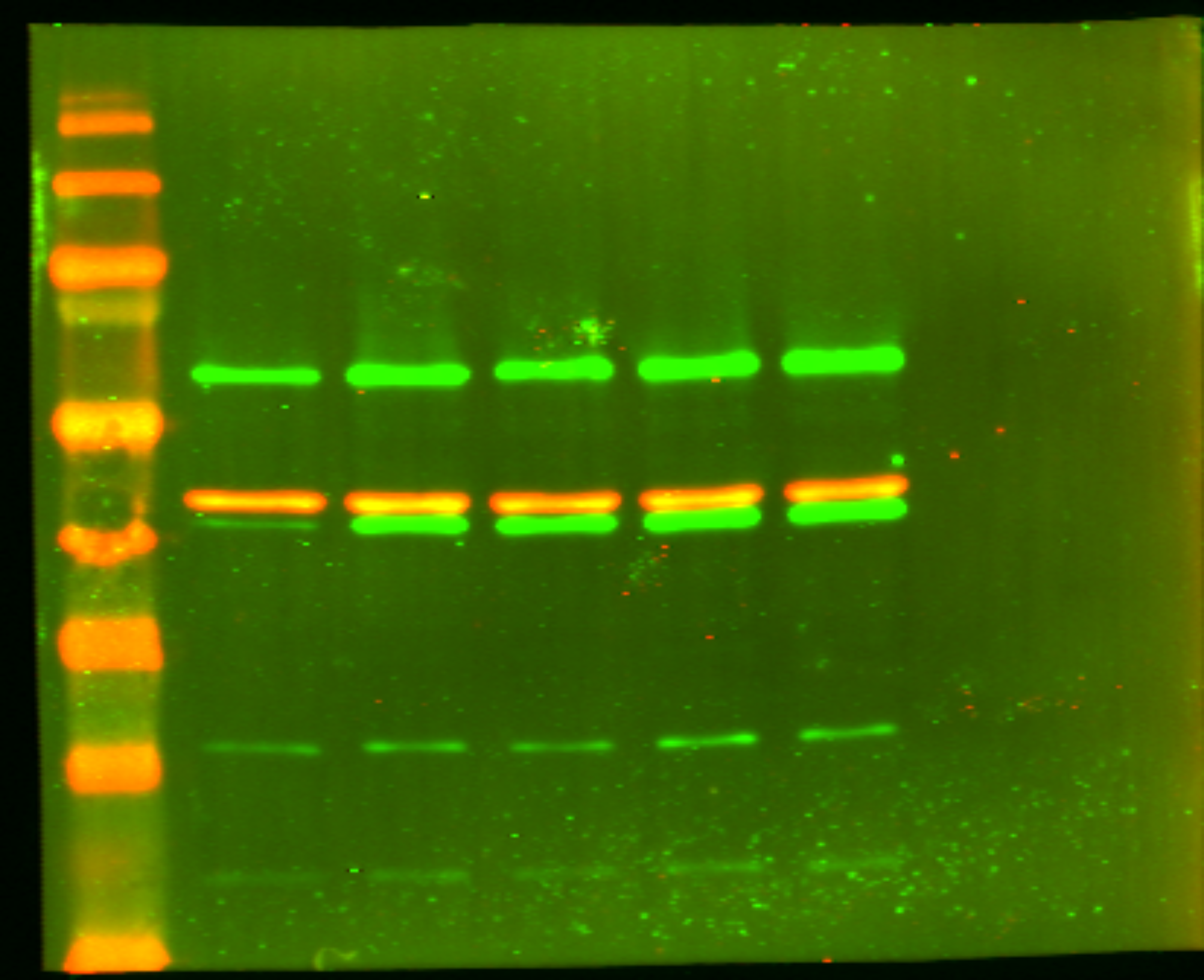

Supplement: Figure 3—figure supplement 8—source data 1. [file elife-82184-fig3-figsupp8-data1.zip › Figure 3-figure supplement 8-source data/A/2/2_KRasG13CacetyledaGDP_tAkt.tif]

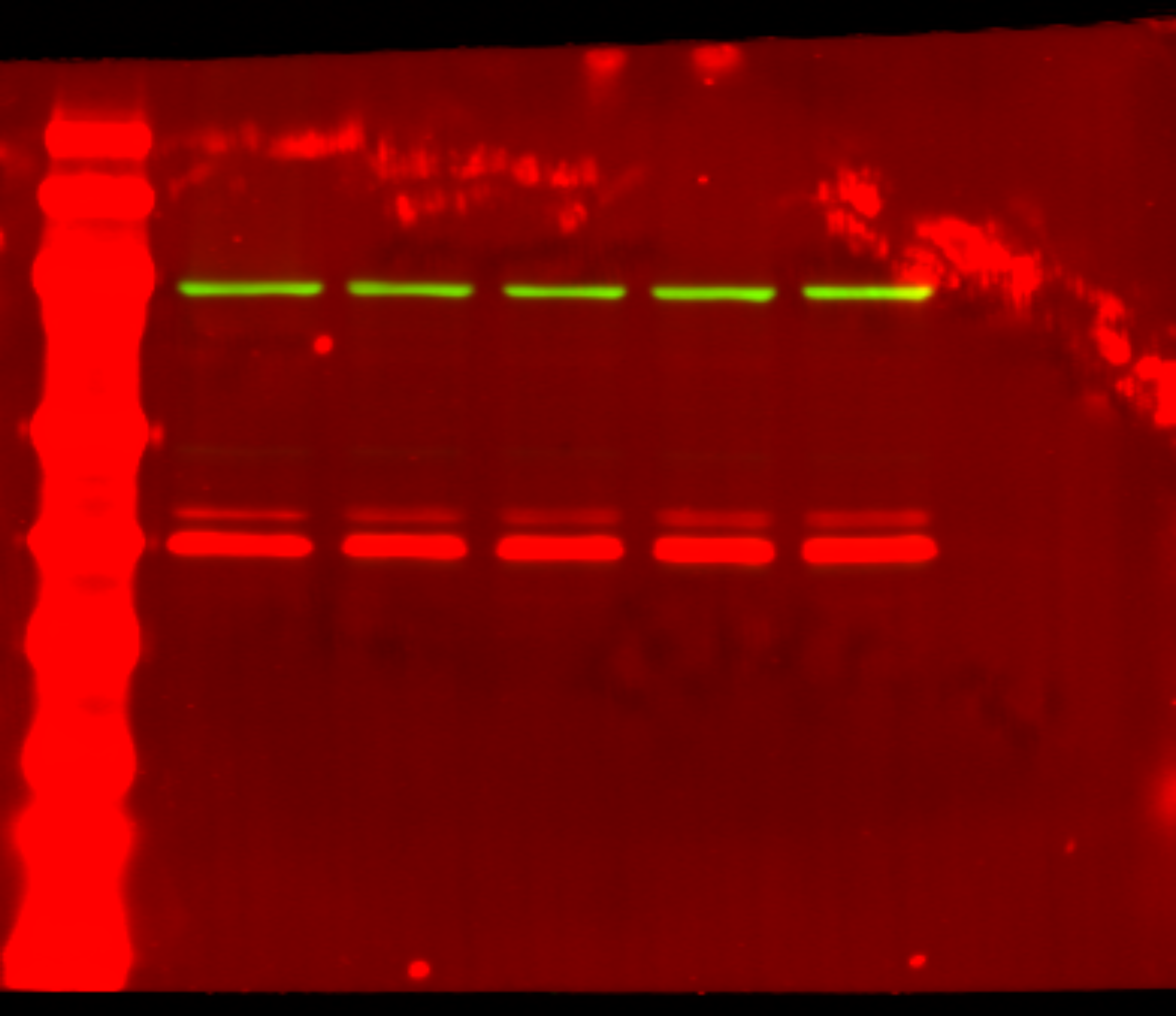

Supplement: Figure 3—figure supplement 8—source data 1. [file elife-82184-fig3-figsupp8-data1.zip › Figure 3-figure supplement 8-source data/A/2/2_KRasG13CacetyledaGDP_tErk.tif]

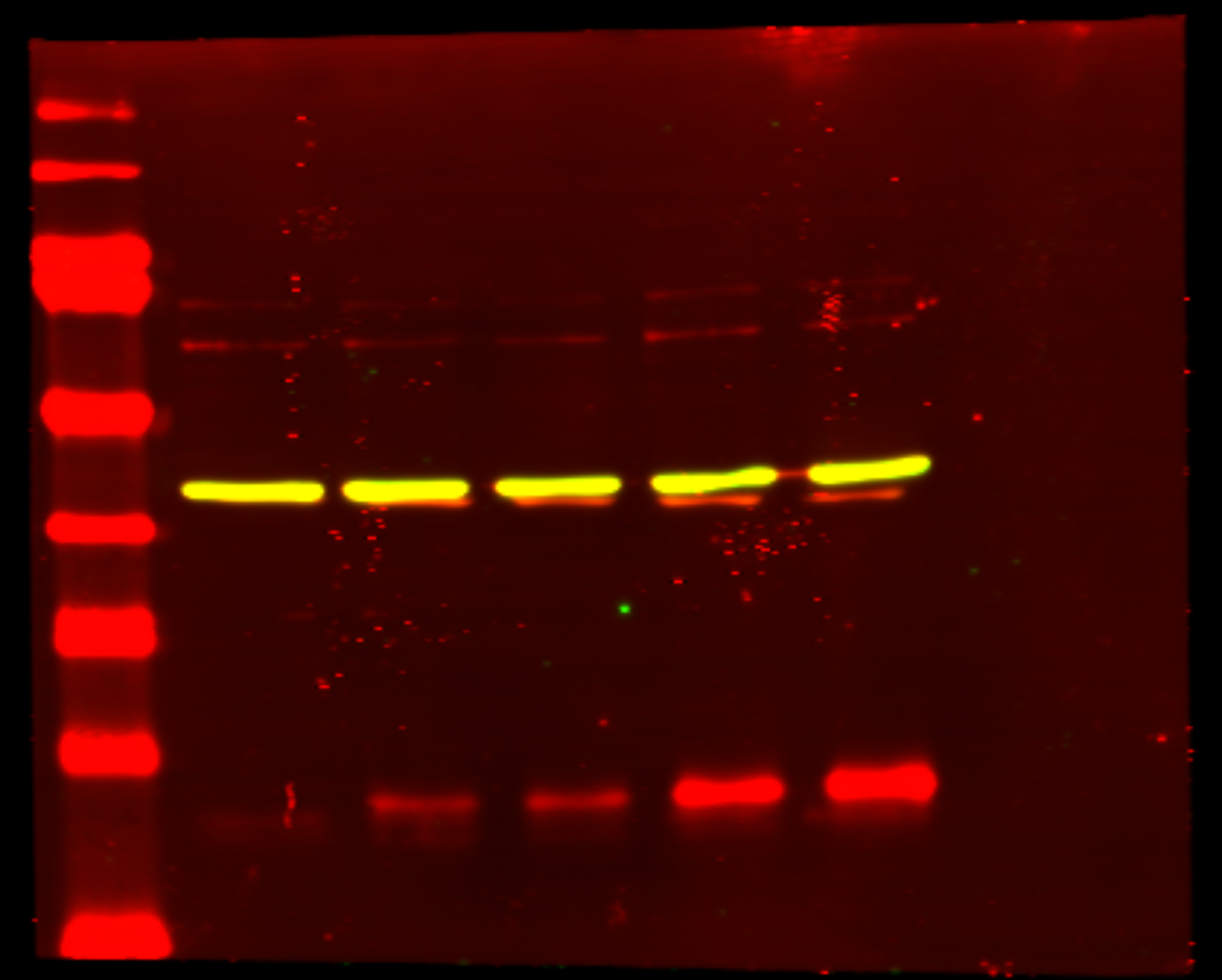

Supplement: Figure 3—figure supplement 8—source data 1. [file elife-82184-fig3-figsupp8-data1.zip › Figure 3-figure supplement 8-source data/A/3/3_KRasG13CacetyledaGDP_KRas.tif]

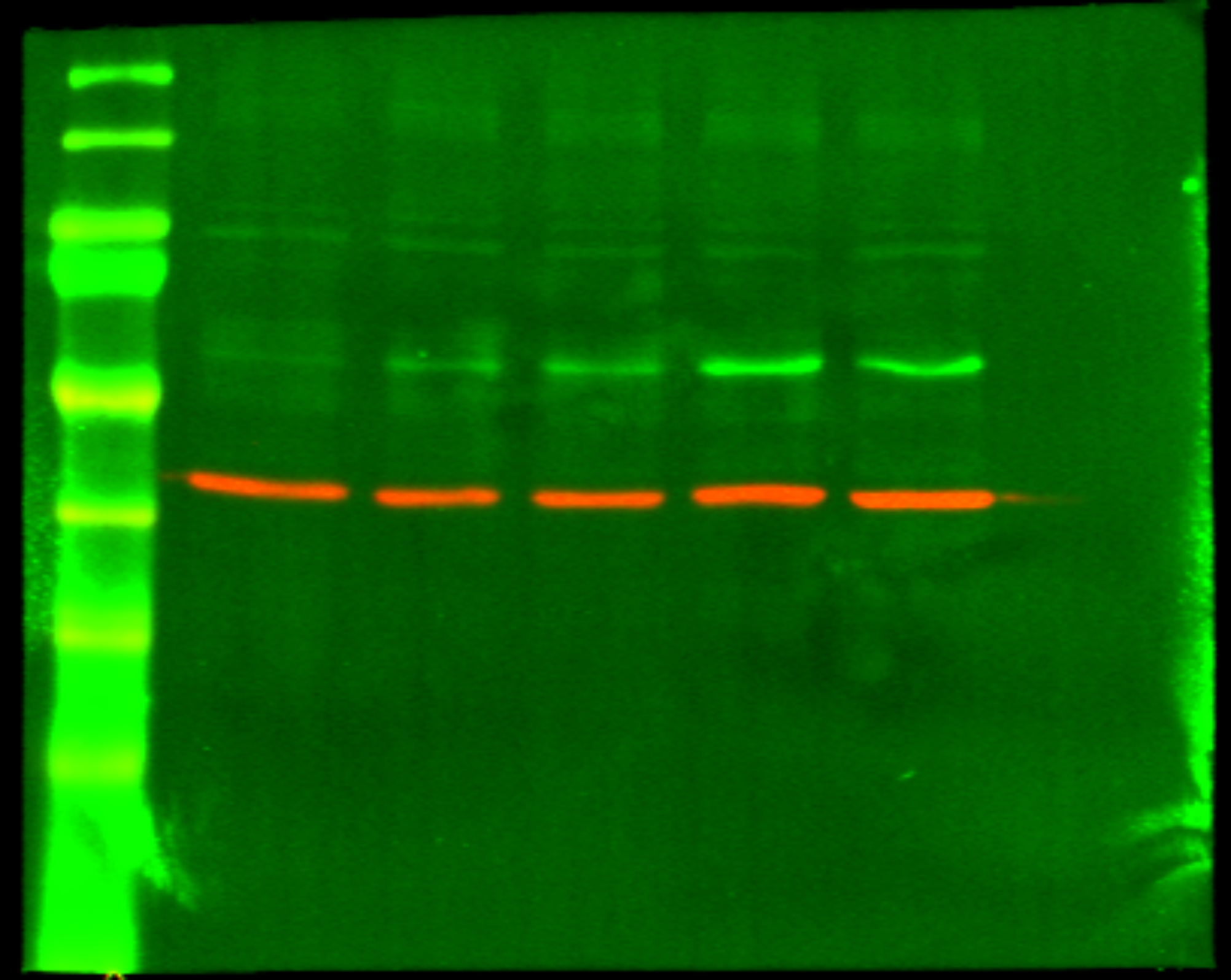

Supplement: Figure 3—figure supplement 8—source data 1. [file elife-82184-fig3-figsupp8-data1.zip › Figure 3-figure supplement 8-source data/A/3/3_KRasG13CacetyledaGDP_pAkt.tif]

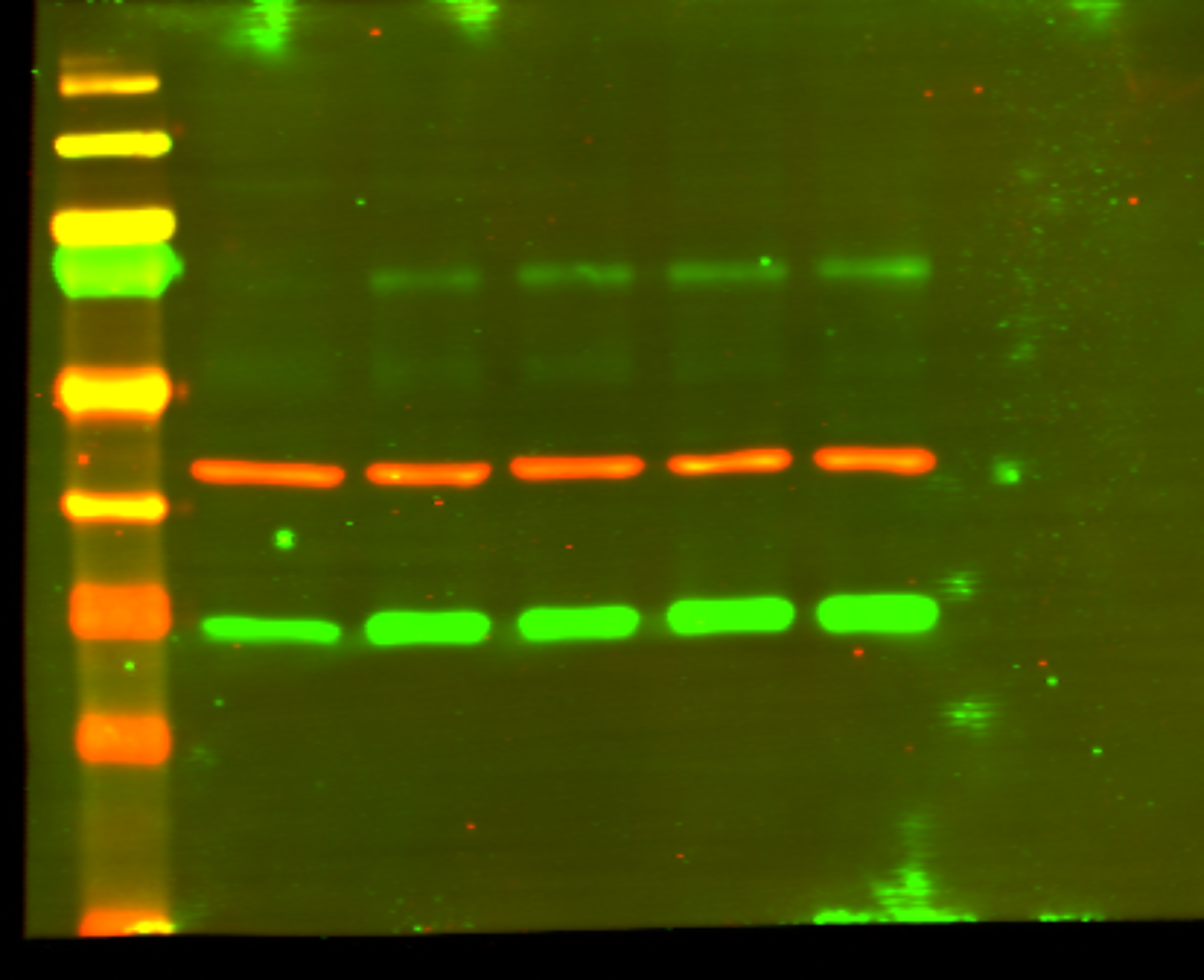

Supplement: Figure 3—figure supplement 8—source data 1. [file elife-82184-fig3-figsupp8-data1.zip › Figure 3-figure supplement 8-source data/A/3/3_KRasG13CacetyledaGDP_pcRaf_S6.tif]

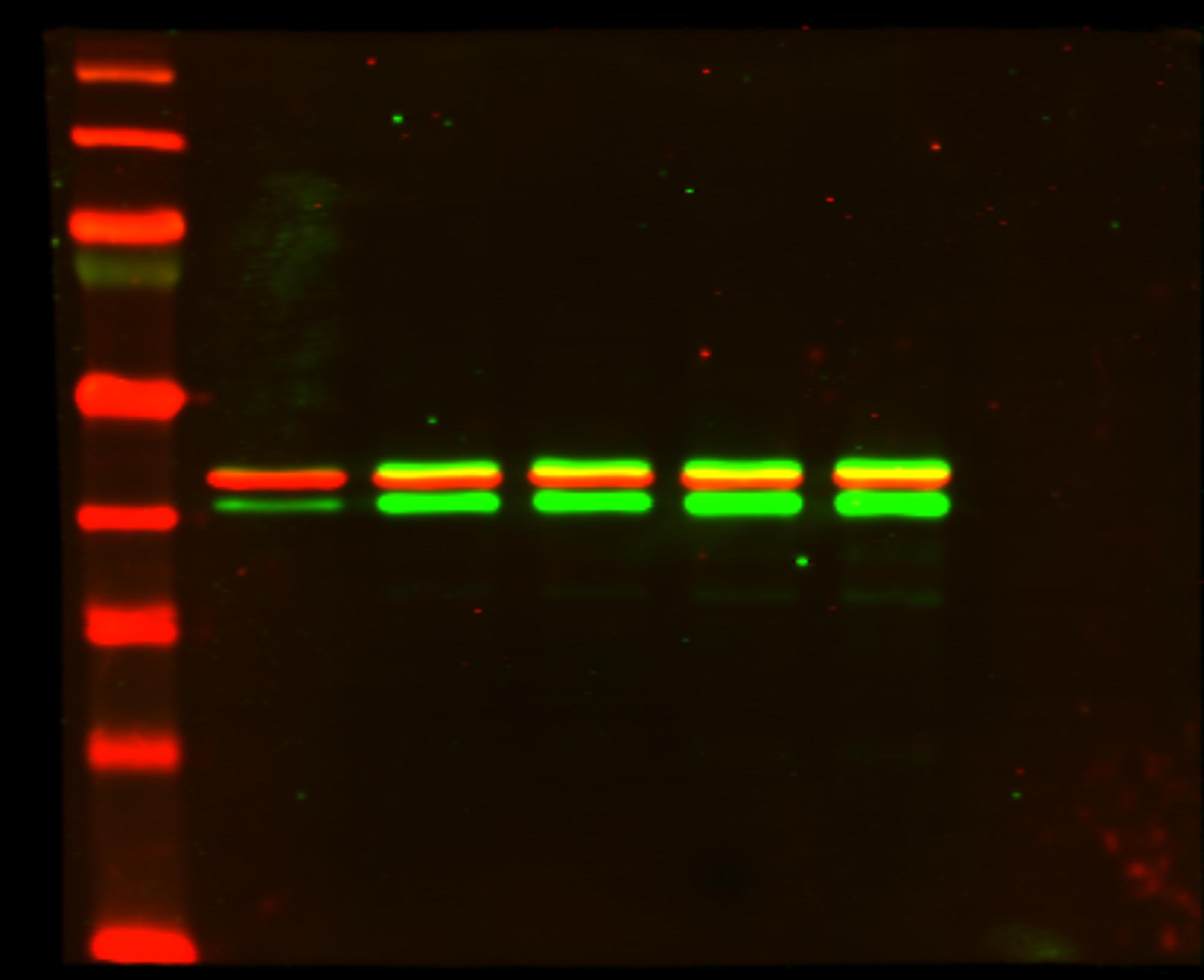

Supplement: Figure 3—figure supplement 8—source data 1. [file elife-82184-fig3-figsupp8-data1.zip › Figure 3-figure supplement 8-source data/A/3/3_KRasG13CacetyledaGDP_pErk.tif]

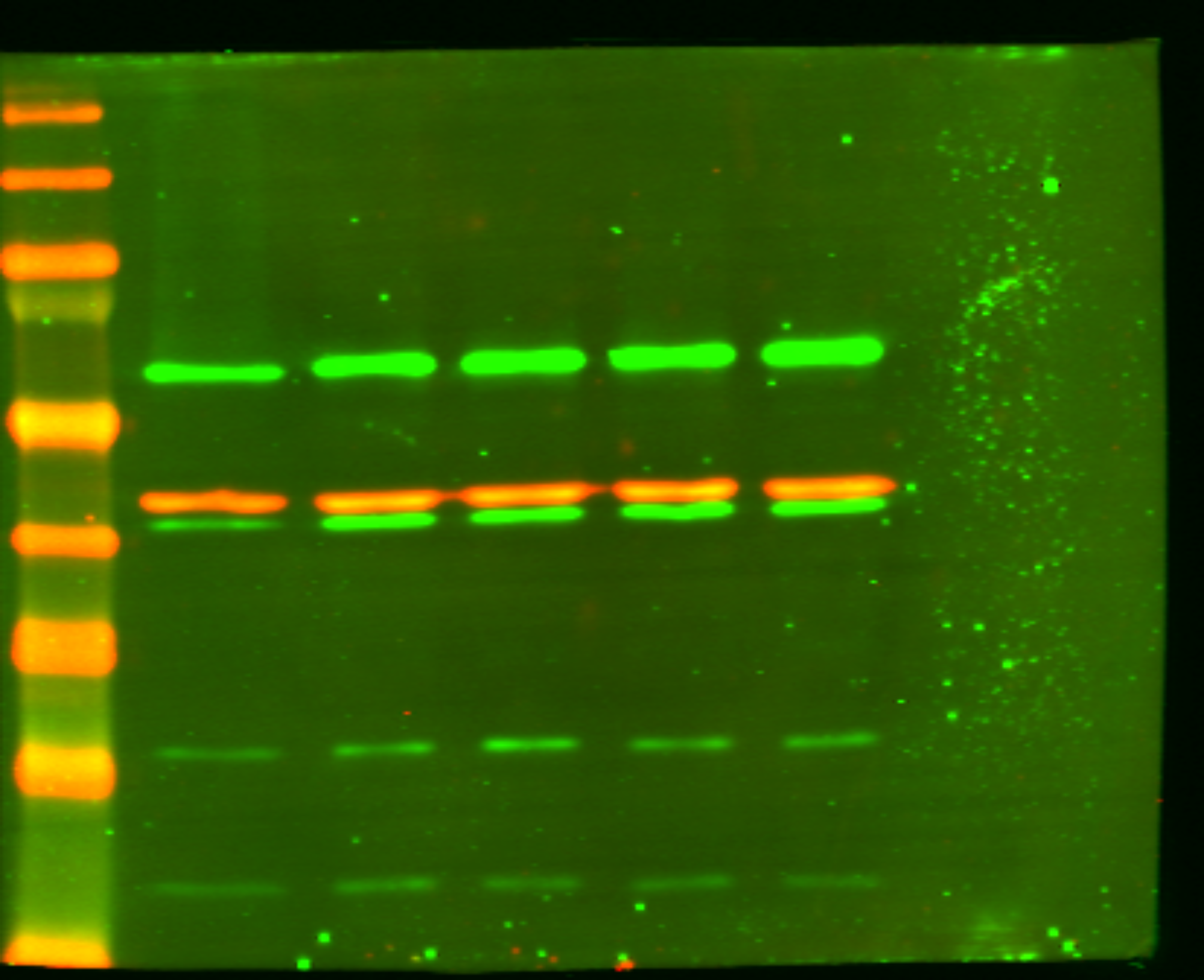

Supplement: Figure 3—figure supplement 8—source data 1. [file elife-82184-fig3-figsupp8-data1.zip › Figure 3-figure supplement 8-source data/A/3/3_KRasG13CacetyledaGDP_tAkt.tif]

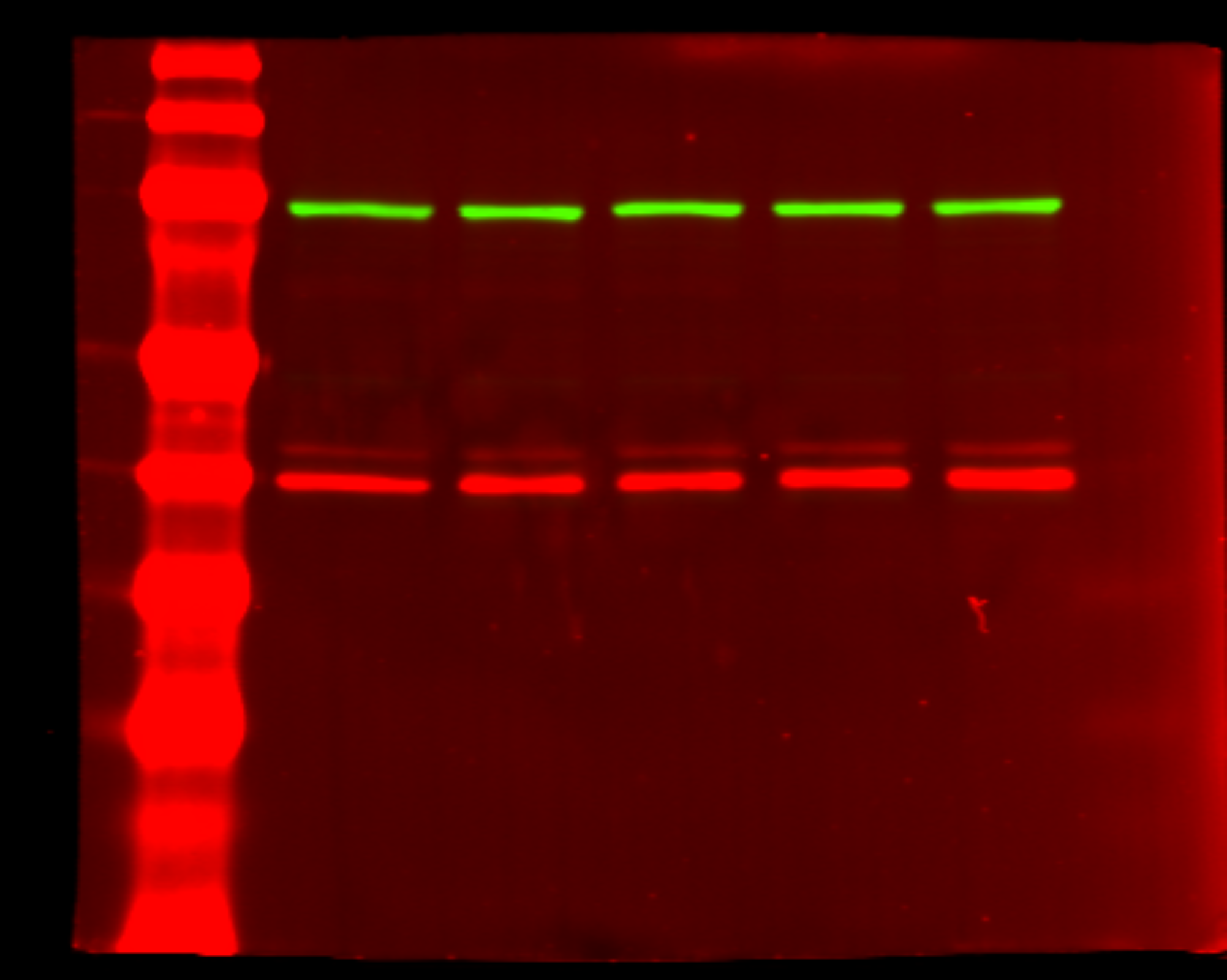

Supplement: Figure 3—figure supplement 8—source data 1. [file elife-82184-fig3-figsupp8-data1.zip › Figure 3-figure supplement 8-source data/A/3/3_KRasG13CacetyledaGDP_tErk.tif]

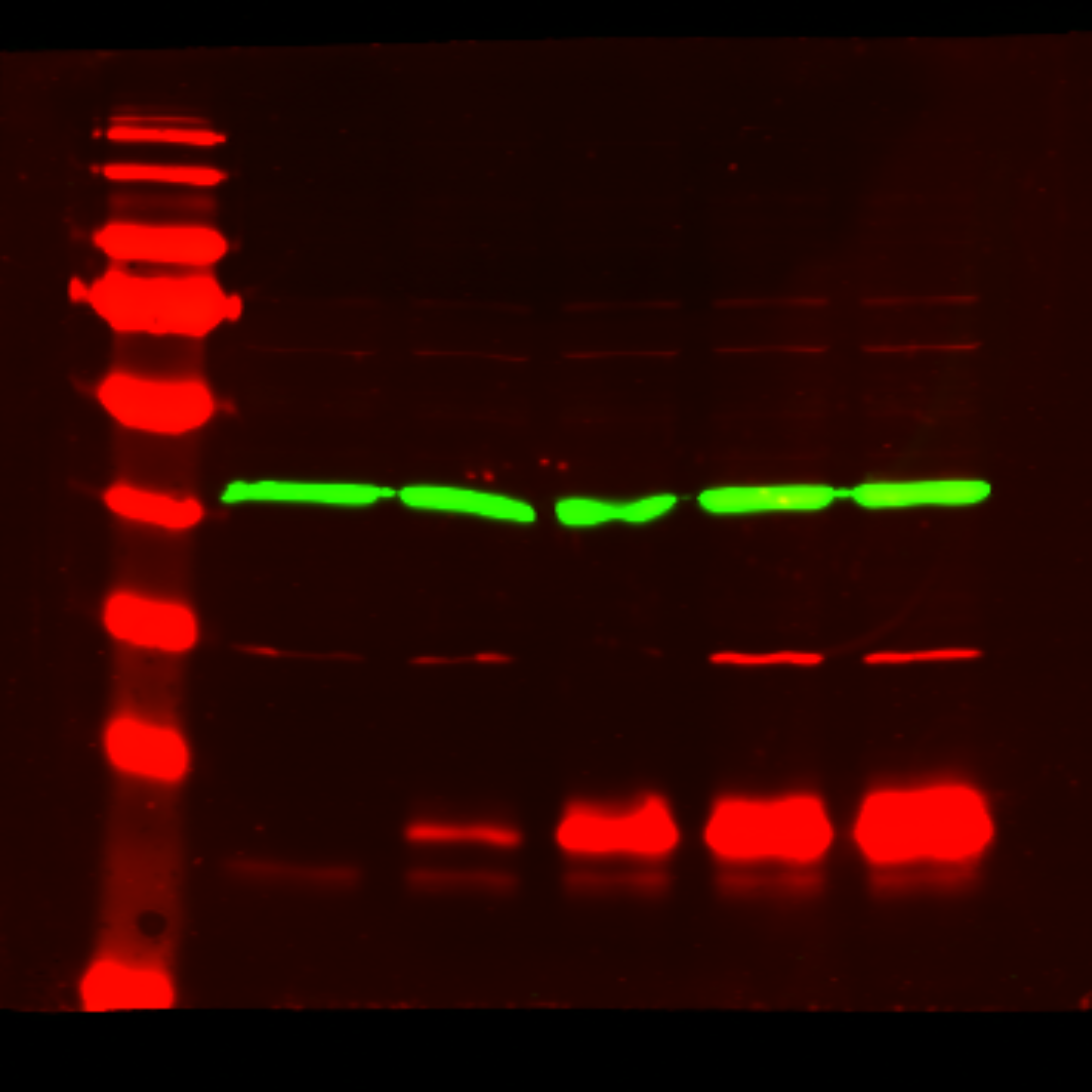

Supplement: Figure 3—figure supplement 9—source data 1. [file elife-82184-fig3-figsupp9-data1.zip › Figure 3-figure supplement 9-source data/A/1/1_KRasG13C-edaGppCp_KRas.tif]

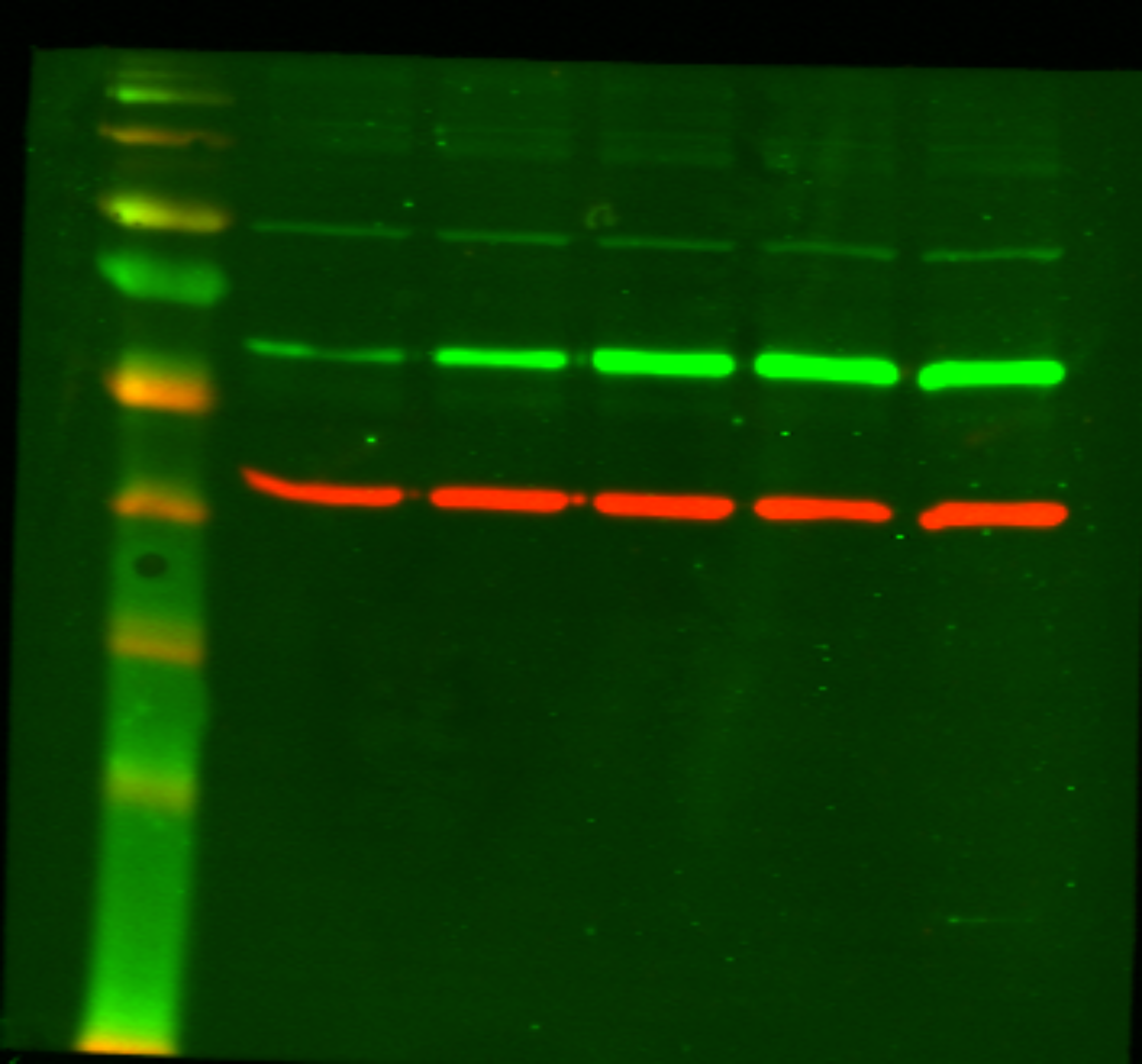

Supplement: Figure 3—figure supplement 9—source data 1. [file elife-82184-fig3-figsupp9-data1.zip › Figure 3-figure supplement 9-source data/A/1/1_KRasG13C-edaGppCp_pAkt.tif]

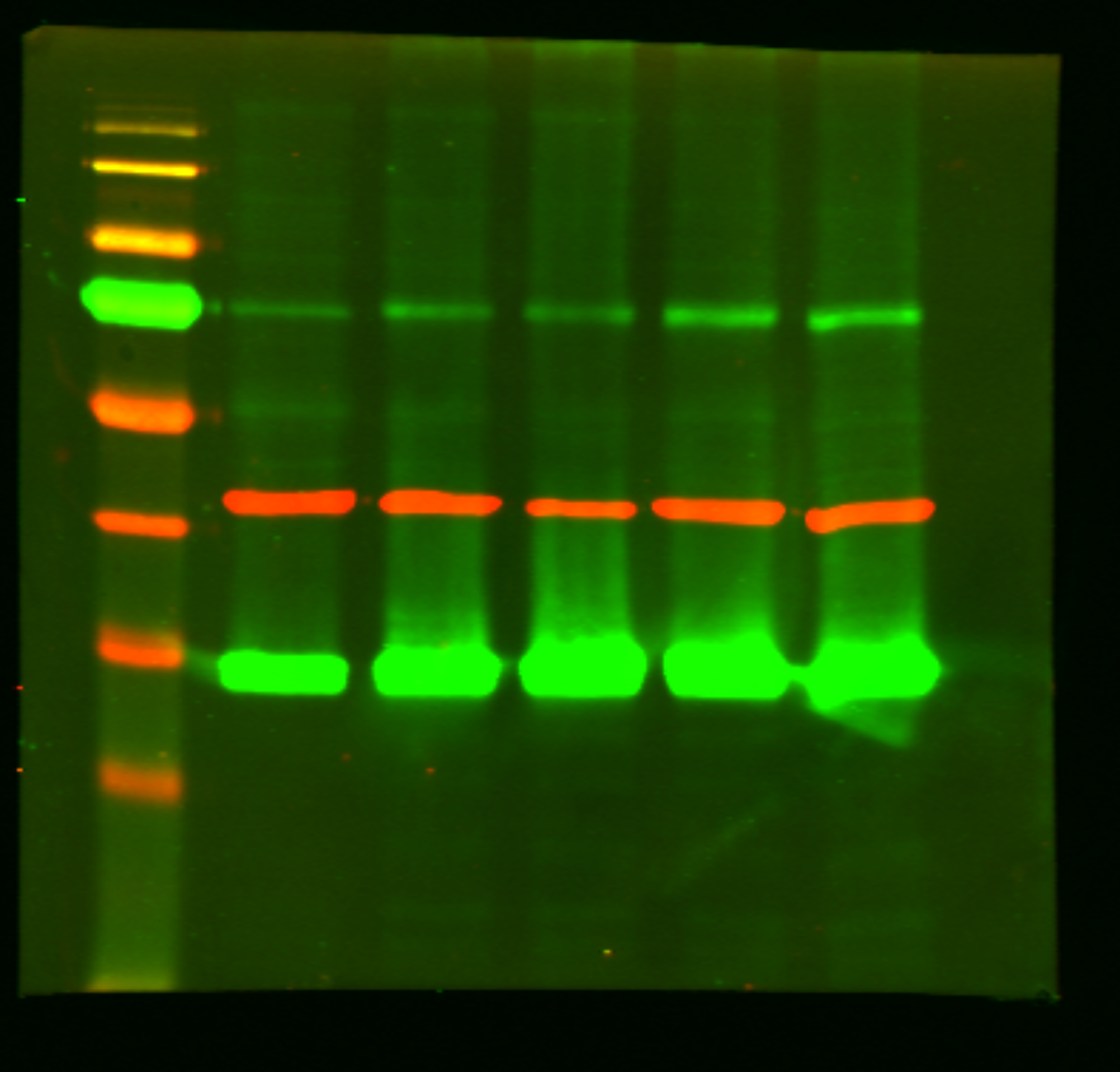

Supplement: Figure 3—figure supplement 9—source data 1. [file elife-82184-fig3-figsupp9-data1.zip › Figure 3-figure supplement 9-source data/A/1/1_KRasG13C-edaGppCp_pcRaf.tif]

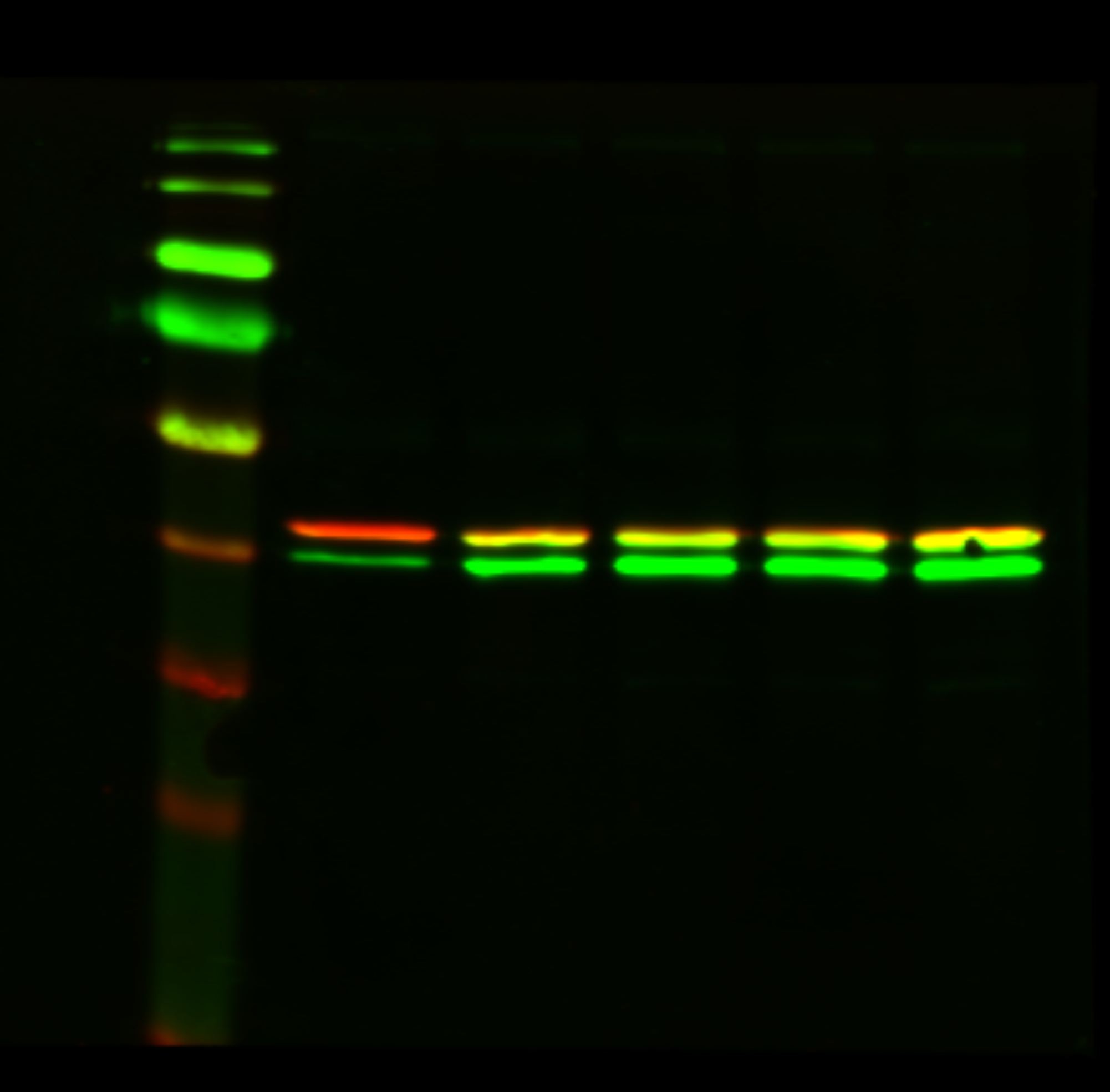

Supplement: Figure 3—figure supplement 9—source data 1. [file elife-82184-fig3-figsupp9-data1.zip › Figure 3-figure supplement 9-source data/A/1/1_KRasG13C-edaGppCp_pErk.tif]

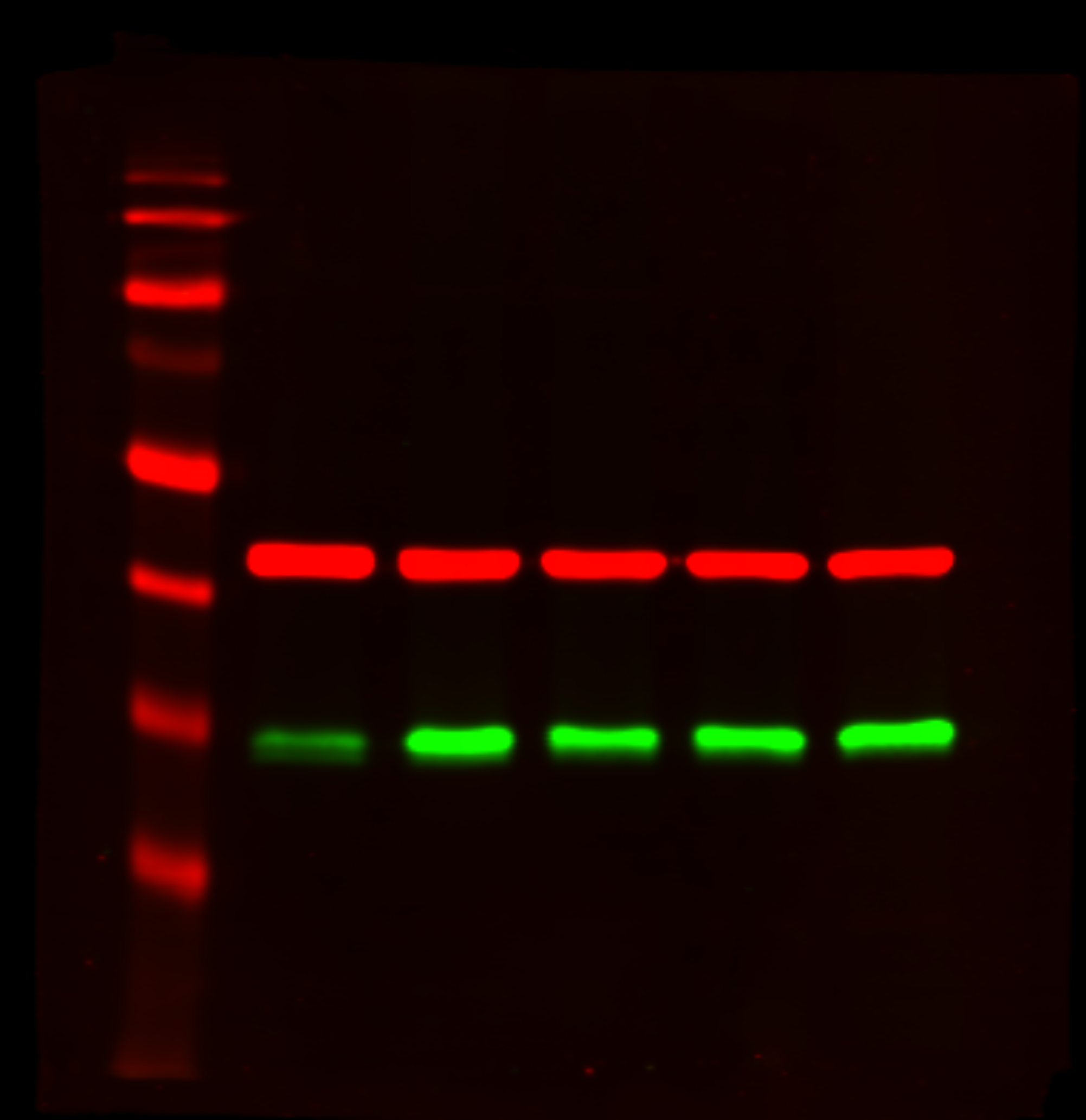

Supplement: Figure 3—figure supplement 9—source data 1. [file elife-82184-fig3-figsupp9-data1.zip › Figure 3-figure supplement 9-source data/A/1/1_KRasG13C-edaGppCp_pS6.tif]

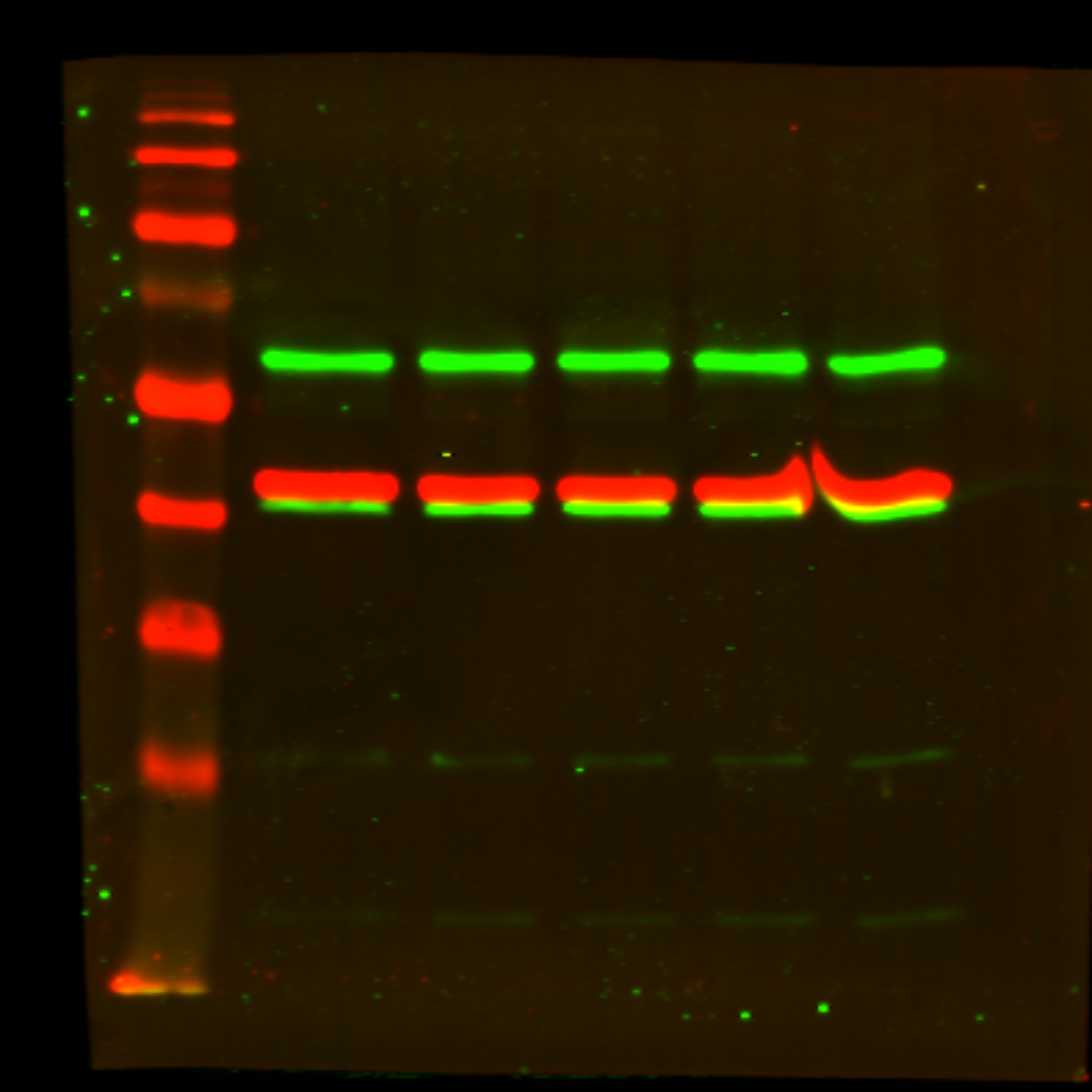

Supplement: Figure 3—figure supplement 9—source data 1. [file elife-82184-fig3-figsupp9-data1.zip › Figure 3-figure supplement 9-source data/A/1/1_KRasG13C-edaGppCp_tAkt.tif]

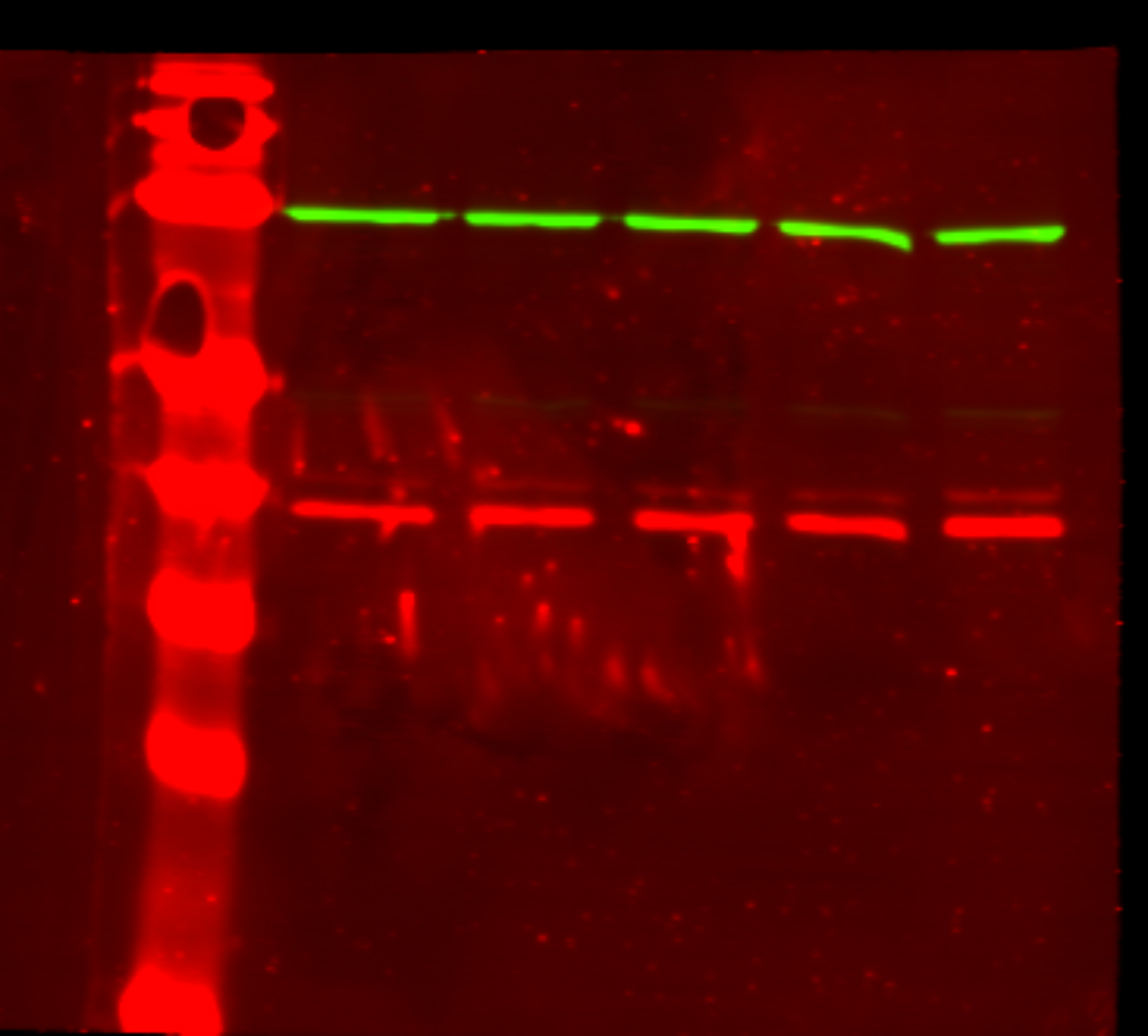

Supplement: Figure 3—figure supplement 9—source data 1. [file elife-82184-fig3-figsupp9-data1.zip › Figure 3-figure supplement 9-source data/A/1/1_KRasG13C-edaGppCp_tErk.tif]

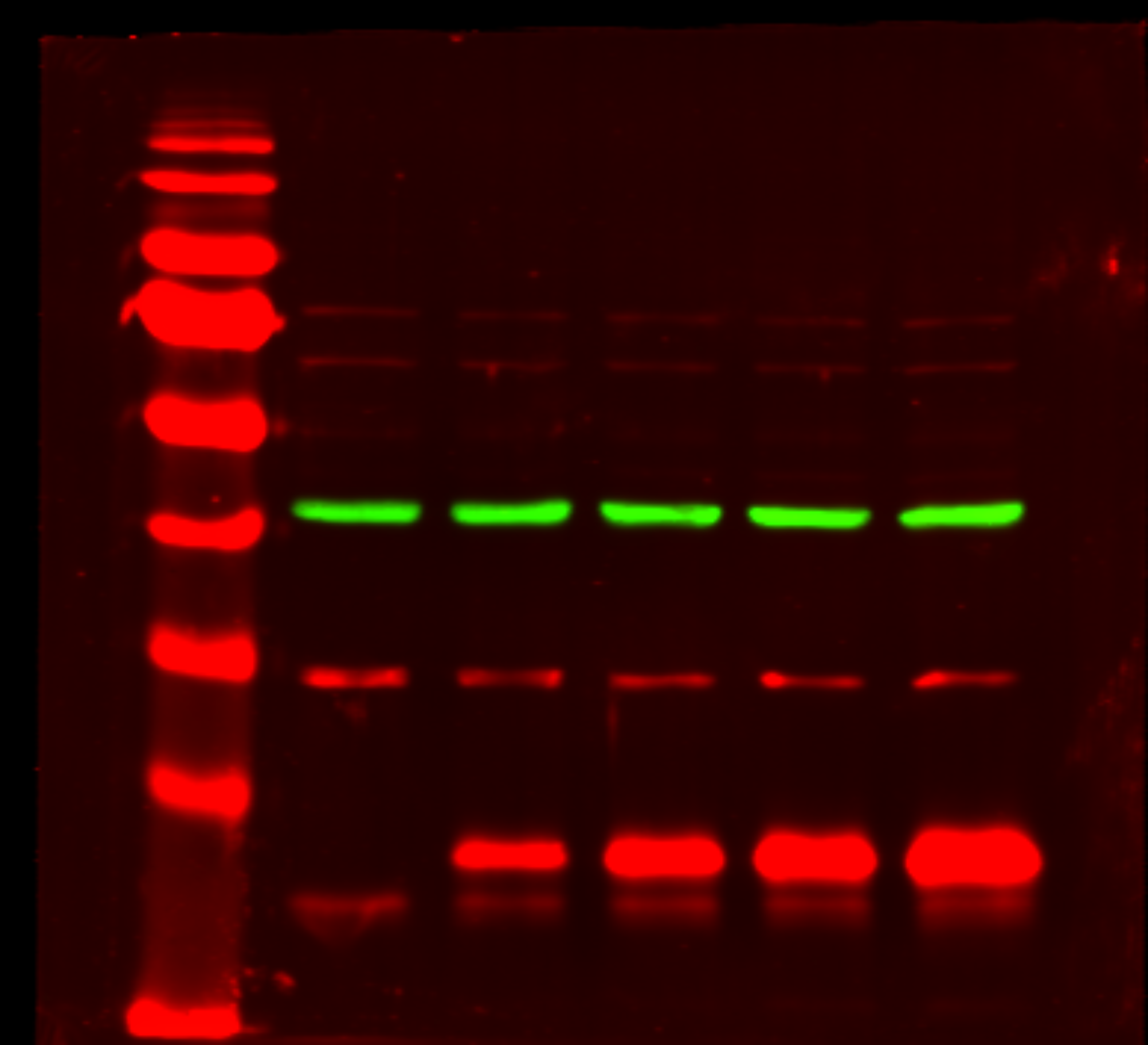

Supplement: Figure 3—figure supplement 9—source data 1. [file elife-82184-fig3-figsupp9-data1.zip › Figure 3-figure supplement 9-source data/A/2/2_KRasG13C-edaGppCp_KRas.tif]

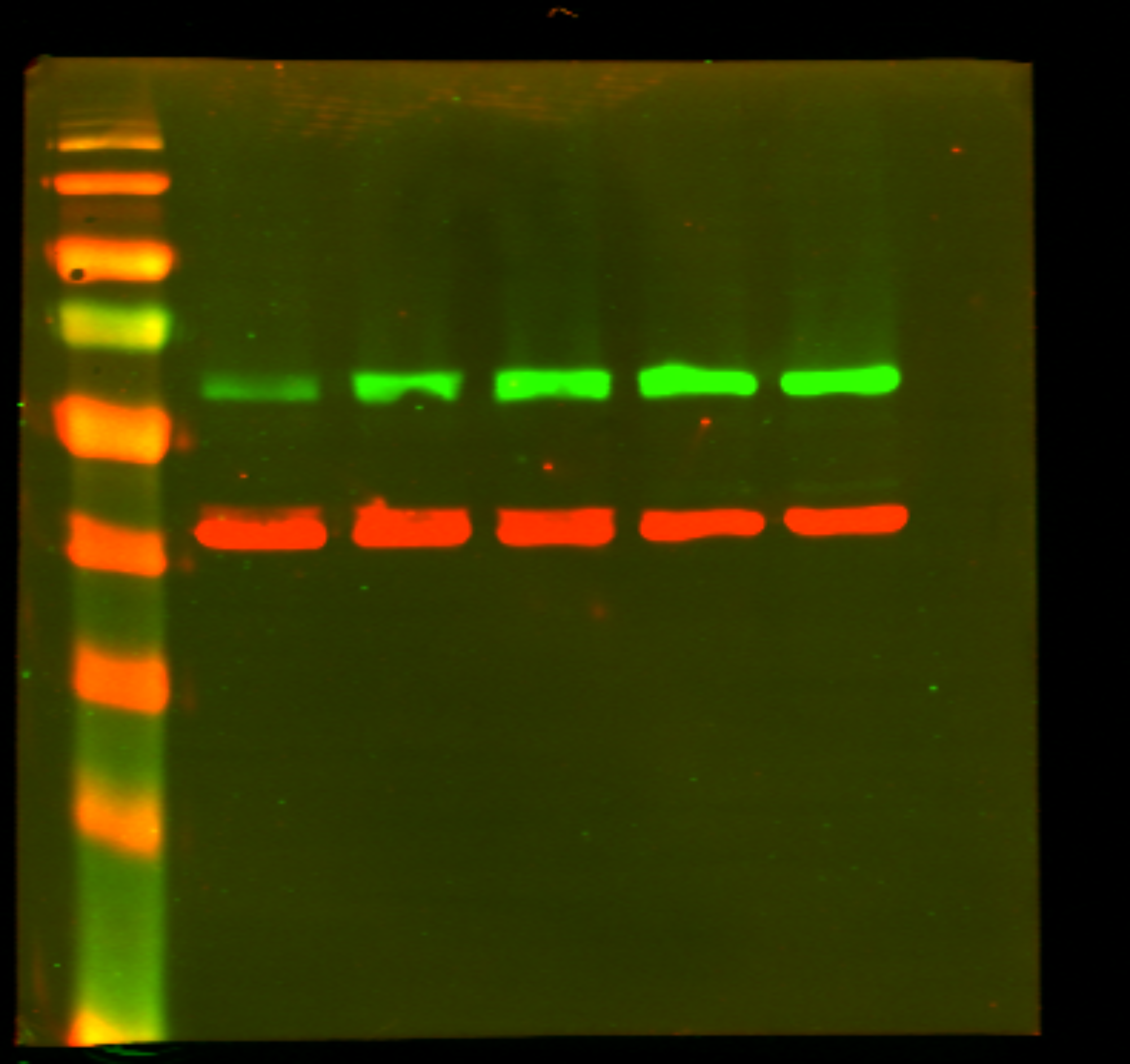

Supplement: Figure 3—figure supplement 9—source data 1. [file elife-82184-fig3-figsupp9-data1.zip › Figure 3-figure supplement 9-source data/A/2/2_KRasG13C-edaGppCp_pAkt.tif]

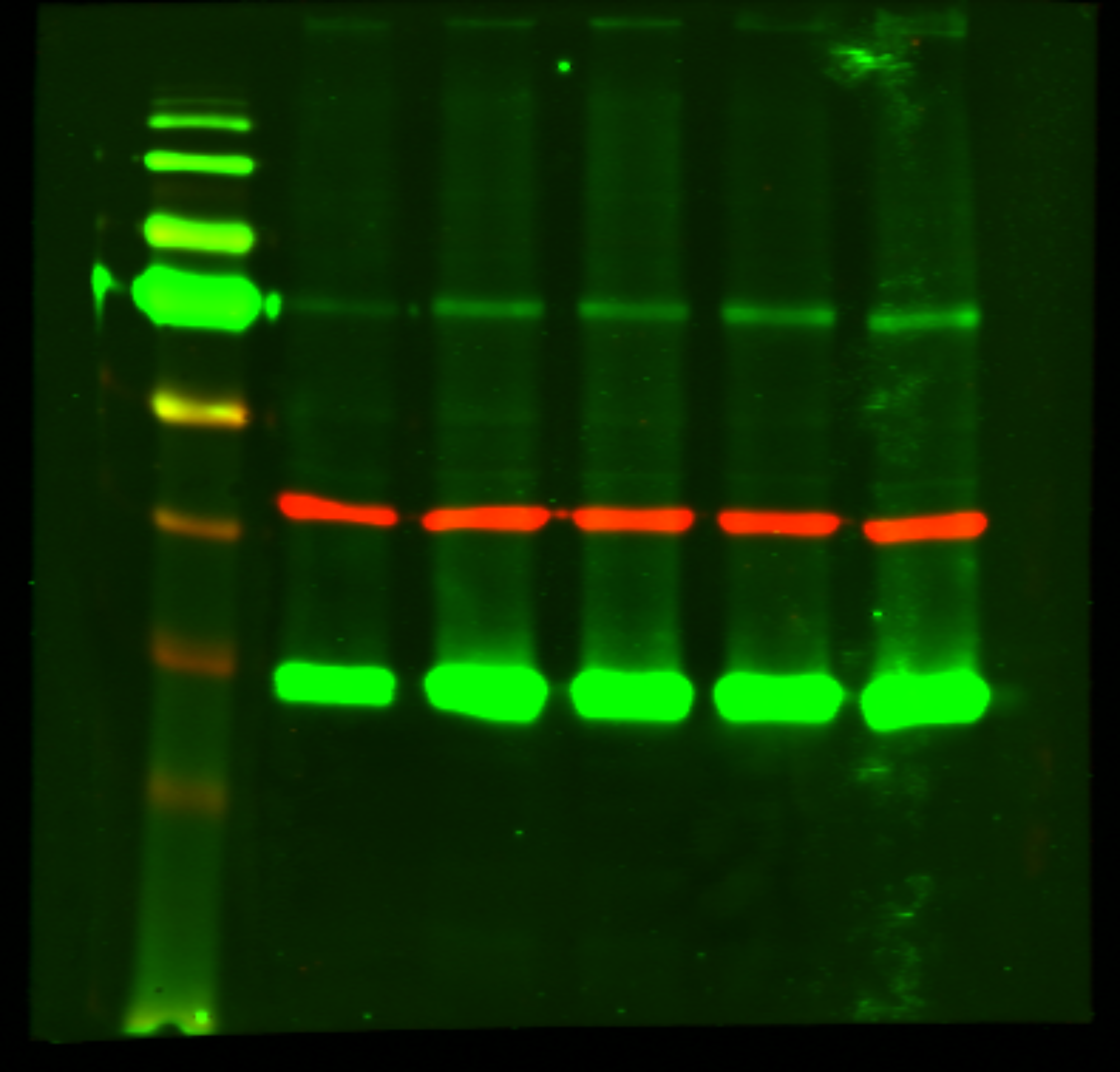

Supplement: Figure 3—figure supplement 9—source data 1. [file elife-82184-fig3-figsupp9-data1.zip › Figure 3-figure supplement 9-source data/A/2/2_KRasG13C-edaGppCp_pcRaf_pS6.tif]

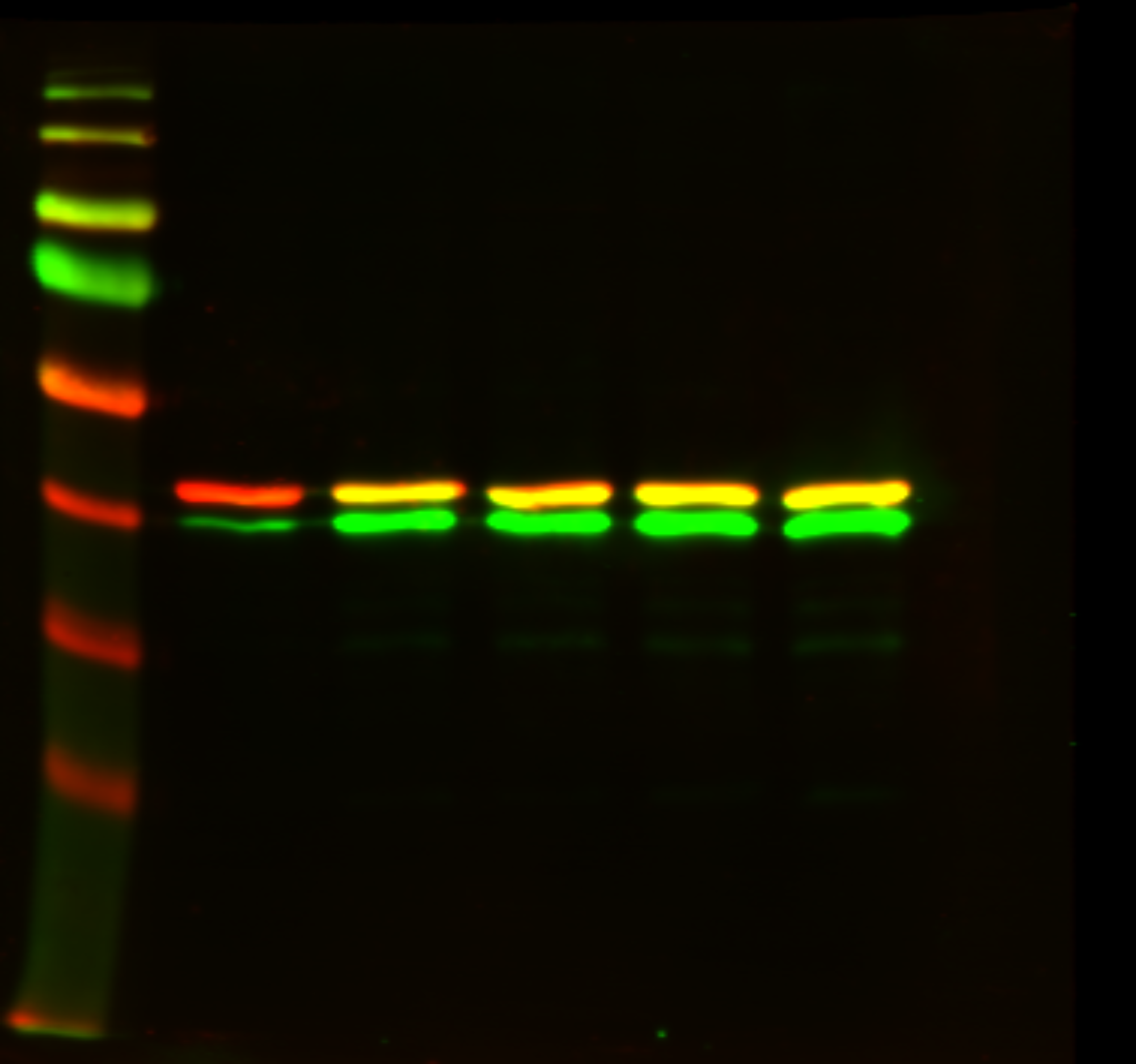

Supplement: Figure 3—figure supplement 9—source data 1. [file elife-82184-fig3-figsupp9-data1.zip › Figure 3-figure supplement 9-source data/A/2/2_KRasG13C-edaGppCp_pErk.tif]

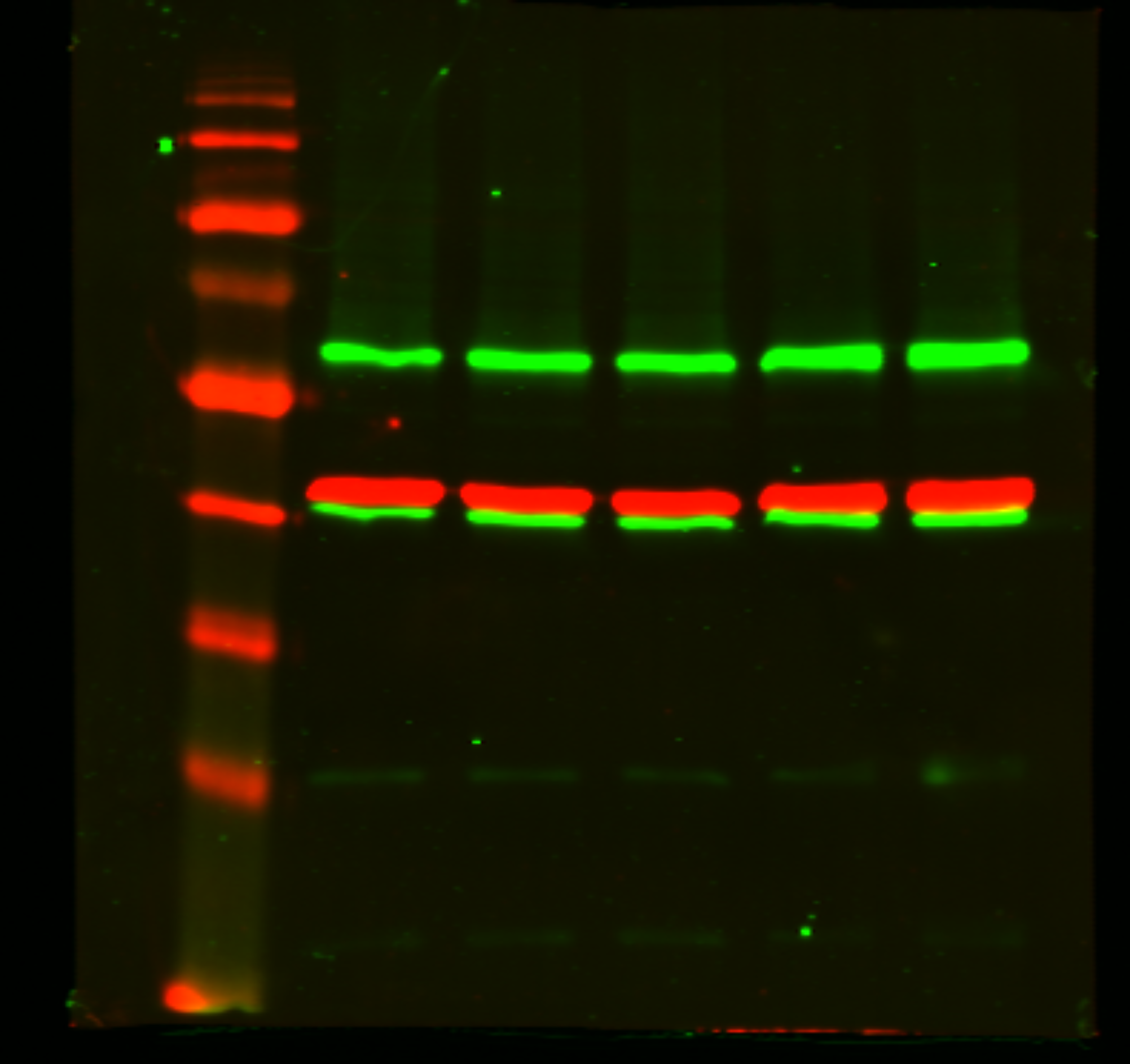

Supplement: Figure 3—figure supplement 9—source data 1. [file elife-82184-fig3-figsupp9-data1.zip › Figure 3-figure supplement 9-source data/A/2/2_KRasG13C-edaGppCp_tAkt.tif]

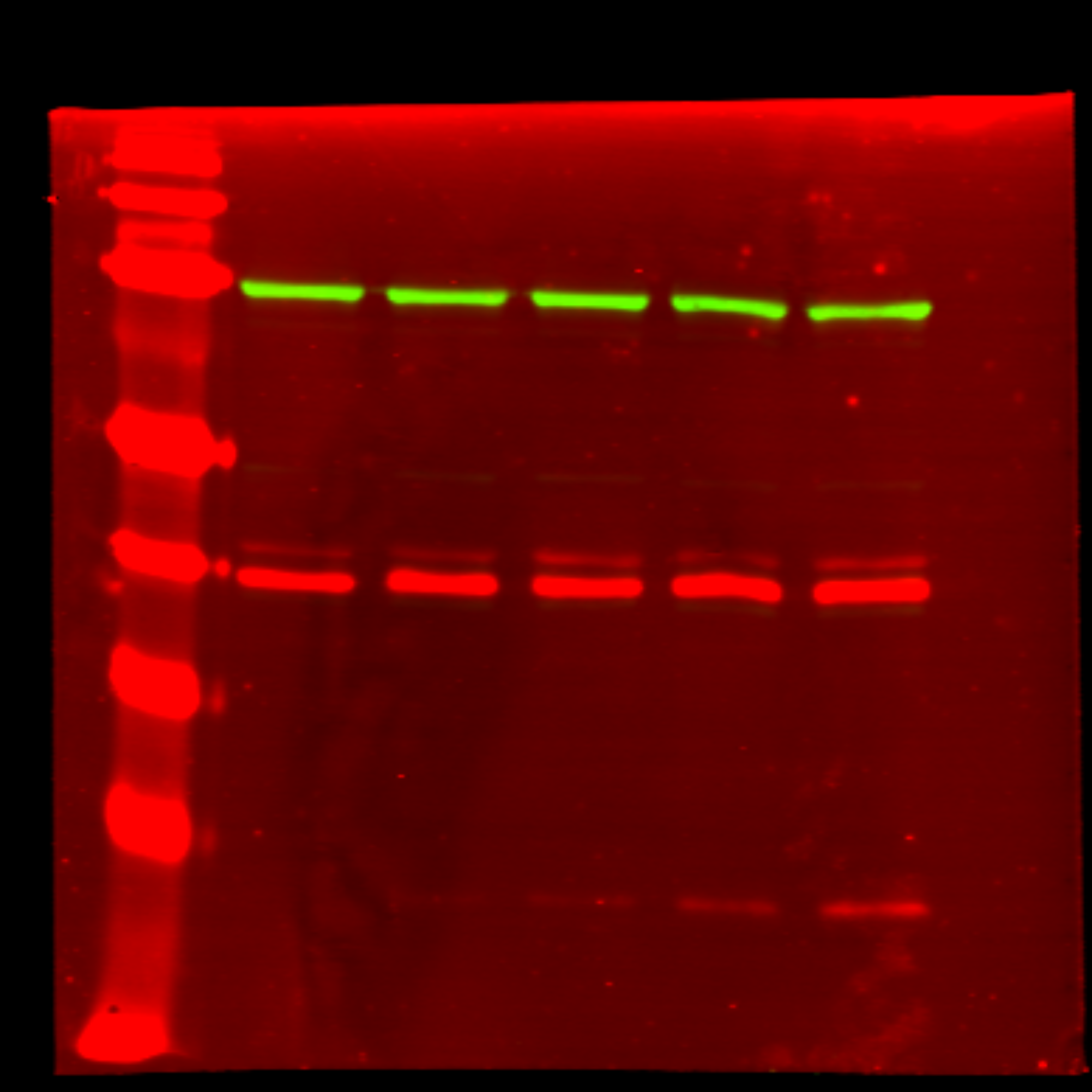

Supplement: Figure 3—figure supplement 9—source data 1. [file elife-82184-fig3-figsupp9-data1.zip › Figure 3-figure supplement 9-source data/A/2/2_KRasG13C-edaGppCp_tErk.tif]
