# Supplementary material for: Targeting oncogenic KRasG13C with nucleotide-based covalent inhibitors
Source: eLife. 2023 Mar 27;12:e82184. doi: 10.7554/eLife.82184 (PMC10042540; doi:10.7554/eLife.82184)
Supplement: Table 1—source data 2. — Overview of kon rate constants obtained from competitive binding experiments with mantdGDP (Table 1—source data 1). [file elife-82184-table1-data2.zip › Table 1-source data 2.docx]

**Table 1-source data 2:** k_on_ calculation. Overview of k_on_ rate constants obtained from competitive binding experiments with mantdGDP (Table 1-source data 1).

|  | **nucleotide** | | **eda** | **pda** | **bda** |
| --- | --- | --- | --- | --- | --- |
| **k_on_**  **[µM^-1^s^-1^]** | **GDP** | 4.22 | 3.73 | 3.34 | 3.12 |
|  | **GTP** | 5.23 | 4.39 | 3.36 | 4.21 |
|  | **dGTP** | - | 4.51 | 4.38 | 4.06 |
