# Supplementary material for: Targeting oncogenic KRasG13C with nucleotide-based covalent inhibitors
Source: eLife. 2023 Mar 27;12:e82184. doi: 10.7554/eLife.82184 (PMC10042540; doi:10.7554/eLife.82184)
Supplement: Table 1—source data 4. — Overview of the calculated KD values of pdaGDP and edaGDP obtained from an HPLC-based approach (Table 1—source data 3); For reference, the KD values of GDP and the nucleotide analogue SML-8-73-1 are also listed. [file elife-82184-table1-data4.zip › Table 1-source data 4.docx]

**Table 1-source data 4.** K_D_ calculations. Overview of the calculated K_D_ values of pdaGDP and edaGDP obtained from an HPLC-based approach (Table 1-source data 3); For reference, the K_D_ values of GDP and the nucleotide analogue SML-8-73-1 are also listed.

|  | **nucleotide** | **-EDTA** | **+EDTA** | **+SOS** |
| --- | --- | --- | --- | --- |
| **K_D_**  **[pM]** | **GDP** | 2.5 | | |
|  | **pdaGDP** | 8.0 | 7.4 | 10.4 |
|  | **bdaGDP** | 10.0 | 8.9 | 10.0 |
|  | **SML-8-73-1** | ~ 140 nM | | |
