# Supplementary material for: Targeting oncogenic KRasG13C with nucleotide-based covalent inhibitors
Source: eLife. 2023 Mar 27;12:e82184. doi: 10.7554/eLife.82184 (PMC10042540; doi:10.7554/eLife.82184)
Supplement: Figure 2—source data 1. [file elife-82184-fig2-data1.docx]

**Figure 2-source data 1:** Data collection and refinement statistics for KRasG13C-edaGDP and KRasG13C-bdaGDP.

| **Data Collection** | **KRasG13C-edaGDP**  **(PDB 7ok3)** | **KRasG13C-bdaGDP**  **(PDB 7ok4)** |
| --- | --- | --- |
| Space group | P 63 | P 63 |
| Cell constants  a, b, c (Å)  α, β, γ (°) | 73.40, 73.40, 54.20  90.00, 90.00, 120.00 | 73.90, 73.90, 54.80  90.00, 90.00, 120.00 |
| Resolution (Å) | 41.243 – 1.6 (1.7-1.6) | 41.625 – 1.7 (1.8-1.7) |
| R_meas_ (%) | 10.3 (101.6) | 7.1 (159.6) |
| R_merge_ (%) | 9.8 (96.6) | 7.0 (155.7) |
| I/σ | 12.71 (2.40) | 24.52 (2.26) |
| CC_1/2_ | 99.9 (80.1) | 100.0 (78.4) |
| Completeness (%) | 100.0 (100.0) | 100.0 (100.0) |
| Redundancy | 10.1 (10.4) | 20.3 (20.5) |
| **Refinement** |  |  |
| Resolution (Å) | 41.243 – 1.6 | 41.625 – 1.7 |
| No. Reflections | 22009 | 18838 |
| R_work_ / R_free_ | 15.80/20.01 (23.30/30.62) | 16.53/18.24 (26.43/35.58) |
| No. Atoms |  |  |
| Protein | 1386 | 1336 |
| Ligand/Ion | 38 | 40 |
| Water | 187 | 106 |
| B-factors |  |  |
| Protein | 27.02 | 36.92 |
| Ligand/Ion | 18.91 | 30.77 |
| Water | 36.23 | 45.00 |
| R.m.s deviations |  |  |
| Bond lengths (Å) | 0.016 | 0.004 |
| Bond angles (°) | 1.483 | 0.711 |
| Wavelength (Å) | 0.91504 | 0.999 |
| Temperature (K) | 100 | 100 |
| X-ray source | X10SA at SLS (Villigen, CH) | X10SA at SLS (Villigen, CH) |
| Detector | Pilatus 6M | EIGER2 X 16M |
| **Ramachandran Plot** |  |  |
| Outliers (%) | 0.00 | 0.00 |
| Allowed (%) | 2.38 | 2.98 |
| Favored (%) | 97.62 | 97.02 |
