# Supplementary material for: Targeting oncogenic KRasG13C with nucleotide-based covalent inhibitors
Source: eLife. 2023 Mar 27;12:e82184. doi: 10.7554/eLife.82184 (PMC10042540; doi:10.7554/eLife.82184)
Supplement: Supplementary file 1. — Multiple sequence alignment of the G domain Ras superfamily members using Uniprot (the uniport accession codes can be taken from this figure). Based on the marked areas that are shown in Figure 1—figure supplement 1, cysteines that are within the reach of the warhead of the eda linker are highlighted in gray. In contrast, regions that are also within reach of the bda linker are highlighted in blue. The cysteines located within these regions are marked in red. Only ~7% of the GTPases have cysteines within the P-loop and are therefore potential off-targets of the designed nucleotide derivatives. [file elife-82184-supp1.docx]

ARF1 P84077 ------------------------------------MGNIFAN---------LFKGLFGKKEMRILMVGLDAAGKTTILYKLKLG--------------------EI-VTTIPTIG---- 50

ARF3 P61204 ------------------------------------MGNIFGN---------LLKSLIGKKEMRILMVGLDAAGKTTILYKLKLG--------------------EI-VTTIPTIG---- 50

ARF4 P18085 ------------------------------------MGLTISS---------LFSRLFGKKQMRILMVGLDAAGKTTILYKLKLG--------------------EI-VTTIPTIG---- 50

ARF5 P84085 ------------------------------------MGLTVSA---------LFSRIFGKKQMRILMVGLDAAGKTTILYKLKLG--------------------EI-VTTIPTIG---- 50

ARF6 P62330 ----------------------------------------MGK---------VLSKIFGNKEMRILMLGLDAAGKTTILYKLKLG--------------------QS-VTTIPTVG---- 46

ARL1 P40616 ------------------------------------MGGFFSS---------IFSSLFGTREMRILILGLDGAGKTTILYRLQVG--------------------EV-VTTIPTIG---- 50

ARL2 P36404 ------------------------------------MGLLTIL---------KKMKQ-KERELRLLMLGLDNAGKTTILKKFNGE--------------------DI-DTISPTLG---- 49

ARL3 P36405 ------------------------------------MGLLSIL---------RKLKSAPDQEVRILLLGLDNAGKTTLLKQLASE--------------------DI-SHITPTQG---- 50

ARL4A P40617 ------------------------------------MGNGLSD----Q-TS-ILSNLPSFQSFHIVILGLD**C**AGKTTVLYRLQFN--------------------EF-VNTVPTKG---- 53

ARL4C P56559 -----------------------------------------------M-GN-ISSNISAFQSLHIVMLGLDSAGKTTVLYRLKFN--------------------EF-VNTVPTIG---- 46

ARL4D P49703 ------------------------------------MGNHLTE----M-APTASSFLPHFQALHVVVIGLDSAGKTSLLYRLKFK--------------------EF-VQSVPTKG---- 54

ARL5A Q9Y689 ------------------------------------MGILFTR----------IWRLFNHQEHKVIIVGLDNAGKTTILYQFSMN--------------------EV-VHTSPTIG---- 49

ARL5B Q96KC2 ------------------------------------MGLIFAK----------LWSLFCNQEHKVIIVGLDNAGKTTILYQFLMN--------------------EV-VHTSPTIG---- 49

ARL5C A6NH57 ------------------------------------MGQLIAK----------LMSIFGNQEHTVIIVGLDNEGKTTILYRFLTN--------------------EV-VHM**C**PTIG---- 49

ARL6 Q9H0F7 ------------------------------------MGLLDRL---------SVLLGLKKKEVHVLCLGLDNSGKTTIINKLKPS----------------N--AQS-QNILPTIG---- 52

ARL8A Q96BM9 ------------------------------------MIALFNK----L--LDWFKALFWKEEMELTLVGLQYSGKTTFVNVIASG--------------------QFNEDMIPTVG---- 54

ARL8B Q9NVJ2 ------------------------------------MLALISR----L--LDWFRSLFWKEEMELTLVGLQYSGKTTFVNVIASG--------------------QFSEDMIPTVG---- 54

ARL9 Q6T311 ------------------------------------MRPTWK--------ALSHPAWPEEKNKQILVLGLDGAGKTSVLHSLASN--------------------RVQHSVAPTQG---- 52

ARL10 Q8N8L6 ---------------D---RGEAWWGA------EAARLPEWDE-WDPEDEEDEEPALEELEQREVLVLGLDGAGKSTFLRVLSGK--------------------PPLEGHIPTWG---- 111

ARL11 Q969Q4 ------------------------------------MGSV--------------NSRGHKAEAQVVMMGLDSAGKTTLLYKLKGH--------------------QL-VETLPTVG---- 45

ARL13A Q5H913 ------------------------------------MFRLLSSCCSCL-----RTTEETRRNVTIPIIGLNNSGKTVLVEAFQKL--------------------LP-SKTDHCMK---- 54

ARL13B Q3SXY8 ------------------------------------MFSLMASCCGWF-----KRWREPVRKVTLLMVGLDNAGKTATAKGIQGE--------------------YP-EDVAPTVG---- 54

ARL14 Q8N4G2 ------------------------------------MGSLG-------------SKNPQTKQAQVLLLGLDSAGKSTLLYKLKLA--------------------KD-ITTIPTIG---- 46

ARL15 Q9NXU5 --------------------------I--------TEAFLYMD-YLCFRALCCKGPPPARPEYDLVCIGLTGSGKTSLLSKLCSE--------------------SP-DNVVSTTG---- 65

ARL16 Q0P5N6 --------------------------V--------AGGRALSR------GA-ELRVPGGAKHGMCLLLGATGVGKTLLVKRLQEV--SS--R------DGKGDLGEP-PPTRPTVG---- 66

ARL17 Q8IVW1 ------------------------------------MGNIFEK---------LFKSLLGKKKMRILILSLDTAGKTTILYKLKLG--------------------ET-VPAVPTVG---- 50

ARFRP1 Q13795 ------------------------------------MYTLLSG---------LYKYMFQKDEYCILILGLDNAGKTTFLEQSKTR--FN--K------NYKG--MSL-SKITTTVG---- 58

SAR1A Q9NR31 ------------------------------------MSFIFEWIYNGF-SSVLQFLGLYKKSGKLVFLGLDNAGKTTLLHMLKDD--------------------RL-GQHVPTLH---- 58

SAR1B Q9Y6B6 ------------------------------------MSFIFDWIYSGF-SSVLQFLGLYKKTGKLVFLGLDNAGKTTLLHMLKDD--------------------RL-GQHVPTLH---- 58

TRIM23 P36406 --------------------------L------DASIPVTFTK--------DNRVHIGPKMEIRVVTLGLDGAGKTTILFKLKQD--------------------EF-MQPIPTIG---- 437

IFT27 Q9BW83 ---------------------------------------------------------MVKLAAKCILAGDPAVGKTALAQIFRSDGAHF--Q-K----SY-----------TLTTG--MD 43

RAB1A P62820 ---------------------------------------------------MSSMNPEYDYLFKLLLIGDSGVGKS**C**LLLRFADD--TY--T-E----SY-----------ISTIG--VD 47

RAB1B Q9H0U4 ------------------------------------------------------MNPEYDYLFKLLLIGDSGVGKS**C**LLLRFADD--TY--T-E----SY-----------ISTIG--VD 44

RAB1C Q92928 ------------------------------------------------------MNPGYDCLFKLLLIGDSGVGKS**C**LLLRFADD--PY--T-E----SY-----------ISTIG--VD 44

RAB2A P61019 --------------------------------------------------------MAYAYLFKYIIIGDTGVGKS**C**LLLQFTDK--RF--Q-P----VH-----------DLTIG--VE 42

RAB2B Q8WUD1 --------------------------------------------------------MTYAYLFKYIIIGDTGVGKS**C**LLLQFTDK--RF--Q-P----VH-----------DLTIG--VE 42

RAB3A P20336 ------------------------------AS-------AT----DSRYGQKESSDQNFDYMFKILIIGNSSVGKTSFLFRYADD--SF--T-P----AF-----------VSTVG--ID 58

RAB3B P20337 ------------------------------AS-------VT----DGKTGVKDASDQNFDYMFKLLIIGNSSVGKTSFLFRYADD--TF--T-P----AF-----------VSTVG--ID 58

RAB3C Q96E17 ------------------------------AS-------AQ----DARYGQKDSSDQNFDYMFKLLIIGNSSVGKTSFLFRYADD--SF--T-S----AF-----------VSTVG--ID 66

RAB3D O95716 ------------------------------AS-------AG----DTQAGPRDAADQNFDYMFKLLLIGNSSVGKTSFLFRYADD--SF--T-P----AF-----------VSTVG--ID 58

RAB4A P20338 -------------------------------------------------MSQTAMSETYDFLFKFLVIGNAGTGKS**C**LLHQFIEK--KF--K-D----DS-----------NHTIG--VE 49

RAB4B P61018 ------------------------------------------------------MAETYDFLFKFLVIGSAGTGKS**C**LLHQFIEN--KF--K-Q----DS-----------NHTIG--VE 44

RAB5A P20339 -------------------------------------MASR----GA-TRPNGPNTGNKICQFKLVLLGESAVGKSSLVLRFVKG--QF--H-E----FQ-----------ESTIG--AA 56

RAB5B P61020 -------------------------------------MTSR----ST-ARPNGQPQASKICQFKLVLLGESAVGKSSLVLRFVKG--QF--H-E----YQ-----------ESTIG--AA 56

RAB5C P51148 ------------------------------------MAGRG----GA-ARPNGPAAGNKICQFKLVLLGESAVGKSSLVLRFVKG--QF--H-E----YQ-----------ESTIG--AA 57

RAB6A P20340 ------------------------------------MSTGG----DFG---------NPLRKFKLVFLGEQSVGKTSLITRFMYD--SF--D-N----TY-----------QATIG--ID 49

RAB6B Q9NRW1 ------------------------------------MSAGG----DFG---------NPLRKFKLVFLGEQSVGKTSLITRFMYD--SF--D-N----TY-----------QATIG--ID 49

RAB6C Q9H0N0 ------------------------------------MSAGG----DFG---------NPLRKFKLVFLGEQSVAKTSLITRFRYD--SF--D-N----TY-----------QAIIG--ID 49

RAB7A P51149 ------------------------------------------------------MTSRKKVLLKVIILGDSGVGKTSLMNQYVNK--KF--S-N----QY-----------KATIG--AD 44

RAB7B Q96AH8 ------------------------------------------------------MNPRKKVDLKLIIVGAIGVGKTSLLHQYVHK--TF--Y-E----EY-----------QTTLG--AS 44

RAB8A P61006 ------------------------------------------------------MAKTYDYLFKLLLIGDSGVGKT**C**VLFRFSED--AF--N-S----TF-----------ISTIG--ID 44

RAB8B Q92930 ------------------------------------------------------MAKTYDYLFKLLLIGDSGVGKT**C**LLFRFSED--AF--N-T----TF-----------ISTIG--ID 44

RAB9A P51151 -------------------------------------------------------MAGKSSLFKVILLGDGGVGKSSLMNRYVTN--KF--D-T----QL-----------FHTIG--VE 43

RAB9B Q9NP90 -------------------------------------------------------MSGKSLLLKVILLGDGGVGKSSLMNRYVTN--KF--D-S----QA-----------FHTIG--VE 43

RAB10 P61026 -----------------------------------------------------MAKKTYDLLFKLLLIGDSGVGKT**C**VLFRFSDD--AF--N-T----TF-----------ISTIG--ID 45

RAB11A P62491 ---------------------------------------------------MGTRDDEYDYLFKVVLIGDSGVGKSNLLSRFTRN--EF--N-L----ES-----------KSTIG--VE 47

RAB11B Q15907 ---------------------------------------------------MGTRDDEYDYLFKVVLIGDSGVGKSNLLSRFTRN--EF--N-L----ES-----------KSTIG--VE 47

RAB12 Q6IQ22 ---------------------------------------GG----QGR--RRKQPPRPADFKLQVIIIGSRGVGKTSLMERFTDD--TF--**C**-E----A**C**-----------KSTVG--VD 78

RAB13 P51153 ------------------------------------------------------MAKAYDHLFKLLLIGDSGVGKT**C**LIIRFAED--NF--N-N----TY-----------ISTIG--ID 44

RAB14 P61106 ---------------------------------------------------MATAPYNYSYIFKYIIIGDMGVGKS**C**LLHQFTEK--KF--M-A----D**C**-----------PHTIG--VE 47

RAB15 P59190 ------------------------------------------------------MAKQYDVLFRLLLIGDSGVGKT**C**LL**C**RFTDN--EF--H-S----SH-----------ISTIG--VD 44

RAB17 Q9H0T7 ------------------------------------MAQAH----R---TPQPRAAPSQPRVFKLVLLGSGSVGKSSLALRYVKN--DF--K-S-----I-----------LPTVG--CA 54

RAB18 Q9NP72 ------------------------------------------------------MDEDVLTTLKILIIGESGVGKSSLLLRFTDD--TF--D-P----EL-----------AATIG--VD 44

RAB19 A4D1S5 ---------------------------------------------MHFSSSARAADENFDYLFKIILIGDSNVGKT**C**VVQHFKSG--VY--T-E----TQ-----------QNTIG--VD 53

RAB20 Q9NX57 ---------------------------------------------------------MRKPDSKIVLLGDMNVGKTSLLQRYMER--RF--P------DT-----------VSTVG--GA 40

RAB21 Q9UL25 ------------------------------------MAAAG----G---GGGGAAAAGRAYSFKVVLLGEG**C**VGKTSLVLRYCEN--KF--N-D----KH-----------ITTLQ--AS 55

RAB22A Q9UL26 ---------------------------------------------------------MALRELKVCLLGDTGVGKSSIVWRFVED--SF--D-P----NI-----------NPTIG--AS 41

RAB23 Q9ULC3 -----------------------------------------------------MLEEDMEVAIKMVVVGNGAVGKSSMIQRYCKG--IF--T-K----DY-----------KKTIG--VD 45

RAB24 Q969Q5 -------------------------------------------------------MSGQRVDVKVVMLGKEYVGKTSLVERYVHD--RF--LVG----PY-----------QNTIG--AA 44

RAB25 P57735 --------------------------------------------------MGNGTEEDYNFVFKVVLIGESGVGKTNLLSRFTRN--EF--S-H----DS-----------RTTIG--VE 48

RAB26 Q9ULW5 ---------------------------------------PL----QPGRPSLGGGVDFYDVAFKVMLVGDSGVGKT**C**LLVRFKDG--AF--LAG----TF-----------ISTVG--ID 100

RAB27A P51159 -----------------------------------------------------MSDGDYDYLIKFLALGDSGVGKTSVLYQYTDG--KF--N-S----KF-----------ITTVG--ID 45

RAB27B O00194 -----------------------------------------------------MTDGDYDYLIKLLALGDSGVGKTTFLYRYTDN--KF--N-P----KF-----------ITTVG--ID 45

RAB28 P51157 --------------------------------------------------MSDSEEESQDRQLKIVVLGDGASGKTSLTT**C**FAQE--TF--G-K----QY-----------KQTIG--LD 48

RAB29 O14966 -------------------------------------------------------MGSRDHLFKVLVVGDAAVGKTSLVQRYSQD--SF--S-K----HY-----------KSTVG--VD 43

RAB30 Q15771 -----------------------------------------------------MSMEDYDFLFKIVLIGNAGVGKT**C**LVRRFTQG--LF--P-P----GQ-----------GATIG--VD 45

RAB31 Q13636 ---------------------------------------------------------MAIRELKVCLLGDTGVGKSSIV**C**RFVQD--HF--D-H----NI-----------SPTIG--AS 41

RAB32 Q13637 ---------------------------------------GD----PGLGAAAAPAPETREHLFKVLVIGELGVGKTSIIKRYVHQ--LF--S-Q----HY-----------RATIG--VD 61

RAB33A Q14088 ------------------------------AA-------GL----ASLELDSSLDQYVQIRIFKIIVIGDSNVGKT**C**LTFRFCGG--TF--P-D----KT-----------EATIG--VD 72

RAB33B Q9H082 ------------------------------FS-------SS----GAVSGASGFLPPARSRIFKIIVIGDSNVGKT**C**LTYRFCAG--RF--P-D----RT-----------EATIG--VD 69

RAB34 Q9BZG1 --PVRR---D--RVLA---ELPQCLRKEAALHGHKDFH-PR----VTCACQEHRTGTVGFKISKVIVVGDLSVGKT**C**LINRFCKD--TF--D-K----NY-----------KATIG--VD 88

RAB35 Q15286 ------------------------------------------------------MARDYDHLFKLLIIGDSGVGKSSLLLRFADN--TF--S-G----SY-----------ITTIG--VD 44

RAB36 O95755 --PVSR---D--RVIA---SFPKWYTPEACLQLREHFH-GQ----VSAACQRRNTGTVGLKLSKVVVVGDLYVGKTSLIHRFCKN--VF--D-R----DY-----------KATIG--VD 159

RAB37 Q96AX2 -------------------------------------------------P---PCSPSYDLTGKVMLLGDTGVGKT**C**FLIQFKDG--AF--LSG----TF-----------IATVG--ID 66

RAB38 P57729 -----------------------------------------------------MQAPHKEHLYKLLVIGDLGVGKTSIIKRYVHQ--NF--S-S----HY-----------RATIG--VD 45

RAB39A Q14964 ------------------------------------------------------METIWIYQFRLIVIGDSTVGKS**C**LLHRFTQG--RF--PG-LRSPA**C**-----------DPTVG--VD 48

RAB39B Q96DA2 ------------------------------------------------------MEAIWLYQFRLIVIGDSTVGKS**C**LIRRFTEG--RF--AQ-V----S-----------DPTVG--VD 44

RAB40A Q8WXH6 ------------------------------------------------MSAPGSPDQAYDFLLKFLLVGDRDVGKSEILESLQDG--AA--E-S----PY-----------SHLGG--ID 50

RAB40B Q12829 ------------------------------------------------MSALGSPVRAYDFLLKFLLVGDSDVGKGEILASLQDG--AA--E-S----PY-----------GHPAG--ID 50

RAB40C Q96S21 ------------------------------------------------MGSQGSPVKSYDYLLKFLLVGDSDVGKGEILESLQDG--AA--E-S----PY-----------AYSNG--ID 50

RAB41 Q5JT25 ---------------------------MSAFGHDEAWMEAG----GFGLEAAERTEYQSLCKSKLLFLGEQSVGKTSIISRFMYN--SF--G-**C**----A**C**-----------QATVG--ID 67

RAB42 Q8N4Z0 -----------------------------------------------------MEAEGCRYQFRVALLGDAAVGKTSLLRSYVAG--AP--GAPEPEPEP-----------EPTVG--AE 50

RAB43 Q86YS6 ----------------------------------------M----AGPGPGPGDPDEQYDFLFKLVLVGDASVGKT**C**VVQRFKTG--AF--S-E----RQ-----------GSTIG--VD 54

RAB44 Q7Z6P3 --PHSR---E--PRAESRLEDPGMDSREAGLT-------PS----PGDPMAGGGPQANPDYLFHVIFLGDSNVGKTSFLHLLHQN--SF--A-T----GL-----------TATVG--VD 869

RASEF Q8IZ41 ------------------------------AL-------SP----QTDLVDDNAKSFSSQKAYKIVLAGDAAVGKSSFLMRLCKN--EF--R-E----NI-----------SATLG--VD 577

DIRAS1 O95057 -------------------------------------------------------MPEQSNDYRVVVFGAGGVGKSSLVLRFVKG--TF--R-D----TY-----------IPTIE--DT 43

DIRAS2 Q96HU8 -------------------------------------------------------MPEQSNDYRVAVFGAGGVGKSSLVLRFVKG--TF--R-E----SY-----------IPTVE--DT 43

DIRAS3 O95661 LKRLRL------------LP--------------------------ALLILRAFKPHRKIRDYRVVVVGTAGVGKSTLLHKWASG--NF--R-H----EY-----------LPTIE--NT 73

ERAS Q7Z444 -KPGTF-----------DLGLATWSPSFQG---------ET-----HRAQARRRDVGRQLPEYKAVVVGASGVGKSALTIQLNHQ--**C**F--V-E----DH-----------DPTIQ--DS 77

HRAS P01112 -----------------------------------------------------------MTEYKLVVVGAGGVGKSALTIQLIQN--HF--V-D----EY-----------DPTIE--DS 39

KRAS P01116 -----------------------------------------------------------MTEYKLVVVGAGGVGKSALTIQLIQN--HF--V-D----EY-----------DPTIE--DS 39

MRAS O14807 -------------------------------------------------MATSAVPSDNLPTYKLVVVGDGGVGKSALTIQFFQK--IF--V-P----DY-----------DPTIE--DS 49

NKIRAS1 Q9NYS0 ----------------------------------------------------------MGKGCKVVVCGLLSVGKTAILEQLLYG--NHTIG-M----ED-----------**C**ETME--DV 42

NKIRAS2 Q9NYR9 ----------------------------------------------------------MGKSCKVVVCGQASVGKTSILEQLLYG--NHVVG-S----EM-----------IETQE--DI 42

NRAS P01111 -----------------------------------------------------------MTEYKLVVVGAGGVGKSALTIQLIQN--HF--V-D----EY-----------DPTIE--DS 39

RALA P11233 ------------------------------------------------MAANKPKGQNSLALHKVIMVGSGGVGKSALTLQFMYD--EF--V-E----DY-----------EPTKA--DS 50

RALB P11234 ------------------------------------------------MAANKSKGQSSLALHKVIMVGSGGVGKSALTLQFMYD--EF--V-E----DY-----------EPTKA--DS 50

RAP1A P62834 -----------------------------------------------------------MREYKLVVLGSGGVGKSALTVQFVQG--IF--V-E----KY-----------DPTIE--DS 39

RAP1B P61224 -----------------------------------------------------------MREYKLVVLGSGGVGKSALTVQFVQG--IF--V-E----KY-----------DPTIE--DS 39

RAP2A P10114 -----------------------------------------------------------MREYKVVVLGSGGVGKSALTVQFVTG--TF--I-E----KY-----------DPTIE--DF 39

RAP2B P61225 -----------------------------------------------------------MREYKVVVLGSGGVGKSALTVQFVTG--SF--I-E----KY-----------DPTIE--DF 39

RAP2C Q9Y3L5 -----------------------------------------------------------MREYKVVVLGSGGVGKSALTVQFVTG--TF--I-E----KY-----------DPTIE--DF 39

RASD1 Q9Y272 MKLAAM------------IK--------------------------KMCPSDSELSIPAKNCYRMVILGSSKVGKTAIVSRFLTG--RF--E-D----AY-----------TPTIE--DF 60

RASD2 Q96D21 -----M------------MK--------------------------TLSSGNCTLSVPAKNSYRMVVLGASRVGKSSIVSRFLNG--RF--E-D----QY-----------TPTIE--DF 55

RASL10A Q92737 ----------------------------------------------------------MGGSLRVAVLGAPGVGKTAIIRQFLFG--DY--P-E----RH-----------RPTDG--PR 40

RASL10B Q96S79 ----------------------------------------------------------MVSTYRVAVLGARGVGKSAIVRQFLYN--EF--S-E----V**C**-----------VPTTA--RR 40

RASL11A Q6T310 ---LSM-----------SGHFL-----LAPI--------PE-----------SSSDYLLPKDIKLAVLGAGRVGKSAMIVRFLTK--RF--I-G----DY-----------EPNTG--KL 63

RASL11B Q9BPW5 ---IQN-----------MCTIA-----EYPA--------PG-----NAAASDCCVGAAGRRLVKIAVVGASGVGKTALVVRFLTK--RF--I-G----DY-----------ERNAG--NL 69

RasL12 Q9NYN1 -----------------MSSVF-----GKPR--------AG------------SGPQSAPLEVNLAILGRRGAGKSALTVKFLTK--RF--I-S----EY-----------DPNLE--DT 56

RERG Q96A58 --------------------------------------------------------MAKSAEVKLAIFGRAGVGKSALVVRFLTK--RF--I-W----EY-----------DPTLE--ST 42

RERGL G5EA41 -----------------------------------------------------------MNDVKLAVLGGEGTGKSALTVRFLTK--RF--I-G----EY-----------ASNFE--SI 39

RHEB Q15382 --------------------------------------------------------MPQSKSRKIAILGYRSVGKSSLTIQFVEG--QF--V-D----SY-----------DPTIE--NT 42

RHEBL1 Q8TAI7 --------------------------------------------------------MPLVRYRKVVILGYR**C**VGKTSLAHQFVEG--EF--S-E----GY-----------DPTVE--NT 42

RIT1 Q92963 -----M-----------DSG-------------------TR-----PVGSC-CSSPAGLSREYKLVMLGAGGVGKSAMTMQFISH--RF--P-E----DH-----------DPTIE--DA 57

RIT2 Q99578 -----M-----------E---------------------VE-----NEASCSPGSASGGSREYKVVMLGAGGVGKSAMTMQFISH--QF--P-D----YH-----------DPTIE--DA 56

RRAS P10301 -----M-----------SSGAAS----GTGR--------GR-----PRGGGPGPGDPPPSETHKLVVVGGGGVGKSALTIQFIQS--YF--V-S----DY-----------DPTIE--DS 65

RRAS2 P62070 ------------------------------------------------MAAAGWRDGSGQEKYRLVVVGGGGVGKSALTIQFIQS--YF--V-T----DY-----------DPTIE--DS 50

GEM P55040 KEPHQYSHRNRHSATPEDHCRRSWSSDST-----------------DSVIS----SESGNTYYRVVLIGEQGVGKSTLANIFAGV--HDSMD-S----D**C**-----------EVLGE--DT 113

REM1 O75628 PRLGQSASLN-PPTQKPSPAPDDWSSESS-----------------DS-------EGSWEALYRVVLLGDPGVGKTSLASLFAGK--QERDL-------H-----------EQLGE--DV 116

REM2 Q8IYK8 -----L-------RRAQAVDELDWPPQASSS--------GS----SDSLGSGEAAPAQKDGIFKVMLVGESGVGKSTLAGTFGGL--QGDSA-H----E------------PENPE--DT 151

RRAD P55042 LTPGALTAAA--AGTGTQGPRLDWPEDSE-----------------DSLSSGG--SDSDESVYKVLLLGAPGVGKSALARIFGGV--EDGPE-A----E--------------AAG--HT 126

CDC42 P60953 -----------------------------------------------------------MQTIKCVVVGDGAVGKT**C**LLISYTTN--KF--P-S----EY-----------VPTVF--DN 39

RAC1 P63000 -----------------------------------------------------------MQAIKCVVVGDGAVGKT**C**LLISYTTN--AF--P-G----EY-----------IPTVF--DN 39

RAC2 P15153 -----------------------------------------------------------MQAIKCVVVGDGAVGKT**C**LLISYTTN--AF--P-G----EY-----------IPTVF--DN 39

RAC3 P60763 -----------------------------------------------------------MQAIKCVVVGDGAVGKT**C**LLISYTTN--AF--P-G----EY-----------IPTVF--DN 39

RHOA P61586 ---------------------------------------------------------MAAIRKKLVIVGDGA**C**GKT**C**LLIVFSKD--QF--P-E----VY-----------VPTVF--EN 41

RHOB P62745 ---------------------------------------------------------MAAIRKKLVVVGDGA**C**GKT**C**LLIVFSKD--EF--P-E----VY-----------VPTVF--EN 41

RHOBTB1 O94844 ------------------------------------------------MDADMDYERPNVETIKCVVVGDNAVGKTRLI**C**ARACN--TT--L-T----QY-----QLLATHVPTVWAIDQ 58

RHOBTB2 Q9BYZ6 ------------------------------------------------MDSDMDYERPNVETIKCVVVGDNAVGKTRLI**C**ARACN--AT--L-T----QY-----QLLATHVPTVWAIDQ 58

RHOC P08134 ---------------------------------------------------------MAAIRKKLVIVGDGA**C**GKT**C**LLIVFSKD--QF--P-E----VY-----------VPTVF--EN 41

RHOD O00212 ---------------------------------------------MTAAQAAGEEAPPGVRSVKVVLVGDGG**C**GKTSLLMVFADG--AF--P-E----SY-----------TPTVF--ER 53

RHOF Q9HBH0 ------------------------------------M--DA----PGA-LAQTAAPGPGRKELKIVIVGDGG**C**GKTSLLMVYSQG--SF--P-E----HY-----------APSVF--EK 55

RHOG P84095 -----------------------------------------------------------MQSIKCVVVGDGAVGKT**C**LLI**C**YTTN--AF--P-K----EY-----------IPTVF--DN 39

RHOH Q15669 ----------------------------------------------------------MLSSIKCVLVGDSAVGKTSLLVRFTSE--TF--P-E----AY-----------KPTVY--EN 40

RHOJ Q9H4E5 -----------------------------------MN--CK----EGTDSSCGCRGNDEKKMLKCVVVGDGAVGKT**C**LLMSYAND--AF--P-E----EY-----------VPTVF--DH 57

RHOQ P17081 -----------------------------------------------------MAHGPGALMLKCVVVGDGAVGKT**C**LLMSYAND--AF--P-E----EY-----------VPTVF--DH 45

RHOU Q7L0Q8 ---RGG-------------R---------------GG--RG----PGEPGGRGRAGGAEGRGVKCVLVGDGAVGKTSLVVSYTTN--GY--P-T----EY-----------IPTAF--DN 85

RHOV Q96L33 -----------------------------------------------------RRSAPPELGIKCVLVGDGAVGKSSLIVSYTCN--GY--P-A----RY-----------RPTAL--DT 67

RND1 Q92730 -----M-------------K---------------ER--RA----PQ----------PVVARCKLVLVGDVQ**C**GKTAMLQVLAKD--**C**Y--P-E----TY-----------VPTVF--EN 49

RND2 P52198 -------------------------------------------------------MEGQSGRCKIVVVGDAE**C**GKTALLQVFAKD--AY--P-G----SY-----------VPTVF--EN 43

RND3 P61587 -----M-------------K---------------ER--RA----SQKLSSKSIMDPNQNVKCKIVVVGDSQ**C**GKTALLHVFAKD--**C**F--P-E----NY-----------VPTVF--EN 59

RAN P62826 ----------------------------------------------------MAAQGEPQVQFKLVLVGDGGTGKTTFVKRHLTG--EF--E-K----KY-----------VATLG--VE 46

RHOT1 Q8IXI2 ----------------------------------------------------------MKKDVRILLVGEPRVGKTSLIMSLVSE--EF--P-E----EV-----------PPRAE---E 39

RHOT2 Q8IXI1 ----------------------------------------------------------MRRDVRILLLGEAQVGKTSLILSLVGE--EF--P-E----EV-----------PPRAE---E 39

IFT22 Q9H7X7 -----------------------------------------------------------MLKAKILFVGP**C**ESGKTVLANFLTES--SD--I-T----EY-----------SPTQG--VR 39

RABL2A Q9UBK7 ------------------------------------MA-ED----KTKPSELDQGKYDADDNVKIICLGDSAVGKSKLMERFLMD--GF--Q-P----QQ-----------LSTYA--LT 57

RABL2B Q9UNT1 ------------------------------------MA-ED----KTKPSELDQGKYDADDNVKIICLGDSAVGKSKLMERFLMD--GF--Q-P----QQ-----------LSTYA--LT 57

RABL3 Q5HYI8 --------------------------------------------------------MASLDRVKVLVLGDSGVGKSSLVHLLCQN--QVLGN-P----SW-----------TVGCS--VD 44

RABL6 Q3YEC7 ----SM----------------------------------------N-QALQRRFAKGVQYNMKIVIRGDRNTGKTALWHRLQGR--PF--V-E----EY-----------IPTQE--IQ 79

RRAGA Q7L523 -------------------------------------------------------MPNTAMKKKVLLMGKSGSGKTSMRSIIFAN--YI--A------RDTRRLGAT------------- 42

RRAGB Q5VZM2 ---------------SEKTTEKENLGPRM----DPPLGEPEGS--------LGWVLPNTAMKKKVLLMGKSGSGKTSMRSIIFAN--YI--A------RDTRRLGATILDRIHSLQINSS 88

RRAGC Q9HB90 ---------------EEAAAAGGGVGAG-----------AGGGCG---------PGGADSSKPRILLMGLRRSGKSSIQKVVFHK--MS--P------N------ET-LFLESTNK---- 98

RRAGD Q9NQL2 ---------------EDELVGLADYGDGP----DSSDADPDSGTEEGV--LDFSDPFSTEVKPRILLMGLRRSGKSSIQKVVFHK--MS--P------N------ET-LFLESTNK---- 99

ARF1 P84077 ---------------FNVETV----E-----Y---------------KNISFTVWDVGGQDKIR----------------------PLWRHYFQNTQGLIFVVDSNDR-ERVNEAREELM 108

ARF3 P61204 ---------------FNVETV----E-----Y---------------KNISFTVWDVGGQDKIR----------------------PLWRHYFQNTQGLIFVVDSNDR-ERVNEAREELM 108

ARF4 P18085 ---------------FNVETV----E-----Y---------------KNICFTVWDVGGQDRIR----------------------PLWKHYFQNTQGLIFVVDSNDR-ERIQEVADELQ 108

ARF5 P84085 ---------------FNVETV----E-----Y---------------KNICFTVWDVGGQDKIR----------------------PLWRHYFQNTQGLIFVVDSNDR-ERVQESADELQ 108

ARF6 P62330 ---------------FNVETV----T-----Y---------------KNVKFNVWDVGGQDKIR----------------------PLWRHYYTGTQGLIFVVDCADR-DRIDEARQELH 104

ARL1 P40616 ---------------FNVETV----T-----Y---------------KNLKFQVWDLGGQTSIR----------------------PYWRCYYSNTDAVIYVVDS**C**DR-DRIGISKSELV 108

ARL2 P36404 ---------------FNIKTL----E-----H---------------RGFKLNIWDVGGQKSLR----------------------SYWRNYFESTDGLIWVVDSADR-QRMQDCQRELQ 107

ARL3 P36405 ---------------FNIKSV----Q-----S---------------QGFKLNVWDIGGQRKIR----------------------PYWKNYFENTDILIYVIDSADR-KRFEETGQELA 108

ARL4A P40617 ---------------FNTEKI----K-----VTL----------GNSKTVTFHFWDVGGQEKLR----------------------PLWKSYTRCTDGIVFVVDSVDV-ERMEEAKTELH 116

ARL4C P56559 ---------------FNTEKI----K-----LSN----------GTAKGISCHFWDVGGQEKLR----------------------PLWKSYSRCTDGIIYVVDSVDV-DRLEEAKTELH 109

ARL4D P49703 ---------------FNTEKI----R-----VPL----------GGSRGITFQVWDVGGQEKLR----------------------PLWRSYTRRTDGLVFVVDAAEA-ERLEEAKVELH 117

ARL5A Q9Y689 ---------------SNVEEI----V-----I---------------NNTRFLMWDIGGQESLR----------------------SSWNTYYTNTEFVIVVVDSTDR-ERISVTREELY 107

ARL5B Q96KC2 ---------------SNVEEI----V-----V---------------KNTHFLMWDIGGQESLR----------------------SSWNTYYSNTEFIILVVDSIDR-ERLAITKEELY 107

ARL5C A6NH57 ---------------SNVEEI----I-----L---------------PKTHFFMWDIVRPEALS----------------------FIWNTYYSNTEFIILVIDSTDR-DRLLTTREELY 107

ARL6 Q9H0F7 ---------------FSIEKF----K-----S---------------SSLSFTVFDMSGQGRYR----------------------NLWEHYYKEGQAIIFVIDSSDR-LRMVVAKEELD 110

ARL8A Q96BM9 ---------------FNMRKI----T-----K---------------GNVTIKLWDIGGQPRFR----------------------SMWERYCRGVSAIVYMVDAADQ-EKIEASKNELH 112

ARL8B Q9NVJ2 ---------------FNMRKV----T-----K---------------GNVTIKIWDIGGQPRFR----------------------SMWERYCRGVNAIVYMIDAADR-EKIEASRNELH 112

ARL9 Q6T311 ---------------FHAVCI----N-----T---------------EDSQMEFLEIGGSKPFR----------------------SYWEMYLSKGLLLIFVVDSADH-SRLPEAKKYLH 110

ARL10 Q8N8L6 ---------------FNSVRL----P-----T---------------KDFEVDLLEIGGSQNLR----------------------FYWKEFVSEVDVLVFVVDSADR-LRLPWARQELH 169

ARL11 Q969Q4 ---------------FNVEPL----K-----AP--------------GHVSLTLWDVGGQAPLR----------------------ASWKDYLEGTDILVYVLDSTDE-ARLPESAAELT 104

ARL13A Q5H913 ---------------SELTTL----L-----L---------------DEYELSIYDLNGDLKGR----------------------EAWPNYYAQAHGLVFVLDSSDI-RRMQEVKIILT 112

ARL13B Q3SXY8 ---------------FSKINL----R-----Q---------------GKFEVTIFDLGGGIRIR----------------------GIWKNYYAESYGVIFVVDSSDE-ERMEETKEAMS 112

ARL14 Q8N4G2 ---------------FNVEMI----E-----LE--------------RNLSLTVWDVGGQEKMR----------------------TVWGCYCENTDGLVYVVDSTDK-QRLEESQRQFE 105

ARL15 Q9NXU5 ---------------FSIKAV----P-----F---------------QNAILNVKELGGADNIR----------------------KYWSRYYQGSQGVIFVLDSASSEDDLEAARNELH 124

ARL16 Q0P5N6 ---------------TNLTDI----V-----A----------------QRKITIRELGGC--MG----------------------PIWSSYYGNCRSLLFVMDASDP-TQLSASCVQLL 121

ARL17 Q8IVW1 ---------------FCVETV----E-----Y---------------KNNTFAVWDVGSHFKIR----------------------PLWQHFFQNTKGARSPG-STHQ-GSLASGVLPIK 107

ARFRP1 Q13795 ---------------LNIGTV----D-----V---------------GKARLMFWDLGGQEELQ----------------------SLWDKYYAECHGVIYVIDSTDE-ERLAESKQAFE 116

SAR1A Q9NR31 ---------------PTSEEL----T-----I---------------AGMTFTTFDLGGHEQAR----------------------RVWKNYLPAINGIVFLVDCADH-SRLVESKVELN 116

SAR1B Q9Y6B6 ---------------PTSEEL----T-----I---------------AGMTFTTFDLGGHVQAR----------------------RVWKNYLPAINGIVFLVDCADH-ERLLESKEELD 116

TRIM23 P36406 ---------------FNVETV----E-----Y---------------KNLKFTIWDVGGKHKLR----------------------PLWKHYYLNTQAVVFVVDSSHR-DRISEAHSELA 495

IFT27 Q9BW83 ---------------LVVKTV----P--------V--------PDTGDSVELFIFDSAGKELFS----------------------EMLDKLWESPNVLCLVYDVTNE-ESFNNCS-KWL 104

RAB1A P62820 ---------------FKIRTI----E--------L----------DGKTIKLQIWDTAGQERFR----------------------TITSSYYRGAHGIIVVYDVTDQ-ESFNNVK-QWL 106

RAB1B Q9H0U4 ---------------FKIRTI----E--------L----------DGKTIKLQIWDTAGQERFR----------------------TITSSYYRGAHGIIVVYDVTDQ-ESYANVK-QWL 103

RAB1C Q92928 ---------------FKIQTI----E--------L----------DGKTIKLQIWDTAGQERFW----------------------TITSSYYRGAHGFLVVYDVTDQ-ESYANVK-QWL 103

RAB2A P61019 ---------------FGARMI----T--------I----------DGKQIKLQIWDTAGQESFR----------------------SITRSYYRGAAGALLVYDITRR-DTFNHLT-TWL 101

RAB2B Q8WUD1 ---------------FGARMV----N--------I----------DGKQIKLQIWDTAGQESFR----------------------SITRSYYRGAAGALLVYDITRR-ETFNHLT-SWL 101

RAB3A P20336 ---------------FKVKTI----Y--------R----------NDKRIKLQIWDTAGQERYR----------------------TITTAYYRGAMGFILMYDITNE-ESFNAVQ-DWS 117

RAB3B P20337 ---------------FKVKTV----Y--------R----------HEKRVKLQIWDTAGQERYR----------------------TITTAYYRGAMGFILMYDITNE-ESFNAVQ-DWA 117

RAB3C Q96E17 ---------------FKVKTV----F--------K----------NEKRIKLQIWDTAGQERYR----------------------TITTAYYRGAMGFILMYDITNE-ESFNAVQ-DWS 125

RAB3D O95716 ---------------FKVKTV----Y--------R----------HDKRIKLQIWDTAGQERYR----------------------TITTAYYRGAMGFLLMYDIANQ-ESFAAVQ-DWA 117

RAB4A P20338 ---------------FGSKII----N--------V----------GGKYVKLQIWDTAGQERFR----------------------SVTRSYYRGAAGALLVYDITSR-ETYNALT-NWL 108

RAB4B P61018 ---------------FGSRVV----N--------V----------GGKTVKLQIWDTAGQERFR----------------------SVTRSYYRGAAGALLVYDITSR-ETYNSLA-AWL 103

RAB5A P20339 ---------------FLTQTV----C--------L----------DDTTVKFEIWDTAGQERYH----------------------SLAPMYYRGAQAAIVVYDITNE-ESFARAK-NWV 115

RAB5B P61020 ---------------FLTQSV----C--------L----------DDTTVKFEIWDTAGQERYH----------------------SLAPMYYRGAQAAIVVYDITNQ-ETFARAK-TWV 115

RAB5C P51148 ---------------FLTQTV----C--------L----------DDTTVKFEIWDTAGQERYH----------------------SLAPMYYRGAQAAIVVYDITNT-DTFARAK-NWV 116

RAB6A P20340 ---------------FLSKTM----Y--------L----------EDRTVRLQLWDTAGQERFR----------------------SLIPSYIRDSTVAVVVYDITNV-NSFQQTT-KWI 108

RAB6B Q9NRW1 ---------------FLSKTM----Y--------L----------EDRTVRLQLWDTAGQERFR----------------------SLIPSYIRDSTVAVVVYDITNL-NSFQQTS-KWI 108

RAB6C Q9H0N0 ---------------FLSKTM----Y--------L----------EDGTIGLRLWDTAGQERLR----------------------SLIPRYIRDSAAAVVVYDITNV-NSFQQTT-KWI 108

RAB7A P51149 ---------------FLTKEV----M--------V----------DDRLVTMQIWDTAGQERFQ----------------------SLGVAFYRGADCCVLVFDVTAP-NTFKTLD-SWR 103

RAB7B Q96AH8 ---------------ILSKII----I--------L----------GDTTLKLQIWDTGGQERFR----------------------SMVSTFYKGSDGCILAFDVTDL-ESFEALD-IWR 103

RAB8A P61006 ---------------FKIRTI----E--------L----------DGKRIKLQIWDTAGQERFR----------------------TITTAYYRGAMGIMLVYDITNE-KSFDNIR-NWI 103

RAB8B Q92930 ---------------FKIRTI----E--------L----------DGKKIKLQIWDTAGQERFR----------------------TITTAYYRGAMGIMLVYDITNE-KSFDNIK-NWI 103

RAB9A P51151 ---------------FLNKDL----E--------V----------DGHFVTMQIWDTAGQERFR----------------------SLRTPFYRGSDCCLLTFSVDDS-QSFQNLS-NWK 102

RAB9B Q9NP90 ---------------FLNRDL----E--------V----------DGRFVTLQIWDTAGQERFK----------------------SLRTPFYRGADCCLLTFSVDDR-QSFENLG-NWQ 102

RAB10 P61026 ---------------FKIKTV----E--------L----------QGKKIKLQIWDTAGQERFH----------------------TITTSYYRGAMGIMLVYDITNG-KSFENIS-KWL 104

RAB11A P62491 ---------------FATRSI----Q--------V----------DGKTIKAQIWDTAGQERYR----------------------AITSAYYRGAVGALLVYDIAKH-LTYENVE-RWL 106

RAB11B Q15907 ---------------FATRSI----Q--------V----------DGKTIKAQIWDTAGQERYR----------------------AITSAYYRGAVGALLVYDIAKH-LTYENVE-RWL 106

RAB12 Q6IQ22 ---------------FKIKTV----E--------L----------RGKKIRLQIWDTAGQERFN----------------------SITSAYYRSAKGIILVYDITKK-ETFDDLP-KWM 137

RAB13 P51153 ---------------FKIRTV----D--------I----------EGKKIKLQVWDTAGQERFK----------------------TITTAYYRGAMGIILVYDITDE-KSFENIQ-NWM 103

RAB14 P61106 ---------------FGTRII----E--------V----------SGQKIKLQIWDTAGQERFR----------------------AVTRSYYRGAAGALMVYDITRR-STYNHLS-SWL 106

RAB15 P59190 ---------------FKMKTI----E--------V----------DGIKVRIQIWDTAGQERYQ----------------------TITKQYYRRAQGIFLVYDISSE-RSYQHIM-KWV 103

RAB17 Q9H0T7 ---------------FFTKVV----D--------V----------GATSLKLEIWDTAGQEKYH----------------------SVCHLYFRGANAALLVYDITRK-DSFLKAQ-QWL 113

RAB18 Q9NP72 ---------------FKVKTI----S--------V----------DGNKAKLAIWDTAGQERFR----------------------TLTPSYYRGAQGVILVYDVTRR-DTFVKLD-NWL 103

RAB19 A4D1S5 ---------------FTVRSL----D--------I----------DGKKVKMQVWDTAGQERFR----------------------TITQSYYRSAHAAIIAYDLTRR-STFESIP-HWI 112

RAB20 Q9NX57 ---------------FYLKQW----R--------S--------------YNISIWDTAGREQFH----------------------GLGSMYCRGAAAIILTYDVNHR-QSLVELE-DRF 95

RAB21 Q9UL25 ---------------FLTKKL----N--------I----------GGKRVNLAIWDTAGQERFH----------------------ALGPIYYRDSNGAILVYDITDE-DSFQKVK-NWV 114

RAB22A Q9UL26 ---------------FMTKTV----Q--------Y----------QNELHKFLIWDTAGQERFR----------------------ALAPMYYRGSAAAIIVYDITKE-ETFSTLK-NWV 100

RAB23 Q9ULC3 ---------------FLERQI----Q--------V----------NDEDVRLMLWDTAGQEEFD----------------------AITKAYYRGAQACVLVFSTTDR-ESFEAVS-SWR 104

RAB24 Q969Q5 ---------------FVAKVM----S--------V----------GDRTVTLGIWDTAGSERYE----------------------AMSRIYYRGAKAAIV**C**YDLTDS-SSFERAK-FWV 103

RAB25 P57735 ---------------FSTRTV----M--------L----------GTAAVKAQIWDTAGLERYR----------------------AITSAYYRGAVGALLVFDLTKH-QTYAVVE-RWL 107

RAB26 Q9ULW5 ---------------FRNKVL----D--------V----------DGVKVKLQMWDTAGQERFR----------------------SVTHAYYRDAHALLLLYDVTNK-ASFDNIQ-AWL 159

RAB27A P51159 ---------------FREKRV----V--------YRASGPDGATGRGQRIHLQLWDTAGQERFR----------------------SLTTAFFRDAMGFLLLFDLTNE-QSFLNVR-NWI 114

RAB27B O00194 ---------------FREKRV----V--------YNAQGPNGSSGKAFKVHLQLWDTAGQERFR----------------------SLTTAFFRDAMGFLLMFDLTSQ-QSFLNVR-NWM 114

RAB28 P51157 ---------------FFLRR----IT--------L---------PGNLNVTLQIWDIGGQTIGG----------------------KMLDKYIYGAQGVLLVYDITNY-QSFENLE-DWY 108

RAB29 O14966 ---------------FALKVL----Q--------W---------SDYEIVRLQLWDIAGQERFT----------------------SMTRLYYRDASACVIMFDVTNA-TTFSNSQ-RWK 103

RAB30 Q15771 ---------------FMIKTV----E--------I----------NGEKVKLQIWDTAGQERFR----------------------SITQSYYRSANALILTYDITCE-ESFRCLP-EWL 104

RAB31 Q13636 ---------------FMTKTV----P--------C----------GNELHKFLIWDTAGQERFH----------------------SLAPMYYRGSAAAVIVYDITKQ-DSFYTLK-KWV 100

RAB32 Q13637 ---------------FALKVL----N--------W---------DSRTLVRLQLWDIAGQERFG----------------------NMTRVYYKEAVGAFVVFDISRS-STFEAVL-KWK 121

RAB33A Q14088 ---------------FREKTV----E--------I----------EGEKIKVQVWDTAGQERFRK---------------------SMVEHYYRNVHAVVFVYDVTKM-TSFTNLK-MWI 132

RAB33B Q9H082 ---------------FRERAV----E--------I----------DGERIKIQLWDTAGQERFRK---------------------SMVQHYYRNVHAVVFVYDMTNM-ASFHSLP-SWI 129

RAB34 Q9BZG1 ---------------FEMERF----E--------V----------LGIPFSLQLWDTAGQERFK----------------------CIASTYYRGAQAIIIVFNLNDV-ASLEHTK-QWL 147

RAB35 Q15286 ---------------FKIRTV----E--------I----------NGEKVKLQIWDTAGQERFR----------------------TITSTYYRGTHGVIVVYDVTSA-ESFVNVK-RWL 103

RAB36 O95755 ---------------FEIERF----E--------I----------AGIPYSLQIWDTAGQEKFK----------------------CIASAYYRGAQVIITAFDLTDV-QTLEHTR-QWL 218

RAB37 Q96AX2 ---------------FRNKVV----T--------V----------DGVRVKLQIWDTAGQERFR----------------------SVTHAYYRDAQALLLLYDITNK-SSFDNIR-AWL 125

RAB38 P57729 ---------------FALKVL----H--------W---------DPETVVRLQLWDIAGQERFG----------------------NMTRVYYREAMGAFIVFDVTRP-ATFEAVA-KWK 105

RAB39A Q14964 ---------------FFSRLL----E--------I---------EPGKRIKLQLWDTAGQERFR----------------------SITRSYYRNSVGGFLVFDITNR-RSFEHVK-DWL 108

RAB39B Q96DA2 ---------------FFSRLV----E--------I---------EPGKRIKLQIWDTAGQERFR----------------------SITRAYYRNSVGGLLLFDITNR-RSFQNVH-EWL 104

RAB40A Q8WXH6 ---------------YKTTTI----L--------L----------DGQRVKLKLWDTSGQGRFC----------------------TIFRSYSRGAQGVILVYDIANR-WSFEGMD-RWI 109

RAB40B Q12829 ---------------YKTTTI----L--------L----------DGRRVKLQLWDTSGQGRFC----------------------TIFRSYSRGAQGVILVYDIANR-WSFDGID-RWI 109

RAB40C Q96S21 ---------------YKTTTI----L--------L----------DGRRVKLELWDTSGQGRFC----------------------TIFRSYSRGAQGILLVYDITNR-WSFDGID-RWI 109

RAB41 Q5JT25 ---------------FLSKTM----Y--------L----------EDQIVQLQLWDTAGQERFH----------------------SLIPSYIRDSTIAVVVYDITNI-NSFKETD-KWV 126

RAB42 Q8N4Z0 ---------------CYRRAL----Q--------L---------RAGPRVKLQLWDTAGHERFR----------------------CITRSFYRNVVGVLLVFDVTNR-KSFEHIQ-DWH 110

RAB43 Q86YS6 ---------------FTMKTL----E--------I----------QGKRVKLQIWDTAGQERFR----------------------TITQSYYRSANGAILAYDITKR-SSFLSVP-HWI 113

RAB44 Q7Z6P3 ---------------FRVKTL----L--------V----------DNKCFVLQLWDTAGQERYH----------------------SMTRQLLRKADGVVLMYDITSQ-ESFAHVR-YWL 928

RASEF Q8IZ41 ---------------FQMKTL----I--------V----------DGERTVLQLWDTAGQERFR----------------------SIAKSYFRKADGVLLLYDVTCE-KSFLNIR-EWV 636

DIRAS1 O95057 ----------------YRQV----IS--------C----------DKSVCTLQITDTTGSHQFP----------------------AMQRLSISKGHAFILVFSVTSK-QSLEELG-PIY 101

DIRAS2 Q96HU8 ----------------YRQV----IS--------C----------DKSICTLQITDTTGSHQFP----------------------AMQRLSISKGHAFILVYSITSR-QSLEELK-PIY 101

DIRAS3 O95661 ----------------YCQL----LG--------C----------SHGVLSLHITDSKSGDGNR----------------------ALQRHVIARGHAFVLVYSVTKK-ETLEELK-AFY 131

ERAS Q7Z444 ----------------YWKE----LT--------L----------DSGDCILNVLDTAGQAIHR----------------------ALRDQCLAVCDGVLGVFALDDP-SSLIQLQ-Q-- 133

HRAS P01112 ----------------YRKQ----VV--------I----------DGETCLLDILDTAGQEEYS----------------------AMRDQYMRTGEGFLCVFAINNT-KSFEDIH-QYR 97

KRAS P01116 ----------------YRKQ----VV--------I----------DGETCLLDILDTAGQEEYS----------------------AMRDQYMRTGEGFLCVFAINNT-KSFEDIH-HYR 97

MRAS O14807 ----------------YLKH----TE--------I----------DNQWAILDVLDTAGQEEFS----------------------AMREQYMRTGDGFLIVYSVTDK-ASFEHVD-RFH 107

NKIRAS1 Q9NYS0 ----------------YMAS----VE--------T---------DRGVKEQLHLYDTRG5QEGV----------------------ELPKHYFSFADGFVLVYSVNNL-ESFQRVE-LLK 101

NKIRAS2 Q9NYR9 ----------------YVGS----IE--------T---------DRGVREQVRFYDTRGLRDGA----------------------ELPRHCFSCTDGYVLVYSTDSR-ESFQRVE-LLK 101

NRAS P01111 ----------------YRKQ----VV--------I----------DGETCLLDILDTAGQEEYS----------------------AMRDQYMRTGEGFLCVFAINNS-KSFADIN-LYR 97

RALA P11233 ----------------YRKK----VV--------L----------DGEEVQIDILDTAGQEDYA----------------------AIRDNYFRSGEGFLCVFSITEM-ESFAATA-DFR 108

RALB P11234 ----------------YRKK----VV--------L----------DGEEVQIDILDTAGQEDYA----------------------AIRDNYFRSGEGFLLVFSITEH-ESFTATA-EFR 108

RAP1A P62834 ----------------YRKQ----VE--------V----------DCQQCMLEILDTAGTEQFT----------------------AMRDLYMKNGQGFALVYSITAQ-STFNDLQ-DLR 97

RAP1B P61224 ----------------YRKQ----VE--------V----------DAQQCMLEILDTAGTEQFT----------------------AMRDLYMKNGQGFALVYSITAQ-STFNDLQ-DLR 97

RAP2A P10114 ----------------YRKE----IE--------V----------DSSPSVLEILDTAGTEQFA----------------------SMRDLYIKNGQGFILVYSLVNQ-QSFQDIK-PMR 97

RAP2B P61225 ----------------YRKE----IE--------V----------DSSPSVLEILDTAGTEQFA----------------------SMRDLYIKNGQGFILVYSLVNQ-QSFQDIK-PMR 97

RAP2C Q9Y3L5 ----------------YRKE----IE--------V----------DSSPSVLEILDTAGTEQFA----------------------SMRDLYIKNGQGFILVYSLVNQ-QSFQDIK-PMR 97

RASD1 Q9Y272 ----------------HRKF----YS--------I----------RGEVYQLDILDTSGNHPFP----------------------AMRRLSILTGDVFILVFSLDNR-DSFEEVQ-RLR 118

RASD2 Q96D21 ----------------HRKV----YN--------I----------RGDMYQLDILDTSGNHPFP----------------------AMRRLSILTGDVFILVFSLDNR-ESFDEVK-RLQ 113

RASL10A Q92737 ---------------LYRPA----VL--------L----------DGAVYDLSIRDGDVAGPGSSPGG-------P------EEWPDAKDWSLQDTDAFVLVYDI**C**SP-DSFDYVK-ALR 108

RASL10B Q96S79 ---------------LYLPA----VV--------M----------NGHVHDLQILDFPPIS-AFPVNT-------L------QEWADTCCRGLRSVHAYILVYDI**C**CF-DSFEYVK-TIR 107

RASL11A Q6T310 ----------------YSRL----VY--------V----------EGDQLSLQIQDTPGGVQIQD---S------------LPQVVDSLSKCVQWAEGFLLVYSITDY-DSYLSIR-PLY 128

RASL11B Q9BPW5 ----------------YTRQ----VQ--------I----------EGETLALQVQDTPGIQVHEN---S-------------LSCSEQLNRCIRWADAVVIVFSITDY-KSYELIS-QLH 133

RASL12 Q9NYN1 ----------------YSSE----ET--------V----------DHQPVHLRVMDTADLDTPR-----------------------NCERYLNWAHAFLVVYSVDSR-QSFDSSS-SYL 113

RERG Q96A58 ----------------YRHQ----AT--------I----------DDEVVSMEILDTAGQEDTI-----------------------QREGHMRWGEGFVLVYDITDR-GSFEEVL-PLK 99

RERGL G5EA41 ----------------YKKH----LC--------L----------ERKQLNLEIYDPCSQR----------------------------------------------------------- 62

RHEB Q15382 ----------------FTKL----IT--------V----------NGQEYHLQLVDTAGQDEYS----------------------IFPQTYSIDINGYILVYSVTSI-KSFEVIK-VIH 100

RHEBL1 Q8TAI7 ----------------YSKI----VT--------L----------GKDEFHLHLVDTAGQDEYS----------------------ILPYSFIIGVHGYVLVYSVTSL-HSFQVIE-SLY 100

RIT1 Q92963 ----------------YKIR----IR--------I----------DDEPANLDILDTAGQAEFT----------------------AMRDQYMRAGEGFII**C**YSITDR-RSFHEVR-EFK 115

RIT2 Q99578 ----------------YKTQ----VR--------I----------DNEPAYLDILDTAGQAEFT----------------------AMREQYMRGGEGFII**C**YSVTDR-QSFQEAA-KFK 114

RRAS P10301 ----------------YTKI----CS--------V----------DGIPARLDILDTAGQEEFG----------------------AMREQYMRAGHGFLLVFAINDR-QSFNEVG-KLF 123

RRAS2 P62070 ----------------YTKQ----CV--------I----------DDRAARLDILDTAGQEEFG----------------------AMREQYMRTGEGFLLVFSVTDR-GSFEEIY-KFQ 108

GEM P55040 ----------------YERT----LM--------V----------DGESATIILLDMWENKG-EN---E-----------------WLHDHCMQVGDAYLIVYSITDR-ASFEKAS-ELR 172

REM1 O75628 ----------------YERT----LT--------V----------DGEDTTLVVVDTWEAEKLDK---S-----------------WSQESCLQGGSAYVIVYSIADR-GSFESAS-ELR 176

REM2 Q8IYK8 ----------------YERR----IM--------V----------DKEEVTLVVYDIWEQGD-AG---G-----------------WLRDHCLQTGDAFLIVFSVTDR-RSFSKVP-ETL 210

RRAD P55042 ----------------YDRS----IV--------V----------DGEEASLMVYDIWEQDG--G---R-----------------WLPGHCMAMGDAYVIVYSVTDK-GSFEKAS-ELR 184

CDC42 P60953 ----------------YAVTV----M--------I----------GGEPYTLGLFDTAGQEDYD----------------------RLRPLSYPQTDVFLV**C**FSVVSP-SSFENVKEKWV 98

RAC1 P63000 ----------------YSANV----M--------V----------DGKPVNLGLWDTAGQEDYD----------------------RLRPLSYPQTDVFLI**C**FSLVSP-ASFENVRAKWY 98

RAC2 P15153 ----------------YSANV----M--------V----------DSKPVNLGLWDTAGQEDYD----------------------RLRPLSYPQTDVFLI**C**FSLVSP-ASYENVRAKWF 98

RAC3 P60763 ----------------YSANV----M--------V----------DGKPVNLGLWDTAGQEDYD----------------------RLRPLSYPQTDVFLI**C**FSLVSP-ASFENVRAKWY 98

RHOA P61586 ----------------YVADI----E--------V----------DGKQVELALWDTAGQEDYD----------------------RLRPLSYPDTDVILM**C**FSIDSP-DSLENIPEKWT 100

RHOB P62745 ----------------YVADI----E--------V----------DGKQVELALWDTAGQEDYD----------------------RLRPLSYPDTDVILM**C**FSVDSP-DSLENIPEKWV 100

RHOBTB1 O94844 ----------------YRVCQ----EVLERSRDVV----------DEVSVSLRLWDTFGDHHKD------------------------RRFAYGRSDVVVL**C**FSIANP-NSLNHVKSMWY 123

RHOBTB2 Q9BYZ6 ----------------YRVCQ----EVLERSRDVV----------DDVSVSLRLWDTFGDHHKD------------------------RRFAYGRSDVVVL**C**FSIANP-NSLHHVKTMWY 123

RHOC P08134 ----------------YIADI----E--------V----------DGKQVELALWDTAGQEDYD----------------------RLRPLSYPDTDVILM**C**FSIDSP-DSLENIPEKWT 100

RHOD O00212 ----------------YMVNL----Q--------V----------KGKPVHLHIWDTAGQDDYD----------------------RLRPLFYPDASVLLL**C**FDVTSP-NSFDNIFNRWY 112

RHOF Q9HBH0 ----------------YTASV----T--------V----------GSKEVTLNLYDTAGQEDYD----------------------RLRPLSYQNTHLVLI**C**YDVMNP-TSYDNVLIKWF 114

RHOG P84095 ----------------YSAQS----A--------V----------DGRTVNLNLWDTAGQEEYD----------------------RLRTLSYPQTNVFVI**C**FSIASP-PSYENVRHKWH 98

RHOH Q15669 ----------------TGVDV----F--------M----------DGIQISLGLWDTAGNDAFR----------------------SIRPLSYQQADVVLM**C**YSVANH-NSFLNLKNKWI 99

RHOJ Q9H4E5 ----------------YAVTV----T--------V----------GGKQHLLGLYDTAGQEDYN----------------------QLRPLSYPNTDVFLI**C**FSVVNP-ASYHNVQEEWV 116

RHOQ P17081 ----------------YAVSV----T--------V----------GGKQYLLGLYDTAGQEDYD----------------------RLRPLSYPMTDVFLI**C**FSVVNP-ASFQNVKEEWV 104

RHOU Q7L0Q8 ----------------FSAVV----S--------V----------DGRPVRLQLCDTAGQDEFD----------------------KLRPLCYTNTDIFLL**C**FSVVSP-SSFQNVSEKWV 144

RHOV Q96L33 ----------------FSVQV----L--------V----------DGAPVRIELWDTAGQEDFD----------------------RLRSLCYPDTDVFLA**C**FSVVQP-SSFQNITEKWL 126

RND1 Q92730 ----------------YTACL----E--------T----------EEQRVELSLWDTSGSPYYD----------------------NVRPLCYSDSDAVLL**C**FDISRP-ETVDSALKKWR 108

RND2 P52198 ----------------YTASF----E--------I----------DKRRIELNMWDTSGSSYYD----------------------NVRPLAYPDSDAVLI**C**FDISRP-ETLDSVLKKWQ 102

RND3 P61587 ----------------YTASF----E--------I----------DTQRIELSLWDTSGSPYYD----------------------NVRPLSYPDSDAVLI**C**FDISRP-ETLDSVLKKWK 118

RAN P62826 ---------------VHPLVF----H--------T----------NRGPIKFNVWDTAGQEKFG----------------------GLRDGYYIQAQCAIIMFDVTSR-VTYKNVP-NWH 105

RHOT1 Q8IXI2 ----------------ITIPA----D--------V----------TPERVPTHIVDYSEAEQSD----------------------EQLHQEISQANVICIVYAVNNK-HSIDKVTSRWI 98

RHOT2 Q8IXI1 ----------------ITIPA----D--------V----------TPEKVPTHIVDYSEAEQTD----------------------EELREEIHKANVVCVVYDVSEE-ATIEKIRTKWI 98

IFT22 Q9H7X7 ---------------ILEFENPHVTS--------N---------NKGTGCEFELWDCGGDAKFE----------------------SCWPALMKDAHGVVIVFNADIP-SHRKEME-MWY 103

RABL2A Q9UBK7 ---------------LYKHTA----T--------V----------DGKTILVDFWDTAGQERFQ----------------------SMHASYYHKAHACIMVFDIQRK-VTYRNLS-TWY 116

RABL2B Q9UNT1 ---------------LYKHTA----T--------V----------DGRTILVDFWDTAGQERFQ----------------------SMHASYYHKAHACIMVFDVQRK-VTYRNLS-TWY 116

RABL3 Q5HYI8 ---------------VRVHDY--KEG--------T---------PEEKTYYIELWDVGGSVGSAS------------------SVKSTRAVFYNSVNGIIFVHDLTNK-KSSQNLR-RWS 110

RABL6 Q3YEC7 ---------------VTSIHW--SYK--------T----------TDDIVKVEVWDVVDKGKCKKRGDGLKMENDPQEAESEMALDAEFLDVYKNCNGVVMMFDITKQ-WTFNYIL-REL 162

RRAGA Q7L523 ---------------IDVEHS----H-------V----------RFLGNLVLNLWDCGGQDTFME-----------------NYFTSQRDNIFRNVEVLIYVFDVESR-E----LEKDMH 104

RRAGB Q5VZM2 LSTYSLVDSVGNTKTFDVEHS----H-------V----------RFLGNLVLNLWDCGGQDTFME-----------------NYFTSQRDNIFRNVEVLIYVFDVESR-E----LEKDMH 165

RRAGC Q9HB90 ---------------IYKD-------------DI----------SNSSFVNFQIWDFPGQMDFFD-----------------P--TFDYEMIFRGTGALIYVIDAQDD-YM--EALTRLH 158

RRAGD Q9NQL2 ---------------ICRE-------------DV----------SNSSFVNFQIWDFPGQIDFFD-----------------P--TFDYEMIFRGTGALIFVIDSQDD-YM--EALARLH 159

ARF1 P84077 R-------MLAEDEL-------------------RDAVL-LVFANKQDLPNAMNAAEI--------------------TDKLGLH------------SL-R---HRNW------------ 153

ARF3 P61204 R-------MLAEDEL-------------------RDAVL-LVFANKQDLPNAMNAAEI--------------------TDKLGLH------------SL-R---HRNW------------ 153

ARF4 P18085 K-------MLLVDEL-------------------RDAVL-LLFANKQDLPNAMAISEM--------------------TDKLGLQ------------SL-R---NRTW------------ 153

ARF5 P84085 K-------MLQEDEL-------------------RDAVL-LVFANKQDMPNAMPVSEL--------------------TDKLGLQ------------HL-R---SRTW------------ 153

ARF6 P62330 R-------IINDREM-------------------RDAII-LIFANKQDLPDAMKPHEI--------------------QEKLGLT------------RI-R---DRNW------------ 149

ARL1 P40616 A-------MLEEEEL-------------------RKAIL-VVFANKQDMEQAMTSSEM--------------------ANSLGLP------------AL-K---DRKW------------ 153

ARL2 P36404 S-------LLVEERL-------------------AGATL-LIFANKQDLPGALSSNAI--------------------REVLELD------------SI-R---SHHW------------ 152

ARL3 P36405 E-------LLEEEKL-------------------SCVPV-LIFANKQDLLTAAPASEI--------------------AEGLNLH------------TI-R---DRVW------------ 153

ARL4A P40617 K-------ITRISEN-------------------QGVPV-LIVANKQDLRNSLSLSEI--------------------EKLLAMG------------EL-S--SSTPW------------ 162

ARL4C P56559 K-------VTKFAEN-------------------QGTPL-LVIANKQDLPKSLPVAEI--------------------EKQLALH------------EL-I--PATTY------------ 155

ARL4D P49703 R-------ISRASDN-------------------QGVPV-LVLANKQDQPGALSAAEV--------------------EKRLAVR------------EL-A--AATLT------------ 163

ARL5A Q9Y689 K-------MLAHEDL-------------------RKAGL-LIFANKQDVKECMTVAEI--------------------SQFLKLT------------SI-K---DHQW------------ 152

ARL5B Q96KC2 R-------MLAHEDL-------------------RKAAV-LIFANKQDMKGCMTAAEI--------------------SKYLTLS------------SI-K---DHPW------------ 152

ARL5C A6NH57 K-------MLAHEAL-------------------QDASV-LIFANKQDVKDSMRMVEI--------------------SHFLTLS------------TI-K---DHSW------------ 152

ARL6 Q9H0F7 T-------LLNHPDI-----------------KHRRIPI-LFFANKMDLRDAVTSVKV--------------------SQLLCLE------------NI-K---DKPW------------ 157

ARL8A Q96BM9 N-------LLDKPQL-------------------QGIPV-LVLGNKRDLPGALDEKEL--------------------IEKMNLS------------AI-Q---DREI------------ 157

ARL8B Q9NVJ2 N-------LLDKPQL-------------------QGIPV-LVLGNKRDLPNALDEKQL--------------------IEKMNLS------------AI-Q---DREI------------ 157

ARL9 Q6T311 Q-------LIAANP---------------------VLPL-VVFANKQDLEAAYHITDI--------------------HEALALS------------EV-G--NDRKM------------ 154

ARL10 Q8N8L6 K-------LLDKDP---------------------DLPV-VVVANKQDLSEAMSMGEL--------------------QRELGLQ------------AI-D--NQREV------------ 213

ARL11 Q969Q4 E-------VLNDPNM-------------------AGVPF-LVLANKQEAPDALPLLKI--------------------RNRLSLE------------RF-Q---DHCW------------ 149

ARL13A Q5H913 H-------LLSDKRV-------------------AGKPI-LILANKQDKKKALMPCDI--------------------IDYLLLK------------KL-VKENKCPC------------ 160

ARL13B Q3SXY8 E-------MLRHPRI-------------------SGKPI-LVLANKQDKEGALGEADV--------------------IECLSLE------------KL-VNEHKCLC------------ 160

ARL14 Q8N4G2 H-------ILKNEHI-------------------KNVPV-VLLANKQDMPGALTAEDI--------------------TRMFKVK------------KL-C--SDRNW------------ 151

ARL15 Q9NXU5 S-------ALQHPQL-------------------CTLPF-LILANHQDKPAARSVQEI--------------------KKYFELE------------PL-A--RGKRW------------ 170

ARL16 Q0P5N6 G-------LLSAEQL-------------------AEASV-LILFNKIDLPCYMSTEEM--------------------KSLIRLP------------DI-IACAKQNI------------ 169

ARL17 Q8IVW1 C--SHVE-FGMWKGG-------------------RSHPF-LPHSSR**C**AG----SGGQL--------------------DSILP---------------H-Q---SPAW------------ 149

ARFRP1 Q13795 K-------VVTSEAL-------------------CGVPV-LVLANKQDVETCLSIPDI--------------------KTAFSDCTS----------KI-G---RRDC------------ 163

SAR1A Q9NR31 A-------LMTDETI-------------------SNVPI-LILGNKIDRTDAISEEKL--------------------REIFGLYGQTTGKGNVTLKEL-N---ARPM------------ 173

SAR1B Q9Y6B6 S-------LMTDETI-------------------ANVPI-LILGNKIDRPEAISEERL--------------------REMFGLYGQTTGKGSISLKEL-N---ARPL------------ 173

TRIM23 P36406 K-------LLTEKEL-------------------RDALL-LIFANKQDVAGALSVEEI--------------------TELLSLH------------KL-C--CGRSW------------ 541

IFT27 Q9BW83 E------KARSQAPG-------------------ISLPG-VLVGNKTDLAG------R--------------------RAVDSAEAR----------AW-----ALG-Q----------- 145

RAB1A P62820 Q------EIDRYASE--------------------NVNK-LLVGNK**C**DLTT------K--------------------KVVDYTTAK----------EF-----ADS-L----------- 146

RAB1B Q9H0U4 Q------EIDRYASE--------------------NVNK-LLVGNKSDLTT------K--------------------KVVDNTTAK----------EF-----ADS-L----------- 143

RAB1C Q92928 Q------EIDRHASE--------------------NVNK-LLVGNKSDLTT------K--------------------KVVDNTTAK----------EF-----ADS-L----------- 143

RAB2A P61019 E------DARQHSNS--------------------NMVI-MLIGNKSDLES------R--------------------REVKKEEGE----------AF-----ARE-H----------- 141

RAB2B Q8WUD1 E------DARQHSSS--------------------NMVI-MLIGNKSDLES------R--------------------RDVKREEGE----------AF-----ARE-H----------- 141

RAB3A P20336 T------QIKTYSWD--------------------NAQV-LLVGNK**C**DMED------E--------------------RVVSSERGR----------QL-----ADH-L----------- 157

RAB3B P20337 T------QIKTYSWD--------------------NAQV-ILVGNK**C**DMEE------E--------------------RVVPTEKGQ----------LL-----AEQ-L----------- 157

RAB3C Q96E17 T------QIKTYSWD--------------------NAQV-ILVGNK**C**DMED------E--------------------RVISTERGQ----------HL-----GEQ-L----------- 165

RAB3D O95716 T------QIKTYSWD--------------------NAQV-ILVGNK**C**DLED------E--------------------RVVPAEDGR----------RL-----ADD-L----------- 157

RAB4A P20338 T------DARMLASQ--------------------NIVI-ILCGNKKDLDA------D--------------------REVTFLEAS----------RF-----AQE-N----------- 148

RAB4B P61018 T------DARTLASP--------------------NIVV-ILCGNKKDLDP------E--------------------REVTFLEAS----------RF-----AQE-N----------- 143

RAB5A P20339 K------ELQRQASP--------------------NIVI-ALSGNKADLAN--------------------------KRAVDFQEAQ----------SY-----ADD-N----------- 155

RAB5B P61020 K------ELQRQASP--------------------SIVI-ALAGNKADLAN--------------------------KRMVEYEEAQ----------AY-----ADD-N----------- 155

RAB5C P51148 K------ELQRQASP--------------------NIVI-ALAGNKADLAS--------------------------KRAVEFQEAQ----------AY-----ADD-N----------- 156

RAB6A P20340 D------DVRTERGS--------------------DVII-MLVGNKTDLAD--------------------------KRQVSIEEGE----------RK-----AKE-L----------- 148

RAB6A Q9NRW1 D------DVRTERGS--------------------DVII-MLVGNKTDLAD--------------------------KRQITIEEGE----------QR-----AKE-L----------- 148

RAB6C Q9H0N0 D------DVRTERGS--------------------DVII-TLVGNRTDLAD--------------------------KRQVSVEEGE----------RK-----AKG-L----------- 148

RAB7A P51149 D------EFLIQASPR----------------DPENFPF-VVLGNKIDLEN---------------------------RQVATKRAQ----------AW-----CYS-K----------- 146

RAB7B Q96AH8 G------DVLAKIVP-----------------MEQSYPM-VLLGNKIDLAD---------------------------RKVPQEVAQ----------GW-----CRE-K----------- 145

RAB8A P61006 R------NIEEHASA--------------------DVEK-MILGNK**C**DVND------K--------------------RQVSKERGE----------KL-----ALD-Y----------- 143

RAB8B Q92930 R------NIEEHASS--------------------DVER-MILGNK**C**DMND------K--------------------RQVSKERGE----------KL-----AID-Y----------- 143

RAB9A P51151 K------EFIYYADVK----------------EPESFPF-VILGNKIDISE---------------------------RQVSTEEAQ----------AW-----CRD-N----------- 145

RAB9B Q9NP90 K------EFIYYADVK----------------DPEHFPF-VVLGNKVDKED---------------------------RQVTTEEAQ----------TW-----CME-N----------- 145

RAB10 P61026 R------NIDEHANE--------------------DVER-MLLGNK**C**DMDD------K--------------------RVVPKGKGE----------QI-----ARE-H----------- 144

RAB11A P62491 K------ELRDHADS--------------------NIVI-MLVGNKSDLRH------L--------------------RAVPTDEAR----------AF-----AEK-N----------- 146

RAB11B Q15907 K------ELRDHADS--------------------NIVI-MLVGNKSDLRH------L--------------------RAVPTDEAR----------AF-----AEK-N----------- 146

RAB12 Q6IQ22 K------MIDKYASE--------------------DAEL-LLVGNKLD**C**ET------D--------------------REITRQQGE----------KF-----AQQIT----------- 178

RAB13 P51153 K------SIKENASA--------------------GVER-LLLGNK**C**DMEA------K--------------------RKVQKEQAD----------KL-----ARE-H----------- 143

RAB14 P61106 T------DARNLTNP--------------------NTVI-ILIGNKADLEA------Q--------------------RDVTYEEAK----------QF-----AEE-N----------- 146

RAB15 P59190 S------DVDEYAPE--------------------GVQK-ILIGNKADEEQ------K--------------------RQVGREQGQ----------QL-----AKE-Y----------- 143

RAB17 Q9H0T7 K------DLEEELHP-------------------GEVLV-MLVGNKTDLSQ--------------------------EREVTFQEGK----------EF-----ADS-Q----------- 154

RAB18 Q9NP72 N------ELETYCTR-------------------NDIVN-MLVGNKIDKE-------N--------------------REVDRNEGL----------KF-----ARK-H----------- 143

RAB19 A4D1S5 H------EIEKYGAA--------------------NVVI-MLIGNK**C**DLWE------K--------------------RHVLFEDAC----------TL-----AEK-Y----------- 152

RAB20 Q9NX57 L------GLTDTASK--------------------DCLF-AIVGNKVDLTEEGALAGQEKEECSPNMDAGDRVSPRAPKQVQLEDAV----------AL-----YKKILKYKMLDEQDVP 173

RAB21 Q9UL25 K------ELRKMLGN--------------------EICL-CIVGNKIDLEK--------------------------ERHVSIQEAE----------SY-----AES-V----------- 154

RAB22A Q9UL26 K------ELRQHGPP--------------------NIVV-AIAGNK**C**DLID--------------------------VREVMERDAK----------DY-----ADS-I----------- 140

RAB23 Q9ULC3 E------KVVAEVG---------------------DIPT-VLVQNKIDLLD------D--------------------SCIKNEEAE----------AL-----AKR-L----------- 143

RAB24 Q969Q5 K------ELRSLEEG---------------------CQI-YLCGTKSDLLEED----------------------RRRRRVDFHDVQ----------DY-----ADN-I----------- 146

RAB25 P57735 K------ELYDHAEA--------------------TIVV-MLVGNKSDLSQ------A--------------------REVPTEEAR----------MF-----AEN-N----------- 147

RAB26 Q9ULW5 T------EIHEYAQH--------------------DVAL-MLLGNKVDSAH------E--------------------RVVKREDGE----------KL-----AKE-Y----------- 199

RAB27A P51159 S------QLQMHAYC-------------------ENPDI-VLCGNKSDLED------Q--------------------RVVKEEEAI----------AL-----AEK-Y----------- 155

RAB27B O00194 S------QLQANAYC-------------------ENPDI-VLIGNKADLPD------Q--------------------REVNERQAR----------EL-----ADK-Y----------- 155

RAB28 P51157 T------VVK--KVSE----------------ESETQPLVALVGNKIDLE------HM--------------------RTIKPEKHL----------R-----FCQEN-----------G 152

RAB29 O14966 Q------DLDSKLTLP----------------NGEPVPC-LLLANK**C**DLSPWA-------------------------V--SRDQID----------RF-----SKE-N----------- 146

RAB30 Q15771 R------EIEQYASN--------------------KVIT-VLVGNKIDLAE------R--------------------REVSQQRAE----------EF-----SEA-Q----------- 144

RAB31 Q13636 K------ELKEHGPE--------------------NIVM-AIAGNK**C**DLSD--------------------------IREVPLKDAK----------EY-----AES-I----------- 140

RAB32 Q13637 S------DLDSKVHLP----------------NGSPIPA-VLLANK**C**DQNKDS-------------------------SQ-SPSQVD----------QF-----CKE-H----------- 165

RAB33A Q14088 Q------ECNGHAVP-------------------PLVPK-VLVGNK**C**DLRE------Q--------------------IQVPSNLAL----------KF-----ADA-H----------- 173

RAB33B Q9H082 E------ECKQHLLA-------------------NDIPR-ILVGNK**C**DLRS------A--------------------IQVPTDLAQ----------KF-----ADT-H----------- 170

RAB34 Q9BZG1 A------DALKENDP-------------------SSVLL-FLVGSKKDLSTPA----Q--------------------YALMEKDAL----------QV-----AQE-M----------- 190

RAB35 Q15286 H------EINQNC-D--------------------DVCR-ILVGNKNDDPE------R--------------------KVVETEDAY----------KF-----AGQ-M----------- 142

RAB36 O95755 E------DALRENEA-------------------GSCFI-FLVGTKKDLLSGA----A--------------------CEQAEADAV----------HL-----ARE-M----------- 261

RAB37 Q96AX2 T------EIHEYAQR--------------------DVVI-MLLGNKADMSS------E--------------------RVIRSEDGE----------TL-----ARE-Y----------- 165

RAB38 P57729 N------DLDSKLSLP----------------NGKPVSV-VLLANK**C**DQGKDV-------------------------LMNNGLKMD----------QF-----CKE-H----------- 150

RAB39A Q14964 E------EAKMYVQP-------------------FRIVF-LLVGHK**C**DLAS------Q--------------------RQVTREEAE----------KL-----SAD-C----------- 149

RAB39B Q96DA2 E------ETKVHVQP-------------------YQIVF-VLVGHK**C**DLDT------Q--------------------RQVTRHEAE----------KL-----AAA-Y----------- 145

RAB40A Q8WXH6 K------KIEEHAP---------------------GVPK-ILVGNRLHLAF------K--------------------RQVPREQAQ----------AY-----AER-L----------- 148

RAB40B Q12829 K------EIDEHAP---------------------GVPK-ILVGNRLHLAF------K--------------------RQVPTEQAQ----------AY-----AER-L----------- 148

RAB40C Q96S21 K------EIDEHAP---------------------GVPR-ILVGNRLHLAF------K--------------------RQVPTEQAR----------AY-----AEK-N----------- 148

RAB41 Q5JT25 E------HVRAERGD--------------------DVVI-MLLGNKIDLDN--------------------------KRQVTAEQGE----------EK-----SRN-L----------- 166

RAB42 Q8N4Z0 Q------EVMATQGP-------------------DKVIF-LLVGHKSDLQS------T--------------------RCVSAQEAE----------EL-----AAS-L----------- 151

RAB43 Q86YS6 E------DVRKYAGS--------------------NIVQ-LLIGNKSDLSE------L--------------------REVSLAEAQ----------SL-----AEH-Y----------- 153

RAB44 Q7Z6P3 D------CLQDAGSD--------------------GVVI-LLLGNKMD**C**EE------E--------------------RQVSVEAGQ----------QL-----AQE-L----------- 968

RASEF Q8IZ41 D------MIEDAAHE--------------------TVPI-MLVGNKADIRDTAATEGQ--------------------KCVPGHFGE----------KL-----AMT-Y----------- 682

DIRAS1 O95057 K------LIV--QIKG--------------S-V-EDIPV-MLVGNK**C**DET------Q---------------------REVDTREAQ----------A-----VAQEW-----------K 143

DIRAS2 Q96HU8 E------QIC--EIKG--------------D-V-ESIPI-MLVGNK**C**DESP-----S---------------------REVQSSEAE----------A-----LARTW-----------K 144

DIRAS3 O95661 E------LIC--KIKG--------------NNL-HKFPI-VLVGNKSDDT------H---------------------REVALNDGA----------T-----CAMEW-----------N 174

ERAS Q7Z444 --------IW--ATWG----------------PHPAQPL-VLVGNK**C**DLV------TT--------------------AGDAHAAAA----------A-----LAHSW-----------G 174

HRAS P01112 E------QIK--RVKD----------------S-DDVPM-VLVGNK**C**DLA------A---------------------RTVESRQAQ----------D-----LARSY-----------G 138

KRAS P01116 E------QIK--RVKD----------------S-EDVPM-VLVGNK**C**DLP------S---------------------RTVDTKQAQ----------D-----LARSY-----------G 138

MRAS O14807 Q------LIL--RVKD----------------R-ESFPM-ILVANKVDLM------HL--------------------RKITREQGK----------E-----MATKH-----------N 149

NKIRAS1 Q9NYS0 K------EID--KFKD----------------K-KEVAI-VVLGNKIDLS------EQ--------------------RQVDAEVAQ----------Q-----WAKSE-----------K 143

NKIRAS2 Q9NYR9 K------EID--KSKD----------------K-KEVTI-VVLGNK**C**DLQ------EQ--------------------RRVDPDVAQ----------H-----WAKSE-----------K 143

NRAS P01111 E------QIK--RVKD----------------S-DDVPM-VLVGNK**C**DLP------T---------------------RTVDTKQAH----------E-----LAKSY-----------G 138

RALA P11233 E------QIL--RVKE----------------D-ENVPF-LLVGNKSDLE------DK--------------------RQVSVEEAK----------N-----RAEQW-----------N 150

RALB P11234 E------QIL--RVKA----------------EEDKIPL-LVVGNKSDLE------ER--------------------RQVPVEEAR----------S-----KAEEW-----------G 151

RAP1A P62834 E------QIL--RVKD----------------T-EDVPM-ILVGNK**C**DLE------DE--------------------RVVGKEQGQ----------N-----LARQW----------CN 140

RAP1B P61224 E------QIL--RVKD----------------T-DDVPM-ILVGNK**C**DLE------DE--------------------RVVGKEQGQ----------N-----LARQW----------NN 140

RAP2A P10114 D------QII--RVKR----------------Y-EKVPV-ILVGNKVDLE------SE--------------------REVSSSEGR----------A-----LAEEW-----------G 139

RAP2B P61225 D------QII--RVKR----------------Y-ERVPM-ILVGNKVDLE------GE--------------------REVSYGEGK----------A-----LAEEW-----------S 139

RAP2C Q9Y3L5 D------QIV--RVKR----------------Y-EKVPL-ILVGNKVDLE------PE--------------------REVMSSEGR----------A-----LAQEW-----------G 139

RASD1 Q9Y272 Q------QIL--DTKS--------CLKNKTKEN-VDVPL-VICGNKGDRDF-----Y---------------------REVDQREIE----------QLV---GDDPQ-----------R 170

RASD2 Q96D21 K------QIL--EVKS--------CLKNKTKEA-AELPM-VICGNKNDHGE-----LC--------------------RQVPTTEA-----------ELL---VSGDE-----------N 165

RASL10A Q92737 Q------RIAETRPAG----------------A-PEAPI-LVVGNKRDRQ------RL--------------------RFGPRRALA----------AL----VRRGW-----------R 153

RASL10B Q96S79 Q------QILETRVIG----------------T-SETPI-IIVGNKRDLQ------RG--------------------RVIPRWNVS----------HL----VRKTW-----------K 152

RASL11A Q6T310 Q------HIR--KVHP----------------D-SKAPV-IIVGNKGDLL------HA--------------------RQVQTQDGI----------Q-----LANEL-----------G 170

RASL11B Q9BPW5 Q------HVQ--QLHL----------------G-TRLPV-VVVANKADLL------HI--------------------KQVDPQLGL----------Q-----LASML-----------G 175

RASL12 Q9NYN1 E------LLALHAKET----------------Q-RSIPA-LLLGNKLDMA------QY--------------------RQVTKAEGV----------A-----LAGRF-----------G 157

RERG Q96A58 N------ILD--EIKK----------------P-KNVTL-ILVGNKADLD------HS--------------------RQVSTEEGE----------K-----LATEL-----------A 141

RERGL G5EA41 ------------------------------------------------------------------------------------------------------------------------

RHEB Q15382 G------KLL--DMVG----------------K-VQIPI-MLVGNKKDLH------ME--------------------RVISYEEGK----------A-----LAESW-----------N 142

RHEBL1 Q8TAI7 Q------KLH--EGHG----------------K-TRVPV-VLVGNKADLS------PE--------------------REVQAVEGK----------K-----LAESW-----------G 142

RIT1 Q92963 Q------LIY--RVRR----------------T-DDTPV-VLVGNKSDLK------QL--------------------RQVTKEEGL----------A-----LAREF-----------S 157

RIT2 Q99578 E------LIF--QVRH----------------T-YEIPL-VLVGNKIDLE------QF--------------------RQVSTEEGL----------S-----LAQEY-----------N 156

RRAS P10301 T------QIL--RVKD----------------R-DDFPV-VLVGNKADLE------SQ--------------------RQVPRSEAS----------A-----FGASH-----------H 165

RRAS2 P62070 R------QIL--RVKD----------------R-DEFPM-ILIGNKADLD------HQ--------------------RQVTQEEGQ----------Q-----LARQL-----------K 150

GEM P55040 I------QLR--RARQ----------------T-EDIPI-ILVGNKSDLV------RC--------------------REVSVSEGR----------A-----CAVVF-----------D 214

REM1 O75628 I------QLR--RTHQ----------------A-DHVPI-ILVGNKADLA------RC--------------------REVSVEEGR----------A-----CAVVF-----------D 218

REM2 Q8IYK8 L------RLR--AGRP----------------H-HDLPV-ILVGNKSDLA------RS--------------------REVSLEEGR----------H-----LAGTL-----------S 252

RRAD P55042 V------QLR--RARQ----------------T-DDVPI-ILVGNKSDLV------RS--------------------REVSVDEGR----------A-----CAVVF-----------D 226

CDC42 P60953 P------EITH---HC------------------PKTPF-LLVGTQIDLRDDPS-TIE-------KLAKN------KQKPITPETAE----------KL-----ARDL------------ 149

RAC1 P63000 P------EVRH---HC------------------PNTPI-ILVGTKLDLRDDKD-TIE-------KLKEK------KLTPITYPQGL----------AM-----AKEI------------ 149

RAC2 P15153 P------EVRH---HC------------------PSTPI-ILVGTKLDLRDDKD-TIE-------KLKEK------KLAPITYPQGL----------AL-----AKEI------------ 149

RAC3 P60763 P------EVRH---HC------------------PHTPI-LLVGTKLDLRDDKD-TIE-------RLRDK------KLAPITYPQGL----------AM-----AREI------------ 149

RHOA P61586 P------EVKH---FC------------------PNVPI-ILVGNKKDLRNDEH-TRR-------ELAKM------KQEPVKPEEGR----------DM-----ANRI------------ 151

RHOB P62745 P------EVKH---FC------------------PNVPI-ILVANKKDLRSDEH-VRT-------ELARM------KQEPVRTDDGR----------AM-----AVRI------------ 151

RHOBTB1 O94844 P------EIKH---FC------------------PRTPV-ILVG**C**QLDLRYADLEAVN-------RARRPLARPIKRGDILPPEKGR----------EV-----AKEL------------ 181

RHOBTB2 Q9BYZ6 P------EIKH---FC------------------PRAPV-ILVG**C**QLDLRYADLEAVN-------RARRPLARPIKPNEILPPEKGR----------EV-----AKEL------------ 181

RHOC P08134 P------EVKH---FC------------------PNVPI-ILVGNKKDLRQDEH-TRR-------ELAKM------KQEPVRSEEGR----------DM-----ANRI------------ 151

RHOD O00212 P------EVNH---FC------------------KKVPI-IVVG**C**KTDL**C**KDKS-LVN-------KLRRN------GLEPVTYHRGQ----------EM-----ARSV------------ 163

RHOF Q9HBH0 P------EVTH---FC------------------RGIPM-VLIG**C**KTDLRKDKE-QLR-------KLRAA------QLEPITYMQGL----------SA-----CEQI------------ 165

RHOG P84095 P------EVCH---HC------------------PDVPI-LLVGTKKDLRAQPD-TLR-------RLKEQ------GQAPITPQQGQ----------AL-----AKQI------------ 149

RHOH Q15669 G------EIRS---NL------------------PCTPV-LVVATQTDQREMGP---------------H------RASCVNAMEGK----------KL-----AQDV------------ 143

RHOJ Q9H4E5 P------ELKD---CM------------------PHVPY-VLIGTQIDLRDDPK-TLA-------RLLYM------KEKPLTYEHGV----------KL-----AKAI------------ 167

RHOQ P17081 P------ELKE---YA------------------PNVPF-LLIGTQIDLRDDPK-TLA-------RLNDM------KEKPICVEQGQ----------KL-----AKEI------------ 155

RHOU Q7L0Q8 P------EIRC---HC------------------PKAPI-ILVGTQSDLREDVK-VLI-------ELDKC------KEKPVPEEAAK----------LC-----AEEI------------ 195

RHOV Q96L33 P------EIRT---HN------------------PQAPV-LLVGTQADLRDDVN-VLI-------QLDQG-----GREGPVPQPQAQ----------GL-----AEKI------------ 178

RND1 Q92730 T------EILD---YC------------------PSTRV-LLIG**C**KTDLRTDLS-TLM-------ELSHQ------KQAPISYEQGC----------AI-----AKQL------------ 159

RND2 P52198 G------ETQE---FC------------------PNAKV-VLVG**C**KLDMRTDLA-TLR-------ELSKQ------RLIPVTHEQGT----------VL-----AKQV------------ 153

RND3 P61587 G------EIQE---FC------------------PNTKM-LLVG**C**KSDLRTDVS-TLV-------ELSNH------RQTPVSYDQGA----------NM-----AKQI------------ 169

RAN P62826 R------DLVRVCE---------------------NIPI-VLCGNKVDIKD---------------------------RKVKAKSIV----------FH-----RKK------------- 142

RHOT1 Q8IXI2 P------LINERTDKD------------------SRLPL-ILVGNKSDLVEYSS----------------------METILPI---------------M-----NQYT------------ 139

RHOT2 Q8IXI1 P------LVNGGTTQG------------------PRVPI-ILVGNKSDLRSGSS----------------------MEAVLPI---------------M-----SQFP------------ 139

IFT22 Q9H7X7 S------CFVQQPSLQ------------------DTQCM-LIAHHKPGSGDDKG--SL--------------------SLSPPLNKL----------KLVHSNLEDD------PEEI--R 158

RABL2A Q9UBK7 T------ELREFRP---------------------EIPC-IVVANKIDDIN------------------------------VTQKSF----------NF-----AKK-F----------- 151

RABL2B Q9UNT1 T------ELREFRP---------------------EIPC-IVVANKIDDIN------------------------------VTQKSF----------NF-----AKK-F----------- 151

RABL3 Q5HYI8 L------EALNRDLVPTGVLVTNGDYDQEQF-ADNQIPL-LVIGTKLDQIH-----ET--------------------KRHEVLTRT----------AF----LAEDFN----PEEINLD 179

RABL6 Q3YEC7 P------KVP------------------------THVPV-CVLGNYRDMG------EH--------------------RVILPDDVR----------DFI-DNLDRPP-----------G 203

RRAGA Q7L523 YYQSCLEAILQN---------------------SPDAKI-FCLVHKMDLVQEDQRD-------------------------LIFKER-----EE---DL-R----RLS------------ 152

RRAGB Q5VZM2 YYQSCLEAILQN---------------------SPDAKI-FCLVHKMDLVQEDQRD-------------------------LIFKER-----EE---DL-R----RLS------------ 213

RRAGC Q9HB90 I-------TVSKAYK-----------------VNPDMNF-EVFIHKVDGLSDDHKIET--------------------QRDIHQRAN-DDLADAGLEKL-H----LSF------------ 215

RRAGD Q9NQL2 L-------TVTRAYK-----------------VNTDINF-EVFIHKVDGLSDDHKIET--------------------QRDIHQRAN-DDLADAGLEKI-H----LSF------------ 216

ARF1 P84077 ----YIQAT**C**AT-S---G------D-GL---YEG----LDWLSNQLRNQK---------------------------------------------------------------------- 181

ARF3 P61204 ----YIQAT**C**AT-S---G------D-GL---YEG----LDWLANQLKNKK---------------------------------------------------------------------- 181

ARF4 P18085 ----YVQAT**C**AT-Q---G------T-GL---YEG----LDWLSNELSKR----------------------------------------------------------------------- 180

ARF5 P84085 ----YVQAT**C**AT-Q---G------T-GL---YDG----LDWLSHELSKR----------------------------------------------------------------------- 180

ARF6 P62330 ----YVQPS**C**AT-S---G------D-GL---YEG----LTWLTSNYKS------------------------------------------------------------------------ 175

ARL1 P40616 ----QIFKTSAT-K---G------T-GL---DEA----MEWLVETLKSRQ---------------------------------------------------------------------- 181

ARL2 P36404 ----CIQG**C**SAV-T---G------E-NL---LPG----IDWLLDDISSRIFTAD------------------------------------------------------------------ 184

ARL3 P36405 ----QIQS**C**SAL-T---G------E-GV---QDG----MNWVCKNVNAKKK--------------------------------------------------------------------- 182

ARL4A P40617 ----HLQPT**C**AI-I---G------D-GL---KEG----LEKLHDMIIKRRKMLRQQKKKR------------------------------------------------------------ 200

ARL4C P56559 ----HVQPA**C**AI-I---G------E-GL---TEG----MDKLYEMILKRRKSLKQKKKR------------------------------------------------------------- 192

ARL4D P49703 ----HVQG**C**SAV-D---G------L-GL---QQG----LERLYEMILKRKKAARGGKKRR------------------------------------------------------------ 201

ARL5A Q9Y689 ----HIQA**CC**AL-T---G------E-GL---CQG----LEWMMSRLKIR----------------------------------------------------------------------- 179

ARL5B Q96KC2 ----HIQS**CC**AL-T---G------E-GL---CQG----LEWMTSRIGVR----------------------------------------------------------------------- 179

ARL5C A6NH57 ----HIQG**CC**AL-T---R------E-GL---PAR----LQWMESQAAAN----------------------------------------------------------------------- 179

ARL6 Q9H0F7 ----HICASDAI-K---G------E-GL---QEG----VDWLQDQIQTVKT--------------------------------------------------------------------- 186

ARL8A Q96BM9 ----CCYSIS**C**K-E---K------D-NI---DIT----LQWLIQHSKSRRS--------------------------------------------------------------------- 186

ARL8B Q9NVJ2 ----CCYSIS**C**K-E---K------D-NI---DIT----LQWLIQHSKSRRS--------------------------------------------------------------------- 186

ARL9 Q6T311 ----FLFGTYLT-K---NGSEIPS---T---MQD----AKDLIAQLAADVQ--------------------------------------------------------------------- 187

ARL10 Q8N8L6 ----FLLAASIA-P---AGPTFEEP-GT---VHI----WKLLLELLS------------------------------------------------------------------------- 244

ARL11 Q969Q4 ----ELRG**C**SAL-T---G------E-GL---PEA----LQSLWSLLKSRSCMCLQARAHGAE---------------R-----------------------------GDSKRS------- 196

ARL13A Q5H913 ----RVEP**C**SAI-R---NLERRNHQ-PI---VEG----LRWLLAVIDTCQLPPTSSISISKN---------------N-----------------------------TGSGERCS----- 215

ARL13B Q3SXY8 ----QIEP**C**SAI-S---GYGKKIDK-SI---KKG----LYWLLHVIARDFDALNERIQKETT---------------E-----------------------------QRALEEQE----- 215

ARL14 Q8N4G2 ----YVQP**CC**AL-T---G------E-GL---AQG----FRKLTGFVKSHMKSRGDTLAFFKQ---------------N------------------------------------------ 192

ARL15 Q9NXU5 ----ILQP**C**SLD-D---M------D-AL---KDS----FSQLINLLEEKDHEAVRM---------------------------------------------------------------- 204

ARL16 Q0P5N6 ----TTAEISAR-E---G------T-GL---AGV----LAWLQATHRAND---------------------------------------------------------------------- 197

ARL17 Q8IVW1 ----GPWG**C**KDL-S---SG---FPS-FL---TSS----ILWKSAVVK------------------------------------------------------------------------- 177

ARFRP1 Q13795 ----LTQA**C**SAL-T---G------K-GV---REG----IEWMVKCVVRNVHRPPRQRDIT------------------------------------------------------------ 201

SAR1A Q9NR31 ----EVFM**C**SVL-K---R------Q-GY---GEG----FRWLSQYID------------------------------------------------------------------------- 198

SAR1B Q9Y6B6 ----EVFM**C**SVL-K---R------Q-GY---GEG----FRWMAQYID------------------------------------------------------------------------- 198

TRIM23 P36406 ----YIQG**C**DAR-S---G------M-GL---YEG----LDWLSRQLVAAGVLDVA----------------------------------------------------------------- 574

IFT27 Q9BW83 -GL-ECFETSVK-E---M------E-NF---EAP----FHCLAKQFHQLYREKVEVFRALA----------------------------------------------------------- 186

RAB1A P62820 -GI-PFLETSAK-N---A------T-NV---EQS----FMTMAAEIKKRMGPGATAGGAEK---S--NVKI---QS-------------------------------------------- 194

RAB1B Q9H0U4 -GI-PFLETSAK-N---A------T-NV---EQA----FMTMAAEIKKRMGPGAASGG-ER---P--NLKI---DS-------------------------------------------- 190

RAB1C Q92928 -GI-PFLETSAK-N---A------T-NV---EQA----FMTMAAEIKKQMGPGAASGG-ER---P--NLKI---DS-------------------------------------------- 190

RAB2A P61019 -GL-IFMETSAK-T---A------S-NV---EEA----FINTAKEIYEKIQEGVFDINNEA-------NGI---KIGP-------------------------------QHAAT------ 194

RAB2B Q8WUD1 -GL-IFMETSAK-T---A------C-NV---EEA----FINTAKEIYRKIQQGLFDVHNEA-------NGI---KIGP-------------------------------QQSIS------ 194

RAB3A P20336 -GF-EFFEASAK-D---N------I-NV---KQT----FERLVDVICEKMSESLDTADPAV---TGAKQGP---QLSD------------------------------------------ 209

RAB3B P20337 -GF-DFFEASAK-E---N------I-SV---RQA----FERLVDAICDKMSDSLDT-DPSM---LGSSKNT---RLSD------------------------------------------ 208

RAB3C Q96E17 -GF-EFFETSAK-D---N------I-NV---KQT----FERLVDIICDKMSESLET-DPAI---TAAKQNT---RLKE------------------------------------------ 216

RAB3D O95716 -GF-EFFEASAK-E---N------I-NV---KQV----FERLVDVICEKMNESLEPSSSS----GSNGKGP---AVGD------------------------------------------ 208

RAB4A P20338 -EL-MFLETSAL-T---G------E-NV---EEA----FVQCARKILNKIESGELDPERMG-------SGI---QYGD-------------------------------AALRQ------ 201

RAB4B P61018 -EL-MFLETSAL-T---G------E-NV---EEA----FLKCARTILNKIDSGELDPERMG-------SGI---QYGD-------------------------------ASLRQ------ 196

RAB5A P20339 -SL-LFMETSAK-T---S------M-NV---NEI----FMAIAKKLPKNEPQNPG---A---NS-ARGRGV---DLTE------------------------------------------ 203

RAB5B P61020 -SL-LFMETSAK-T---A------M-NV---NDL----FLAIAKKLPKSEPQNLG---G---AA-GRSRGV---DLHE------------------------------------------ 203

RAB5C P51148 -SL-LFMETSAK-T---A------M-NV---NEI----FMAIAKKLPKNEPQNAT---G---AP-GRNRGV---DLQE------------------------------------------ 204

RAB6A P20340 -NV-MFIETSAK-A---G------Y-NV---KQL----FRRVAAALPGMESTQDRSR-----------EDMIDIKLEK------------------------------------------ 195

RAB6B Q9NRW1 -SV-MFIETSAK-T---G------Y-NV---KQL----FRRVASALPGMENVQEKSK-----------EGMIDIKLDK------------------------------------------ 195

RAB6C Q9H0N0 -NV-TFIETRAK-A---G------Y-NV---KQL----FRRVAAALPGMESTQDGSR-----------EDMSDIKLEK------------------------------------------ 195

RAB7A P51149 -NNIPYFETSAK-E---A------I-NV---EQA----FQTIARNALKQETEVELYNEFPEP------IKL---DKND------------------------------------------ 196

RAB7B Q96AH8 --DIPYFEVSAK-N---D------I-NV---VQA----FEMLASRALSRYQSILE-N--HLT------ESI---KLS------------------------------------------- 190

RAB8A P61006 -GI-KFMETSAK-A---N------I-NV---ENA----FFTLARDIKAKMDKKLEGNSPQ-----GSNQGV---KI-------------------------------------------- 191

RAB8B Q92930 -GI-KFLETSAK-S---S------A-NV---EEA----FFTLARDIMTKLNRKMNDSNSA-----GAGGPV---KI-------------------------------------------- 191

RAB9A P51151 -GDYPYFETSAK-D---A------T-NV---AAA----FEEAVRRVLATEDRSDHLI---QT------DTV---NLHR------------------------------------------ 192

RAB9B Q9NP90 -GDYPYLETSAK-D---D------T-NV---TVA----FEEAVRQVLAVEEQLEHCM---LG------HTI---DLNS------------------------------------------ 192

RAB10 P61026 -GI-RFFETSAK-A---N------I-NI---EKA----FLTLAEDILRKTPVKEPNSENVD---ISSGGGV---T--------------------------------------------- 193

RAB11A P62491 -GL-SFIETSAL-D---S------T-NV---EAA----FQTILTEIYRIVSQKQMSDRRENDMSPSNNV-V---PIHV------------------------------------------ 200

RAB11B Q15907 -NL-SFIETSAL-D---S------T-NV---EEA----FKNILTEIYRIVSQKQIADRAAHDESPGNNV-V---DISV------------------------------------------ 200

RAB12 Q6IQ22 -GM-RFCEASAK-D---N------F-NV---DEI----FLKLVDDILKKMPLDILRNELS-------NSIL---SLQP-------------------------------EPE-------- 229

RAB13 P51153 -GI-RFFETSAK-S---S------M-NV---DEA----FSSLARDILLKSGGRRSGNGNKP---PSTD--L---K--------------------------------------------- 190

RAB14 P61106 -GL-LFLEASAK-T---G------E-NV---EDA----FLEAAKKIYQNIQDGSLDLNAAE-------SGV---QHKP-------------------------------SAPQG------ 199

RAB15 P59190 -GM-DFYETSA**C**-T---N------L-NI---KES----FTRLTELVLQAHRKELEGLRMRA---S-NELAL---AELE-------------------------------EEE-------- 197

RAB17 Q9H0T7 -KL-LFMETSAK-L---N------H-QV---SEV----FNTVAQELLQRSDEEGQA-----------LRGDAAVALN------------------------------------------- 200

RAB18 Q9NP72 -SM-LFIEASAK-T---**C**------D-GV---QCA----FEELVEKIIQTPGLWESENQ---------NKGV---KLSH-------------------------------REE-------- 192

RAB19 A4D1S5 -GLLAVLETSAK-E---S------K-NI---EEV----FVLMAKELIARNSLHLYGESALN--------GL---PLDS-------------------------------SPV-------- 203

RAB20 Q9NX57 AAEQMCFETSAK-T---G------Y-NV---DLL----FETLFDLVVPMILQQRAERPSHT---------V---DISS------------------------------------------ 221

RAB21 Q9UL25 -GA-KHYHTSAK-Q---N------K-GI---EEL----FLDLCKRMIETAQVDERAKGNGSSQPGTARRGVQIIDDEP------------------------------------------ 212

RAB22A Q9UL26 -HA-IFVETSAK-N---A------I-NI---NEL----FIEISRRIPSTDANLPS---G--------GKGF---KLRR------------------------------------------ 184

RAB23 Q9ULC3 -KL-RFYRTSVK-E---D------L-NV---NEV----FKYLAEKYLQKLKQQIAEDPELTH-SSSNKIGV---FNTSG-----------------------------GSHSGQ-NSGTL 209

RAB24 Q969Q5 -KA-QLFETSSK-T---G------Q-SV---DEL----FQKVAEDYVSVAAFQVMTE----------DKGV---DLGQ------------------------------------------ 191

RAB25 P57735 -GL-LFLETSAL-D---S------T-NV---ELA----FETVLKEIFAKVSKQRQNSIRTNAITLGSAQ--------------------------------------------------- 196

RAB26 Q9ULW5 -GL-PFMETSAK-T---G------L-NV---DLA----FTAIAKELKQRSMKAPSEPRF---------------RLHD-------------------------------YVK-------- 245

RAB27A P51159 -GI-PYFETSAA-N---G------T-NI---SQA----IEMLLDLIMKRMERCVDKSWIPEGVVRSNGH-A---STD------------------------------------------- 208

RAB27B O00194 -GI-PYFETSAA-T---G------Q-NV---EKA----VETLLDLIMKRMEQCVEKTQIPDTVNGGNSG-N------------------------------------------------- 205

RAB28 P51157 FS---SHFVSAK-T---G------D-SV---FLC----FQKVAAEILGIKLNKAEI----EQ------SQR---VVKA--DIVNYN------------QE-------------------- 204

RAB29 O14966 -GFTGWTETSVK-E---N------K-NI---NEA----MRVLIEKMMRNSTEDIMSLSTQG-------DYI---NLQT------------------------------------------ 195

RAB30 Q15771 -DM-YYLETSAK-E---S------D-NV---EKL----FLDLACRLISEARQNTLVNNV--------------------------------------------------SSP-------- 186

RAB31 Q13636 -GA-IVVETSAK-N---A------I-NI---EEL----FQGISRQIPPLDPHENG---N--------NGTI---KVEK------------------------------------------ 184

RAB32 Q13637 -GFAGWFETSAK-D---N------I-NI---EEA----ARFLVEKILVNHQSFPNE-ENDV-------DKI---KLDQ------------------------------------------ 213

RAB33A Q14088 -NM-LLFETSAK-DPKES------Q-NV---ESI----FMCLACRLKAQKSLLYRDAERQQ----GKVQKL---EF-------------------------------------------- 225

RAB33B Q9H082 -SM-PLFETSAK-NPNDN------D-HV---EAI----FMTLAHKLKSHKPLMLSQPPDN-----G----I---IL-------------------------------------------- 217

RAB34 Q9BZG1 -KA-EYWAVSSL-T---G------E-NV---REF----FFRVAALTFEANVLAELEKSGARRI----GDVV---RINS------------------------------------------ 241

RAB35 Q15286 -GI-QLFETSAK-E---N------V-NV---EEM----FNCITELVLRAKKDNLAKQQQQQ---Q--NDVV---KL-------------------------------------------- 190

RAB36 O95755 -QA-EYWSVSAK-T---G------E-NV---KAF----FSRVAALAFEQSVLQDLERQSSARLQVGNGDLI---QMEG------------------------------------------ 316

RAB37 Q96AX2 -GV-PFLETSAK-T---G------M-NV---ELA----FLAIAKELKYRAGHQADEPSF---------------QIRD-------------------------------YVE-------- 211

RAB38 P57729 -GFVGWFETSAK-E---N------I-NI---DEA----SRCLVKHILANECDLMESIEPDV-------VKP---HLT------------------------------------------- 198

RAB39A Q14964 -GM-KYIETSAK-D---A------T-NV---EES----FTILTRDIYELIKKGEICIQDGW-------EGV---KSGF------------------------------------------ 197

RAB39B Q96DA2 -GM-KYIETSAR-D---A------I-NV---EKA----FTDLTRDIYELVKRGEITIQEGW-------EGV---KSGF------------------------------------------ 193

RAB40A Q8WXH6 -GV-TFFEVSPL-**C**---N------F-NI---IES----FTELARIVLLRHRMNWLGRPSKV---------L---S--------------------------------------------- 191

RAB40B Q12829 -GV-TFFEVSPL-**C**---N------F-NI---TES----FTELARIVLLRHGMDRLWRPSKV---------L---S--------------------------------------------- 191

RAB40C Q96S21 -CM-TFFEVSPL-**C**---N------F-NV---IES----FTELSRIVLMRHGMEKIWRPNRV---------F---S--------------------------------------------- 191

RAB41 Q5JT25 -NV-MFIETSAK-T---G------Y-NV---KKL----FRRVASALLSTRTSPP-PK-----------EGTVEIELES------------------------------------------ 212

RAB42 Q8N4Z0 -GM-AFVETSVK-N---N------C-NV---DLA----FDTLADAIQQALQQGDIKLEEGW-------GGV---RLIH------------------------------------------ 199

RAB43 Q86YS6 -DILCAIETSAK-D---S------S-NV---EEA----FLRVATELIMRHGGPLFSEKSPD--------HI---QLNS-------------------------------KDI-------- 204

RAB44 Q7Z6P3 -GV-YFGE**C**SAA-L---G------H-NI---LEP----VVNLARSLRMQEEGLKD-SL------------V---KV--------------------------------------------1008

RASEF Q8IZ41 -GA-LFCETSAK-D---G------S-NI---VEA----VLHLAREVKKRTDKDDSRSITNL---TGTN---------------------------------------------------- 727

DIRAS1 O95057 CA---FMETSAK-M---N------Y-NV---KEL----FQELLTLETRRNMSLN------ID------GKR---SGK----------------QKRTD---------------------- 189

DIRAS2 Q96HU8 CA---FMETSAK-L---N------H-NV---KEL----FQELLNLEKRRTVSLQ------ID------GKK---SKQ----------------QKRKE---------------------- 190

DIRAS3 O95661 CA---FMEISAK-T---D------V-NV---QEL----FHMLLNYKKKPTTGLQ------EP------EKK---SQM----------------PNTTE---------------------- 220

ERAS Q7Z444 AH---FVETSAK-T---R------Q-GV---EEA----FSLLVHEIQRVQEAMAKE----PM------ARS---CREK----TRH----------------------------------- 221

HRAS P01112 IP---YIETSAK-T---R------Q-GV---EDA----FYTLVREIRQHKLRKLNP----P--------------DES----GPG----------------------------------- 180

KRAS P01116 IP---FIETSAK-T---R------Q-RV---EDA----FYTLVREIRQYRLKKISK----E---------------EK----TPG----------------------------------- 179

MRAS O14807 IP---YIETSAKDP---P------L-NV---DKA----FHDLVRVIRQQIPEKSQK----K--------------KKK----TKWR------------GD-------------------- 195

NKIRAS1 Q9NYS0 VR---LWEVTVT-D---R------K-TL---IEP----FTLLASKLSQPQSKSSFP----LP------GRK---NKGNS------N---------------------------------- 189

NKIRAS2 Q9NYR9 VK---LWEVSVA-D---R------R-SL---LEP----FVYLASKMTQPQSKSAFP----LS------RKN---K-GSG------S---------------------------------- 188

NRAS P01111 IP---FIETSAK-T---R------Q-GV---EDA----FYTLVREIRQYRMKKLNS----S--------------DDG----TQG----------------------------------- 180

RALA P11233 VN---YVETSAK-T---R------A-NV---DKV----FFDLMREIRARKMEDSKE----KN------GKK---KRKS---------------------L-------------------- 195

RALB P11234 VQ---YVETSAK-T---R------A-NV---DKV----FFDLMREIRTKKMSENKD----KN------GKK---SSKN------------------------------------------ 195

RAP1A P62834 CA---FLESSAK-S---K------I-NV---NEI----FYDLVRQINRKTPV-------------------------------------------------------------------- 171

RAP1B P61224 CA---FLESSAK-S---K------I-NV---NEI----FYDLVRQINRKTPV-------------------------------------------------------------------- 171

RAP2A P10114 CP---FMETSAK-S---K------T-MV---DEL----FAEIVRQMNYAAQP-------------------------------------------------------------------- 170

RAP2B P61225 CP---FMETSAK-N---K------A-SV---DEL----FAEIVRQMNYAAQP-------------------------------------------------------------------- 170

RAP2C Q9Y3L5 CP---FMETSAK-S---K------S-MV---DEL----FAEIVRQMNYSSLP-------------------------------------------------------------------- 170

RASD1 Q9Y272 CA---YFEISAK-K---N------S-SL---DQM----FRALFAMAKLPSEMSPD-----LH------RKV---SVQYC-DV-------LHKKALRNKKLLRAGSGGGGGDPGDAFGIVA 247

RASD2 Q96D21 CA---YFEVSAK-K---N------T-NV---DEM----FYVLFSMAKLPHEMSPA-----LH------RKI---SVQYG-DA-------FHPRPFCMRRV----------KEMDAYGMVS 232

RASL10A Q92737 CG---YLE**C**SAK-Y---N------W-HV---LRL----FRELLRCA-LVRARPAHP-------------------------------------ALRLQGA-------------------- 194

RASL10B Q96S79 CG---YVE**C**SAK-Y---N------W-HI---LLL----FSELLKSVGCARCKHVHA-------------------------------------ALRFQGA-------------------- 194

RASL11A Q6T310 SL---FLEISTSEN---Y------E-DV---CDV----FQHLCKEVSKMHG--LSG----ER------RRA---SIIPR-PRSPNM------------QD-------------------- 222

RASL11B Q9BPW5 CS---FYEVSVSEN---Y------N-DV---YSA----FHVLCKEVSHKQQPSSTP----EK------RRT---SLIPR-PKSPNM------------QD-------------------- 229

RAS12 Q9NYN1 CL---FFEVSA**C**LD---F------E-HV---QHV----FHEAVREARRELEKS--P----LT------RPL---FISEE-RALPHQ------------AP-------------------- 209

RERG Q96A58 CA---FYE**C**SA**C**TG---E------G-NI---TEI----FYELCREVRRRRMVQ--G----KT------RRR---SSTTH----------------------------------------- 185

RERGL G5EA41 ------------------------------------------------------------------------------------------------------------------------

RHEB Q15382 AA---FLESSAK-E---N------Q-TA---VDV----FRRIILEAEKMDGAASQG----K----------------------------------------------------------- 178

RHEBL1 Q8TAI7 AT---FMESSAR-E---N------Q-LT---QGI----FTKVIQEIARVENSYGQE---------------------------------------------------------------- 177

RIT1 Q92963 CP---FFETSAA-Y---R------Y-YI---DDV----FHALVREIRRKEKEAVLA----ME------KKS---KPKN----SVWK------------RL-------------------- 207

RIT2 Q99578 CG---FFETSAA-L---R------F-CI---DDA----FHGLVREIRKKESMPS-L----ME------KKL---KRKD----SLWK------------KL-------------------- 205

RRAS P10301 VA---YFEASAK-L---R------L-NV---DEA----FEQLVRAVRKYQEQELPP----S--------------PPS----AP------------------------------------ 206

RRAS2 P62070 VT---YMEASAK-I---R------M-NV---DQA----FHELVRVIRKFQEQECPP----S--------------PEP----TRK----------------------------------- 192

GEM P55040 CK---FIETSAA-V---Q------H-NV---KEL----FEGIVRQVRLRRDSKEKN----ER-----------------RLAYQKRKESMPRKARRFWGK-------------------- 272

REM1 O75628 CK---FIETSAT-L---Q------H-NV---AEL----FEGVVRQLRLRRRDSAAK-----------------------EPPAPRRPASLAQRARRFLAR-------------------- 274

REM2 Q8IYK8 CK---HIETSAA-L---H------H-NT---REL----FEGAVRQIRLRRGRNHAG----GQ------RPD---PGSPEGPAPPARRESLTKKAKRFLAN-------------------- 318

RRAD P55042 CK---FIETSAA-L---H------H-NV---QAL----FEGVVRQIRLRRDSKEAN----AR-----------------RQAGTRRRESLGKKAKRFLGR-------------------- 284

CDC42 P60953 -KAVKYVE**C**SAL-T---Q------K-GL---KNV----FDEAILAALEPPEPKK--S---R----------------------------------------------------------- 186

RAC1 P63000 -GAVKYLE**C**SAL-T---Q------R-GL---KTV----FDEAIRAVLCPPPVKK-RK---R----------------------------------------------------------- 187

RAC2 P15153 -DSVKYLE**C**SAL-T---Q------R-GL---KTV----FDEAIRAVLCPQPTRQ-QK---R----------------------------------------------------------- 187

RAC3 P60763 -GSVKYLE**C**SAL-T---Q------R-GL---KTV----FDEAIRAVLCPPPVKK-PG---K----------------------------------------------------------- 187

RHOA P61586 -GAFGYME**C**SAK-T---K------D-GV---REV----FEMATRAALQARRGKK-KS--------------------------------------------------------------- 188

RHOB P62745 -QAYDYLE**C**SAK-T---K------E-GV---REV----FETATRAALQKRYGSQ-N---------------------------------------------------------------- 187

RHOBTB1 O94844 -GL-PYYETSVF-D---Q------F-GI---KDV----FDNAIRAALISRRHLQFWKSHLK-------------KVQK-----------------P------------------------ 227

RHOBTB2 Q9BYZ6 -GI-PYYETSVV-A---Q------F-GI---KDV----FDNAIRAALISRRHLQFWKSHLR-------------NVQR-----------------P------------------------ 227

RHOC P08134 -SAFGYLE**C**SAK-T---K------E-GV---REV----FEMATRAGLQVRKNKR-RR--------------------------------------------------------------- 188

RHOD O00212 -GAVAYLE**C**SAR-L---H------D-NV---HAV----FQEAAEVALSSRGRNF-WRRIT------------------------------------------------------------ 203

RHOF Q9HBH0 -RAALYLE**C**SAK-F---R------E-NV---EDV----FREAAKVALSALKKAQ-RQKKRR----------------------------------------------------------- 206

RHOG P84095 -HAVRYLE**C**SAL-Q---Q------D-GV---KEV----FAEAVRAVLNPTPIKR--G---R----------------------------------------------------------- 186

RHOH Q15669 -RAKGYLE**C**SAL-S---N------R-GV---QQV----FECAVRTAVNQARRRN-RRRLFS----------------------------------------------------------- 184

RHOJ Q9H4E5 -GAQCYLE**C**SAL-T---Q------K-GL---KAV----FDEAILTIFHPKKKKK-RCSEGH----------------------------------------------------------- 208

RHOQ P17081 -GACCYVE**C**SAL-T---Q------K-GL---KTV----FDEAIIAILTPKKHTV-KKRIGS----------------------------------------------------------- 196

RHOU Q7L0Q8 -KAASYIE**C**SAL-T---Q------K-NL---KEV----FDAAIVAGIQYSDTQQ-QPKKSK-------------SRTP-----------------D------------------------ 241

RHOV Q96L33 -RACCYLE**C**SAL-T---Q------K-NL---KEV----FDSAILSAIEHKARLE------K-------------KLNA-----------------K------------------------ 219

RND1 Q92730 -GAEIYLEGSAF-T---S------EKSI---HSI----FRTASMLCLNKPSPLP-QKSPVR-------------SLSK-----------------R------------------------ 206

RND2 P52198 -GAVSYVE**C**SSR-S---S------ERSV---RDV----FHVATVASLGRGHRQL-RRTDSR-------------RGMQ-----------------R------------------------ 200

RND3 P61587 -GAATYIE**C**SAL-Q---S------ENSV---RDI----FHVATLACVNKTNKNV-KRNKSQ-------------RATK-----------------R------------------------ 216

RAN P62826 -NL-QYYDISAK-S---N------Y-NF---EKP----FLWLARKLIGDPNLEFVAMPALAP------PEV---VMDP-------------------------------ALAAQ-YEHDL 201

RHOT1 Q8IXI2 -EIETCVE**C**SAK-N---L------K-NI---SEL----FYYAQKAVLHPTGPLYCPE--EK-------------EMKP-----------------A------------------------ 184

RHOT2 Q8IXI1 -EIETCVE**C**SAK-N---L------R-NI---SEL----FYYAQKAVLHPTAPLYDPE--AK-------------QLRP-----------------A------------------------ 184

IFT22 Q9H7X7 MEFIKYLKSIIN-----------------------------SMSESRDREEMSIMT---------------------------------------------------------------- 185

RABL2A Q9UBK7 -SL-PLYFVSAA-D---G------T-NV---VKL----FNDAIRLAVSYKQNSQDFMDEIFQ----ELENF---SLEQ-------------------------------E---------- 203

RABL2B Q9UNT1 -SL-PLYFVSAA-D---G------T-NV---VKL----FNDAIRLAVSYKQNSQDFMDEIFQ----ELENF---SLEQ-------------------------------E---------- 203

RABL3 Q5HYI8 CTNPRYLAAGSS-N---A------V-KL---SRF----FDKVIEKRYFLREGNQIP-------------------------GFPDR--------KRFG---------AGTLKSLHYD--- 236

RABL6 Q3YEC7 SSYFRYAESSMK-N---S------F-GLKYLHKFFNIPFLQLQRETLLRQLETNQLDMDATL------EEL---SVQQE-------------------------------TEDQNYGIFL 272

RRAGA Q7L523 ----RPLE**C**A**C**F-R---T--SIWDE-TL---YKA----WSSIVYQLIPNVQQLEMNLRNFAQ---------------I-----------------------------IEADEVLLFERAT 210

RRAGB Q5VZM2 ----RPLE**C**S**C**F-R---T--SIWDE-TL---YKA----WSSIVYQLIPNVQQLEMNLRNFAE---------------I-----------------------------IEADEVLLFERAT 271

RRAGC Q9HB90 ----YLT--S-----------IYDH-SI---FEA----FSKVVQKLIPQLPTLENLLNIFIS---------------N-----------------------------SGIEKAFLFDVVS 266

RRAGD Q9NQL2 ----YLT--S-----------IYDH-SI---FEA----FSKVVQKLIPQLPTLENLLNIFIS---------------N-----------------------------SGIEKAFLFDVVS 267

ARF1 P84077 ------------------------------------------------------------

ARF3 P61204 ------------------------------------------------------------

ARF4 P18085 ------------------------------------------------------------

ARF5 P84085 ------------------------------------------------------------

ARF6 P62330 ------------------------------------------------------------

ARL1 P40616 ------------------------------------------------------------

ARL2 P36404 ------------------------------------------------------------

ARL3 P36405 ------------------------------------------------------------

ARL4A P40617 ------------------------------------------------------------

ARL4C P56559 ------------------------------------------------------------

ARL4D P49703 ------------------------------------------------------------

ARL5A Q9Y689 ------------------------------------------------------------

ARL5B Q96KC2 ------------------------------------------------------------

ARL5C A6NH57 ------------------------------------------------------------

ARL6 Q9H0F7 ------------------------------------------------------------

ARL8A Q96BM9 ------------------------------------------------------------

ARL8B Q9NVJ2 ------------------------------------------------------------

ARL9 Q6T311 ------------------------------------------------------------

ARL10 Q8N8L6 ------------------------------------------------------------

ARL11 Q969Q4 ------------------------------------------------------------

ARL13A Q5H913 SHSF------S---------TRTGMSK---------------EKRQH--LEQC--SIE-- 239

ARL13B Q3SXY8 KQER------A---------ERVRKLR---------------EERKQNEQEQA--ELDGT 243

ARL14 Q8N4G2 ------------------------------------------------------------

ARL15 Q9NXU5 ------------------------------------------------------------

ARL16 Q0P5N6 ------------------------------------------------------------

ARL17 Q8IVW1 ------------------------------------------------------------

ARFRP1 Q13795 ------------------------------------------------------------

SAR1A Q9NR31 ------------------------------------------------------------

SAR1B Q9Y6B6 ------------------------------------------------------------

TRIM23 P36406 ------------------------------------------------------------

IFT27 Q9BW83 ------------------------------------------------------------

RAB1A P62820 --------TP------------VKQSG----------------GGCC------------- 205

RAB1B Q9H0U4 --------TP------------VKPAG----------------GGCC------------- 201

RAB1C Q92928 --------TP------------VKPAG----------------GGCC------------- 201

RAB2A P61019 -----NATHAG-------N-QGGQQAG----------------GGCC------------- 212

RAB2B Q8WUD1 -----TSVGPSAS-QR--N-SRDIGSN----------------SGCC------------- 216

RAB3A P20336 --------QQ-------------VPPH----------------QDCAC------------ 220

RAB3B P20337 --------TP-------------PLLQ----------------QNCSC------------ 219

RAB3C Q96E17 --------TP-------------PPPQ----------------PNCAC------------ 227

RAB3D O95716 --------AP-------------APQP----------------SSCSC------------ 219

RAB4A P20338 -----LRSPRR-------A-QAPNAQE----------------CGC-------------- 218

RAB4B P61018 -----LRQPRS-------A-QAVAPQP----------------CGC-------------- 213

RAB5A P20339 ------------------P-TQP--TR----------------NQCCSN----------- 215

RAB5B P61020 ------------------Q-SQQ--NK----------------SQCCSN----------- 215

RAB5C P51148 ------------------N-NPA--SR----------------SQCCSN----------- 216

RAB6A P20340 ------------------P-QEQPVSE----------------GGCSC------------ 208

RAB6B Q9NRW1 ------------------P-QEPPASE----------------GGCSC------------ 208

RAB6C Q9H0N0 ------------------P-QEQTVSE----------------GGCSCYSPMS-----S- 214

RAB7A P51149 ----------------------RAKAS----------------AESCSC----------- 207

RAB7B Q96AH8 ----------------------PDQSR----------------SRCC------------- 199

RAB8A P61006 --------TPD-------Q--QKRSSF----------------FRCVLL----------- 207

RAB8B Q92930 --------TEN-------R--SKKTSF----------------FRCSLL----------- 207

RAB9A P51151 ----------------------KPKPS----------------SSCC------------- 201

RAB9B Q9NP90 ----------------------GSKAG----------------SSCC------------- 201

RAB10 P61026 ---------GW-------K--S----------------------KCC------------- 200

RAB11A P62491 --------PPT-------T-EN--KPK----------------VQCCQNI---------- 216

RAB11B Q15907 --------PPT-------T-DGQKPNK----------------LQCCQNL---------- 218

RAB12 Q6IQ22 -------IPPE-------L--PPPRPH----------------VRCC------------- 244

RAB13 P51153 ------------------T--CDKKNT----------------NKCSLG----------- 203

RAB14 P61106 -----GRL-TS-------E-PQPQREG----------------CGC-------------- 215

RAB15 P59190 -------GKPE-------G--PAN-SS----------------KTCWC------------ 212

RAB17 Q9H0T7 ------------------K-GPA--RQ----------------AKCCAH----------- 212

RAB18 Q9NP72 ------------------G-Q-GGGAC----------------GGYCSVL---------- 206

RAB19 A4D1S5 --------LM----------AQGPSEK----------------THCTC------------ 217

RAB20 Q9NX57 ------------------H-KPPKRTR----------------SGCCA------------ 234

RAB21 Q9UL25 ------------------Q-AQT--SG----------------GGCCSSG---------- 225

RAB22A Q9UL26 ------------------Q-PSE--PK----------------RSCC------------- 194

RAB23 Q9ULC3 NGGDVINLRPN-K-QR--T-KKNRNPF----------------SSCSIP----------- 237

RAB24 Q969Q5 ------------------K-PNP--YF----------------YSCCHH----------- 203

RAB25 P57735 ---------AG-------Q-EPGPGEK----------------RACCISL---------- 213

RAB26 Q9ULW5 ----------------------REGRG----------------ASCCRP----------- 256

RAB27A P51159 ------------------Q-LSEEKEK----------------GACGC------------ 221

RAB27B O00194 ------------------L-DGEKPPE----------------KKCIC------------ 218

RAB28 P51157 ------------P-------MSRTVNPPRSS-------------MCAVQ----------- 221

RAB29 O14966 ------------------K-S----SS----------------WSCC------------- 203

RAB30 Q15771 --------LP----------GEGKSIS----------------YLTCCNFN--------- 203

RAB31 Q13636 ------------------P-TMQ--AS----------------RRCC------------- 194

RAB32 Q13637 ------------------E-TLRAENK----------------SQCC------------- 225

RAB33A Q14088 --------------------PQEANSK----------------TSCPC------------ 237

RAB33B Q9H082 --------------------KPEPKPA----------------MTCWC------------ 229

RAB34 Q9BZG1 ----------DDS-NL--Y-LTASKKK----------------PTCCP------------ 259

RAB35 Q15286 --------TK------------NSKRK----------------KRCC------------- 201

RAB36 O95755 ----------SPP-ET--Q-ESKRPSS----------------LGCC------------- 333

RAB37 Q96AX2 ----------------------SQKKR----------------SSCCSFM---------- 223

RAB38 P57729 --------------------STKVASC----------------SGCAKS----------- 211

RAB39A Q14964 ----VPNTVHS-------S-EEAVKPR----------------KECFC------------ 217

RAB39B Q96DA2 ----VPNVVHS-------S-EEVVKSE----------------RRCLC------------ 213

RAB40A Q8WXH6 -------------------------LQ----------------DLCCRTIVSC-----TP 205

RAB40B Q12829 -------------------------LQ----------------DLCCRAVVSC-----TP 205

RAB40C Q96S21 -------------------------LQ----------------DLCCRAIVSC-----TP 205

RAB41 Q5JT25 ------------------F-E-----E----------------SGNRSYC---------- 222

RAB42 Q8N4Z0 ----KTQIPRS-------P--SRKQHS----------------GPCQC------------ 218

RAB43 Q86YS6 ------------------------GEG----------------WGCGC------------ 212

RAB44 Q7Z6P3 ------------------A-PKRPPKR----------------FGCCS------------ 1021

RASEF Q8IZ41 --------------------SKKSPQM----------------KNCCNG----------- 740

DIRAS1 O95057 ---------------------------RVKG-------------KCTLM----------- 198

DIRAS2 Q96HU8 ---------------------------KLKG-------------KCVIM----------- 199

DIRAS3 O95661 ---------------------------KLLD-------------KCIIM----------- 229

ERAS Q7Z444 -----------------------QKATCHC--------------GCSVA----------- 233

HRAS P01112 --------------------------CMSCK--------------CVLS----------- 189

KRAS P01116 --------------------------CVKIK-------------KCIIM----------- 189

MRAS O14807 ----------------------RATGTHKL--------------QCVIL----------- 208

NKIRAS1 Q9NYS0 ------------S--------------------------------EN------------- 192

NKIRAS2 Q9NYR9 ------------L--------------------------------DG------------- 191

NRAS P01111 --------------------------CMGLP--------------CVVM----------- 189

RALA P11233 ------------A-------K-----RIRE--------------RCCIL----------- 206

RALB P11234 ------------K-------K-----SFKE--------------RCCLL----------- 206

RAP1A P62834 -----------------------EKKKPKKK-------------SCLLL----------- 184

RAP1B P61224 -----------------------PGKARKKS-------------SCQLL----------- 184

RAP2A P10114 -----------------------DKDDPCCS-------------ACNIQ----------- 183

RAP2B P61225 -----------------------NGDEGCCS-------------ACVIL----------- 183

RAP2C Q9Y3L5 -----------------------EKQDQCCT-------------TCVVQ----------- 183

RASD1 Q9Y272 PFARRPS-VHSDLMYIREK-ASAGSQAKDKE-------------RCVIS----------- 281

RASD2 Q96D21 PFARRPS-VNSDLKYIKAK-VLREGQARERD-------------KCTIQ----------- 266

RASL10A Q92737 ---------------------------LHPA-------------RCSLM----------- 203

RASL10B Q96S79 ---------------------------LRRN-------------RCAIM----------- 203

RASL11A Q6T310 ------------L-------KRRFKQALS----------------PKVKAPSA------- 240

RASL11B Q9BPW5 ------------L-------KRRFKQALS----------------AKVRTVTS------- 247

RASL12 Q9NYN1 ------------L-------TARHGLA-S----------------CTFNTLST------- 226

RERG Q96A58 -----------------------VKQAIN----------------KMLTKISS------- 199

RERGL G5EA41 ------------------------------------------------------------

RHEB Q15382 ------------------------------S-------------SCSVM----------- 184

RHEBL1 Q8TAI7 ------------------------------R-------------RCHLM----------- 183

RIT1 Q92963 ------------K-------SPFRKKKDSVT----------------------------- 219

RIT2 Q99578 ------------K-------GSLKKKRENMT----------------------------- 217

RRAS P10301 -----------------------RKKGGGC--------------PCVLL----------- 218

RRAS2 P62070 -----------------------EKDKKGC--------------HCVIF----------- 204

GEM P55040 ------------IVAKNNK-NMAFKLK--SK-------------SCHDLSVL-------- 296

REM1 O75628 ------------LTARSAR-RRALKAR--SK-------------SCHNLAVL-------- 298

REM2 Q8IYK8 ------------LVPRNAK---FFKQR--SR-------------SCHDLSVL-------- 340

RRAD P55042 ------------IVARNSR-KMAFRAK--SK-------------SCHDLSVL-------- 308

CDC42 P60953 -----------------------------------------RCVLL-------------- 191

RAC1 P63000 -----------------------------------------KCLLL-------------- 192

RAC2 P15153 -----------------------------------------ACSLL-------------- 192

RAC3 P60763 -----------------------------------------KCTVF-------------- 192

RHOA P61586 -----------------------------------------GCLVL-------------- 193

RHOB P62745 -----------------------------------------GCINCCKVL---------- 196

RHOBTB1 O94844 -LLQAPFLPPKAP-------PPVIKIPECPSMGTNEAACLLDNPLCADVLFI-------- 271

RHOBTB2 Q9BYZ6 -LLQAPFLPPKPP-------PPIIVVPDPPSSSEECPAHLLEDPLCADVILV-------- 271

RHOC P08134 -----------------------------------------GCPIL-------------- 193

RHOD O00212 -------------------------------------------QGFCVVT---------- 210

RHOF Q9HBH0 ---------------------------------------------LCLLL---------- 211

RHOG P84095 -----------------------------------------SCILL-------------- 191

RHOH Q15669 -------------------------------------------INECKIF---------- 191

RHOJ Q9H4E5 -----------------------------------------S---CCSII---------- 214

RHOQ P17081 -----------------------------------------RCINCCLIT---------- 205

RHOU Q7L0Q8 -KMKNLSKSW-------------------------------WKKYCCFV----------- 258

RHOV Q96L33 -GVRTLSRCR-------------------------------WKKFFCFV----------- 236

RND1 Q92730 -LLHLPSRSE---------------------LI--SSTFKKEKAKSCSIM---------- 232

RND2 P52198 -SAQLSGRPD---------------------RGN-EGEIHKDRAKSCNLM---------- 227

RND3 P61587 -ISHMPSRPE---------------------LSAVATDLRKDKAKSCTVM---------- 244

RAN P62826 EVAQTTALPDE-------------D----------------------------------- 213

RHOT1 Q8IXI2 -CIKALTR--------------IFKISDQDNDGTLNDAELNFFQRICFNTPLA------P 223

RHOT2 Q8IXI1 -CAQALTR--------------IFRLSDQDLDQALSDEELNAFQKSCFGHPLA------P 223

IFT22 Q9H7X7 ------------------------------------------------------------

RABL2A Q9UBK7 -EEDVPDQEQS-------S--SIETPS----------------EEVASPHS--------- 228

RABL2B Q9UNT1 -EEDVPDQEQS-------S--SIETPS----------------EEAASPHS--------- 228

RABL3 Q5HYI8 ------------------------------------------------------------

RABL6 Q3YEC7 EMMEARSRGHASPLAANGQSPSPGSQSPVVPAGAVST---GSSSPGTPQPAPQ------- 322

RRAGA Q7L523 FLVISHYQC-K---------EQRDVHR---------------FEKISNIIKQF--KLSCS 243

RRAGB Q5VZM2 FLVISHYQC-K---------EQRDAHR---------------FEKISNIIKQF--KLSCS 304

RRAGC Q9HB90 KIYIAT--D-S---------SPVDMQS---------------YELCC-DMIDVVIDVSCI 298

RRAGD Q9NQL2 KIYIAT--D-S---------TPVDMQT---------------YELCC-DMIDVVIDISCI 299

**Sequence alignment of Ras small GTPase superfamily.** Multiple sequence alignment of the G domain Ras superfamily members using Uniprot (the uniport accession codes can be taken from this figure). Based on the marked areas that are shown in Figure 1-figure supplement 1, cysteines that are within the reach of the warhead of the eda linker are highlighted in gray. In contrast, regions that are also within reach of the bda linker are highlighted in blue. The cysteines located within these regions are marked in red. Only ~ 7% of the GTPases have cysteines within the P-loop and are therefore potential off-targets of the designed nucleotide derivatives.
