# Supplementary material for: Targeting oncogenic KRasG13C with nucleotide-based covalent inhibitors
Source: eLife. 2023 Mar 27;12:e82184. doi: 10.7554/eLife.82184 (PMC10042540; doi:10.7554/eLife.82184)
Supplement: Supplementary file 2. — Overview of calculated pKa values of the KRasG12C and G13C mutants in the presence (+) and absence (-) of GDP. The linker in case of the G13C mutants was removed so that all featured structures had GDP and a free cysteine in its active center. The pKa calculations showed that both cysteines at position 12 and 13 have similar pKa values indicating that also position 13 should generally be addressable by covalent warheads. [file elife-82184-supp2.docx]

**pKa calculations.** Overview of calculated pKa values of the KRasG12C and G13C mutants in the presence (+) and absence (-) of GDP. The linker in case of the G13C mutants was removed so that all featured structures had GDP and a free cysteine in its active center. The pKa calculations showed that both cysteines at position 12 and 13 have similar pKa values indicating that also position 13 should generally be addressable by covalent warheads.

|  | **KRas structure** | | **pKa Cys (12/13)** | | |
| --- | --- | --- | --- | --- | --- |
| **KRas Mutant** | **PDB** | **Ligand** | **+** | **-** | **Δ** |
| G13C | 7ok3 | GDP (from edaGDP) | 9.85 | 8.85 | 1.0 |
|  | 7ok4 | GDP (from bdaGDP) | 9.85 | 8.85 | 1.0 |
| G12C | 4ldj | GDP | 10.65 | 9.35 | 1.3 |
|  | 4l8g | GDP | 10.35 | 9.05 | 1.3 |
